# Long-term forecasting and evaluation of medicine consumption for the ATC class H with a focus on thyroid hormones in OECD countries using ARIMA models

**DOI:** 10.1007/s00210-025-03930-5

**Published:** 2025-03-03

**Authors:** Lilly Josephine Bindel, Roland Seifert

**Affiliations:** Institute of Pharmacology, Hannover Medical School, D-30625 Hannover, Germany

**Keywords:** Pharmaceutical consumption, Surveillance, Forecast, ARIMA, Systemic hormones, OECD, Thyroid hormones, Levothyroxine, Prescribing behaviour, Irrational, Rational, Countries, Cultural differences

## Abstract

Thyroid hormones are among the most prescribed medicines. In many countries, there are shortages combined with evidence of overuse and irrational prescribing. An analysis was conducted for ATC class H with a focus on thyroid hormones for OECD countries. This study aims to evaluate prescribing behaviours, forecast long-term developments and promote rational prescribing behaviour. The ARIMA(2,1,2) (autoregressive integrated moving average) model successfully predicted the future for 30 OECD countries and the non-OECD country Croatia until 2040. An upward trend is forecast for 18 countries, including Austria (+ 5.7%), Chile (+ 220.0%), Czechia (+ 52.8%), Denmark (+ 15.6%), Estonia (+ 87.8%), Greece (+ 238.7%), Hungary (+ 5.7%), Iceland (+ 18.6%), Italy (+ 42.9%), Latvia (+ 83.7%), Lithuania (+ 131.2%), Portugal (+ 106.7%), Slovakia (+ 182.1%), Slovenia (+ 57.4%), Spain (+ 162.8%), Turkey (+ 168.7%), the United Kingdom (+ 138.1%) and Croatia (+ 190.6%). A downward trend is forecast for 13 countries, including Australia (-3.4%), Belgium (-38.8%), Canada (-95.1%), Costa Rica (-79.5%), Finland (-14.7%), France (-100.0%), Germany (-16.4%), Israel (-21.6%), Korea (-100.0%), Luxembourg (-100.0%), the Netherlands (-35.9%), Norway (-23.6%) and Sweden (-43.6%). The reliability and accuracy of the forecasts varies, being influenced by data quality. While a downward trend is favoured, an upward trend is seen as problematic. Increasing trends predominate in Southern and Eastern Europe and Latin America, while decreasing trends predominate in Northern and Western Europe and the Asia–Pacific Region. Some external factors affect all countries, like an increasing prevalence of thyroid disease. There is evidence of cultural influences on prescribing behaviour. While there is evidence of inappropriate use in countries where prescriptions are predicted to increase, measures to restrict the use of thyroid hormones are more common in countries with a recently reported and predicted declining trend.

**Supplementary Information:**

The online version contains supplementary material available at 10.1007/s00210-025-03930-5.

## Introduction

Thyroid hormones, particularly levothyroxine, rank among the most frequently prescribed medications in numerous countries (Ludwig et al. 2024; MHRA 2021). These medicines fall under the ATC H classification, which includes systemic hormonal preparations excluding sex hormones and insulins, with thyroid hormones constituting a significant proportion of this category (Table 1). Within this ATC classification, thyroid hormones (ATC H03) dominate the ATC H group, while levothyroxine itself accounts for more than 50% of ATC H consumption, reflecting its importance and high prescription volume. Since these medicines are primarily used to manage chronic conditions, ensuring an uninterrupted supply is vital for patients. Any disruption in access to necessary thyroid medications heightens the risk of adverse health outcomes, potentially worsening symptoms for patients with thyroid disorders (MHRA 2021; Alexander et al. 2017). This can also lead to increased healthcare costs due to the management of complications, lost productivity due to illness and higher defined daily dose (DDD) costs when switching to alternative medications or brands (Fox et al. 2014).

**Table 1:** Breakdown of the consumption within systemic hormones (ATC H), exemplarily on countries with publicly available consumption data (WHO 2024)

Country	Consumption of ATC H	Consumption of ATC H3 (thyroid hormones)	Consumption of ATC H03AA01 (Levothyroxine)	Percentage of Levothyroxine on ATC H	Year	Source	Database link (last accessed: December 1 2024)
Australia	3´727´662 total prescriptions	2´339´506 total prescriptions	2´060´160 total prescriptions	55%	2023	PBS 2024	https://www.pbs.gov.au/info/statistics/expenditure-prescriptions/pbs-expenditure-and-prescriptions
Denmark	37.9 DDD per 1000 inhabitants per day	22.4 DDD per 1000 inhabitants per day	20.2 DDD per 1000 inhabitants per day	53.30%	2022	The Danish Health Data Authority 2024	https://www.medstat.dk/en
Estonia	28.3 DDD per 1000 inhabitants per day	19.3 DDD per 1000 inhabitants per day	18.04 DDD per 1000 inhabitants per day	63.75%	2023	ANDMEBAAS 2024	https://statistika.tai.ee/pxweb/en/Andmebaas/Andmebaas__06Ravimistatistika/
Finland	55.43 DDD per 1000 inhabitants per day	36.31 DDD per 1000 inhabitants per day	35.8 DDD per 1000 inhabitants per day	64.59%	2021	FIMEA 2024b	https://fimea.fi/en/databases_and_registeries/consumption
Germany	2´383.4 DDD in million	1´903.4 DDD in million	1´458.3 DDD in million	61.19%	2022	Ludwig et al. 2024	-
Latvia	27.31 DDD per 1000 inhabitants per day	17.53 DDD per 1000 inhabitants per day	15.47 DDD per 1000 inhabitants per day	56.65%	2018	State Agency of Medicines 2019	https://www.zva.gov.lv/en/news-and-publications/publications/statistics-medicines-consumption
Netherlands	199´280′000 DDD	131´743´800 DDD	121´210´700 DDD	60.82%	2023	GIP 2024	https://www.gipdatabank.nl/
Norway	81´391´112 DDD	51´145´416 DDD	46´968´308 DDD	51.39%	2020	Norwegian Prescription Database 2024	https://www.norpd.no/Prevalens.aspx

Alarmingly, thyroid hormone supplies have experienced multiple shortages in many countries, with some facing ongoing shortages (Table 2). Paradoxically, there are also numerous reports suggesting that thyroid hormone replacement therapy is being overprescribed across several regions (Ludwig et al. 2024; Brito et al. 2021; Backman 2023; Der Arzneimittelbrief 2021; Negro et al. 2024). A significant rise in thyroid hormone consumption has been observed in many countries (Brito et al. 2021; Ludwig et al. 2024). Such irrational prescribing practices do not only increase the risk of supply bottlenecks but also impose an unnecessary substantial financial burden on healthcare systems.

**Table 2:**
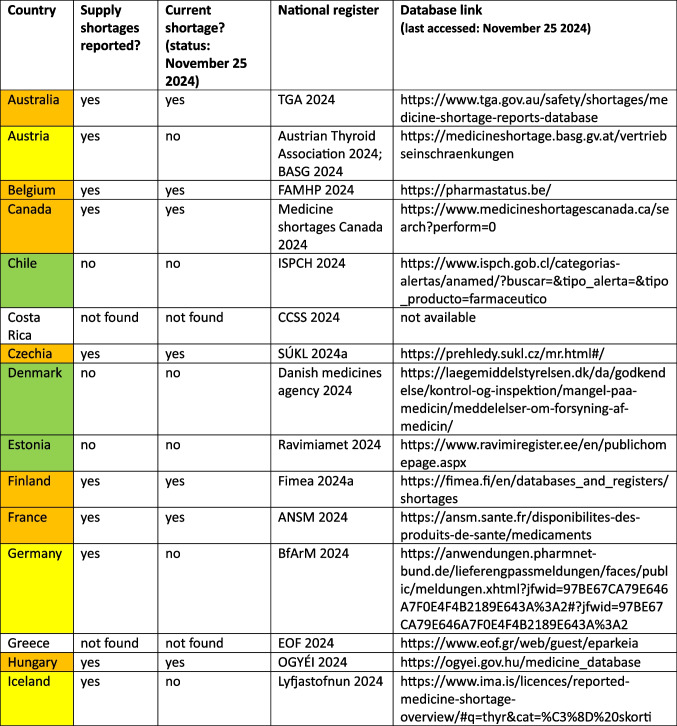

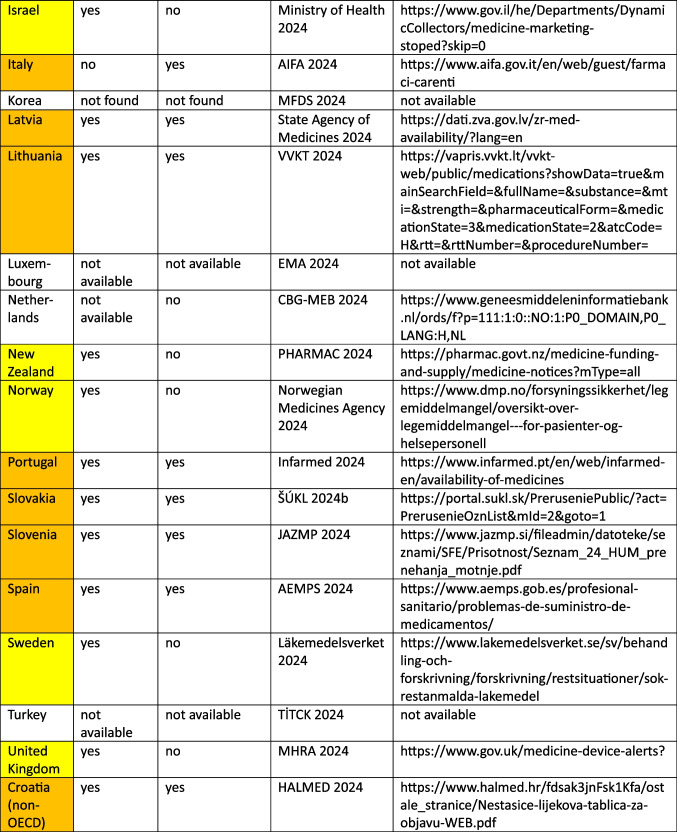
Reported supply shortages for thyroidal hormones (ATC H03) among OECD countries and the non-OECD country Croatia. Reports are based on national registers. Information is given whether any supply shortages emerged within the last 5 years, and if yes, if there is a current supply shortage (status: November 25 2024). In some cases, databases were non-existent, as for Luxembourg, or no database was found, e.g. for Greece, Korea or the Netherlands. Green colour highlights countries where no supply shortage was found. Yellow colour highlights countries where supply shortages were reported in the past, while preparations are available recently. Orange colour highlights countries that experience a supply shortage recently

This study aims to evaluate prescribing behaviours for systemic hormones across OECD countries by modelling trends and forecasting long-term developments using the ARIMA model. The objective is to identify reasons for rational versus irrational use, highlighting countries with favourable trends and those facing problematic developments. Additionally, we estimate the long-term future demand for each country, which can aid in strategic demand planning. A key goal of this research is to encourage rational prescribing practices.

Previous studies conducted by our group have already highlighted irrational prescribing patterns in other medicine categories, such as antibacterial agents and other commonly used medications (Bindel and Seifert 2024, 2025). To our knowledge, this study represents the first comprehensive analysis of thyroid and systemic hormone utilization across multiple countries over an extended period, with projections for future trends. This analysis is particularly timely, given the interconnected nature of global markets where demand and supply dynamics extend beyond national borders. In the context of ongoing supply shortages, it is clear that no country operates in isolation.

## Materials and methods

### Setting and data

The analysis focuses on national consumption data of the medicine class ATC H (systemic hormonal preparations, exclusive sex hormones and insulins). Data is based on the OECD data explorer (OECD 2024a), spanning from 1980 to 2022. Data is given in DDD prescriptions per 1000 inhabitants per day (DID).

For 30 OECD countries and the non-OECD country Croatia, consumption data was available, including Australia, Austria, Belgium, Canada, Chile, Costa Rica, Czechia, Denmark, Estonia, Finland, France, Germany, Greece, Hungary, Iceland, Israel, Italy, Korea, Latvia, Lithuania, Luxembourg, the Netherlands, New Zealand, Norway, Portugal, Slovakia, Slovenia, Spain, Sweden, Turkey, the United Kingdom and Croatia (non-OECD).

### Selection of method for time series analysis

The ARIMA model, which stands for “Auto Regressive Integrated Moving Average”, was selected for its versatility in handling various components of a time series, including autoregressive (AR) behaviour, differencing (I), and moving average (MA) effects (Bindel and Seifert 2025). This model is particularly well-suited to data such as ours, where past observations influence future values. ARIMA models are widely used in forecasting medicine consumption trends (Hyndman and Athanasopoulos 2021).

It comprises three key components. The autoregressive (AR) part accounts for the influence of previous observations on the current value. The differencing (I) component helps in making the series stationary by removing trends or seasonality. Finally, the moving average (MA) component captures the dependency between an observation and past forecast errors (Nau 2020; Box et al. 2015).

The general form of an ARIMA(*p,d,q*) model is expressed as follows:

$$\widehat{y}t = \mu + \phi 1yt-1 +\dots +\phi p yt-p-\theta 1et-1-\dots -\theta qet-q$$

In this equation, $\widehat{y}t$ represents the value of the series at time, $t, p$ refers to the number of autoregressive terms, *d* is the degree of differencing applied to make the series stationary, and *q* is the number of lagged forecast errors in the moving average model. The error term $et$ captures the random shocks that cannot be explained by the model (Nau 2020; Box et al. 2015).

### Data preprocessing for time series analysis

A precondition for ARIMA modelling is data stationarity (Hyndman and Athanasopoulos 2021). Autocorrelation function (ACF) and partial autocorrelation function (PACF) tests were applied on each medicine in the dataset, revealing significant trends that confirmed the presence of non-stationarity in the original time series (Figs. S32-S63), which is also visible within the plotted development (Figs. S1-S31). Consequently, differencing was necessary to remove these trends, ensuring the necessary stationarity for accurate predictions. Differencing allows the ARIMA model to capture temporal dependencies without the distortion caused by persistent trends, enabling more reliable forecasting.

### Determination of parameters

The ARIMA model is shaped by its three parameters *p, d* and *q.* The autoregressive term *p* defines how many past observations influence the current prediction, with higher values increasing reliance on historical data and potentially adding complexity. The differencing order *d* helps achieve stationarity by removing trends in the data, but excessive differencing may lead to over-fitting and loss of important structure. Finally, the moving average term *q* determines how many past forecast errors affect current predictions, where larger values mean more past errors are incorporated, potentially raising the model's complexity (Hyndman and Athanasopoulos 2021; NIST/SEMATECH 2012).

Optimal values for *p*, *d*, and *q* were identified post-preprocessing, based on an analysis of the ACF and PACF plots, which highlighted significant lag structures in the data. The differencing parameter *d* = 2 was selected to remove the trend and achieve stationarity in the time series. It had to be differentiated twice because after the first differentiation there was still a trend (Figs. S1-S31 and S63-S96), which manifested itself in unrealistic predictions and poor fit metrics, in particular a stationary R-squared close to zero. The autoregressive term *p* and the moving average term *q* were determined by analysing the autocorrelation function (ACF) and partial autocorrelation function (PACF) plots. Further explanations and graphical illustrations of the modelling process can be found in the supplement.

The ARIMA(1,2,1) model emerged as the most suitable variant for our time series analysis. This model is particularly effective for time series that exhibit a strong trend over time. It employs second-order differencing (Figs. S94-S124) to achieve stationarity, which is essential when the data shows a more persistent trend that is not fully addressed by first-order differencing (Figs. S1-S31). The inclusion of both autoregressive (AR) and moving average (MA) components allows the model to account for dependencies in the data as well as random fluctuations, resulting in improved predictive accuracy. By combining these elements, the ARIMA(1,2,1) model offers a robust approach for capturing complex patterns, though it may increase the risk of overfitting due to its higher differencing and parameter complexity (Nau 2020; Box et al. 2015). Literature research confirms that this specific ARIMA model has demonstrated effectiveness in forecasting long-term trends across several population-level studies (Perone 2020; Thabani 2019).

### Model adjustments

The ARIMA models were developed using SPSS, utilizing data spanning from 1980 to 2022 for model construction. However, structural changes in definitions and methodological procedures were observed in several countries, resulting in time breaks and limiting the comparability of absolute values. A summary of these variations, including time breaks and the range of available years, is provided in Table S1. More detailed information can be found in the “Methods and Sources” section from the OECD data explorer (OECD 2024b).

Since time breaks cause level shifts in consumption data, they can significantly impact forecast reliability. To address this, automatic outlier detection was applied for countries with documented time breaks (Table S1). For countries where no such breaks were identified according to the OECD data (OECD 2024a), no automatic outlier adjustments were made.

ARIMA(1,2,1) models were successfully developed for 30 of 31 OECD countries and Croatia. For the OECD country New Zealand, the limited number of data points prevented model creation. While more complex models might better capture underlying regularities and offer more precise forecasts—indicated by higher stationary R-squared and R-squared values—there is a significant risk of overfitting, where random fluctuations are mistakenly identified as patterns. Given the relatively limited dataset, the model's capacity is restricted. Therefore, greater emphasis should be placed on the reliability of trends rather than the precise prediction of specific DDD values in long-term periods, while forecast for a shorter time period are considered to be more reliable. An overview of predicted values for future consumption is provided in Table S5.

Forecasts were extended up to the year 2040, providing long-term projections of consumption trends for each country (Table S5). Additionally, confidence intervals were calculated to illustrate the uncertainty of these forecasts, indicating a range within which future consumption values are likely to fall.

### Assessment of fit metrics

To assess the performance of the ARIMA models, several fit metrics were used. This includes stationary R-squared, R-squared, RMSE (Root Mean Squared Error), MAPE (Mean Absolute Percentage Error), MAE (Mean Absolute Error) and normalised BIC (Bayesian Information Criterion) (Hyndman and Athanasopoulos 2021; NIST/SEMATECH 2012; Bindel and Seifert 2025).

The Stationary R-squared and R-squared metrics assess how well the model explains the variance in the data. Stationary R-squared is particularly useful for differenced data, as it indicates how well the model fits on a stationary scale. Values of 0.65 and above are considered a good fit, while values between 0.4 and 0.64 are moderate, and anything below 0.4 is poor. On the other hand, R-squared measures the model’s overall explanatory power, with values above 0.85 considered good, between 0.6 and 0.84 as moderate and below 0.6 as poor.

RMSE and MAE are absolute error measures that assess prediction accuracy. RMSE, which emphasizes larger deviations, is more sensitive to outliers, while MAE provides the average of absolute deviations. Ideally, both metrics should approach zero. However, their interpretation depends on the scale of the data, making context decisive for evaluating whether an RMSE or MAE value is good, moderate or poor.

MAPE and MaxAPE quantify forecast accuracy as percentages, making them useful for comparing models across different scales. MAPE values under 6% indicate a good fit, between 7 and 20% are moderate, while values exceeding 20% are poor. MaxAPE focuses on the worst-case relative error, with values below 15% regarded as good, 16% to 40% as moderate and anything higher as poor.

MaxAE measures the largest single deviation observed in the model’s predictions, highlighting its worst-case performance. Lower values are preferred, indicating that the model avoids significant prediction errors.

Finally, the Normalized BIC assesses model fit in relation to complexity. Lower BIC values indicate a better balance between accuracy and simplicity, as the BIC penalizes models with unnecessary parameters to prevent overfitting.

**Fig. 1:**
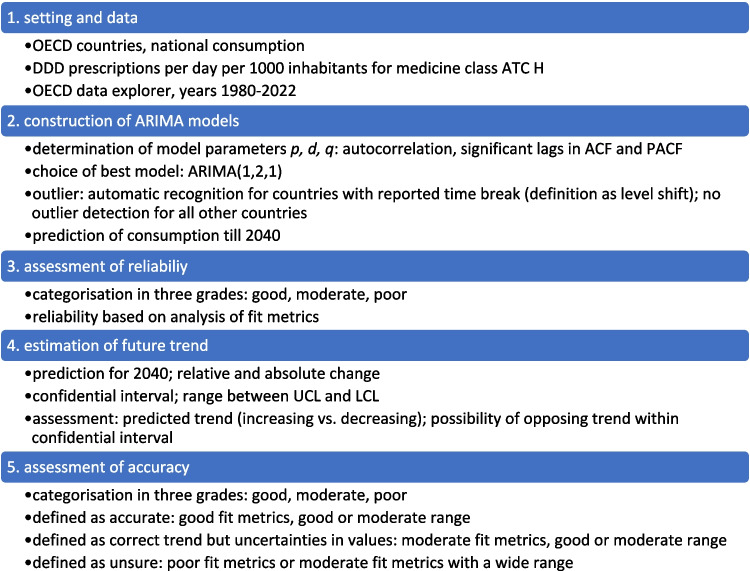
Methodical approach for time series analysis in SPSS with the ARIMA-model to predict futural development of consumption for the ATC group H for OECD countries

Together, these metrics provide a comprehensive evaluation of both the accuracy and efficiency of the ARIMA models, enabling a balanced assessment of model performance across different countries and time series data. The methodological procedure is illustrated in Fig. 1 and the classification of the fit metrics is summarized in Table S2.

## Results and discussion

### Historical development of the medicine class systemic hormones (ATC H)

Consumption data for OECD countries is available for several years for the medicine class systemic hormones, ATC H (OECD 2024b; WHO 2024) (Table S1). Therefore, a foundation for understanding general trends and variations across individual countries is offered. While direct comparison of absolute values is limited due to differences in data collection methodologies, assessing trends within each country remains viable. Therefore, this analysis focuses on changes in consumption trends, classifying countries based on overall increases, decreases or stabilization over time, as well as on specific patterns observed in recent years.

A broad review of data from the initial to the final observation points reveals a general increase in systemic hormone consumption across most OECD countries. Countries with this upward trend include Australia, Belgium, Canada, Chile, Costa Rico, Czechia, Denmark, Estonia, Finland, France, Germany, Greece, Hungary, Iceland, Israel, Italy, Korea, Latvia, Lithuania, the Netherlands, Norway, Portugal, Slovakia, Slovenia, Spain, Sweden, Turkey, United Kingdom and Croatia. Only a few countries reported a decrease over the observed period, including Austria, Luxembourg and New Zealand.

Notably, countries that exhibit a decreasing trend tend to have fewer data points, meaning that reports are only covering a short period of time and do not give insight in further post. Specifically, data is available for Austria from 2010 to 2021, for Luxembourg from 2005 to 2022, and for New Zealand from 2018 to 2022, although here the values are only estimations. In contrast, countries showing an upward trend often have longer observation periods, for example Czechia from 1980 to 2021 or Germany from 1986 to 2021.

Overall, the general pattern showed an increase in consumption from the first to the last data point. Exceptions with a decreasing trend can be attributed to a short observation period, as seen in Austria, reported time breaks, as in Greece, or strong distortions, such as recent spikes in France or Lithuania. Therefore, a ranking by relative or absolute changes may be misleading because of the large differences in the time periods covered as well as frequent and strong time breaks leading to distortions. Literature research confirms an overall increasing trend over the decades for many countries, reasoned by an increase in routine diagnostics of TSH (thyroid-stimulating hormone) measurements and a lowered treatment initiation threshold (Schröder and Seifert 2024; Jonklaas and DeSale 2020; Taylor et al. 2018; Turunen et al. 2021), along with an increasing prevalence in population (Jonklaas and DeSale 2020; Taylor et al. 2018).

In the most recent years of observation, two different patterns are emerging. Some countries are continuing their increasing trend, including Chile, Croatia, Czechia, Denmark, Estonia, Greece, Italy, Latvia, Portugal, Slovenia, Spain and Turkey. Notably, other countries experienced a changing trend by reaching a plateau or even starting to decrease. A stabilization is shown for Australia, Belgium, Finland, Germany, Hungary, Iceland, Israel, the Netherlands, New Zealand, Norway and the United Kingdom, while a decrease is reported for Austria, Canada, Sweden, Korea and Luxembourg. An uncertain development due to strong fluctuations within the last years is present for Costa Rica, France, Lithuania and Slovakia.

The appearing change in behaviour was noted in several countries, following a certain course: increasing first, then followed by a stabilisation or even a down-trend (Jonklaas and DeSale 2020). Contributing factors may include the saturation of diagnostics, where most cases of hypothyroidism have been detected, and the implementation of measures to reduce potential overuse, such as stricter guidelines and critical medical discourse (Ludwig et al. 2024). Thus, countries with a continued increase may still be in the early stages of this progression.

Countries with low dynamic changes in consumption tend to exhibit consistent prescribing practices, whereas strong increases suggest shifts that could indicate unstable prescribing practices or potential overuse. Low consumption may indicate underdiagnosis or untreated cases, and a moderate increase may be less concerning compared to similar increases in high-consumption countries. Conversely, a high consumption is indicative for potential overuse.

### Countries with an increasing trend: prediction and assessment of reliability

To estimate a potential future trend, a time series analysis using the ARIMA(1,2,1) model was conducted. Beside the trend of the predicted values, the range between UCL and LCL (providing the confidential interval), as well as the fit metrics for the ARIMA model have to be considered to assess the reliability of the forecasts. Countries were categorized by their predicted trends, including potential alternative trends indicated by the confidence intervals as well as absolute and relative changes in the forecasted consumption in 2040.

**Fig. 2:**
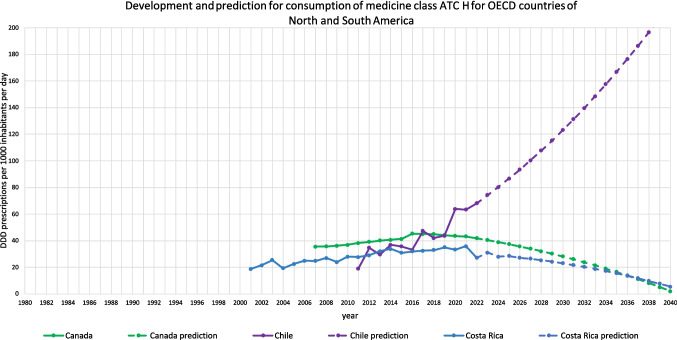
Overview of historical development and future predictions of the analysed OECD countries of North and South America with the ARIMA(1,2,1) model

**Fig. 3:**
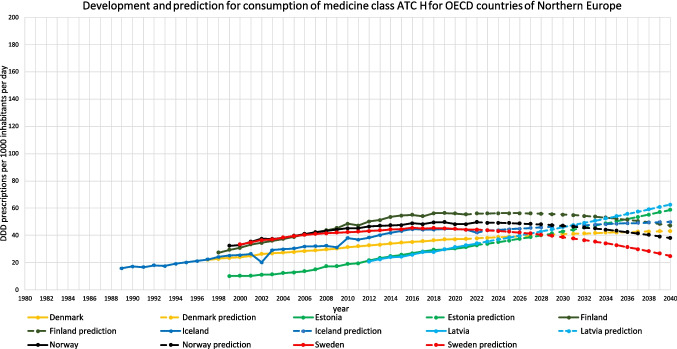
Overview of historical development and future predictions of the analysed OECD countries of Northern Europe with the ARIMA(1,2,1) model

**Fig. 4:**
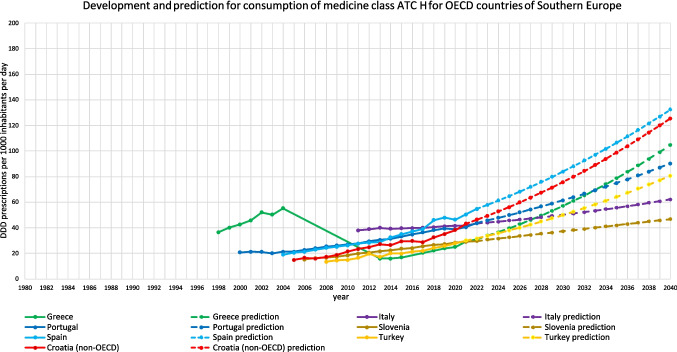
Overview of historical development and future predictions of the analysed OECD countries of Southern Europe and the non-OECD country Croatia with the ARIMA(1,2,1) model

**Fig. 5:**
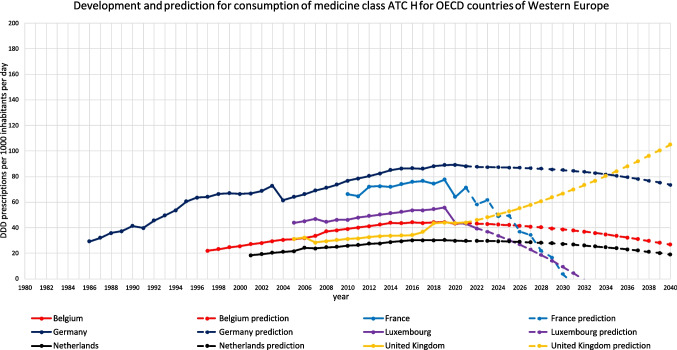
Overview of historical development and future predictions of the analysed OECD countries of Western Europe with the ARIMA(1,2,1) model

**Fig. 6:**
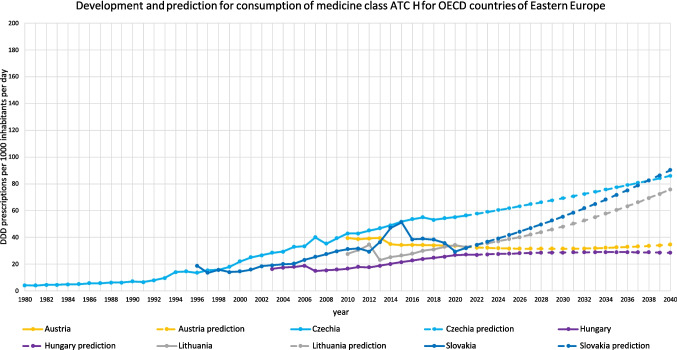
Overview of historical development and future predictions of the analysed OECD countries of Eastern Europe with the ARIMA(1,2,1) model

For 18 countries, an increasing trend is depicted (Table 3, Figs. 2, 3, 4, 5, 6). This includes Chile (+ 220.0%, reaching 217.9 DDD prescriptions per 1000 inhabitants per day by 2040), Austria (+ 5.7%, reaching 34.7 DDD), Czechia (+ 52.8%, reaching 86.1 DDD), Denmark (+ 15.6%, reaching 43.2 DDD), Estonia (+ 87.8%, reaching 58.5 DDD), Greece (+ 238.7%, reaching 104.7 DDD), Hungary (+ 5.7%, reaching 28.5 DDD), Iceland (+ 18.6%, reaching 49.9 DDD), Italy (+ 42.9%, reaching 62.2 DDD), Latvia (+ 83.7%, reaching 62.6 DDD), Lithuania (+ 131.2%, reaching 75.8 DDD), Portugal (+ 106.7%, reaching 90.3 DDD), Slovakia (+ 182.1%, reaching 90.3 DDD), Slovenia (+ 57.4%, reaching 46.7 DDD), Spain (+ 162.8%, reaching 132.4 DDD), Turkey (+ 168.7%, reaching 80.6 DDD), the United Kingdom (+ 138.1%, reaching 105.0 DDD) and Croatia (+ 190.6%, reaching 125.5 DDD).

**Table 3:**
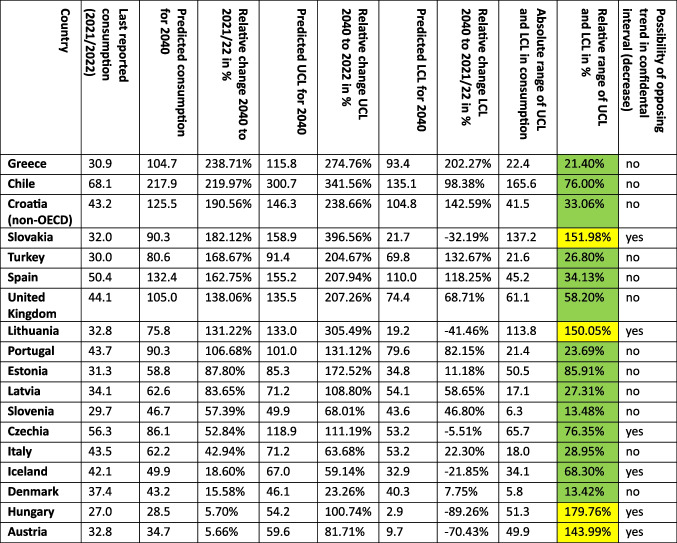
Overview about predictions for the OECD countries with a predicted increase. Countries are sorted descending by their relative change. The range between LCL and UCL for the prediction for 2040 is divided into a good fit (green colour) with a range under 100%, a moderate fit (yellow colour) with a range between 100–200%, and a poor fit (orange colour) with a range exceeding 200%

An opposing trend, offered by the LCL and aiming towards a stabilizing or decreasing trend, is possible for Czechia (LCL of −5.5%, predicting 53.2 DDD), Austria (−70.4% with 9.7 DDD), Hungary (−89.3% with 2.9 DDD), Iceland (−21.9% with 32.9 DDD), Lithuania (−41.5% with 19.2 DDD) and Slovakia (−32.2% with 21.7 DDD). No opposing trend is enabled for Chile, Denmark, Greece, Italy, Latvia, Portugal, Slovenia, Spain, Turkey, the United Kingdom and Croatia.

The relative changes range from 5.7% for Austria to 238.7% for Greece. While Austria has the smallest absolute increase with + 1.9, Chile has the highest absolute increase with + 149.8. Within the countries with a predicted increase, Austria has the smallest predicted consumption with 34.7, while Chile has the largest prescription volume with 217.9. As higher the predicted increase, the more dynamic the curve but the more questionable is the reliance whether the forecast is correct. The stronger the increase, the more problematic the development tends to be. The smaller the increase, the less critical the trend can be interpreted. Due to the uninterrupted increase, a continuing increase is plausible in the model. However, the extent to which the prediction appears realistic must be considered critically.

The reliability of each ARIMA(1,2,1) model is further evaluated by examining fit metrics (Table S3). A robust model fit is expected to have high R-squared and stationary R-squared values, paired with low MAPE and BIC scores. Conversely, poor fit models often show low R-squared metrics with higher MAPE and RMSE values, indicating the model’s limited ability to capture data dynamics accurately. A good model fit is assessed for Denmark, Estonia, Hungary, Latvia, Slovenia, Spain, Portugal and Turkey. Moderate fit metrics include the ARIMA models of Czechia, Iceland, Slovakia, the United Kingdom and Croatia. In contrast, poor fit metrics are exhibited in Austria, Chile, Greece, Italy and Lithuania. Reasons for a poor model adjustment can be diverse. A very important factor is the data quality. If there are only a few years available, like in Austria, or many distortions or fluctuations, like in Chile, the model can’t be built that precisely as if the data quality is very high. Furthermore, dynamic or unsteady developments leads to an uncertain prediction, because there might be strong external influences that can change over long-terms. When there is present a good model fit, the prediction can be considered as reliable. The worse the fit metrics, the more limited is the reliability of the forecast.

The preciseness of the model can be analysed by the range between the UCL and the LCL (Table 3). A narrow range is defined with a range under 100.0%, while a moderate is apparent with a range between 100.0–200.0%, and a range exceeding 200.0% being considered as large. A narrow range is depicted for Chile (76.0%), Czechia (76.4%), Denmark (13.4%), Estonia (85.9%), Greece (21.4%), Iceland (68.3%), Italy (29.0%), Latvia (27.3%), Portugal (23.7%), Slovenia (13.5%), Spain (34.1%), Turkey (26.8%), the United Kingdom (58.2%) and Croatia (33.1%). A moderate range is exhibited for Austria (144.0%), Hungary (179.8%), Lithuania (150.1%) and Slovakia (152.0%). There is no country with a range over 200.0%. The range offers an assessment of the uncertainty of the model forecast and the variety of possible developments. Countries with a narrow range offer a quite secure forecast, while a moderate range depicts more uncertainties and the possibility for differing developments than the predicted consumption.

Countries that have shown a decreasing trend over the whole period analysed may still be predicted to increase in the future. This can be due to recent trend reversals or time breaks that distort the trajectory. The most notable example is Greece, which shows an overall decreasing trend, but is predicted to show a sharp increase in the coming years. After a long period without published data, Greece experienced a time break in 2013, with consumption levels significantly lower than the last available data point before. It remains uncertain whether this sharp decline reflects an actual reduction in use or is fully caused by a change in data collection methodology. Moreover, Greece shows an upward trend when the earlier and later periods of available data are analysed separately. These circumstances create the illusion of a misleading downward trend in the past, a misrepresentation that is correctly recognised by the forecasting model, which predicts a future increase.

**Fig. 7:**
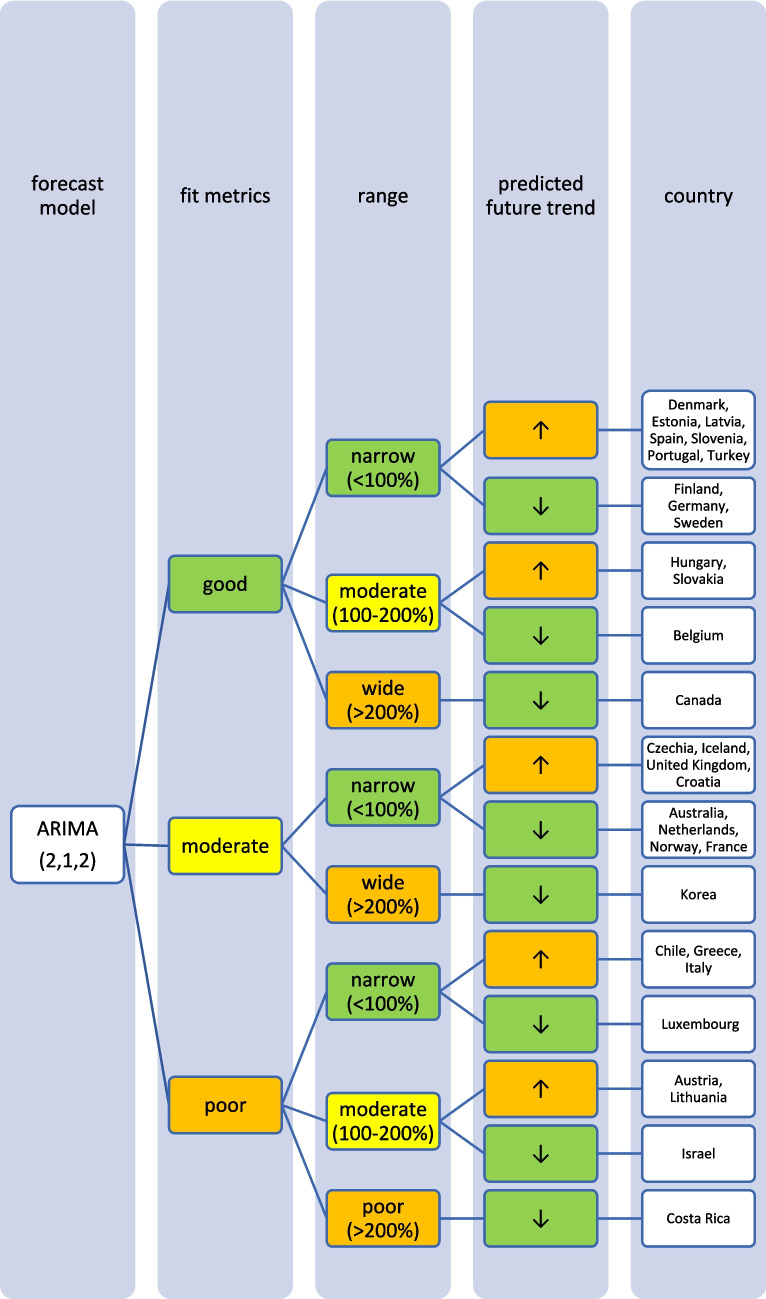
Overview of trends in OECD countries, with reliability assessed based on fit metrics and variations within the confidence interval. Green indicates strong fit metrics, narrow ranges and decreasing trends (positive outcome). Yellow represents moderate fit metrics and moderate ranges. Orange highlights poor fit metrics, wide ranges and increasing trends (areas of concern)

Summarised, predictions being suggested to be accurate include Denmark, Estonia, Latvia, Slovenia, Spain, Portugal and Turkey (Table 4, Fig. 7). These countries have both good fit metrics and a narrow range, pointing towards a very robust model and reliable prediction. Countries assessed with a right predicted trend but uncertainties in the exact consumption include Czechia, Hungary, Iceland, Slovakia, the United Kingdom and Croatia. These countries offer good or moderate fit metrics and a moderate range. Countries with an uncertain development include Austria, Chile, Greece, Italy and Lithuania. These countries have poor fit metrics and a narrow or moderate range. Because of poor data quality, a truly reliable model could not build. Poor fit metrics does not mean that the prediction is unlikely, but that the model is not able to capture the historical development quite exactly.

**Table 4:**
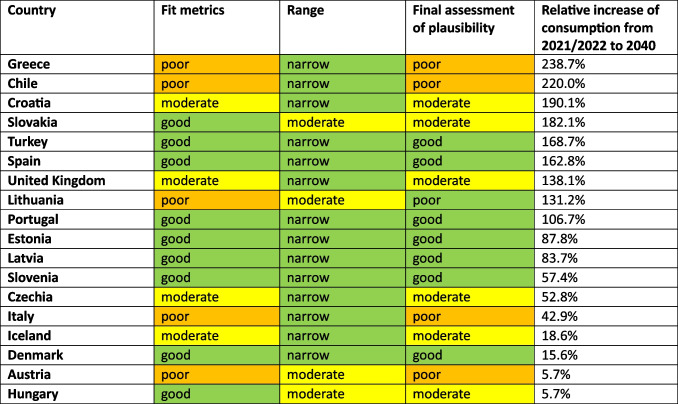
Overview about characteristics for the final assessment of accuracy for countries with a predicted increase. Countries are sorted descending by their relative change

### Countries with a decreasing trend: prediction and assessment of reliability

**Fig. 8:**
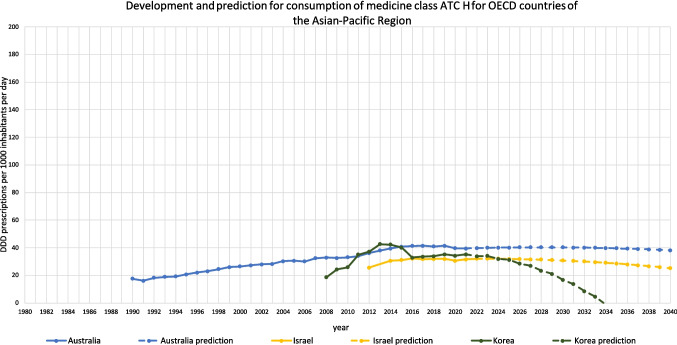
Overview of historical development and future predictions of the analysed OECD countries of the Asian-Pacific Region with the ARIMA(1,2,1) model

For 13 countries, a decreasing trend is depicted (Table 5, Figs. 2, 5, 8). This includes Australia (−3.4%, reaching 38.2 DDD prescriptions per 1000 inhabitants per day by 2040), Belgium (−38.8%, reaching 26.8 DDD), Canada (−95.1%, reaching 2.0 DDD), Costa Rica (−79.5%, reaching 5.6 DDD), Finland (−14.7%, reaching 47.3 DDD), France (−100.0%, reaching 0.0 DDD), Germany (−16.4%, reaching 73.6 DDD), Israel (−21.6%, reaching 25.2 DDD), Korea (−100.0%, reaching 0.0 DDD), Luxembourg (−100.0%, reaching 0.0 DDD), the Netherlands (−35.9%, reaching 19.0 DDD), Norway (−23.6%, reaching 38.0 DDD) and Sweden (−43.6%, reaching 24.9 DDD).

**Table 5:**
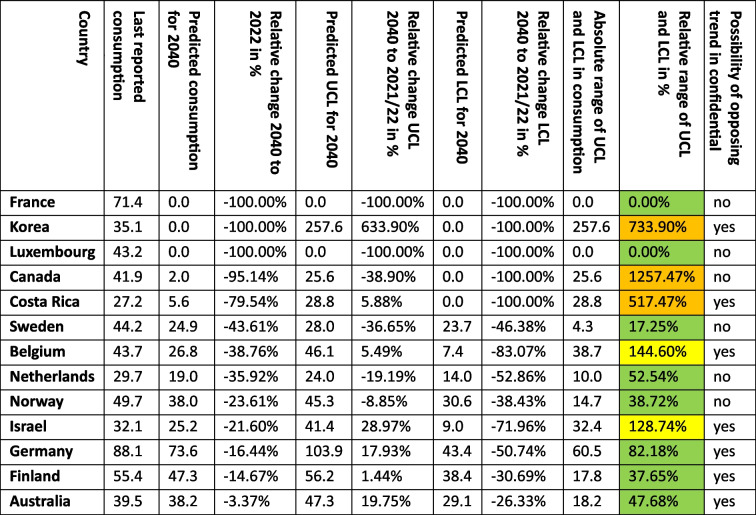
Overview about predictions for the OECD countries with a predicted decrease. Countries are sorted descending by their relative change. The range between LCL and UCL for the prediction for 2040 is divided into a good fit (green colour) with a range under 100%, a moderate fit (yellow colour) with a range between 100–200%, and a poor fit (orange colour) with a range exceeding 200%

An opposing trend, offered by the UCL and aiming towards an increasing trend, is possible for Australia (UCL of + 19.8%, prediction up to 47.3 DDD), Belgium (+ 5.5% with 46.1 DDD), Costa Rica (+ 5.9% with 28.8 DDD), Finland (+ 1.4% with 56.2 DDD), Germany (+ 17.9% with 103.9 DDD), Israel (+ 29.0% with 41.4%) and Korea (+ 633.9% with 257.6 DDD). No opposing trend is possible for Canada, France, Luxembourg, the Netherlands, Norway, Belgium and Sweden (Table 5).

The relative changes range from −3.4% for Australia to −100.0% for France, Korea and Luxembourg. While Australia has the smallest absolute decrease with −1.3 DDD, France has the highest absolute decrease with −71.4 DDD. Within the countries with a predicted decrease, France, Korea and Luxembourg have the smallest predicted consumption with 0.0, while Germany has the largest prescription volume with 73.6 DDD. As higher the predicted decrease, the more abrupt or ongoing decrease for a long time, but the more questionable is the reliance whether the forecast is correct, especially if the predicted consumption is zero. In general, a decrease is the favoured development, hinting towards a rational prescribing, especially of thyroid hormones. However, the extent to which the prediction appears realistic must be considered critically.

The reliability of each ARIMA(2,1,2) model is further evaluated by examining fit metrics (Table S4). A robust model fit is expected to have high R-squared and stationary R-squared values, paired with low MAPE and BIC scores. Conversely, poor fit models often show low R-squared metrics with higher MAPE and RMSE values, indicating the model’s limited ability to capture data dynamics accurately. A good model fit is assessed for Belgium, Canada, Finland, Germany and Sweden. Moderate fit metrics include the ARIMA models of Australia, Korea, the Netherlands and Norway. In contrast, poor fit metrics are exhibited for Costa Rica, France, Israel and Luxembourg. Analogue to the models of the countries considered as increasing, reasons for a poor model adjustment can be diverse. Especially important is the data quality and the historical development of the curve. If there is a low number of data points, strong fluctuations or distortions, the curve can’t be adjusted as well as with a good data quality. External influences, leading to strong changes, can also affect the ability to fit a good model.

The preciseness of the model can be analysed by the range between the UCL and the LCL. A narrow range is defined with a range under 100.0%, while a moderate is apparent with a range between 100.0–200.0%, and a range exceeding 200.0% being considered as large. A narrow range is depicted for Australia (47.7%), Finland (37.7%), France (0.0%), Germany (82.2%), Luxembourg (0.0%), the Netherlands (52.5%), Norway (38.7%) and Sweden (17.3%). A moderate range is exhibited for Belgium (144.6%) and Israel (128.7%). A wide range is existing for Canada (1257.5%), Costa Rica (517.5%) and Korea (733.9%). The range offers an assessment of the uncertainty of the model forecast and the variety of possible developments. Countries with a narrow range offer a quite secure forecast, while a moderate range depicts more uncertainties and the possibility for differing developments than the predicted consumption. The countries with wide ranges are predicted with a strong decrease, resulting in low DDDs, which explains the large relative range between LCL and UCL.

Countries that have shown an increasing trend over the whole period analysed may be expected to show a decreasing trend in the future. This may be due to recent trend reversals or time breaks that distort the trend. Examples are Korea, Belgium and the Netherlands, which were increasing when looking at the first to the last data, but are predicted to show moderate to strong decreases in the coming years, with good to moderate fit metrics. Looking at past trends, in all these countries there was a trend towards reversal in the last years, with consumption reaching a plateau or even decreasing. The forecast model recognises and continues this trend.

Summarised, predictions being suggested to be surely accurate include Finland, Germany and Sweden (Table 6, Fig. 7). These countries have both good fit metrics and a narrow range, pointing towards a very robust model and reliable prediction. The predicted decreases seem plausible and realistic. Countries assessed with a right predicted trend but uncertainties in the exact consumption include Australia, Belgium, Canada, the Netherlands and Norway. These countries offer good or moderate fit metrics and a moderate range. In most cases, predictions seem realistic but offer a greater variety than the countries considered as accurate. An exception is Canada, the relative decrease of −95.1% seem unrealistic, but the offered range is plausible. Countries with an uncertain development include Costa Rica, France, Israel, Korea and Luxembourg. These countries have poor fit metrics and a narrow or moderate range. Because of poor data quality, a truly reliable model could not build. In these cases, the predicted consumption seems unrealistic with decreases up to −100.0%. Nevertheless, a continuing decreasing trend is plausible, since the countries have experienced a sharp decrease in recent years.

**Table 6:**
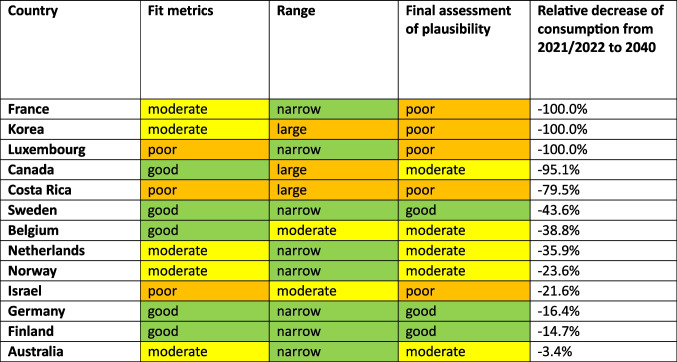
Overview about characteristics for the final assessment of accuracy for countries with a predicted decrease. Countries are sorted descending by their relative change

### Present and predicted patterns in thyroid hormone consumption and the influence of cultural practices

**Fig. 9:**
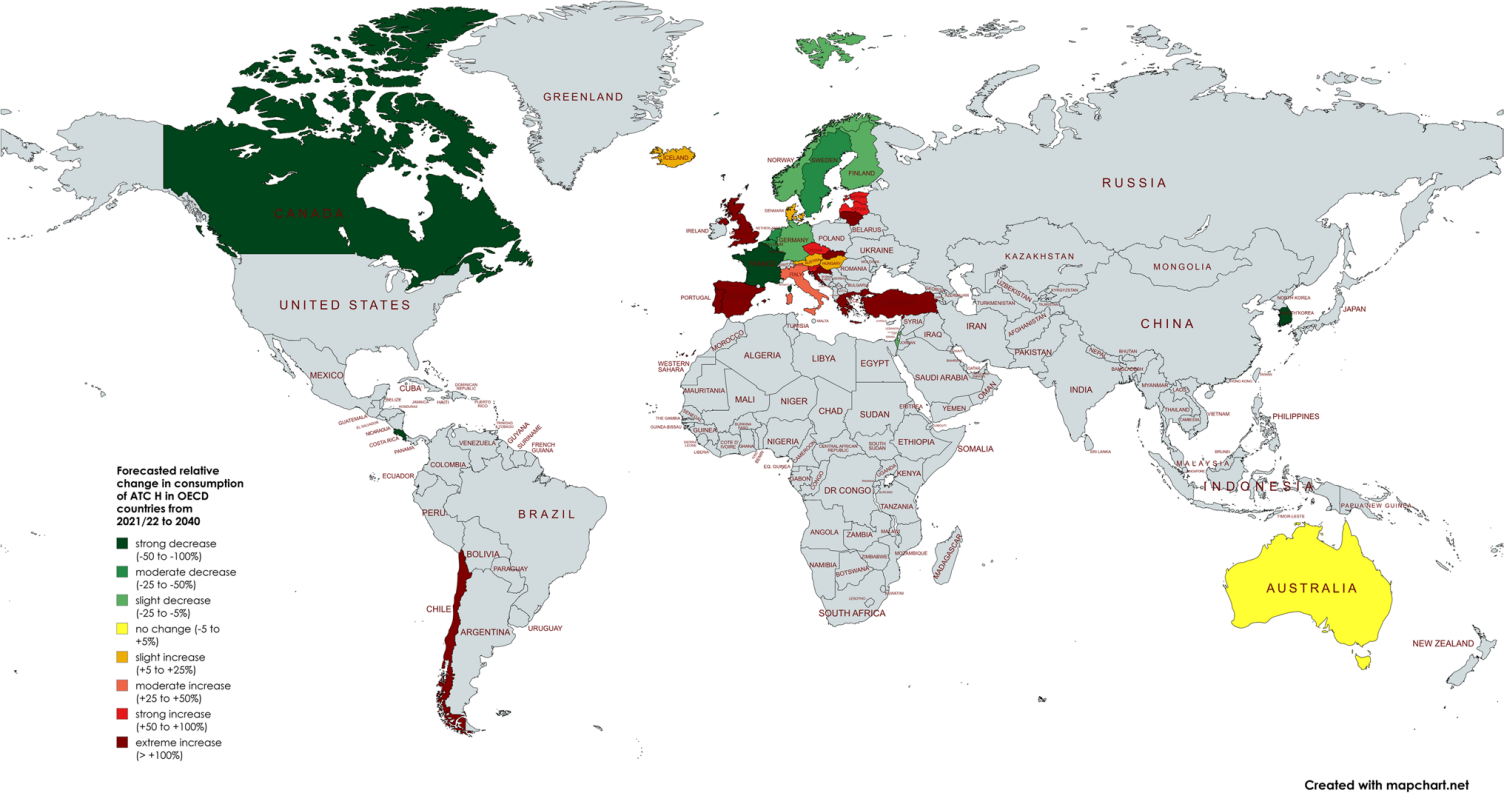
The world map presents an overview of forecasted relative changes in the consumption of ATC H in OECD countries from 2021/22 to 2040. Countries are color-coded to indicate trends. Dark green represents strong decreases (positive outcomes), light green shows moderate decreases, yellow represents slight to moderate increases, orange and red highlight regions with strong or very strong increases (areas of concern). Gray indicates regions that were not analysed. The world map was created with mapchart.net (https://www.mapchart.net/index.html)

**Fig. 10:**
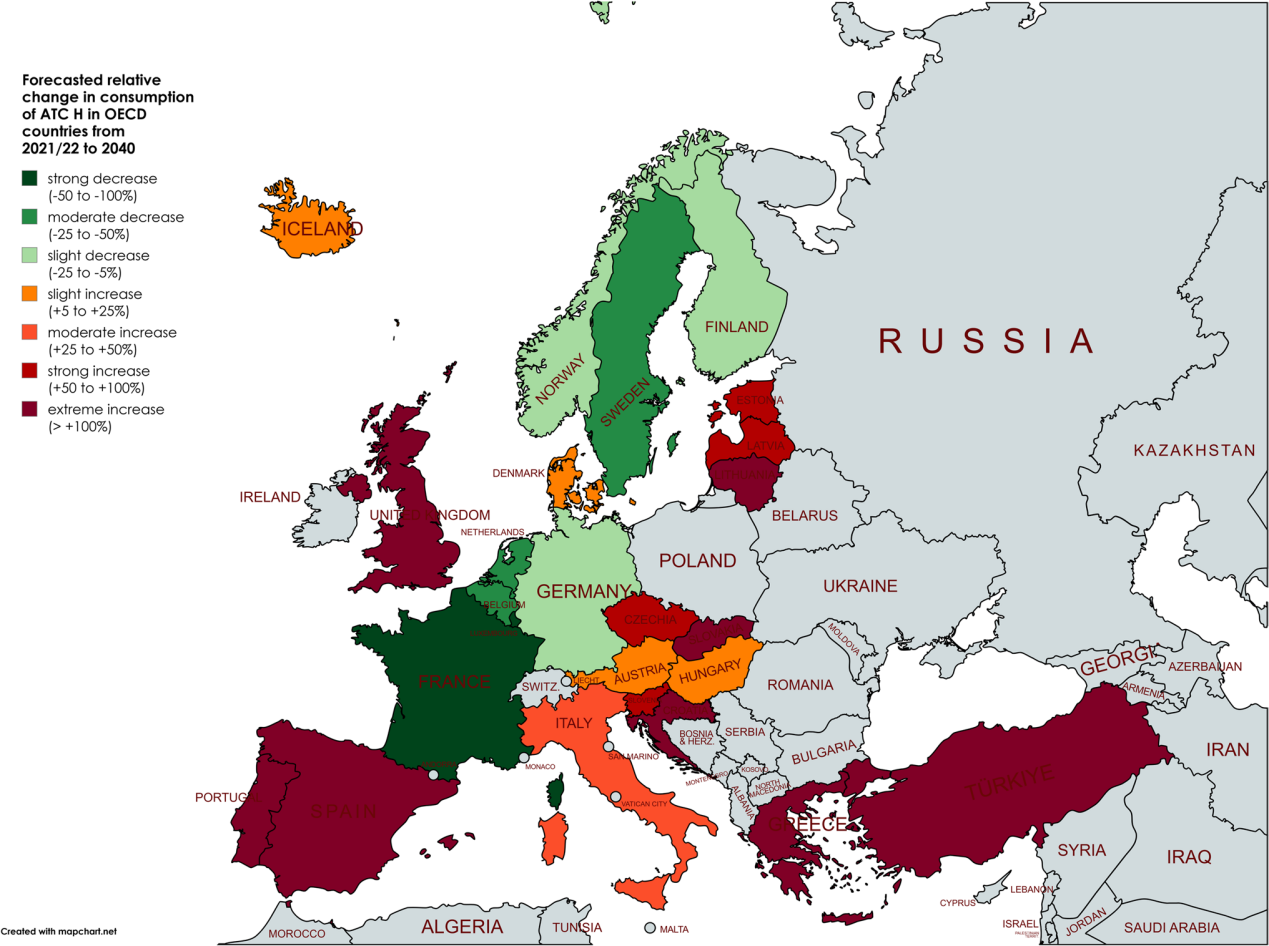
The map of Europe presents an overview of forecasted relative changes in the consumption of ATC H in European OECD countries from 2021/22 to 2040. Countries are color-coded to indicate trends. Dark green represents strong decreases (positive outcomes), light green shows moderate decreases, yellow represents slight to moderate increases, orange and red highlight regions with strong or very strong increases (areas of concern). Gray indicates regions that were not analysed. The world map was created with mapchart.net (https://www.mapchart.net/index.html)

The strongly differing consumption levels in thyroid hormone use across OECD countries suggest that irrational factors, rather than clinical need, play a dominant role (Figs. 9 and 10). It is unlikely that variations in the incidence or prevalence of thyroid disorders, especially in geographically close countries, can fully explain these significant differences and trends. For example, the strong contrast between declining consumption in countries like France and Luxembourg in recent years and in the future versus strong increases in Greece and Spain underscores the influence of cultural norms, health care practices and prescribing habits rather than simply increasing incidence. To address this issue, countries with outstanding declines and increases are analysed, followed by an assessment of patterns at regional levels.

France, Korea, Luxembourg and Canada are expected to experience the most significant reductions in hormone use, with projected declines of up to −100.0% for the first three and −95.1% for Canada. These forecasts suggest a move toward addressing overtreatment and optimizing medical practices. In terms of current consumption, Korea, Canada and Luxembourg are at an average level, while France is comparatively high. For all four countries, this level changes towards the lowest consumption level in the OECD until 2040. However, the reliability of these predictions varies. For instance, France and Luxembourg have poor model fit metrics, which casts some doubt on the accuracy of their forecasts. Meanwhile, Korea and Canada present stronger evidence, with Korea showing moderate model fit and Canada demonstrating high reliability, although confidence intervals remain wide for all four nations. These reductions align with efforts in these countries to adopt stricter guidelines for diagnosing and treating conditions like subclinical hypothyroidism. In France, health authorities recommend against unnecessary hormone substitution, particularly for older patients (HAS 2023). Similarly, Korea advises against treatment for mild subclinical hypothyroidism in elderly populations (Ku et al. 2023), and Canada promotes evidence-based initiatives to reduce unnecessary testing and prescribing (UBC 2024; Birtwhistle et al. 2019). These measures align with observed trends, even as rising prevalence rates for thyroid-related conditions continue to present challenges (Jonklaas and DeSale 2020; Taylor et al. 2018).

Conversely, many countries are projected to see significant increases in hormone consumption. Particularly strong increases within predictions include Greece (+ 238.1%), Chile (+ 220.0%), Croatia (+ 190.1%), Slovakia (+ 182.1%), Turkey (+ 168.7%), Spain (+ 162.8%), the United Kingdom (+ 138.5%), Lithuania (+ 131.2%), Portugal (+ 106.7%), Estonia (+ 87.8%), Latvia (+ 83.7%), Slovenia (+ 57.4%) and Czechia (+ 52.8%). These increases, ranging from moderate to very high, suggest that by 2040, many of these countries will have alarmingly high levels of hormone use, driven by a mix of rising prevalence, expanding screening programs and problematic prescribing practices. Looking at recent consumption levels, the group of rising countries is heterogeneous, including countries with below-average consumption levels such as Greece, Slovenia, Turkey, Estonia and Slovakia, moderate consumption levels for Lithuania and Latvia, and above-average levels for Croatia, Portugal, the United Kingdom, Spain, Czechia and Chile. In 2040, these countries are expected to have high levels of consumption above average.

In countries such as Chile, Spain and Croatia, a combination of moderate to high baseline consumption levels and widespread overprescription significantly contributes to these trends (Cassemiro et al. 2023; Sawka 2019; Díez and Iglesias 2023). Evidence from Slovakia indicates that up to 84% of levothyroxine prescriptions lack proper medical justification, often targeting euthyroid patients or those with mild thyroid conditions (Negro et al. 2024). Similarly, Chile has been found to issue roughly 50% of doctors with inappropriate prescriptions (Cassemiro et al. 2023), with Greece and Spain also demonstrating concerning levels of overprescription (Negro et al. 2024; Galofré et al. 2021). In Greece and Spain, cultural expectations for immediate pharmacological interventions (Borg and Camilleri 2019) often lead to overprescription, including the use of thyroid hormone therapy in euthyroid patients, despite international guidelines advising against it. These findings suggest that many of these increases are not solely a result of rising need but are also heavily influenced by non-rational prescribing behaviours and overtreatment.

Regional patterns offer further insight into these trends. Declines in hormone consumption are more common in North and Western Europe as well as parts of the Asia–Pacific region (Figs. 3, 5, 8, 9, 10), where stricter prescribing guidelines and improved diagnostic practices help prevent overuse. In contrast, increases are concentrated in Southern and Eastern Europe as well as Latin America (Figs. 2, 4, 5, 9, 10). This may represent a discrepancy with recent consumption levels, where Western Europe (mean 53.4 DID), America (mean 45.7 DID) and Northern Europe (mean 42.0 DID) have high to moderate levels of consumption, whereas the regions of Southern Europe (mean 38.8 DID), Eastern Europe (mean 36.2 DID) and the Asia–Pacific region (mean 35.6 DID) are considered to have low levels of consumption. These high levels of consumption in Western and Central Europe towards lower levels in Southeastern Europe recently are projected to reverse. In 2040, the highest consumption levels are predicted for Southern Europe (mean of 91.8 DID), America (mean of 75.2 DID) and Eastern Europe (mean of 63.1 DID), while moderate consumption is predicted for Northern Europe (mean of 46.4 DID) as well as low levels for Western Europe (mean of 37.4 DID) and the Asia–Pacific Region (mean of 21.1 DID) (Table 7). Although comparisons of the means are limited by differences in data methodology (Table S1), model reliability, forecast plausibility and different consumption between countries within a region, they provide a good overview of regional patterns.

**Table 7:**
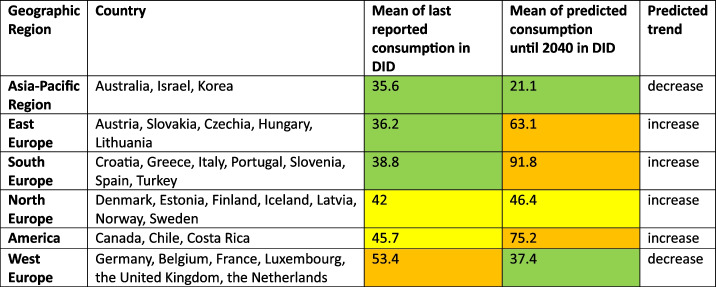
Characterisation of the analysed OECD countries in geographical regions, the mean of the consumption in the latest reported data as well as for the forecast in 2040 and a characterisation of the predicted trend of increase or decrease. Regions are sorted ascending by their last reported consumption. Consumption levels are coloured green for low consumption levels below 40 DID, yellow for moderate consumption levels between 40–50 DID and orange for high consumption levels above 50 DID

Present and future regional differences are partly explained by varying prevalence rates of thyroid disorders, influenced by factors such as iodine deficiency and demographic changes. Areas with persistent iodine deficiency, including parts of South America, South-East Asia and Europe, tend to have higher rates of thyroid-related diseases (Seo and Chung 2015; Vanderpump 2011; Taylor et al. 2018; The Health Policy Partnership 2024). Additionally, aging populations in many countries increase the demand for treatment, as older individuals are more likely to require hormone replacement therapy (Mitchell et al. 2009). Overall, prevalence is increasing in many countries (Strikić Đula et al. 2022; Langer et al. 2003; Atmis et al. 2021; Ayvaz et al. 2022; Mitchell et al. 2009). However, women and Caucasian populations tend to be more affected by thyroid disease than men or African populations (Vanderpump 2011; Mendes et al. 2019). Furthermore, thyroid diseases appear to be adequately detected and treated sufficiently in most regions, for example with only small differences in undetected overt hypothyroidism in Europe (Mendes et al. 2019). Notably, variabilities can arise depending on the study considered (Mendes et al. 2019). The similar prevalence rates and treatment sufficiency in these countries cannot explain the discrepancy of different consumption trends.

Advanced health care systems in Western and Northern Europe (European Commission 2017; OECD 2024c), with comprehensive screening and diagnostic protocols, may have led to higher detection rates of thyroid disorders and increased thyroid hormone prescriptions (Thiyagarajan et al. 2024). In contrast, ongoing improvements in healthcare systems in southern and eastern Europe (Frank et al. 2014; OECD 2024c) are expanding diagnostic capabilities, which increases the potential for higher thyroid hormone prescribing in these regions. Furthermore, in regions where health care systems are already well established and thyroid hormone consumption has historically been high, concerns about potential misuse have led to regulatory interventions and surveillance that have successfully resulted in plateauing or even declining prescription trends in many countries.

Beyond these external factors, prescribing behaviour and cultural factors play a decisive role in shaping trends, with greater risk of unchecked increases in consumption where evidence-based guidelines are poorly followed. Countries with robust healthcare policies and evidence-based guidelines, such as France and Canada, are better positioned to manage thyroid hormone use effectively and avoid unnecessary treatments. Conversely, in regions like Eastern and Southern Europe as well as Latin America, non-evidence-based practices lead to widespread overprescription of levothyroxine. Nearly 48.5% of physicians in Eastern Europe prescribe hormones without evidence-based justification, with similar rates of 39.1% reported in Southern Europe and Western Asia (Ayvaz et al. 2022; Negro et al. 2024). Established healthcare routines further influence these trends (Fagan 2023), as observed in other medicine classes like antibacterial drugs (Bindel and Seifert 2024, Borg and Camilleri 2019). In countries with a long-standing culture of medical conservatism, like France, stricter adherence to guidelines curbs unnecessary prescriptions. On the other hand, in more interventionist cultures such as Greece, Spain and Croatia, patients demand for immediate treatment (Borg and Camilleri 2019) often override clinical guidelines (Negro et al. 2024). In Southern and Eastern Europe, as well as Latin America, patients often expect pharmacological solutions for minor health concerns. These differences suggest that cultural expectations deeply shape prescribing behaviour. A comparison of pharmacotherapeutic practices is provided in Table 8.

**Table 8:**
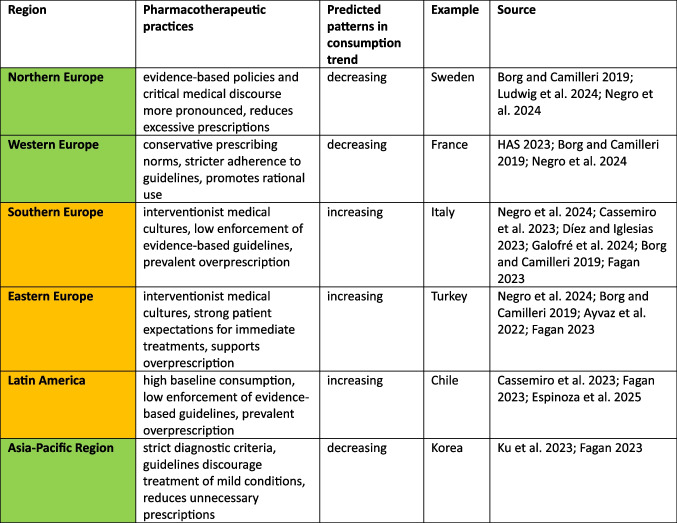
Overview about characteristics for pharmacotherapeutic practices by geographical region. Regions with a predominantly increasing trend are highlighted in orange, while those with a decreasing trend are highlighted in green. A representative country is provided as an example for each region

**Fig. 11:**
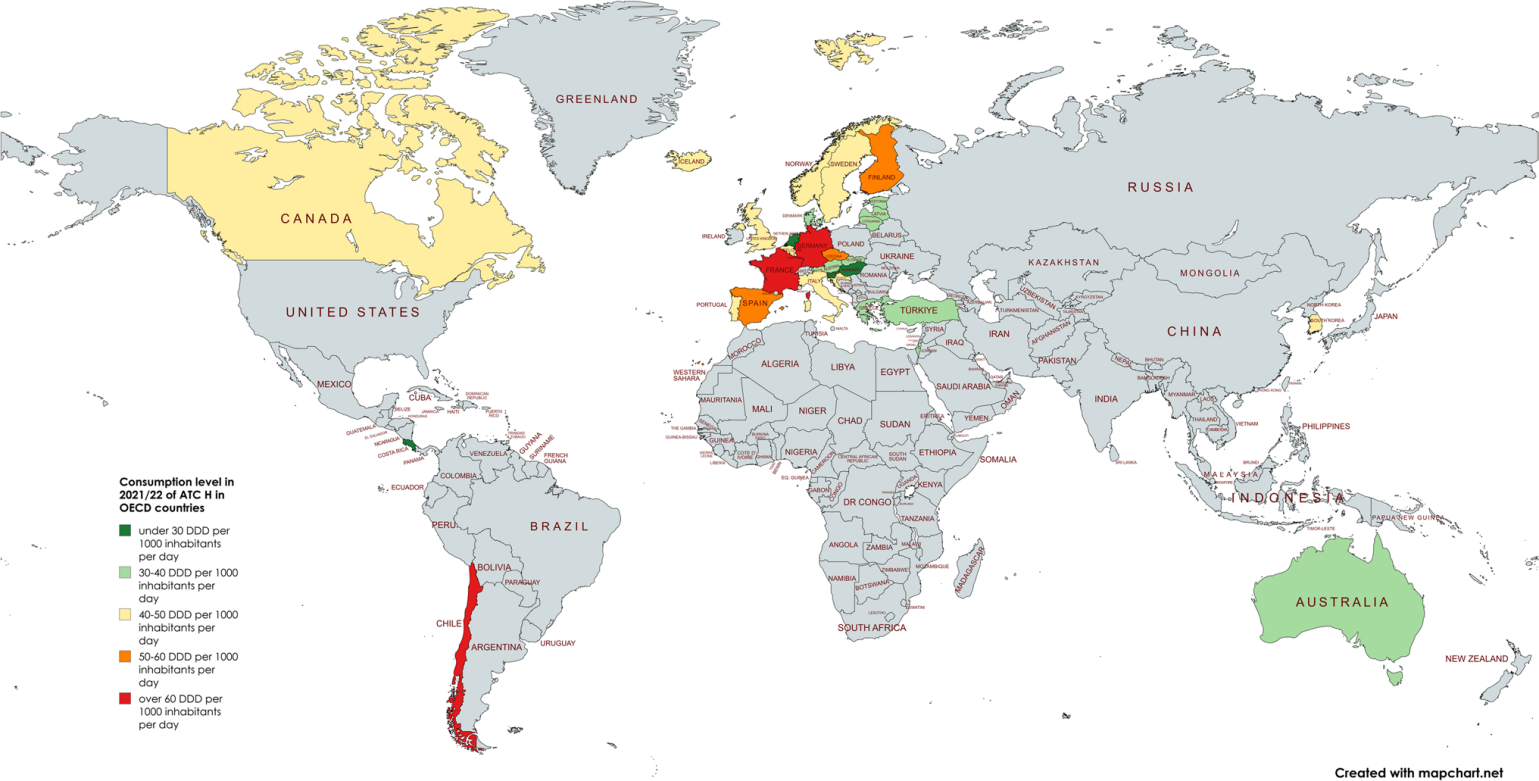
Global overview of the consumption level of ATC H in OECD countries in 2021/22. Quantities are expressed in DDD per 1000 inhabitants per day. Countries are color-coded to indicate trends. Dark green represents a level under 30 DDD, light green exhibits a level between 30 and 40 DDD, yellow represents 40 to 50 DDD, orange marks 50 to 60 DDD and red highlight regions with a level exceeding 60 DDD. Gray indicates regions that were not analysed. It is important to note that the consumption levels are not directly comparable across countries due to variations in data collection methodologies. The world map was created with mapchart.net (https://www.mapchart.net/index.html)

However, when considering consumption levels in 2021/22, Central Europe shows a rather high level of prescribing, while Eastern Europe, for example, has more moderate levels (Fig. 11). This may suggest a discrepancy with the hypothesis of a forecasted increase in consumption for several regions, including Eastern Europe.

Summarized, there a both positive and problematic trends in recent years and in the forecast for the analysed OECD countries. Some countries are in the process of counteracting overtreatment and are already experiencing a starting decline, while others remain at high risk of uncritical usage, with limited measures to address this development. Non-evidence based used is widespread in countries of east and south Europe as well as in Latin America, which is also reflected within the trend of future consumption. Within Europe, a reversal in consumption levels is predicted. While Western and Central Europe have recently had above-average consumption levels, Southeastern Europe has lower consumption levels. In 2040, the south-east is predicted to increase its level, while the northern countries are predicted to decrease. These observations emphasize the significant role cultural practices play in shaping hormone consumption trends, alongside systemic and regulatory factors. Addressing these discrepancies requires a broader recognition of cultural influences in pharmacology and targeted efforts to align prescribing norms with evidence-based practices worldwide.

## Limitations

The analysis presented in this study relies on data extracted from the OECD data explorer (OECD 2024a), which aggregates information provided by national authorities of the respective countries. Variations in methodologies, data collection procedures and the inclusion of different medical prescription fields have occurred across several countries (Table S1). These variations have led to time series disruptions and potential distortions in the data. Furthermore, the comparability of medicine consumption data between countries is limited due to differences in definitions, data collection approaches and the scope of included medical or geographical fields. As a result, only part of the consumption could be reported, which could be misleading when comparing consumption levels between countries and regions. The OECD provides a comprehensive list detailing the specific methodologies and reasons for these discrepancies (OECD 2024b).

This study focused on examining the overall trends within the ATC H medicine class, particularly emphasizing thyroid hormones due to their significant representation within the category of systemic hormones. Consequently, the analysis primarily addressed the general development of the medicine class and thyroid hormones, excluding smaller subcategories of medical preparations from consideration. This focus aimed to capture broader historical developments and trends rather than specific, isolated events.

The feasibility of forecast modelling was contingent on the quality of data available for each country. In some instances, limited data quality resulted in inadequate model adjustments, as reflected in poor fit metrics or in the inability to construct a viable model, as happened for New Zealand.

While ARIMA models are widely recognized for their effectiveness in time series forecasting (Hyndman and Athanasopoulos 2021; Bindel and Seifert 2025), they also come with inherent limitations. One significant drawback is the assumption of linear relationships between time points, which may not adequately capture non-linear patterns within the data (Bindel and Seifert 2025). Additionally, the models do not account for external factors that might influence outcomes but were not included in the analysis. The extent of such influences remains uncertain. Predictions are inherently limited to the scope of current knowledge. Therefore, unanticipated future developments cannot be accurately forecasted (Bindel and Seifert 2025). While the prediction of a shorter period of time can be considered as reliable within countries considered to have an accurate model, there are some concerns about the ability to make accurate long-term projections to 2040. Therefore, long-term projections should be understood as focusing on the trend rather than the actual values.

This study adhered to specific criteria for statistical methodologies and the evaluation of model fit (detailed in the "Materials and Methods" chapter). A single ARIMA model was selected as the most suitable approach based on these predefined criteria. However, modifications in the ARIMA model, alternative statistical techniques or changes in the criteria for evaluating fit metrics could lead to different conclusions.

## Conclusions

The forecast of future consumption of systemic hormones was possible for 30 of 31 OECD countries with available data as well as Croatia (non-OECD). Due to insufficient data, no model could be built for New Zealand. Both increasing and decreasing trends were observed, with variation in reliability and preciseness. Overall, the forecasts appear plausible to a certain extent, with the ARIMA(1,2,1) model emerging as the most suitable for the time series analysis conducted.

Countries with a consideration of a very reliable forecast have models with good fit metrics (Tables 4 and 6). The narrower the range of the confidential interval, the more precise the estimation. A very precise and reliable forecast exists for Denmark (increasing), Estonia (increasing), Finland (decreasing), Germany (decreasing), Latvia (increasing), Spain (increasing), Sweden (decreasing), Portugal (increasing) and Turkey (increasing). A moderate range is depicted for Belgium (decreasing) and Hungary (increasing).

Countries having a correctly predicted trend direction but with potential discrepancies in the absolute values, the fit metrics were moderate, suggesting that while the models were reasonably well-calibrated, there remains some degree of uncertainty. Within this category, narrower prediction ranges were observed for Australia (decreasing), Czechia (increasing), Iceland (increasing), the Netherlands (decreasing), Norway (decreasing), Slovenia (increasing), the United Kingdom (increasing) and Croatia (increasing). A moderate range was observed for Slovakia (increasing). However, the case of Korea (decreasing), with its moderate fit metrics coupled with particularly wide prediction intervals, suggests a high degree of uncertainty regarding future trends.

Several countries exhibited poor fit metrics, which rendered their forecasts unreliable, as the models could not be adequately aligned with historical trends. This is evident for Austria (increasing), Chile (increasing), Costa Rica (decreasing), France (decreasing), Greece (increasing), Israel, Italy (increasing), Lithuania (increasing) and Luxembourg (decreasing).

The reliability of these forecasts is significantly influenced by the quality of the data and the shape of the observed time series. When there are numerous data points available and the trend shows a steady, consistent development, the fit metrics are generally stronger. Conversely, when data is sparse, disrupted by changes in methodology or characterized by significant fluctuations, sharp spikes or abrupt trend changes, the fit metrics tend to deteriorate. Since “the ARIMA model’s reliability is predicated on the assumption that conditions remain stable, making it less effective when forecasting within unpredictable or volatile conditions” (Bindel and Seifert 2025). Conclusive, long-term predictions can be used to estimate a trend rather than predicting accurate consumption volumes, while in a short-term the forecast can be seen as reliable in those instances where the model was considered accurate.

Thyroid hormones represent the largest share of prescriptions within the analysed ATC group H. Literature suggests a prototypical pattern of consumption, with an initial rise followed by a plateau or gradual decline (Jonklaas and DeSale 2020). The timing, extent and intensity of this trend reversal depend on several factors, including extensive diagnostics for thyroid disorders, the availability of high-quality healthcare and low thresholds for laboratory values indicating treatment. Conversely, updated guidelines, community discussions and health initiatives promoting awareness of rational use often leads to a stabilization or reduction in consumption (Jonklaas and DeSale 2020; Thiyagarajan et al. 2024; Turunen et al. 2021; Ludwig et al. 2024).

Consumption and trends vary considerably by geographical region (Figs. 9 and 10). Consumption is forecast to increase in South-Eastern Europe and Latin America, while it is expected to decrease in Western Europe and the Asia–Pacific Region. The regions predicted to decline have recently reported relatively high levels of consumption, whereas the rising countries have more heterogeneous levels. External factors such as the increasing prevalence of thyroid disorders (Kalere et al. 2019; Mitchell et al. 2009; Atmis et al. 2021; Langer et al. 2003) or the risk of iodine deficiency (Vanderpump 2011; Taylor et al. 2018) cannot explain past and future trends, as these factors are also present in countries with declining consumption. Instead, widespread nonrational prescribing appears to be the main driver of the predicted high consumption levels in increasing countries (Negro et al. 2024; Cassemiro et al. 2023; Ayvaz et al. 2022). In Europe, there appears to be a reversal of the current north–south shift, with consumption levels decreasing from high to moderate in northern Europe and increasing to moderate and high in southeastern Europe. Latin America continues its problematic trend with high consumption levels, while the Asian-Pacific Region is considered with a further decline. These patterns strongly suggest that cultural differences and prescribing behaviour, rather than the prevalence of thyroid disorders alone, are largely responsible for the observed trends. As this trend is also observed in other classes of medicines (Bindel and Seifert 2024), there is strong evidence of a structural problem.

The ability to plan the required quantities in advance has now been created. From a policy perspective, a stable or declining trend in hormone prescriptions is generally preferable to an upward trend, as an increasing volume of prescriptions can indicate potential overuse (Thiyagarajan et al. 2024; Schröder and Seifert 2024). Reducing unnecessary medication use is crucial not only for protecting patients from undue pharmaceutical exposure but also for alleviating the financial burden on healthcare systems. Notably, countries that have already implemented measures to curb overuse, such as France, Korea, and Canada (HAS 2023; UBC 2024; Birtwhistle et al. 2019), show a significant, continuing decline in prescriptions. These examples demonstrate the effectiveness of educational campaigns and policy interventions in achieving desirable reductions in prescription rates. It isreasonable to assume that similar outcomes could be achieved in other countries. Therefore, it is both necessary and appropriate to pursue further reductions in systemic hormone prescriptions across all regions.

## Further perspectives

Further research is needed to validate our findings and to expand the analysis to include more countries and parameters. As new data becomes available, continuous refinement and recalibration of forecast models will be necessary to improve their accuracy.

Expanding the analysis to include other medicine classes is imperative for a deeper understanding of prescribing patterns with the aim to optimize medicine use and to ensure sustainable availability on a global scale. This broader approach aims to improve medicine utilization and to ensure the sustainable availability of essential medications worldwide.

## Take home messages for improving prescribing practices

1. **Treat the patient, not just the lab results!** Focusing solely on laboratory values such as TSH levels is not always appropriate, as mild subclinical hypothyroidism without symptoms often does not require treatment (Calissendorff and Falhammar 2020).
2. **Prevent overtreatment!** Be mindful of age-adjusted reference ranges, particularly in older adults, as TSH levels naturally increase with age. Exercise caution when making treatment decisions, as both hypothyroidism and hyperthyroidism can have adverse effects (Effraimidis et al. 2021). In general, aim to minimize the number of different medications prescribed per patient.
3. **Limit unnecessary thyroid function testing!** Excessive screening in the absence of symptoms or risk factors significantly increases the risk of over-prescribing thyroid hormones (Thiyagarajan et al. 2024). Prioritize testing for high-risk groups instead.
4. **Stay informed and up to date!** Regularly review the latest guidelines and adhere to their evidence-based recommendations to ensure best practices in prescribing.

## Supplementary Information

Below is the link to the electronic supplementary material.

Supplementary file1 (DOCX 52417 KB)

## Data Availability

All source data for this study are available upon reasonable request from the authors.

## Abbreviations

ACF: Autocorrelation function
ARIMA: Auto Regressive Integrated Moving Average model (mathematical model for time series analysis)
AVR: Arzneiverordnungsreport (Medicine Prescription Report regarding the GKV system of Germany)
BIC: Bayesian information criterion
DDD: Defined Daily Dose
DID: Defined Daily Dose per 1000 inhabitants per day
LCL: Lower confidence limit
MAE: Mean absolute error
MAPE: Mean absolute percentage error
MaxAE: Maximum absolute error
MaxAPE: Maximum absolute percentage error
OECD: Organisation for economic cooperation and development
PACF: Partial autocorrelation function
RMSE: Root mean squared error
SPSS: Statistical Product and Service Solutions (software for statistical analyses, IBM)
UCL: Upper confidence limit
