# Supplementary material for: Long-term forecasting and evaluation of medicine consumption for the ATC class H with a focus on thyroid hormones in OECD countries using ARIMA models
Source: Naunyn Schmiedebergs Arch Pharmacol. 2025 Mar 3;398(8):10691–717. doi: 10.1007/s00210-025-03930-5 (PMC12350577; doi:10.1007/s00210-025-03930-5)
Supplement: Supplementary file 1 — Supplementary file1 (DOCX 52417 KB) [file 210_2025_3930_MOESM1_ESM.docx]

**Long-term forecasting and evaluation of medicine consumption for the ATC class H with a focus on thyroid hormones in OECD countries using ARIMA models**

**Lilly Josephine Bindel and Roland Seifert**

Supplemental Methods and Materials

Modelling the best-fitting ARIMA model requires balancing complexity and precision. While it is essential to remove trends from the data to achieve stationarity, overfitting poses a significant risk by eliminating inherent structures within the dataset. Sequence, ACF and PACF plots of all degrees of differentiation can be found in the supplemental figures (Fig. S32-S124).

The initial step involves ensuring that the time series for each country is stationary. To achieve this, autocorrelation analysis was performed in SPSS, producing autocorrelation function (ACF) and partial autocorrelation function (PACF) plots. In the original series, the ACF plots for all countries indicated non-stationarity (Fig. S32-S62). This was evident through a slow decline in significant lags, reflecting a persistent trend. Examples of this behaviour include Czechia (Fig. S38) and Belgium (Fig. S34). The sequence plots of the original time series further confirmed these trends (Fig. S1-S31), underscoring the need for differentiation.

After the first differentiation, interpreting the ACF plots proved challenging (Fig. S63-S93), likely due to the complex structure of the original data, which included time breaks and missing data points (Table S1). In some cases, such as Belgium (Fig. S65) and Canada (Fig. S66), the ACF plots hinted at residual trends. To verify these observations, sequence plots of the first-degree differentiation were examined (Fig. S1-S32). These plots revealed a remaining trend for several countries, for example Canada (Fig. S4), Estonia (Fig. S9) and Germany (Fig. S12). Notably, the mean in all sequence plots deviated above or below zero, indicating residual non-stationarity. Testing ARIMA models with the structure ARIMA(p,1,q) lead to implausible forecasts and poor fit metrics, further supporting the need for additional differentiation.

A second differentiation was performed, addressing these issues. Although the ACF plots remained difficult to interpret, the sequence plots showed a significant improvement (Fig. S1-S31 and S94-S124). The data now exhibited balanced fluctuations around a mean of zero, indicating the removal of residual trends. This transformation towards reaching stationarity is well to observe in countries like Portugal (Fig. S24), Germany (Fig. S12) and Czechia (Fig. S7). This confirms the need of a second differentiation, now confirming that the data could be considered stationary and ready for parameter adjustment. Referring to the ACF and PACF plots (Fig. S94-S124), lag one was often significant in both, guiding parameter selection. Testing these models and evaluating fit metrics supported the choice of these parameters (ARIMA(1,2,1)) as optimal.

However, differences in behaviour between countries were observed. For instance, some countries like Finland (Fig. S10) displayed clear sequence plots with smooth trends that were straightforward to interpret. In contrast, others such as Israel (Fig. S16) exhibited strong fluctuations, complicating interpretation. Structural changes in the data such as time breaks, missing values or a low number of observations likely contributed to these challenges. These differences were also reflected in fit metrics. Countries like Belgium and Canada demonstrated well-fitting metrics, whereas Israel and Italy showed poor fit and interpretability (Table S3 and S4).

In summary, after evaluating various parameter configurations, a generalized ARIMA(1,2,1) model was selected. This model allows for cross-country comparisons and the identification of overarching trends, which is essential for analysing systemic hormone trends. However, the generalization may limit the precision of forecasts for certain countries, emphasizing the importance of focusing on trends rather than exact values in the analysis.

Supplemental Tables

***Table S1:*** *Summary of reported time breaks and differing definitions (OECD 2024b) for OECD countries with available data. Furthermore, the available years are mentioned.*

| **Country** | **Available Years** | **Marked as Definition Differs** | **Main Reasons for Differing Definition** | **Reported Time Series Break** | **Reason for Distortion** |
| --- | --- | --- | --- | --- | --- |
| Australia | 1990-2021 | Yes | Exclusion of combination products; only outpatient data | 2013, level shift | Exclusion of private prescriptions |
| Austria | 2010-2021 | Yes | Exclusion of combination products before 2019; only outpatient data | No |  |
| Belgium | 1997-2021 | Yes | Only outpatient data | 2008, level shift | Inclusion of self-employed persons; increase around 5.4% |
| Canada | 2007-2022 | Yes | Only three provinces (British Columbia, Manitoba, Saskatchewan); limited medicine programs included | 2016, level shift | Change of medicine information system; inclusion of public, private, OTC claims |
| Chile | 2011-2022 | No |  | No |  |
| Costa Rica | 2001-2022 | No |  | 2007, level shift | Change in reporting institution |
| Czechia | 1980-2021 | No |  | No |  |
| Denmark | 1997-2021 | No |  | No |  |
| Estonia | 2000-2022 | No |  | 2006, level shift | Change in ATC Index classification |
| Finland | 1998-2021 | No |  | No |  |
| France | 2010-2021 | No |  | No |  |
| Germany | 1986-2021 | Yes, then stabilizing | Only statutory health insurance; only outpatient data | 1991, 2004, 2018 level shifts | Reunification; GMG law; partial hospital consumption inclusion |
| Greece | 1998-2004; 2013-2015; 2016-2022 | Yes | Only outpatient data; exclusion of growth factors | 2013, level shift | Change of source & methodology; hospital consumption inclusion |
| Hungary | 2003-2022 | Yes | Only includes subsidized preparations in pharmacies | 2007, level shift | Exclusion of hospital consumption |
| Iceland | 1989-2022 | Yes | Method changes; data pre-2007 not updated with ATC version | 2011, level shift | Change of methodology; exclusion of hospital consumption |
| Israel | 2012; 2014-2022 | Yes | Not all regions included; only outpatient sector | No |  |
| Italy | 2011-2022 | No |  | No |  |
| Korea | 2008-2021 | No | Changes in ATC classification without updating outdated values | 2011, 2016 level shifts | Change in methodology |
| Latvia | 2012-2022 | No |  | No |  |
| Lithuania | 2010-2021 | No |  | No |  |
| Luxembourg | 2005-2022 | Yes | Inclusion of insured resident population; changes in ATC classification | No |  |
| Netherlands | 2001-2021 | Yes | Only outpatient sector; pre/post-2008 data non-comparable (change in DDD) | No |  |
| New Zealand | Estimated values 2018-2022 | Yes | Exclusion of combination products | No |  |
| Norway | 1999-2022 | No |  | No |  |
| Portugal | 2000-2022 | Yes | Only outpatient sector included | No | Change in data source/reporting system |
| Slovakia | 1996-2021 | No |  | 2016, level shift |  |
| Slovenia | 2006-2022 | Yes | Only outpatient sector included | No |  |
| Spain | 2004-2022 | Yes | Only outpatient sector until 2018; ATC classification changes | 2014, level shift | Inclusion of special health insurance schemes |
| Sweden | 2000-2022 | No |  | No |  |
| Turkey | 2007-2021 | Yes | Only includes data dispensed in pharmacies | No |  |
| United Kingdom | 2005-2021 (estimated values 2004-2005; 2018-2021) | Yes | Only outpatient sector; only statutory health insurance | 2013, level shift | Data for England till 2012; UK-wide data from 2013 |
| Croatia (non-OECD) | 2005-2021 | No |  | No |  |

***Table S2:*** *Summary of fit metric classifications for the assessment of model accuracy. The classification is divided into three categories: good, moderate and poor fit.*

| **Fit Metric** | **Good** | **Moderate** | **Poor** |
| --- | --- | --- | --- |
| Stationary R-squared | ≥ 0.65 | 0.4 - 0.64 | < 0.4 |
| R-squared | ≥ 0.85 | 0.6 - 0.84 | < 0.6 |
| MAPE | ≤ 6% | 7% - 20% | > 20% |
| MaxAPE | ≤ 15% | 16% - 40% | > 40% |
| RMSE / MAE | Lower values (context-dependent) | Mid-range (context-dependent) | Higher values (context-dependent) |
| MaxAE | Lower values | Mid-range | Higher values |
| Normalized BIC | Lower values indicate better fit, with higher penalties for complexity | | |

***Table S3:*** *Overview about fit metrics of the ARIMA(2,1,2) model for the OECD countries with a predicted increasing trend. Countries are sorted alphabetically. The table evaluates model fit based on several metrics. A good fit (green colour) is indicated by high Stationary R-squared, high R-squared and low values for RMSE, MAPE, MaxAPE, MAE, MaxAE and Normalized BIC. A medium fit (yellow colour) falls between with moderate values for Stationary R-squared, R-squared, MAPE, MaxAPE, and MaxAE. A poor fit (orange colour) is characterized by low R-squared, low stationary R-squared, high errors (MAPE, MaxAPE, and MaxAE), and high RMSE, MAE and BIC values (Hyndman and Athanasopoulos 2018).*

| **country** | **Stationary R-squared** | **R-squared** | **RMSE** | **MAPE** | **MaxAPE** | **MAE** | **MaxAE** | **Normalized BIC** | **assessment of fit metrics** |
| --- | --- | --- | --- | --- | --- | --- | --- | --- | --- |
| **Austria** | 0.38738854 | 0.46980994 | 1.8939879 | 2.81056633 | 13.0376745 | 0.9918445 | 4.56318607 | 1.96814474 | poor |
| **Chile** | 0.55674029 | 0.45118121 | 11.7176871 | 19.9875864 | 70.5801455 | 7.99079055 | 20.8917231 | 5.61297436 | poor |
| **Czechia** | 0.63012926 | 0.99025042 | 1.88467467 | 5.50775425 | 29.1454507 | 1.16051219 | 5.7979336 | 1.5441764 | moderate |
| **Denmark** | 0.3584465 | 0.99717901 | 0.24614013 | 0.60382528 | 1.61910965 | 0.17887976 | 0.50840043 | -2.39473101 | good |
| **Estonia** | 0.50365335 | 0.99254093 | 0.69848851 | 2.64371954 | 7.92539559 | 0.50550764 | 1.37109344 | -0.15566538 | good |
| **Greece** | 0.90707834 | -3.17309422 | 33.4214148 | 70.0248114 | 482.673313 | 11.6012415 | 77.22773 | 7.77241009 | poor |
| **Hungary** | 0.90696928 | 0.9855211 | 0.56243576 | 1.78707908 | 6.17570061 | 0.36641756 | 1.08692331 | -0.50865189 | good |
| **Iceland** | 0.68472709 | 0.94532966 | 2.38878533 | 5.2426671 | 34.0859087 | 1.48286692 | 6.88535356 | 2.174787 | moderate |
| **Italy** | 0.32506523 | 0.61998228 | 0.90423187 | 1.27624429 | 4.49723255 | 0.52000118 | 1.76291516 | 0.4894366 | poor |
| **Latvia** | 0.55143512 | 0.96213696 | 0.80658863 | 1.81307723 | 5.29335761 | 0.48618703 | 1.29157926 | 0.3025252 | good |
| **Lithuania** | 0.43567454 | -0.73263789 | 6.01643817 | 11.0441472 | 65.6591047 | 2.84514628 | 15.1015941 | 4.27976636 | poor |
| **Portugal** | 0.37242304 | 0.98515871 | 0.94006454 | 2.32811557 | 6.49316547 | 0.65378101 | 2.50636187 | 0.31131828 | good |
| **Slovakia** | 0.64712139 | 0.88257517 | 3.75717611 | 9.85037775 | 46.869919 | 2.57093312 | 7.54085173 | 3.17701092 | moderate |
| **Slovenia** | 0.54531226 | 0.99276986 | 0.39363369 | 1.35214135 | 4.20493408 | 0.29718563 | 0.70226703 | -1.32305903 | good |
| **Spain** | 0.52723494 | 0.97276024 | 1.96003031 | 2.86935877 | 9.87692196 | 1.09653131 | 4.5433841 | 2.0125583 | good |
| **Turkey** | 0.76709099 | 0.91944341 | 1.44468058 | 4.7853194 | 17.8448439 | 0.9367159 | 3.06931315 | 1.35700315 | good |
| **United Kingdom** | 0.33979059 | 0.82852708 | 2.64402296 | 4.63188468 | 18.5044611 | 1.62736304 | 5.26329246 | 2.66674993 | moderate |
| **Croatia (non-OECD)** | 0.39738324 | 0.95541167 | 1.76299839 | 4.84678531 | 13.4973656 | 1.25938259 | 3.07700894 | 1.67564202 | moderate |

***Table S4:*** *Overview about fit metrics of the ARIMA(2,1,2) model for the OECD countries with a predicted decreasing trend. Countries are sorted alphabetically. The table evaluates model fit based on several metrics. A good fit (green colour) is indicated by high Stationary R-squared, high R-squared and low values for RMSE, MAPE, MaxAPE, MAE, MaxAE and Normalized BIC. A medium fit (yellow colour) falls between with moderate values for Stationary R-squared, R-squared, MAPE, MaxAPE, and MaxAE. A poor fit (orange colour) is characterized by low R-squared, low stationary R-squared, high errors (MAPE, MaxAPE, and MaxAE), and high RMSE, MAE and BIC values (Hyndman and Athanasopoulos 2018).*

| **country** | **Stationary R-squared** | **R-squared** | **RMSE** | **MAPE** | **MaxAPE** | **MAE** | **MaxAE** | **Normalized BIC** | **assessment of fit metrics** |
| --- | --- | --- | --- | --- | --- | --- | --- | --- | --- |
| **Australia** | 0.27692746 | 0.97833791 | 1.17536226 | 2.8331893 | 20.491814 | 0.78327275 | 3.72951015 | 0.7766458 | moderate |
| **Belgium** | 0.71408464 | 0.99406184 | 0.57658212 | 1.01828486 | 2.75994255 | 0.39125849 | 1.19478203 | -0.55597165 | good |
| **Canada** | 0.93032031 | 0.98338623 | 0.44737563 | 0.70576451 | 2.22310284 | 0.28809803 | 0.93148009 | -0.85469703 | good |
| **Costa Rica** | 0.60222608 | 0.50261115 | 3.49637479 | 8.67385145 | 46.0307012 | 2.27585606 | 8.88392533 | 3.10259977 | poor |
| **Finland** | 0.68387713 | 0.98561105 | 1.07181575 | 1.64739652 | 4.50566594 | 0.79307715 | 2.18975365 | 0.56021414 | good |
| **France** | 0.75897405 | -0.63783733 | 5.46274931 | 4.95446189 | 13.6016296 | 3.5455172 | 9.80677496 | 4.08667993 | moderate |
| **Germany** | 0.90048413 | 0.99268514 | 1.47240026 | 1.80412875 | 5.70293206 | 1.07138142 | 3.12063517 | 1.39608672 | good |
| **Israel** | 0.47185563 | -2.09640737 | 1.20452813 | 2.52201531 | 4.72446995 | 0.79344317 | 1.44096333 | 1.20613729 | poor |
| **Korea** | 0.78379361 | 0.74667481 | 2.88126842 | 5.19398764 | 15.1905181 | 1.79319634 | 4.08590692 | 3.15183901 | moderate |
| **Luxembourg** | 0.56655644 | 0.51148609 | 3.53600418 | 4.62914821 | 24.2098634 | 2.20220549 | 10.5555004 | 3.04585504 | poor |
| **Netherlands** | 0.6311977 | 0.97047012 | 0.60856812 | 1.50777175 | 7.44895665 | 0.39560108 | 1.80264751 | -0.52838144 | moderate |
| **Norway** | 0.48015204 | 0.96449869 | 0.89735277 | 1.47514397 | 4.72416564 | 0.6350401 | 1.67868636 | 0.20489336 | moderate |
| **Sweden** | 0.59877419 | 0.98119015 | 0.39872682 | 0.65488849 | 2.62330646 | 0.27658904 | 0.97062339 | -1.40402575 | good |

***Table S5:*** *Predicted values for the future consumption of the ATC class H from 2023 to 2040 for analysed OECD countries. The prediction is based on the ARIMA(1,2,1) model, performed in SPSS. Given is the prediction as well as the UCL and LCL. Countries are sorted alphabetically.*

| **country** | **parameter** | **2023** | **2024** | **2025** | **2026** | **2027** | **2028** | **2029** | **2030** | **2031** | **2032** | **2033** | **2034** | **2035** | **2036** | **2037** | **2038** | **2039** | **2040** |
| --- | --- | --- | --- | --- | --- | --- | --- | --- | --- | --- | --- | --- | --- | --- | --- | --- | --- | --- | --- |
| **Australia** | forecast | 39.91 | 40.04 | 40.15 | 40.22 | 40.27 | 40.28 | 40.27 | 40.22 | 40.15 | 40.05 | 39.92 | 39.76 | 39.57 | 39.35 | 39.10 | 38.82 | 38.51 | 38.17 |
|  | UCL | 42.63 | 43.34 | 43.95 | 44.50 | 44.98 | 45.41 | 45.79 | 46.12 | 46.41 | 46.66 | 46.87 | 47.04 | 47.17 | 47.27 | 47.33 | 47.35 | 47.34 | 47.29 |
|  | LCL | 37.19 | 36.74 | 36.34 | 35.95 | 35.55 | 35.16 | 34.75 | 34.33 | 33.89 | 33.44 | 32.97 | 32.47 | 31.96 | 31.42 | 30.86 | 30.28 | 29.68 | 29.05 |
| **Austria** | forecast | 32.18 | 31.95 | 31.76 | 31.62 | 31.52 | 31.48 | 31.48 | 31.53 | 31.63 | 31.77 | 31.97 | 32.21 | 32.50 | 32.83 | 33.22 | 33.65 | 34.13 | 34.66 |
|  | UCL | 38.15 | 39.52 | 40.77 | 41.97 | 43.13 | 44.28 | 45.43 | 46.59 | 47.76 | 48.95 | 50.17 | 51.41 | 52.69 | 54.00 | 55.35 | 56.73 | 58.16 | 59.63 |
|  | LCL | 26.22 | 24.37 | 22.74 | 21.26 | 19.91 | 18.67 | 17.53 | 16.47 | 15.50 | 14.59 | 13.76 | 13.00 | 12.30 | 11.66 | 11.09 | 10.56 | 10.10 | 9.68 |
| **Belgium** | forecast | 42.94 | 42.49 | 42.04 | 41.49 | 40.89 | 40.22 | 39.47 | 38.66 | 37.78 | 36.83 | 35.81 | 34.73 | 33.57 | 32.35 | 31.05 | 29.69 | 28.26 | 26.76 |
|  | UCL | 44.63 | 44.87 | 45.13 | 45.36 | 45.58 | 45.79 | 45.97 | 46.13 | 46.27 | 46.37 | 46.46 | 46.51 | 46.53 | 46.52 | 46.47 | 46.39 | 46.28 | 46.13 |
|  | LCL | 41.24 | 40.11 | 38.95 | 37.63 | 36.20 | 34.65 | 32.98 | 31.19 | 29.30 | 27.29 | 25.17 | 22.94 | 20.61 | 18.18 | 15.64 | 12.99 | 10.24 | 7.40 |
| **Canada** | forecast | 40.45 | 38.94 | 37.37 | 35.71 | 33.97 | 32.15 | 30.22 | 28.20 | 26.08 | 23.85 | 21.51 | 19.07 | 16.52 | 13.85 | 11.07 | 8.17 | 5.16 | 2.04 |
|  | UCL | 41.44 | 41.05 | 40.73 | 40.41 | 40.07 | 39.66 | 39.16 | 38.56 | 37.86 | 37.03 | 36.07 | 34.99 | 33.77 | 32.42 | 30.92 | 29.29 | 27.53 | 25.62 |
|  | LCL | 39.46 | 36.84 | 34.01 | 31.01 | 27.88 | 24.64 | 21.29 | 17.84 | 14.30 | 10.67 | 6.96 | 3.15 | -0.74 | -4.72 | -8.79 | -12.95 | -17.20 | -21.54 |
| **Chile** | forecast | 74.30 | 80.23 | 86.74 | 93.40 | 100.42 | 107.69 | 115.28 | 123.15 | 131.31 | 139.76 | 148.51 | 157.55 | 166.88 | 176.50 | 186.41 | 196.62 | 207.11 | 217.90 |
|  | UCL | 96.20 | 105.60 | 118.35 | 129.26 | 140.90 | 152.23 | 163.79 | 175.38 | 187.14 | 199.02 | 211.07 | 223.29 | 235.69 | 248.28 | 261.08 | 274.08 | 287.30 | 300.73 |
|  | LCL | 52.40 | 54.87 | 55.12 | 57.54 | 59.93 | 63.16 | 66.76 | 70.91 | 75.48 | 80.51 | 85.95 | 91.81 | 98.07 | 104.71 | 111.75 | 119.15 | 126.93 | 135.07 |
| **Costa Rica** | forecast | 31.09 | 28.07 | 28.62 | 27.10 | 26.54 | 25.32 | 24.30 | 23.03 | 21.75 | 20.32 | 18.82 | 17.21 | 15.52 | 13.72 | 11.83 | 9.84 | 7.75 | 5.57 |
|  | UCL | 37.73 | 35.53 | 37.83 | 37.37 | 38.06 | 37.90 | 37.95 | 37.67 | 37.37 | 36.88 | 36.29 | 35.56 | 34.72 | 33.76 | 32.68 | 31.49 | 30.18 | 28.76 |
|  | LCL | 24.45 | 20.62 | 19.41 | 16.83 | 15.02 | 12.74 | 10.66 | 8.39 | 6.13 | 3.76 | 1.36 | -1.13 | -3.69 | -6.32 | -9.03 | -11.82 | -14.69 | -17.63 |
| **Czechia** | forecast | 59.05 | 60.45 | 61.86 | 63.29 | 64.76 | 66.24 | 67.76 | 69.30 | 70.86 | 72.45 | 74.06 | 75.70 | 77.36 | 79.05 | 80.76 | 82.50 | 84.26 | 86.05 |
|  | UCL | 63.96 | 66.76 | 69.48 | 72.30 | 75.18 | 78.13 | 81.14 | 84.23 | 87.39 | 90.62 | 93.92 | 97.29 | 100.72 | 104.23 | 107.80 | 111.44 | 115.14 | 118.91 |
|  | LCL | 54.14 | 54.13 | 54.23 | 54.28 | 54.33 | 54.36 | 54.37 | 54.36 | 54.33 | 54.27 | 54.20 | 54.11 | 54.00 | 53.87 | 53.72 | 53.56 | 53.38 | 53.19 |
| **Denmark** | forecast | 38.24 | 38.66 | 39.06 | 39.45 | 39.82 | 40.17 | 40.51 | 40.84 | 41.14 | 41.44 | 41.72 | 41.98 | 42.22 | 42.46 | 42.67 | 42.87 | 43.06 | 43.23 |
|  | UCL | 38.99 | 39.61 | 40.19 | 40.74 | 41.26 | 41.75 | 42.22 | 42.68 | 43.11 | 43.52 | 43.91 | 44.28 | 44.64 | 44.98 | 45.29 | 45.60 | 45.88 | 46.14 |
|  | LCL | 37.50 | 37.71 | 37.93 | 38.16 | 38.38 | 38.59 | 38.80 | 39.00 | 39.18 | 39.36 | 39.52 | 39.67 | 39.81 | 39.94 | 40.05 | 40.15 | 40.24 | 40.31 |
| **Estonia** | forecast | 33.80 | 35.02 | 36.22 | 37.47 | 38.75 | 40.08 | 41.43 | 42.83 | 44.26 | 45.73 | 47.23 | 48.77 | 50.35 | 51.96 | 53.61 | 55.30 | 57.02 | 58.78 |
|  | UCL | 35.26 | 37.17 | 39.26 | 41.46 | 43.78 | 46.20 | 48.73 | 51.36 | 54.09 | 56.92 | 59.84 | 62.86 | 65.96 | 69.15 | 72.42 | 75.78 | 79.23 | 82.75 |
|  | LCL | 32.34 | 32.87 | 33.17 | 33.48 | 33.73 | 33.95 | 34.14 | 34.29 | 34.43 | 34.53 | 34.62 | 34.69 | 34.74 | 34.78 | 34.80 | 34.82 | 34.82 | 34.81 |
| **Finland** | forecast | 56.10 | 56.27 | 56.28 | 56.25 | 56.12 | 55.92 | 55.63 | 55.27 | 54.83 | 54.31 | 53.71 | 53.03 | 52.27 | 51.43 | 50.51 | 49.51 | 48.43 | 47.27 |
|  | UCL | 58.72 | 59.46 | 59.92 | 60.33 | 60.61 | 60.81 | 60.91 | 60.91 | 60.83 | 60.66 | 60.40 | 60.05 | 59.62 | 59.10 | 58.50 | 57.82 | 57.05 | 56.19 |
|  | LCL | 53.47 | 53.08 | 52.64 | 52.16 | 51.62 | 51.03 | 50.36 | 49.63 | 48.83 | 47.96 | 47.02 | 46.00 | 44.91 | 43.75 | 42.52 | 41.20 | 39.82 | 38.35 |
| **France** | forecast | 61.63 | 49.05 | 49.28 | 36.88 | 34.29 | 21.71 | 16.62 | 3.60 | -3.76 | -17.45 | -26.89 | -41.40 | -52.77 | -68.24 | -81.42 | -97.95 | -112.86 | -130.53 |
|  | UCL | 71.05 | 62.14 | 62.71 | 52.96 | 50.84 | 40.35 | 35.82 | 24.53 | 17.80 | 5.60 | -3.16 | -16.38 | -27.05 | -41.36 | -53.82 | -69.30 | -83.48 | -100.18 |
|  | LCL | 52.22 | 35.95 | 35.85 | 20.80 | 17.74 | 3.08 | -2.58 | -17.34 | -25.33 | -40.49 | -50.61 | -66.42 | -78.49 | -95.12 | -109.02 | -126.61 | -142.24 | -160.88 |
| **Germany** | forecast | 87.40 | 87.25 | 87.08 | 86.87 | 86.58 | 86.20 | 85.72 | 85.14 | 84.46 | 83.67 | 82.78 | 81.79 | 80.69 | 79.49 | 78.18 | 76.76 | 75.24 | 73.62 |
|  | UCL | 92.80 | 94.96 | 96.90 | 98.61 | 100.09 | 101.36 | 102.43 | 103.32 | 104.03 | 104.59 | 104.99 | 105.24 | 105.35 | 105.33 | 105.16 | 104.87 | 104.44 | 103.89 |
|  | LCL | 82.00 | 79.54 | 77.27 | 75.13 | 73.07 | 71.04 | 69.01 | 66.97 | 64.89 | 62.76 | 60.58 | 58.34 | 56.03 | 53.65 | 51.19 | 48.66 | 46.05 | 43.35 |
| **Greece** | forecast | 33.87 | 36.57 | 39.61 | 42.75 | 46.08 | 49.57 | 53.24 | 57.07 | 61.07 | 65.24 | 69.58 | 74.09 | 78.76 | 83.60 | 88.62 | 93.80 | 99.14 | 104.66 |
|  | UCL | 37.15 | 40.09 | 43.86 | 47.55 | 51.44 | 55.44 | 59.60 | 63.90 | 68.36 | 72.98 | 77.76 | 82.70 | 87.80 | 93.07 | 98.50 | 104.10 | 109.86 | 115.79 |
|  | LCL | 30.59 | 33.04 | 35.35 | 37.94 | 40.72 | 43.71 | 46.88 | 50.24 | 53.78 | 57.50 | 61.40 | 65.47 | 69.72 | 74.14 | 78.73 | 83.50 | 88.43 | 93.54 |
| **Hungary** | forecast | 27.35 | 27.62 | 27.87 | 28.10 | 28.30 | 28.47 | 28.62 | 28.74 | 28.84 | 28.91 | 28.95 | 28.97 | 28.96 | 28.93 | 28.87 | 28.79 | 28.67 | 28.54 |
|  | UCL | 28.55 | 29.65 | 30.83 | 32.07 | 33.37 | 34.73 | 36.14 | 37.60 | 39.10 | 40.64 | 42.22 | 43.83 | 45.48 | 47.16 | 48.86 | 50.60 | 52.36 | 54.15 |
|  | LCL | 26.15 | 25.60 | 24.92 | 24.13 | 23.23 | 22.22 | 21.10 | 19.89 | 18.58 | 17.18 | 15.69 | 14.11 | 12.45 | 10.70 | 8.88 | 6.97 | 4.99 | 2.93 |
| **Iceland** | forecast | 43.77 | 43.80 | 44.50 | 44.89 | 45.39 | 45.82 | 46.26 | 46.67 | 47.07 | 47.45 | 47.82 | 48.17 | 48.50 | 48.82 | 49.12 | 49.41 | 49.68 | 49.93 |
|  | UCL | 48.53 | 49.38 | 51.25 | 52.53 | 53.90 | 55.14 | 56.35 | 57.49 | 58.60 | 59.66 | 60.69 | 61.68 | 62.64 | 63.57 | 64.47 | 65.34 | 66.18 | 66.99 |
|  | LCL | 39.01 | 38.23 | 37.74 | 37.26 | 36.87 | 36.50 | 36.17 | 35.85 | 35.54 | 35.24 | 34.94 | 34.65 | 34.36 | 34.06 | 33.77 | 33.47 | 33.17 | 32.87 |
| **Israel** | forecast | 32.05 | 32.00 | 31.91 | 31.77 | 31.59 | 31.36 | 31.09 | 30.77 | 30.41 | 30.00 | 29.55 | 29.06 | 28.52 | 27.94 | 27.31 | 26.64 | 25.93 | 25.17 |
|  | UCL | 35.16 | 36.44 | 37.50 | 38.39 | 39.15 | 39.79 | 40.34 | 40.80 | 41.17 | 41.47 | 41.69 | 41.83 | 41.91 | 41.92 | 41.87 | 41.76 | 41.58 | 41.35 |
|  | LCL | 28.94 | 27.57 | 26.32 | 25.15 | 24.03 | 22.92 | 21.83 | 20.74 | 19.64 | 18.54 | 17.42 | 16.29 | 15.13 | 13.96 | 12.75 | 11.53 | 10.27 | 8.99 |
| **Italy** | forecast | 43.96 | 44.76 | 45.55 | 46.38 | 47.25 | 48.16 | 49.12 | 50.11 | 51.14 | 52.21 | 53.32 | 54.46 | 55.65 | 56.88 | 58.14 | 59.45 | 60.80 | 62.18 |
|  | UCL | 45.88 | 47.34 | 48.73 | 50.11 | 51.49 | 52.87 | 54.27 | 55.68 | 57.12 | 58.57 | 60.05 | 61.56 | 63.09 | 64.65 | 66.24 | 67.87 | 69.52 | 71.20 |
|  | LCL | 42.03 | 42.19 | 42.36 | 42.65 | 43.02 | 43.46 | 43.96 | 44.53 | 45.16 | 45.84 | 46.58 | 47.37 | 48.21 | 49.10 | 50.05 | 51.04 | 52.07 | 53.16 |
| **Korea** | forecast | 33.97 | 31.94 | 31.25 | 28.44 | 26.95 | 23.35 | 21.07 | 16.68 | 13.60 | 8.42 | 4.55 | -1.42 | -6.08 | -12.84 | -18.30 | -25.85 | -32.11 | -40.44 |
|  | UCL | 47.38 | 54.49 | 64.09 | 72.98 | 84.14 | 94.36 | 106.71 | 117.97 | 131.28 | 143.39 | 157.46 | 170.27 | 185.00 | 198.39 | 213.66 | 227.55 | 243.27 | 257.59 |
|  | LCL | 20.55 | 9.40 | -1.58 | -16.10 | -30.23 | -47.65 | -64.57 | -84.61 | -104.07 | -126.54 | -148.36 | -173.10 | -197.16 | -224.07 | -250.26 | -279.25 | -307.49 | -338.47 |
| **Latvia** | forecast | 35.52 | 36.97 | 38.43 | 39.91 | 41.41 | 42.92 | 44.46 | 46.02 | 47.59 | 49.19 | 50.80 | 52.43 | 54.08 | 55.75 | 57.44 | 59.15 | 60.88 | 62.63 |
|  | UCL | 37.27 | 39.20 | 41.20 | 43.14 | 45.09 | 47.03 | 48.98 | 50.93 | 52.89 | 54.87 | 56.85 | 58.85 | 60.86 | 62.90 | 64.94 | 67.01 | 69.10 | 71.20 |
|  | LCL | 33.78 | 34.73 | 35.65 | 36.67 | 37.72 | 38.81 | 39.94 | 41.10 | 42.29 | 43.51 | 44.75 | 46.01 | 47.30 | 48.61 | 49.94 | 51.29 | 52.66 | 54.05 |
| **Lithuania** | forecast | 35.69 | 37.13 | 38.65 | 40.30 | 42.06 | 43.95 | 45.95 | 48.07 | 50.31 | 52.68 | 55.15 | 57.75 | 60.47 | 63.31 | 66.26 | 69.34 | 72.53 | 75.84 |
|  | UCL | 51.47 | 56.62 | 61.44 | 66.15 | 70.78 | 75.37 | 79.95 | 84.52 | 89.11 | 93.72 | 98.38 | 103.07 | 107.82 | 112.62 | 117.49 | 122.42 | 127.43 | 132.50 |
|  | LCL | 19.91 | 17.63 | 15.85 | 14.44 | 13.34 | 12.52 | 11.96 | 11.63 | 11.52 | 11.63 | 11.93 | 12.43 | 13.12 | 13.99 | 15.03 | 16.25 | 17.63 | 19.18 |
| **Luxembourg** | forecast | 36.83 | 33.73 | 30.39 | 26.77 | 22.86 | 18.66 | 14.19 | 9.43 | 4.40 | -0.93 | -6.53 | -12.41 | -18.58 | -25.03 | -31.76 | -38.77 | -46.07 | -53.65 |
|  | UCL | 44.28 | 43.70 | 42.63 | 41.03 | 38.99 | 36.55 | 33.74 | 30.57 | 27.05 | 23.19 | 18.99 | 14.47 | 9.63 | 4.46 | -1.02 | -6.82 | -12.93 | -19.35 |
|  | LCL | 29.39 | 23.77 | 18.16 | 12.50 | 6.72 | 0.78 | -5.36 | -11.70 | -18.26 | -25.04 | -32.05 | -39.30 | -46.78 | -54.52 | -62.50 | -70.73 | -79.21 | -87.95 |
| **Netherlands** | forecast | 29.59 | 29.43 | 29.22 | 28.95 | 28.62 | 28.23 | 27.78 | 27.27 | 26.71 | 26.09 | 25.41 | 24.67 | 23.88 | 23.03 | 22.11 | 21.14 | 20.12 | 19.03 |
|  | UCL | 31.06 | 31.23 | 31.28 | 31.26 | 31.17 | 31.01 | 30.78 | 30.48 | 30.12 | 29.69 | 29.20 | 28.65 | 28.04 | 27.36 | 26.62 | 25.81 | 24.95 | 24.03 |
|  | LCL | 28.11 | 27.64 | 27.16 | 26.63 | 26.06 | 25.44 | 24.78 | 24.07 | 23.30 | 22.49 | 21.62 | 20.70 | 19.72 | 18.69 | 17.61 | 16.47 | 15.28 | 14.04 |
| **Norway** | forecast | 49.27 | 49.23 | 49.01 | 48.76 | 48.42 | 48.02 | 47.56 | 47.02 | 46.42 | 45.75 | 45.02 | 44.21 | 43.34 | 42.40 | 41.39 | 40.32 | 39.17 | 37.96 |
|  | UCL | 50.96 | 51.40 | 51.65 | 51.80 | 51.85 | 51.81 | 51.69 | 51.49 | 51.21 | 50.85 | 50.41 | 49.90 | 49.31 | 48.66 | 47.93 | 47.12 | 46.25 | 45.30 |
|  | LCL | 47.58 | 47.07 | 46.38 | 45.71 | 45.00 | 44.24 | 43.43 | 42.56 | 41.64 | 40.66 | 39.62 | 38.52 | 37.36 | 36.14 | 34.86 | 33.51 | 32.10 | 30.62 |
| **Portugal** | forecast | 45.76 | 47.78 | 49.86 | 52.03 | 54.26 | 56.58 | 58.97 | 61.44 | 63.98 | 66.60 | 69.30 | 72.07 | 74.92 | 77.85 | 80.85 | 83.93 | 87.09 | 90.32 |
|  | UCL | 47.65 | 50.63 | 53.49 | 56.32 | 59.16 | 62.04 | 64.97 | 67.94 | 70.97 | 74.05 | 77.20 | 80.41 | 83.68 | 87.01 | 90.42 | 93.89 | 97.42 | 101.03 |
|  | LCL | 43.87 | 44.92 | 46.24 | 47.73 | 49.37 | 51.12 | 52.98 | 54.94 | 57.00 | 59.15 | 61.40 | 63.74 | 66.17 | 68.68 | 71.29 | 73.98 | 76.75 | 79.61 |
| **Slovakia** | forecast | 36.80 | 39.23 | 41.73 | 44.31 | 46.99 | 49.75 | 52.61 | 55.56 | 58.61 | 61.75 | 64.98 | 68.31 | 71.74 | 75.26 | 78.87 | 82.58 | 86.38 | 90.28 |
|  | UCL | 50.32 | 58.02 | 65.21 | 72.03 | 78.60 | 84.98 | 91.25 | 97.43 | 103.57 | 109.67 | 115.77 | 121.86 | 127.97 | 134.09 | 140.24 | 146.43 | 152.65 | 158.91 |
|  | LCL | 23.29 | 20.44 | 18.25 | 16.59 | 15.37 | 14.52 | 13.97 | 13.69 | 13.65 | 13.82 | 14.20 | 14.76 | 15.51 | 16.42 | 17.49 | 18.73 | 20.11 | 21.65 |
| **Slovenia** | forecast | 30.70 | 31.60 | 32.54 | 33.47 | 34.40 | 35.34 | 36.28 | 37.22 | 38.16 | 39.10 | 40.05 | 41.00 | 41.95 | 42.91 | 43.86 | 44.82 | 45.78 | 46.75 |
|  | UCL | 31.44 | 32.53 | 33.67 | 34.78 | 35.88 | 36.97 | 38.06 | 39.14 | 40.22 | 41.30 | 42.37 | 43.45 | 44.52 | 45.59 | 46.66 | 47.73 | 48.80 | 49.87 |
|  | LCL | 29.95 | 30.67 | 31.40 | 32.16 | 32.93 | 33.71 | 34.50 | 35.29 | 36.10 | 36.91 | 37.73 | 38.56 | 39.39 | 40.23 | 41.07 | 41.91 | 42.76 | 43.62 |
| **Spain** | forecast | 57.88 | 61.19 | 64.63 | 68.21 | 71.92 | 75.76 | 79.74 | 83.86 | 88.11 | 92.49 | 97.01 | 101.67 | 106.46 | 111.38 | 116.44 | 121.63 | 126.96 | 132.43 |
|  | UCL | 62.06 | 67.28 | 72.29 | 77.24 | 82.22 | 87.25 | 92.35 | 97.54 | 102.82 | 108.20 | 113.68 | 119.27 | 124.97 | 130.78 | 136.71 | 142.76 | 148.93 | 155.21 |
|  | LCL | 53.70 | 55.09 | 56.98 | 59.17 | 61.62 | 64.28 | 67.14 | 70.18 | 73.40 | 76.79 | 80.35 | 84.07 | 87.94 | 91.98 | 96.17 | 100.51 | 105.00 | 109.64 |
| **Sweden** | forecast | 43.72 | 43.26 | 42.69 | 42.05 | 41.34 | 40.54 | 39.67 | 38.72 | 37.69 | 36.58 | 35.40 | 34.13 | 32.79 | 31.38 | 29.88 | 28.31 | 26.65 | 24.92 |
|  | UCL | 44.49 | 44.21 | 43.85 | 43.38 | 42.83 | 42.18 | 41.45 | 40.64 | 39.75 | 38.77 | 37.71 | 36.56 | 35.34 | 34.04 | 32.65 | 31.18 | 29.64 | 28.01 |
|  | LCL | 42.95 | 42.31 | 41.53 | 40.73 | 39.85 | 38.90 | 37.88 | 36.79 | 35.63 | 34.40 | 33.09 | 31.71 | 30.25 | 28.72 | 27.11 | 25.43 | 23.67 | 21.84 |
| **Turkey** | forecast | 33.74 | 35.71 | 37.89 | 40.09 | 42.43 | 44.82 | 47.32 | 49.90 | 52.58 | 55.33 | 58.18 | 61.12 | 64.15 | 67.26 | 70.46 | 73.75 | 77.13 | 80.60 |
|  | UCL | 36.99 | 39.80 | 42.45 | 45.24 | 48.05 | 50.94 | 53.90 | 56.92 | 60.02 | 63.20 | 66.44 | 69.77 | 73.17 | 76.65 | 80.21 | 83.85 | 87.57 | 91.37 |
|  | LCL | 30.49 | 31.61 | 33.34 | 34.95 | 36.81 | 38.70 | 40.75 | 42.88 | 45.13 | 47.47 | 49.92 | 52.47 | 55.12 | 57.87 | 60.71 | 63.66 | 66.70 | 69.83 |
| **United Kingdom** | forecast | 48.13 | 50.38 | 52.76 | 55.29 | 57.95 | 60.75 | 63.68 | 66.75 | 69.96 | 73.30 | 76.79 | 80.40 | 84.16 | 88.05 | 92.08 | 96.24 | 100.55 | 104.98 |
|  | UCL | 55.89 | 60.20 | 64.41 | 68.60 | 72.81 | 77.08 | 81.42 | 85.83 | 90.34 | 94.93 | 99.62 | 104.42 | 109.32 | 114.34 | 119.46 | 124.69 | 130.05 | 135.52 |
|  | LCL | 40.37 | 40.56 | 41.12 | 41.98 | 43.08 | 44.41 | 45.94 | 47.67 | 49.58 | 51.68 | 53.95 | 56.39 | 58.99 | 61.77 | 64.70 | 67.79 | 71.04 | 74.45 |
| **Croatia (non-OECD)** | forecast | 49.43 | 52.74 | 56.19 | 59.79 | 63.54 | 67.43 | 71.46 | 75.65 | 79.98 | 84.45 | 89.08 | 93.85 | 98.76 | 103.82 | 109.03 | 114.38 | 119.88 | 125.52 |
|  | UCL | 54.82 | 59.53 | 64.23 | 68.96 | 73.77 | 78.66 | 83.65 | 88.75 | 93.95 | 99.27 | 104.71 | 110.26 | 115.95 | 121.75 | 127.69 | 133.75 | 139.94 | 146.26 |
|  | LCL | 44.04 | 45.94 | 48.15 | 50.62 | 53.30 | 56.19 | 59.28 | 62.55 | 66.01 | 69.64 | 73.45 | 77.43 | 81.57 | 85.89 | 90.37 | 95.01 | 99.82 | 104.79 |

Supplemental Figures

***Fig. S1:*** *Sequence chart of the development of consumption of ATC class H for Australia. Depicted is the original series (a), the first-degree differentiation (b), and the second-degree differentiation (c). A mean line is overlaid to facilitate the assessment of trends and determine whether the data is stationary or non-stationary. The original time series (a) clearly displays a strong trend, which diminishes progressively with each level of differentiation, ultimately leading to stationarity in (c).*


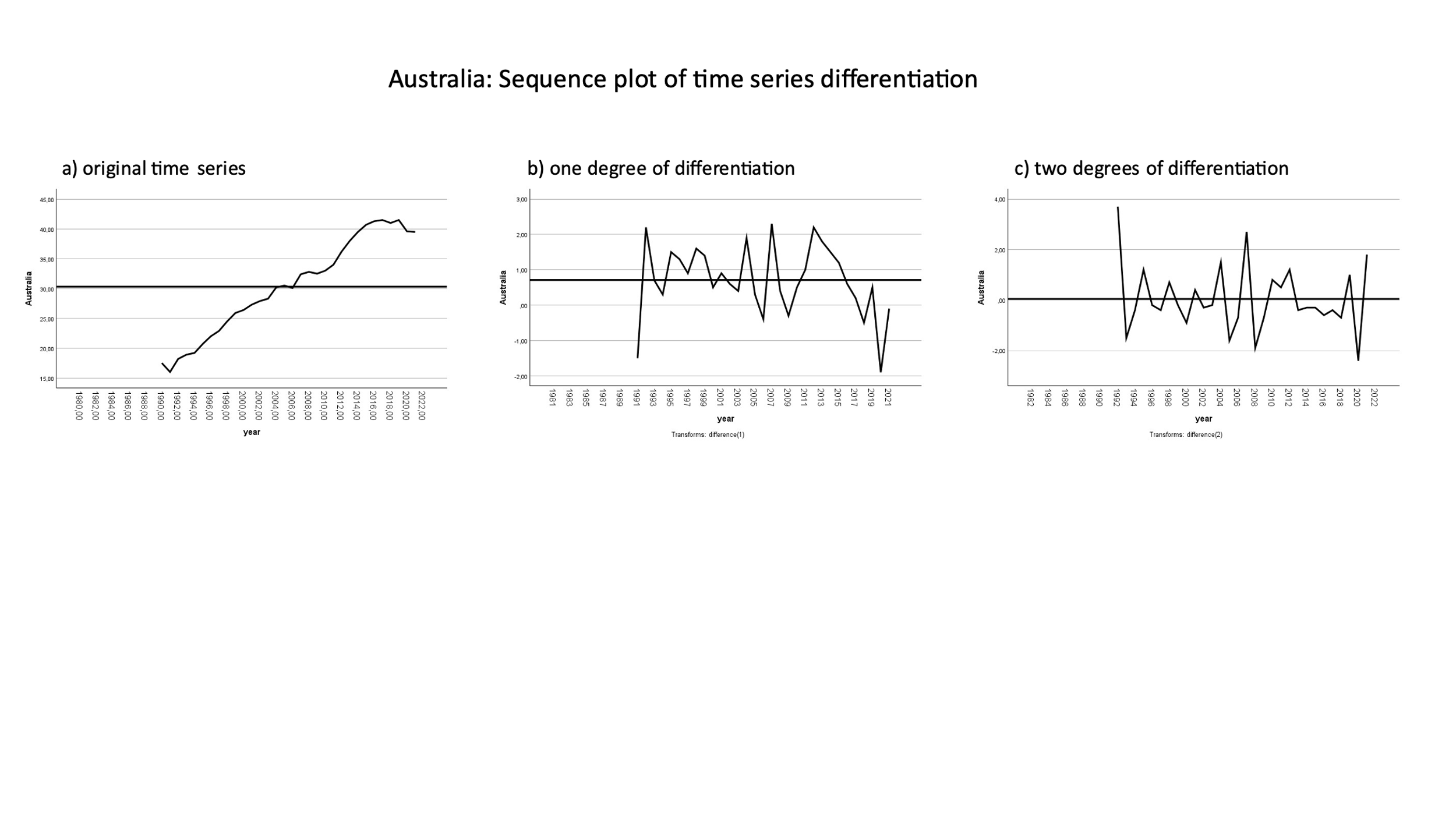


***Fig. S2:*** *Sequence chart of the development of consumption of ATC class H for Austria. Depicted is the original series (a), the first-degree differentiation (b), and the second-degree differentiation (c). A mean line is overlaid to facilitate the assessment of trends and determine whether the data is stationary or non-stationary. The original time series (a) clearly displays a strong trend, which diminishes progressively with each level of differentiation, ultimately leading to stationarity in (c).*


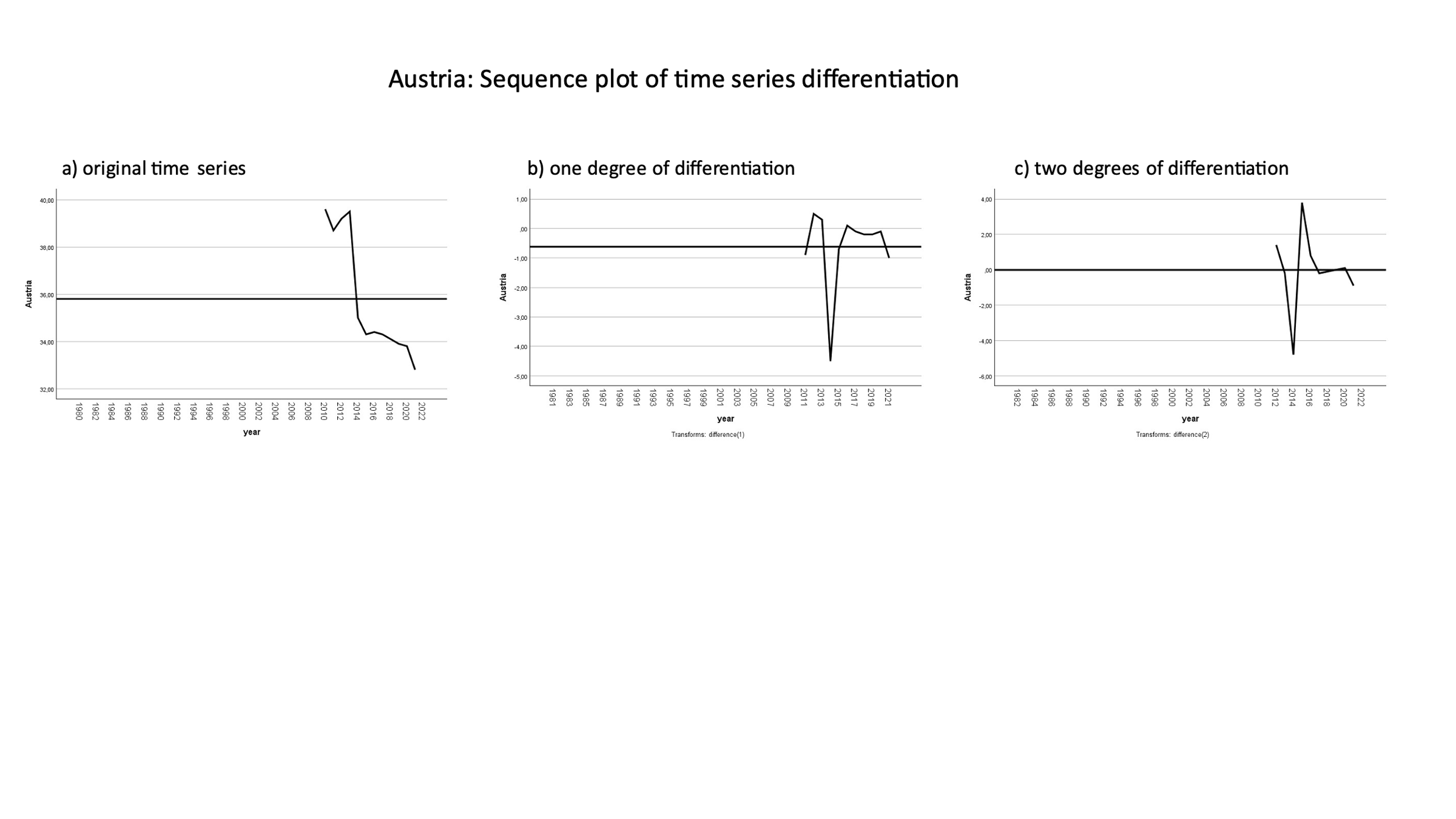


***Fig. S3:*** *Sequence chart of the development of consumption of ATC class H for Belgium. Depicted is the original series (a), the first-degree differentiation (b), and the second-degree differentiation (c). A mean line is overlaid to facilitate the assessment of trends and determine whether the data is stationary or non-stationary. The original time series (a) clearly displays a strong trend, which diminishes progressively with each level of differentiation, ultimately leading to stationarity in (c).*
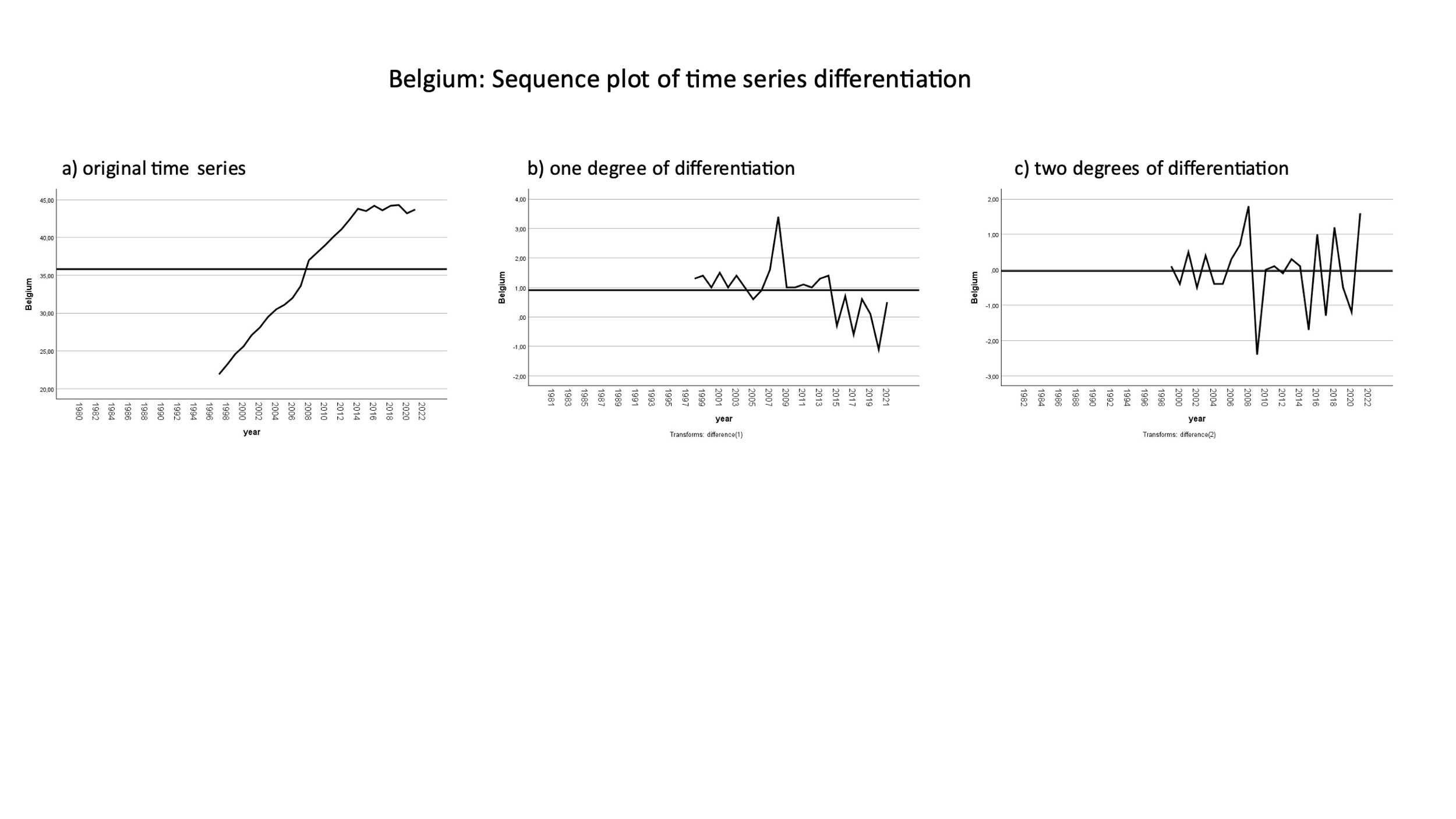


***Fig. S4:*** *Sequence chart of the development of consumption of ATC class H for Canada. Depicted is the original series (a), the first-degree differentiation (b), and the second-degree differentiation (c). A mean line is overlaid to facilitate the assessment of trends and determine whether the data is stationary or non-stationary. The original time series (a) clearly displays a strong trend, which diminishes progressively with each level of differentiation, ultimately leading to stationarity in (c).*


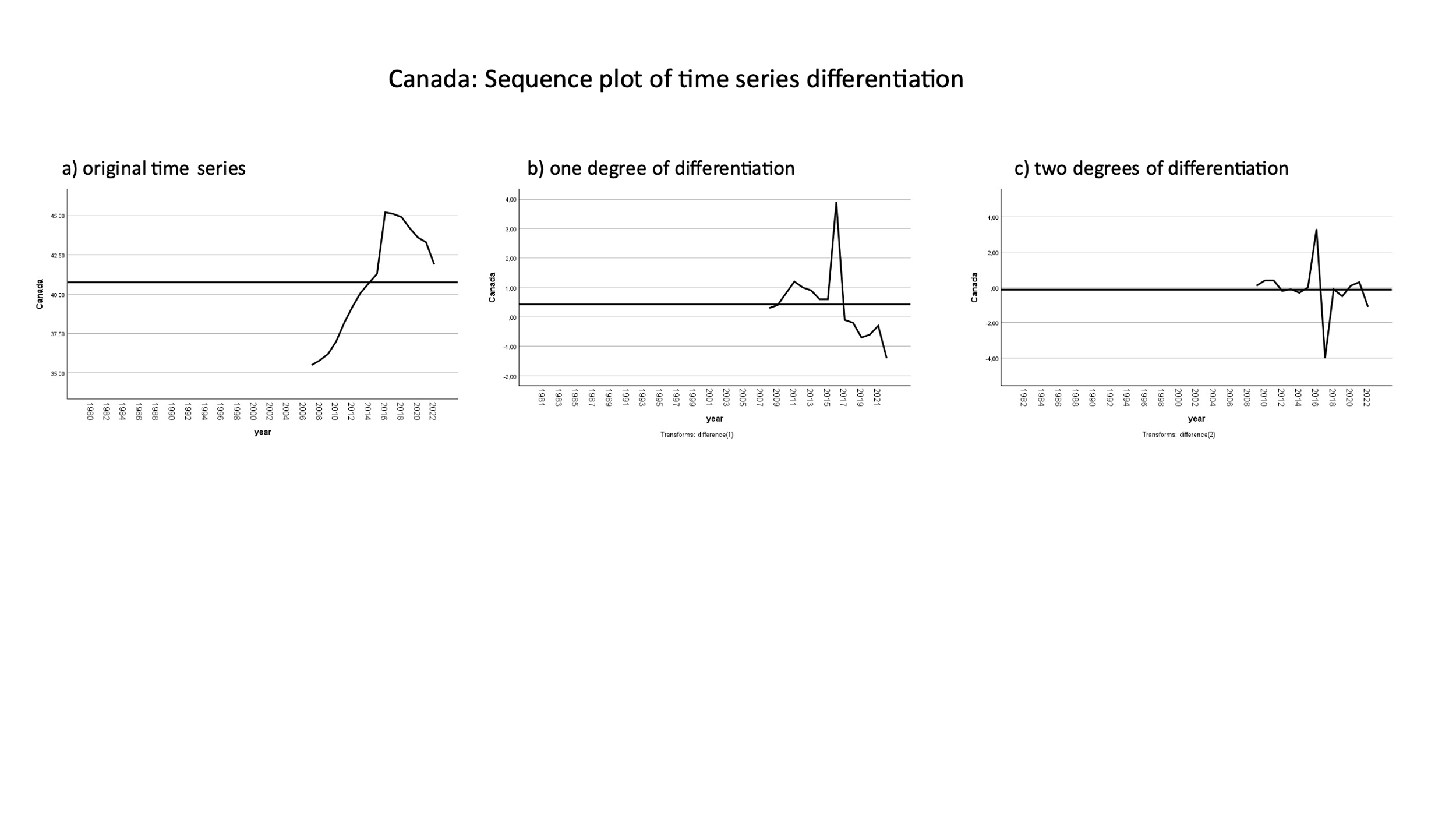


***Fig. S5:*** *Sequence chart of the development of consumption of ATC class H for Chile. Depicted is the original series (a), the first-degree differentiation (b), and the second-degree differentiation (c). A mean line is overlaid to facilitate the assessment of trends and determine whether the data is stationary or non-stationary. The original time series (a) clearly displays a strong trend, which diminishes progressively with each level of differentiation, ultimately leading to stationarity in (c).*
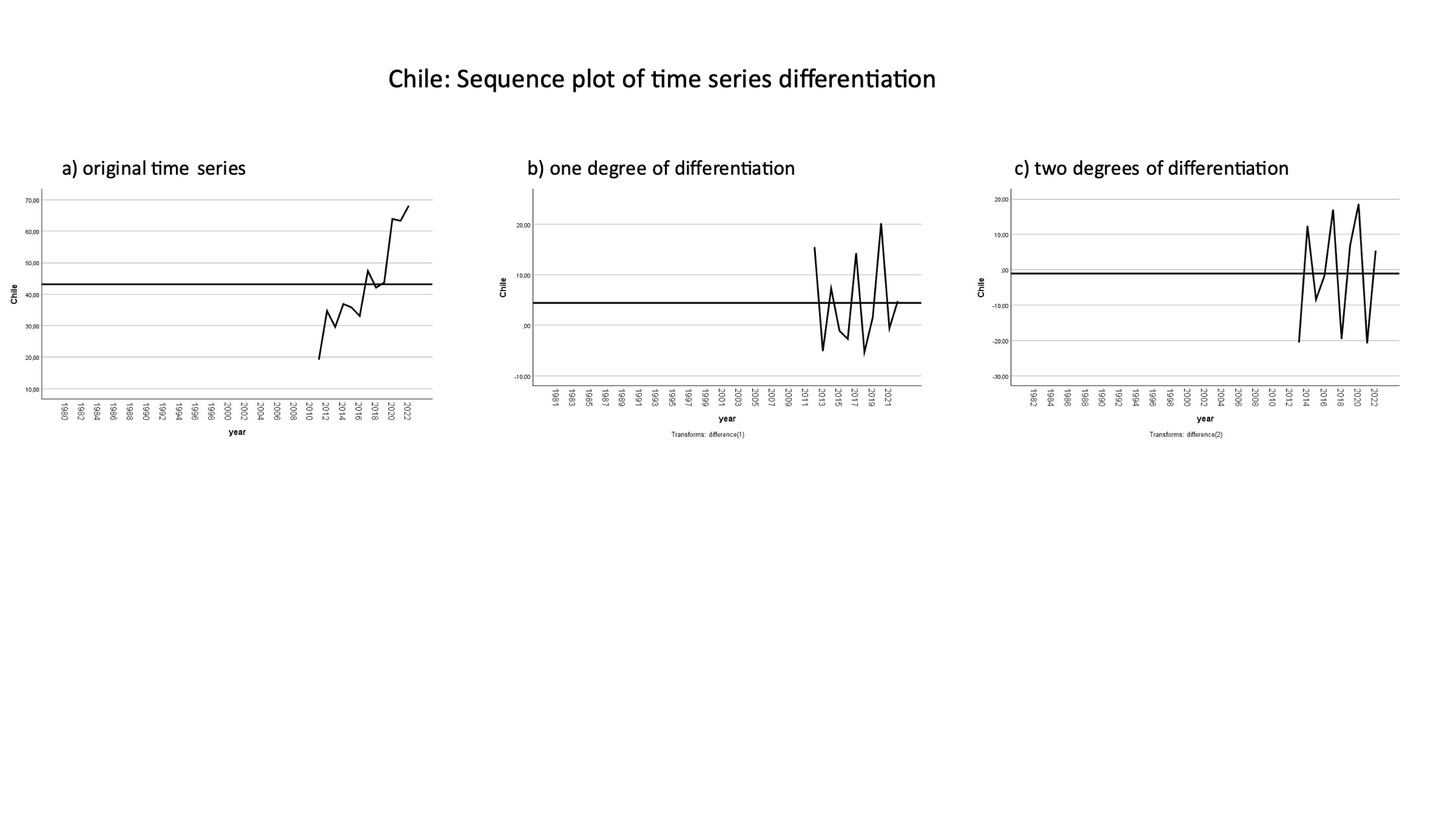


***Fig. S6:*** *Sequence chart of the development of consumption of ATC class H for Costa Rica. Depicted is the original series (a), the first-degree differentiation (b), and the second-degree differentiation (c). A mean line is overlaid to facilitate the assessment of trends and determine whether the data is stationary or non-stationary. The original time series (a) clearly displays a strong trend, which diminishes progressively with each level of differentiation, ultimately leading to stationarity in (c).*

*
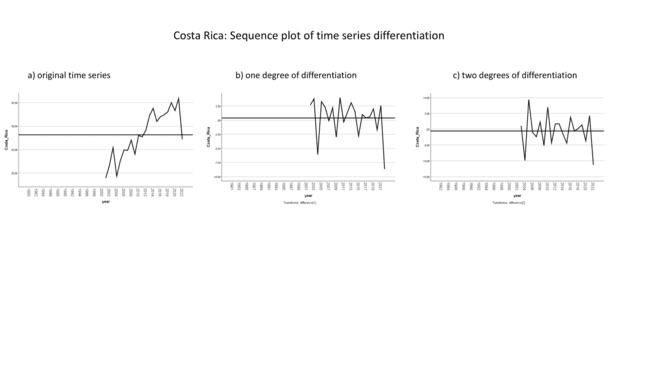
*

***Fig. S7:*** *Sequence chart of the development of consumption of ATC class H for Czechia. Depicted is the original series (a), the first-degree differentiation (b), and the second-degree differentiation (c). A mean line is overlaid to facilitate the assessment of trends and determine whether the data is stationary or non-stationary. The original time series (a) clearly displays a strong trend, which diminishes progressively with each level of differentiation, ultimately leading to stationarity in (c).*


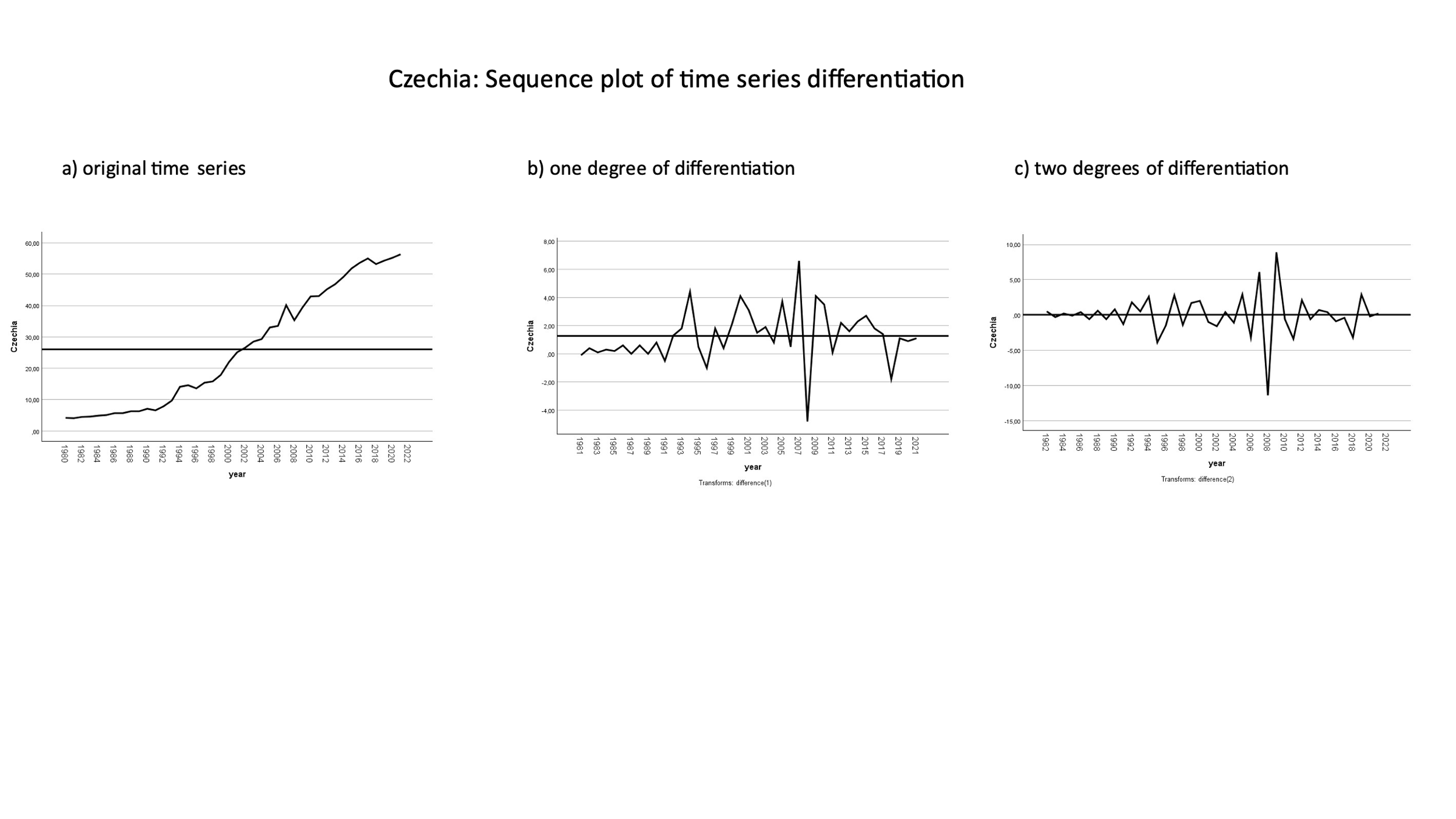


***Fig. S8:*** *Sequence chart of the development of consumption of ATC class H for Denmark. Depicted is the original series (a), the first-degree differentiation (b), and the second-degree differentiation (c). A mean line is overlaid to facilitate the assessment of trends and determine whether the data is stationary or non-stationary. The original time series (a) clearly displays a strong trend, which diminishes progressively with each level of differentiation, ultimately leading to stationarity in (c).*
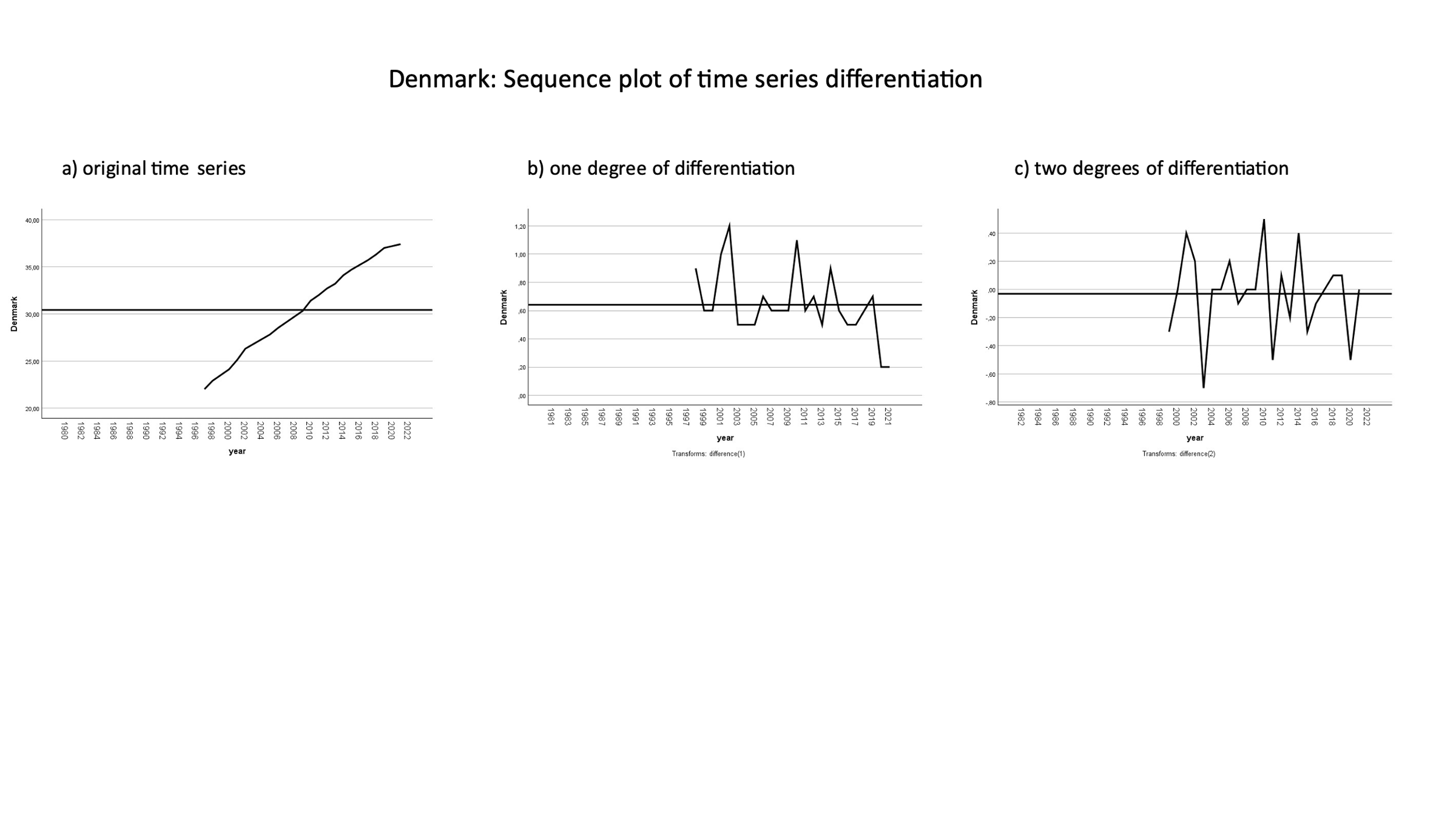


***Fig. S9:*** *Sequence chart of the development of consumption of ATC class H for Estonia. Depicted is the original series (a), the first-degree differentiation (b), and the second-degree differentiation (c). A mean line is overlaid to facilitate the assessment of trends and determine whether the data is stationary or non-stationary. The original time series (a) clearly displays a strong trend, which diminishes progressively with each level of differentiation, ultimately leading to stationarity in (c).*


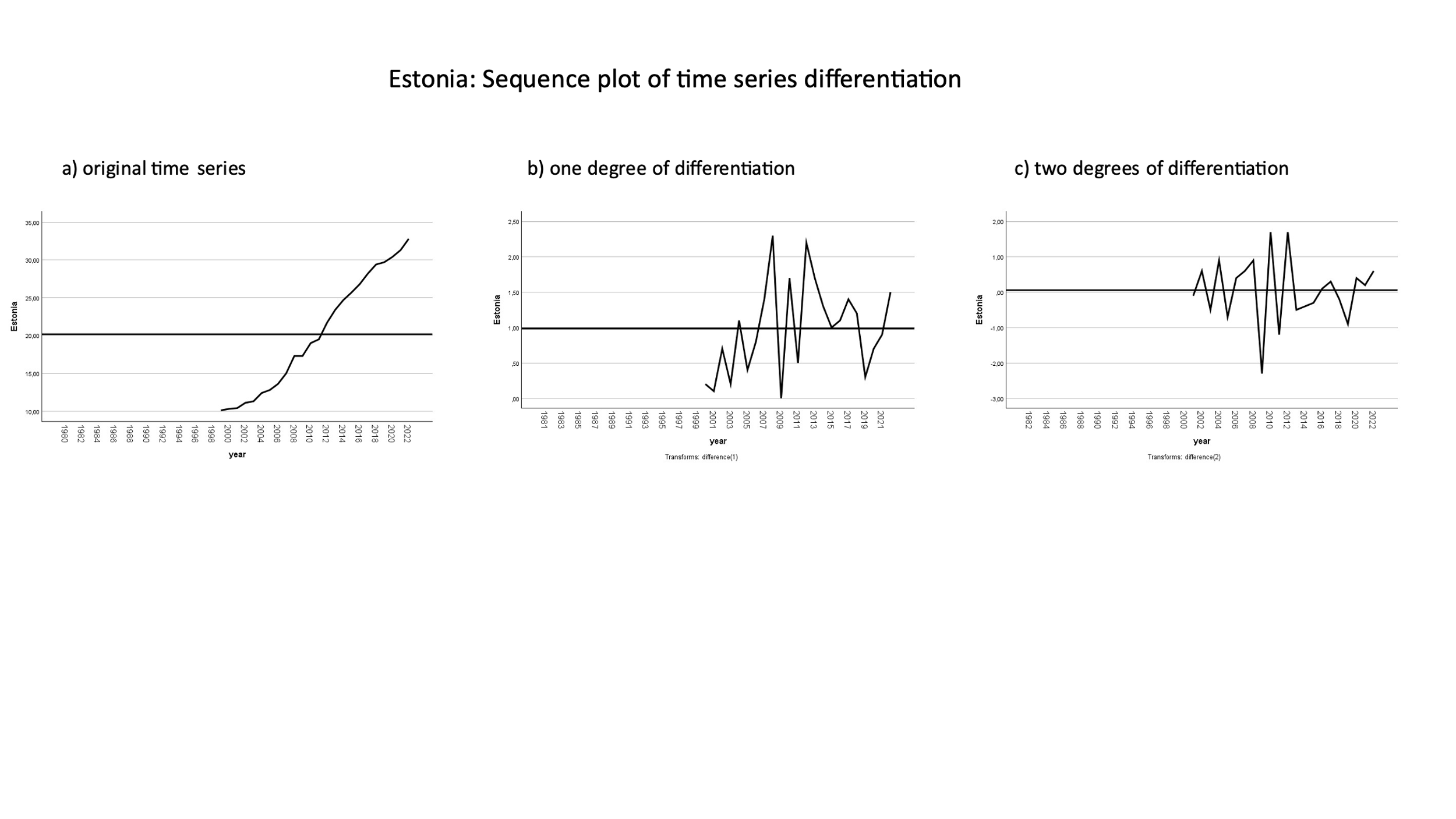


***Fig. S10:*** *Sequence chart of the development of consumption of ATC class H for Finland. Depicted is the original series (a), the first-degree differentiation (b), and the second-degree differentiation (c). A mean line is overlaid to facilitate the assessment of trends and determine whether the data is stationary or non-stationary. The original time series (a) clearly displays a strong trend, which diminishes progressively with each level of differentiation, ultimately leading to stationarity in (c).*


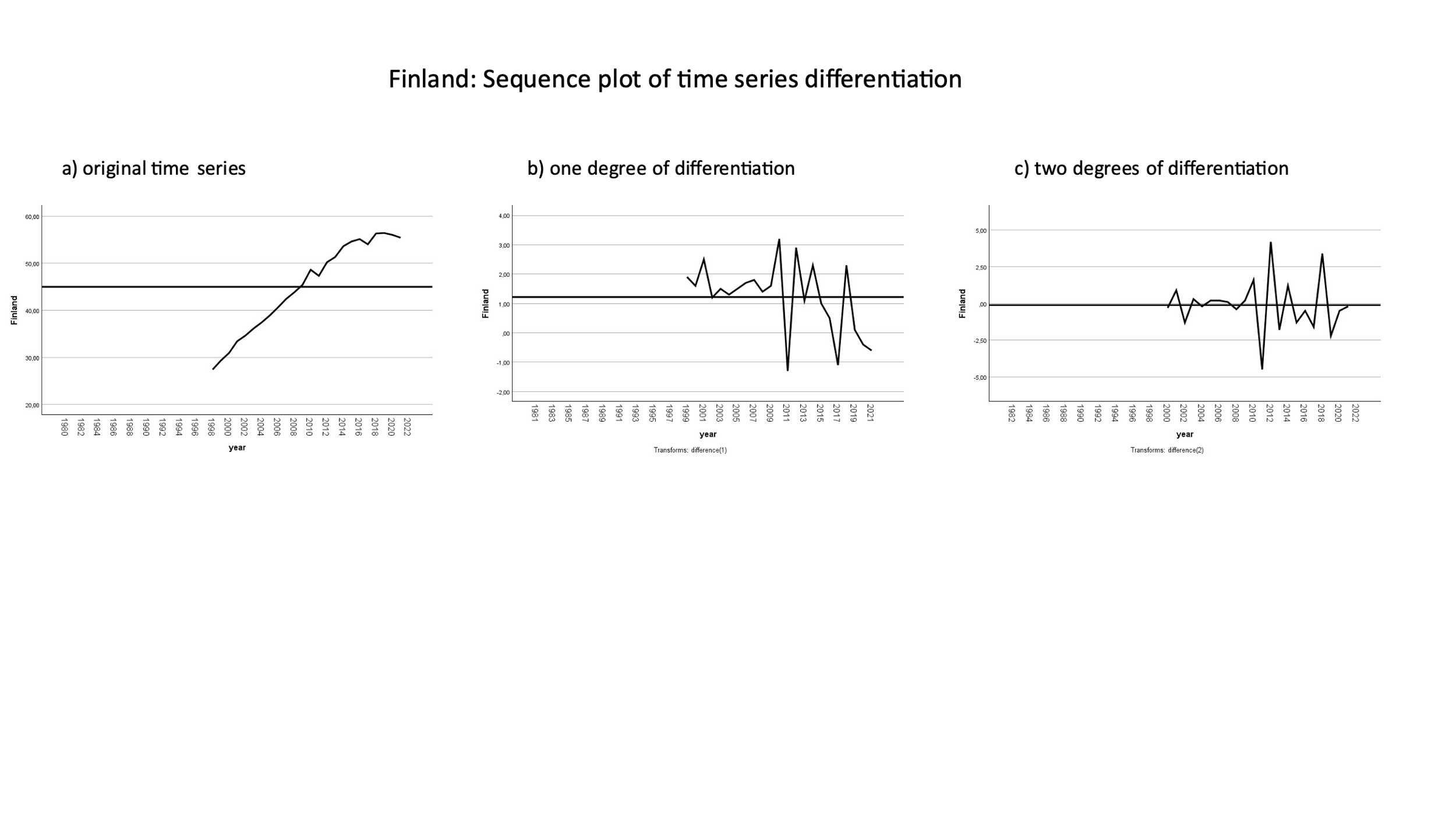


***Fig. S11:*** *Sequence chart of the development of consumption of ATC class H for France. Depicted is the original series (a), the first-degree differentiation (b), and the second-degree differentiation (c). A mean line is overlaid to facilitate the assessment of trends and determine whether the data is stationary or non-stationary. The original time series (a) clearly displays a strong trend, which diminishes progressively with each level of differentiation, ultimately leading to stationarity in (c).*


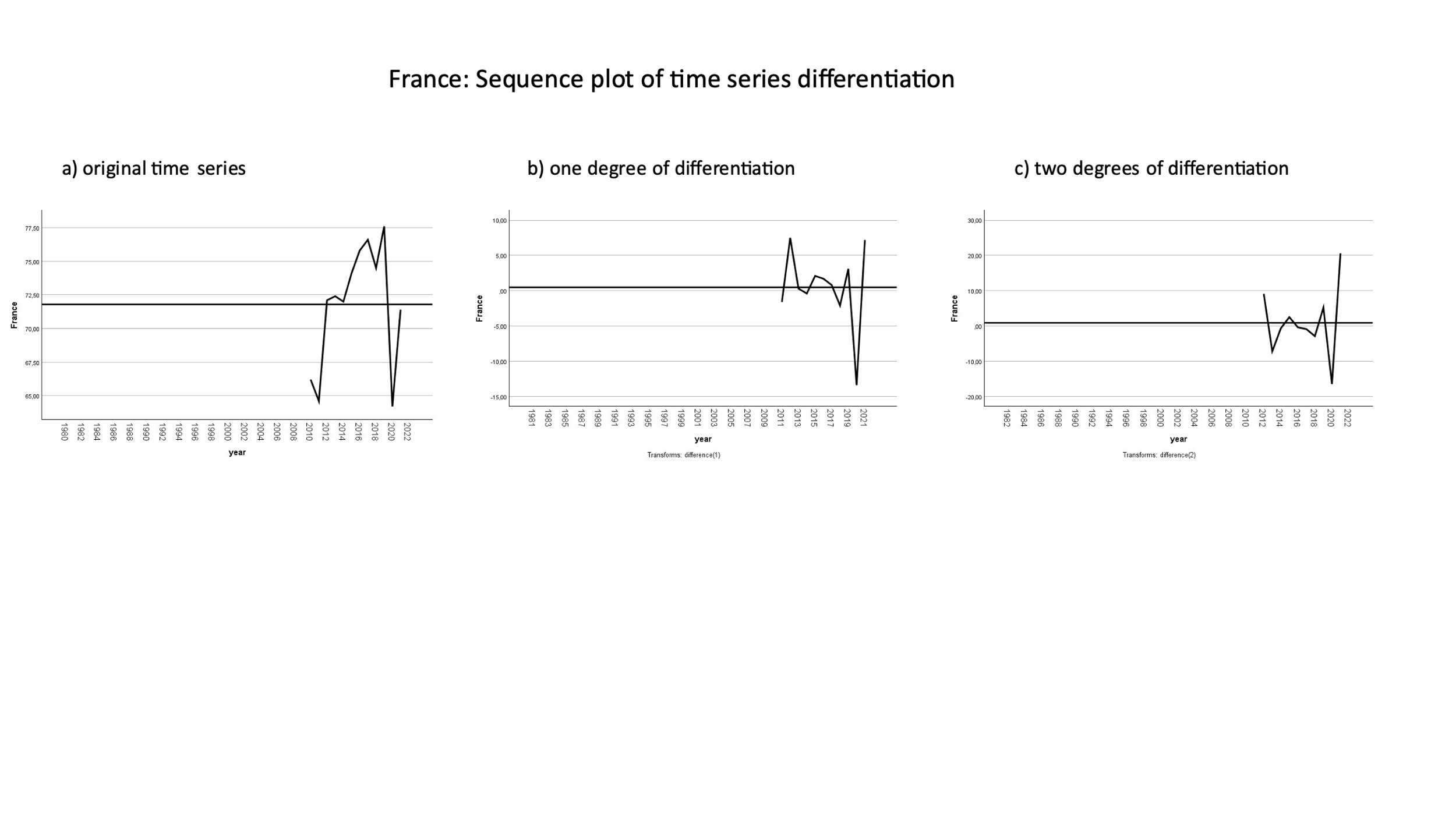


***Fig. S12:*** *Sequence chart of the development of consumption of ATC class H for Germany. Depicted is the original series (a), the first-degree differentiation (b), and the second-degree differentiation (c). A mean line is overlaid to facilitate the assessment of trends and determine whether the data is stationary or non-stationary. The original time series (a) clearly displays a strong trend, which diminishes progressively with each level of differentiation, ultimately leading to stationarity in (c).*


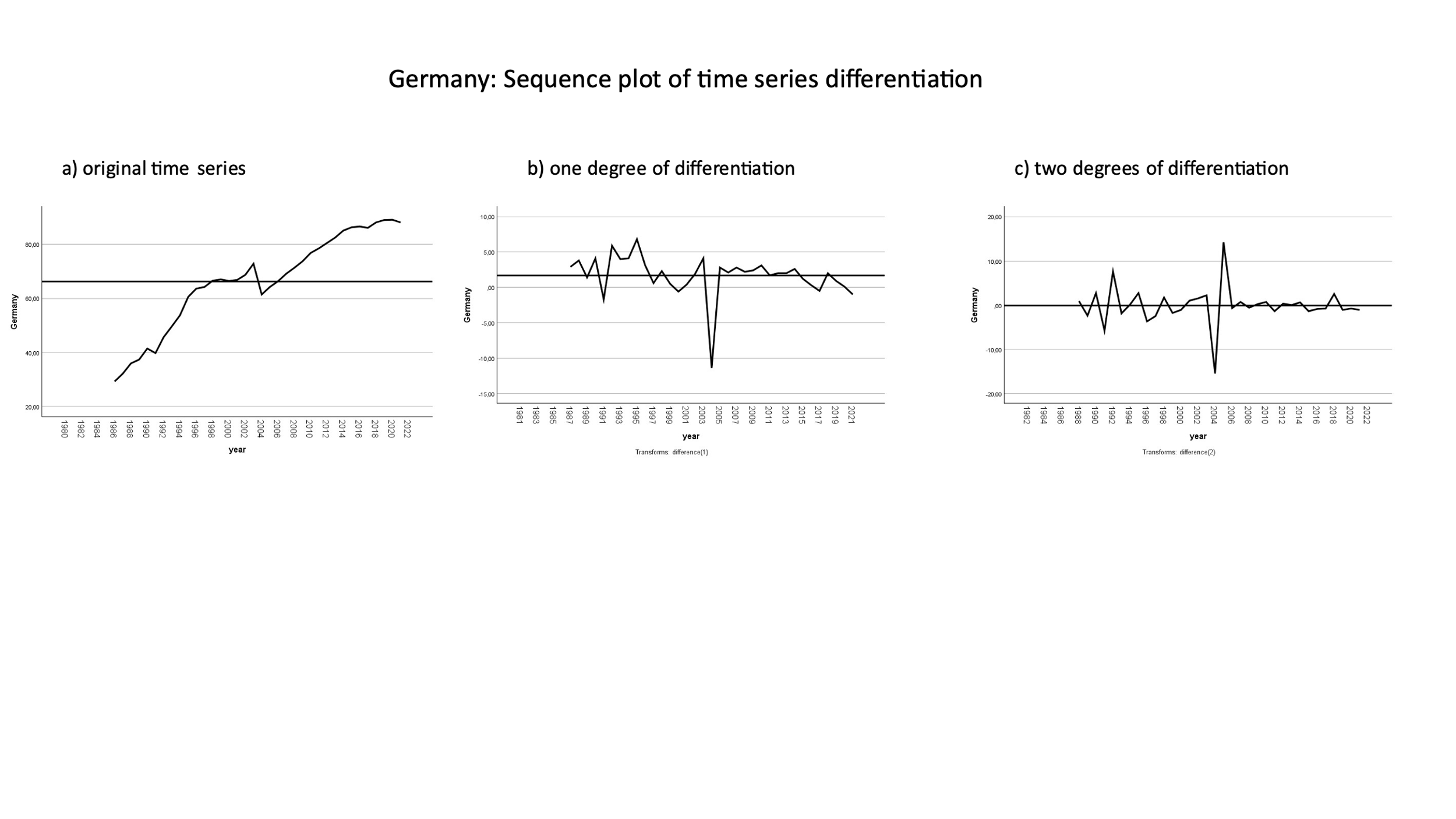


***Fig. S13:*** *Sequence chart of the development of consumption of ATC class H for Greece. Depicted is the original series (a), the first-degree differentiation (b), and the second-degree differentiation (c). A mean line is overlaid to facilitate the assessment of trends and determine whether the data is stationary or non-stationary. The original time series (a) clearly displays a strong trend, which diminishes progressively with each level of differentiation, ultimately leading to stationarity in (c).*


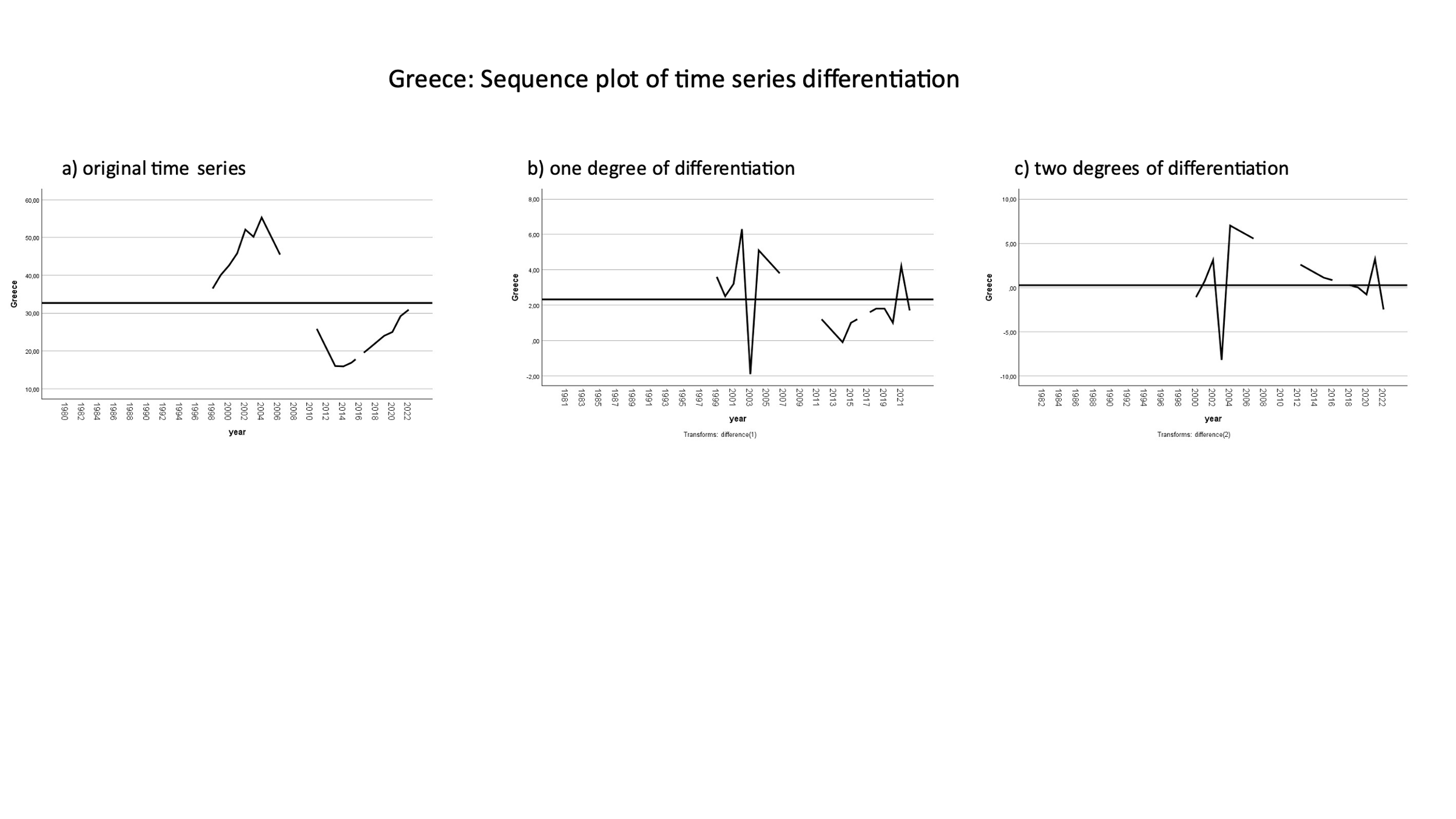


***Fig. S14:*** *Sequence chart of the development of consumption of ATC class H for Australia. Depicted is the original series (a), the first-degree differentiation (b), and the second-degree differentiation (c). A mean line is overlaid to facilitate the assessment of trends and determine whether the data is stationary or non-stationary. The original time series (a) clearly displays a strong trend, which diminishes progressively with each level of differentiation, ultimately leading to stationarity in (c).*


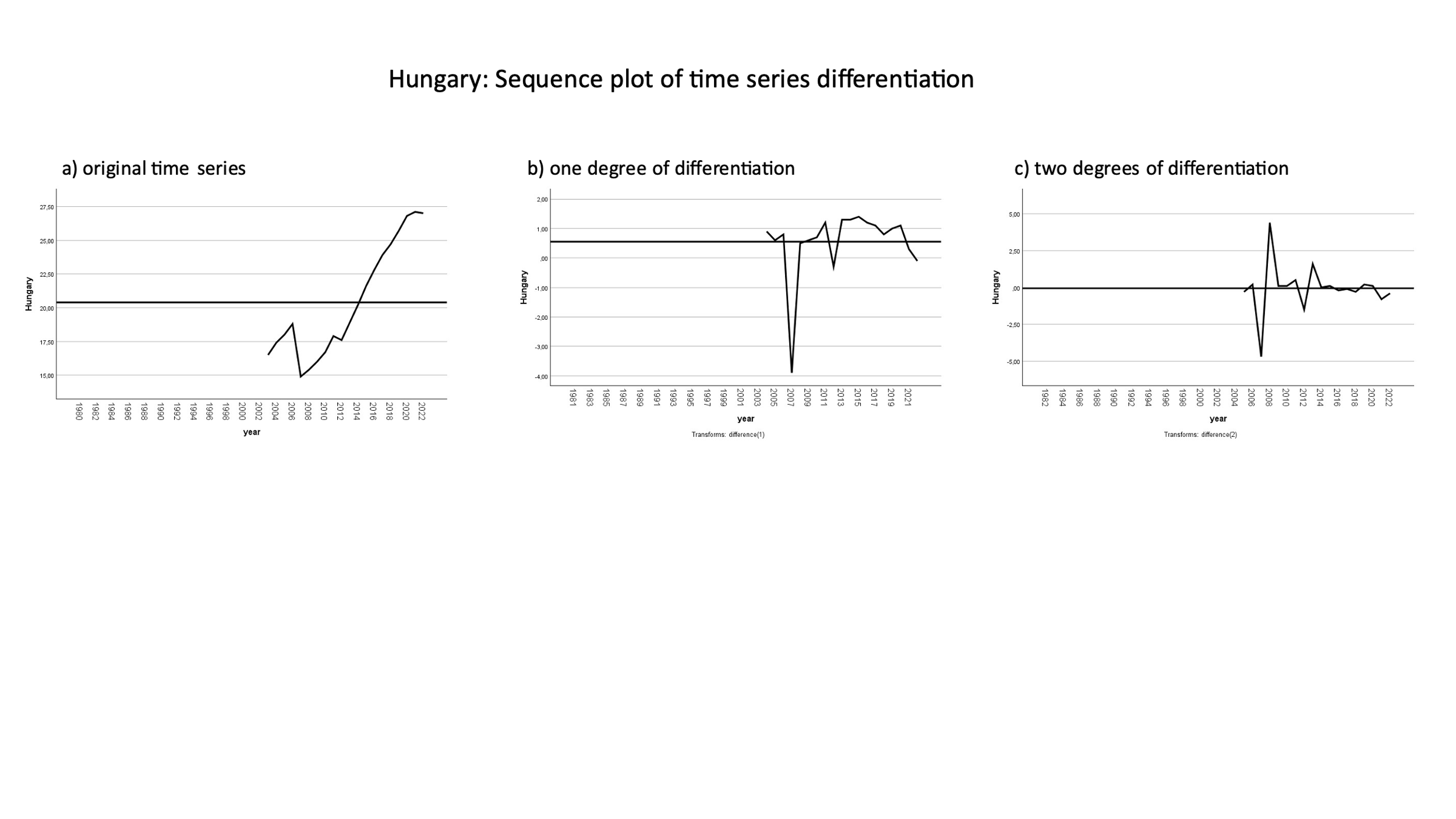


***Fig. S15:*** *Sequence chart of the development of consumption of ATC class H for Australia. Depicted is the original series (a), the first-degree differentiation (b), and the second-degree differentiation (c). A mean line is overlaid to facilitate the assessment of trends and determine whether the data is stationary or non-stationary. The original time series (a) clearly displays a strong trend, which diminishes progressively with each level of differentiation, ultimately leading to stationarity in (c).*


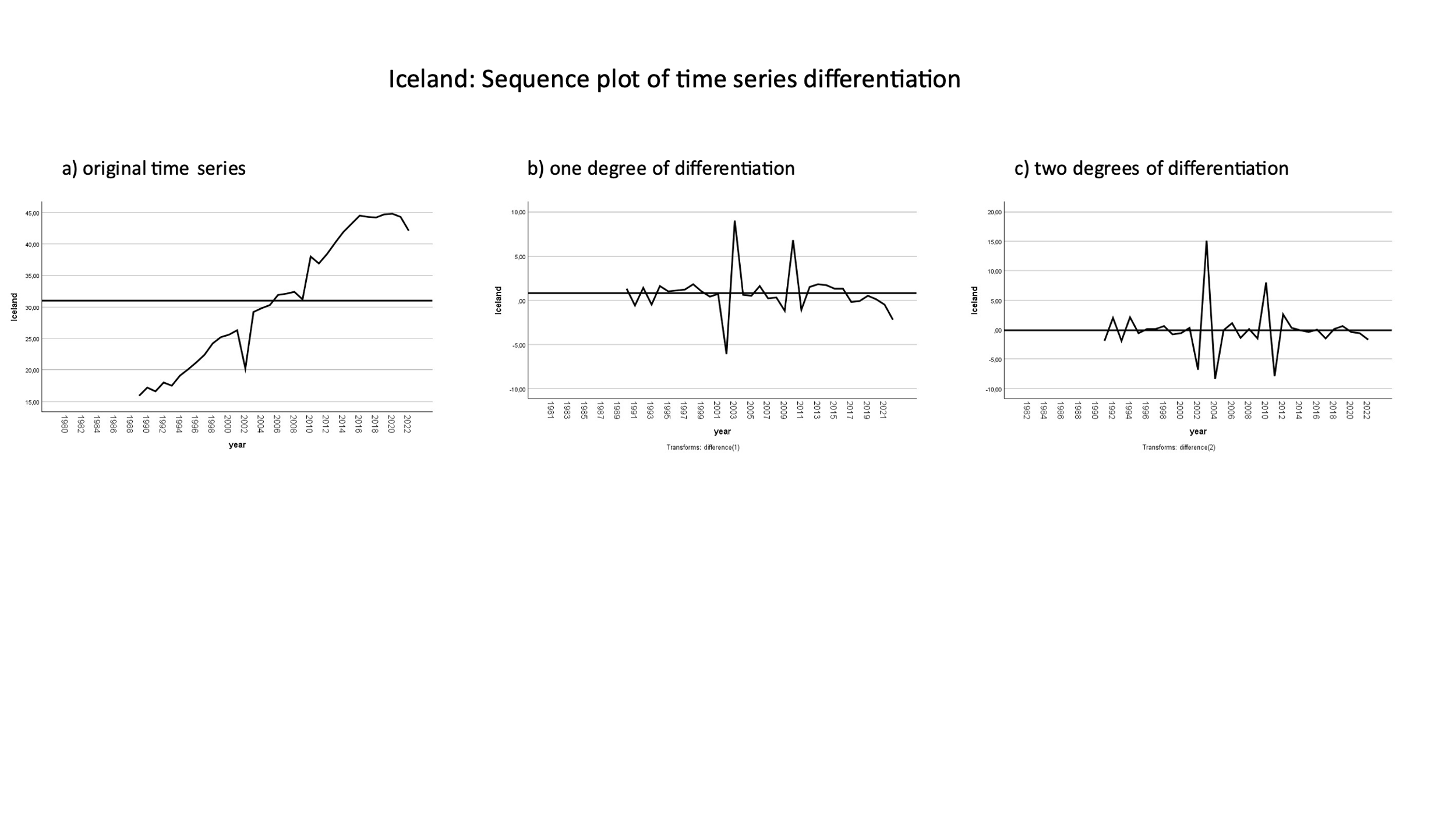


***Fig. S16:*** *Sequence chart of the development of consumption of ATC class H for Israel. Depicted is the original series (a), the first-degree differentiation (b), and the second-degree differentiation (c). A mean line is overlaid to facilitate the assessment of trends and determine whether the data is stationary or non-stationary. The original time series (a) clearly displays a strong trend, which diminishes progressively with each level of differentiation, ultimately leading to stationarity in (c).*


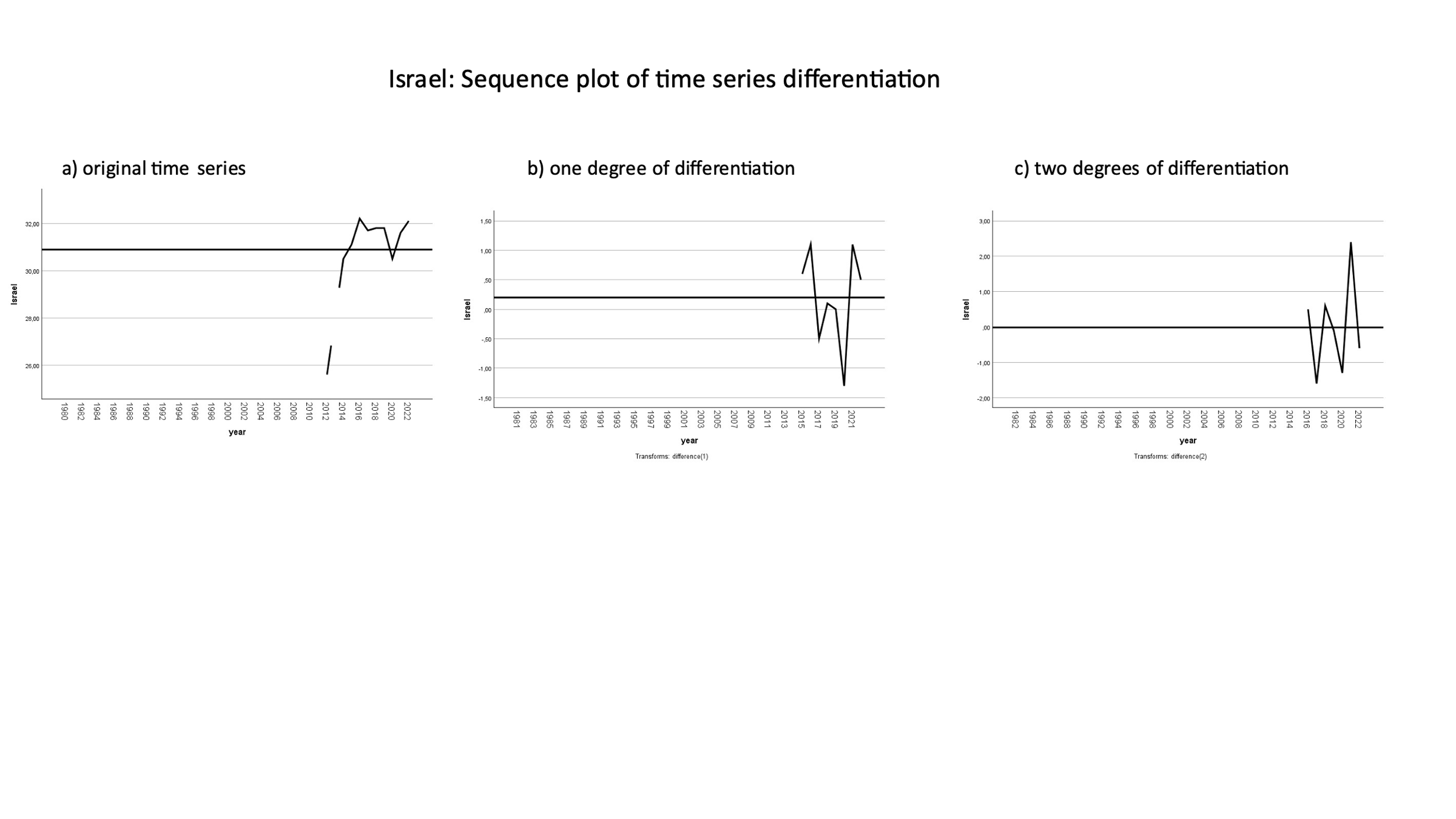


***Fig. S17:*** *Sequence chart of the development of consumption of ATC class H for Italy. Depicted is the original series (a), the first-degree differentiation (b), and the second-degree differentiation (c). A mean line is overlaid to facilitate the assessment of trends and determine whether the data is stationary or non-stationary. The original time series (a) clearly displays a strong trend, which diminishes progressively with each level of differentiation, ultimately leading to stationarity in (c).*


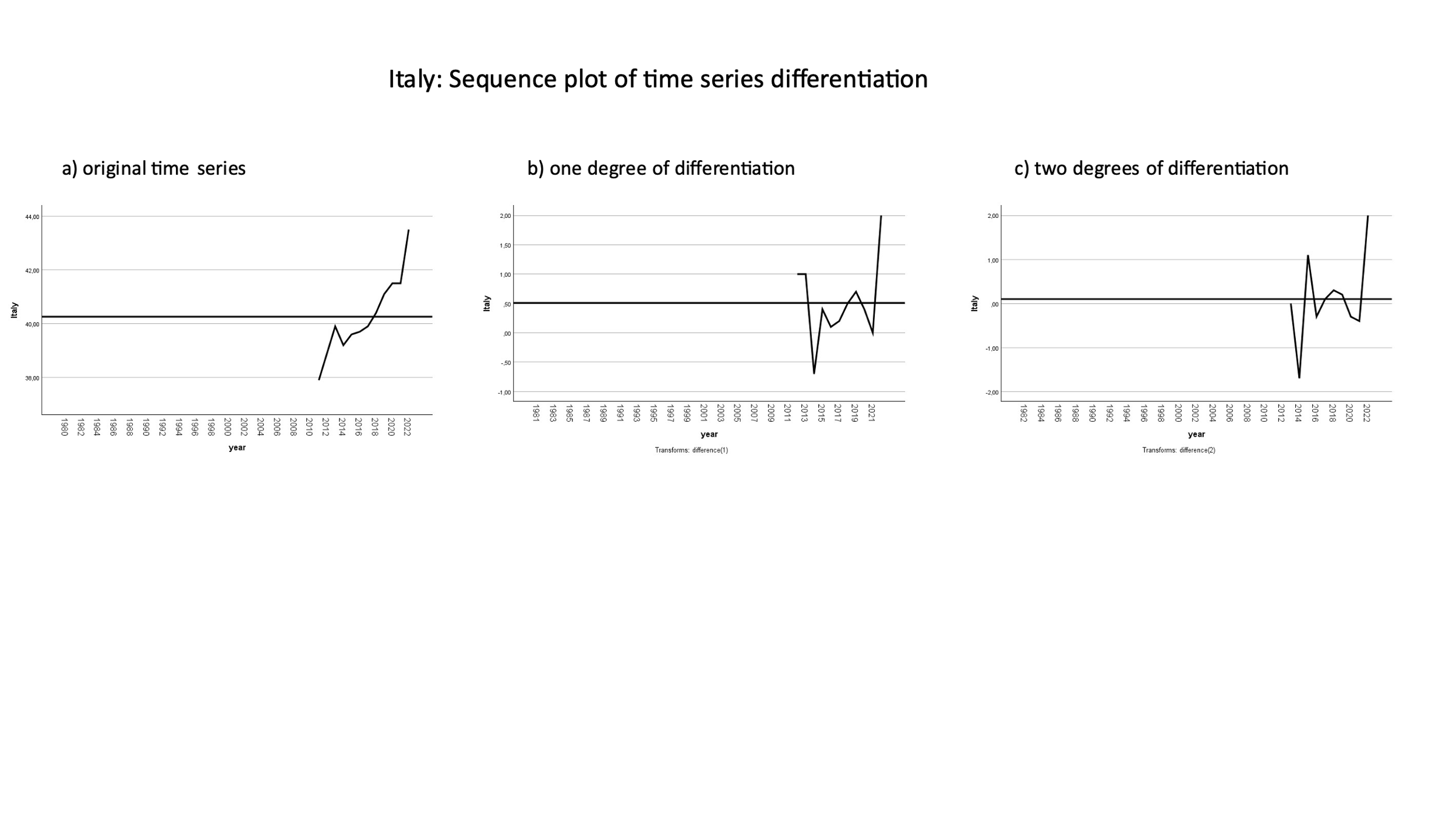


***Fig. S18:*** *Sequence chart of the development of consumption of ATC class H for Korea. Depicted is the original series (a), the first-degree differentiation (b), and the second-degree differentiation (c). A mean line is overlaid to facilitate the assessment of trends and determine whether the data is stationary or non-stationary. The original time series (a) clearly displays a strong trend, which diminishes progressively with each level of differentiation, ultimately leading to stationarity in (c).*

*
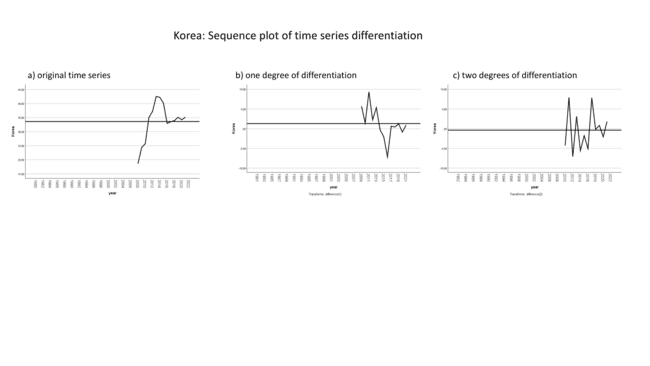
*

***Fig. S19:*** *Sequence chart of the development of consumption of ATC class H for Latvia. Depicted is the original series (a), the first-degree differentiation (b), and the second-degree differentiation (c). A mean line is overlaid to facilitate the assessment of trends and determine whether the data is stationary or non-stationary. The original time series (a) clearly displays a strong trend, which diminishes progressively with each level of differentiation, ultimately leading to stationarity in (c).*


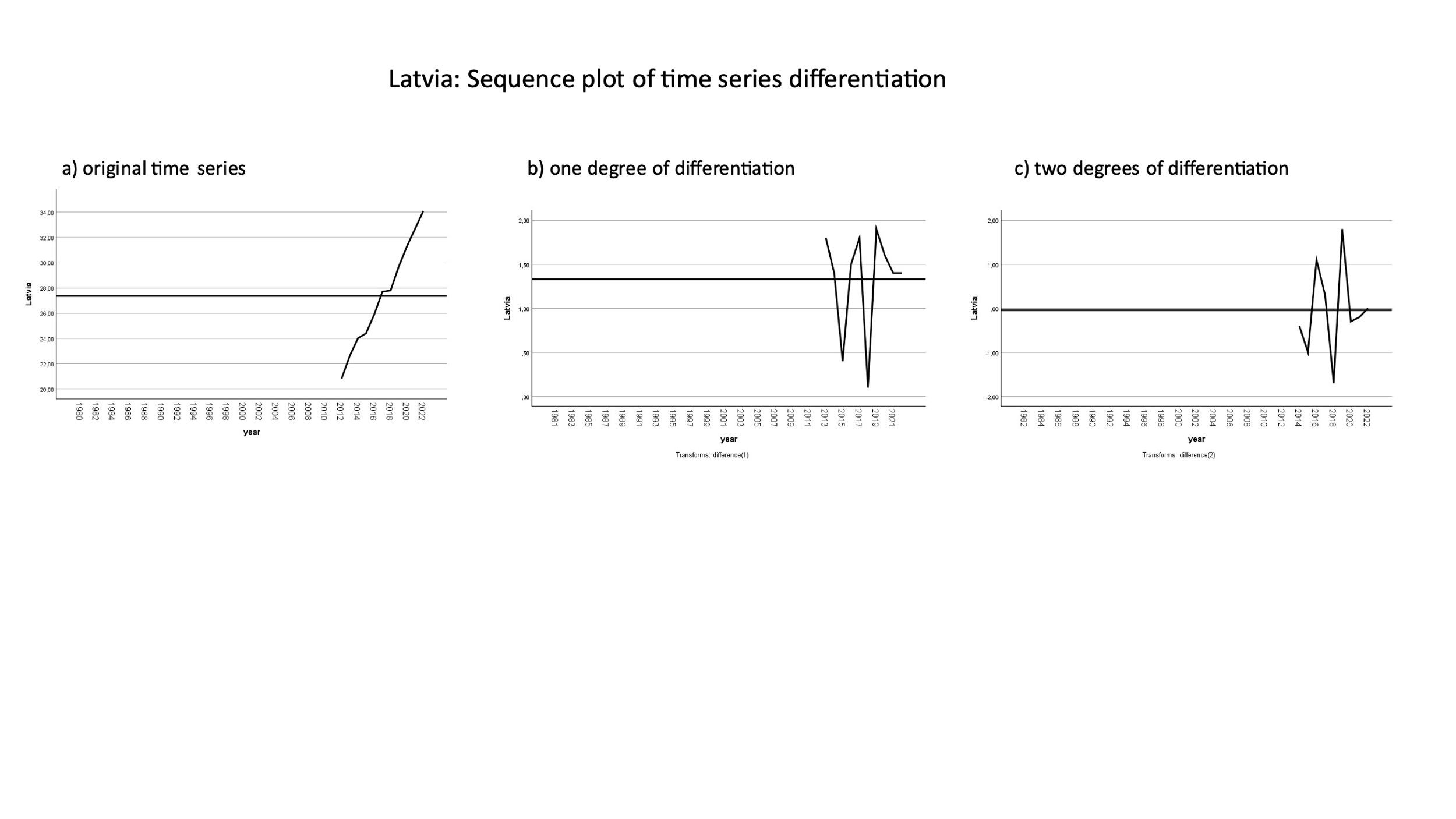


***Fig. S20:*** *Sequence chart of the development of consumption of ATC class H for Lithuania. Depicted is the original series (a), the first-degree differentiation (b), and the second-degree differentiation (c). A mean line is overlaid to facilitate the assessment of trends and determine whether the data is stationary or non-stationary. The original time series (a) clearly displays a strong trend, which diminishes progressively with each level of differentiation, ultimately leading to stationarity in (c).*


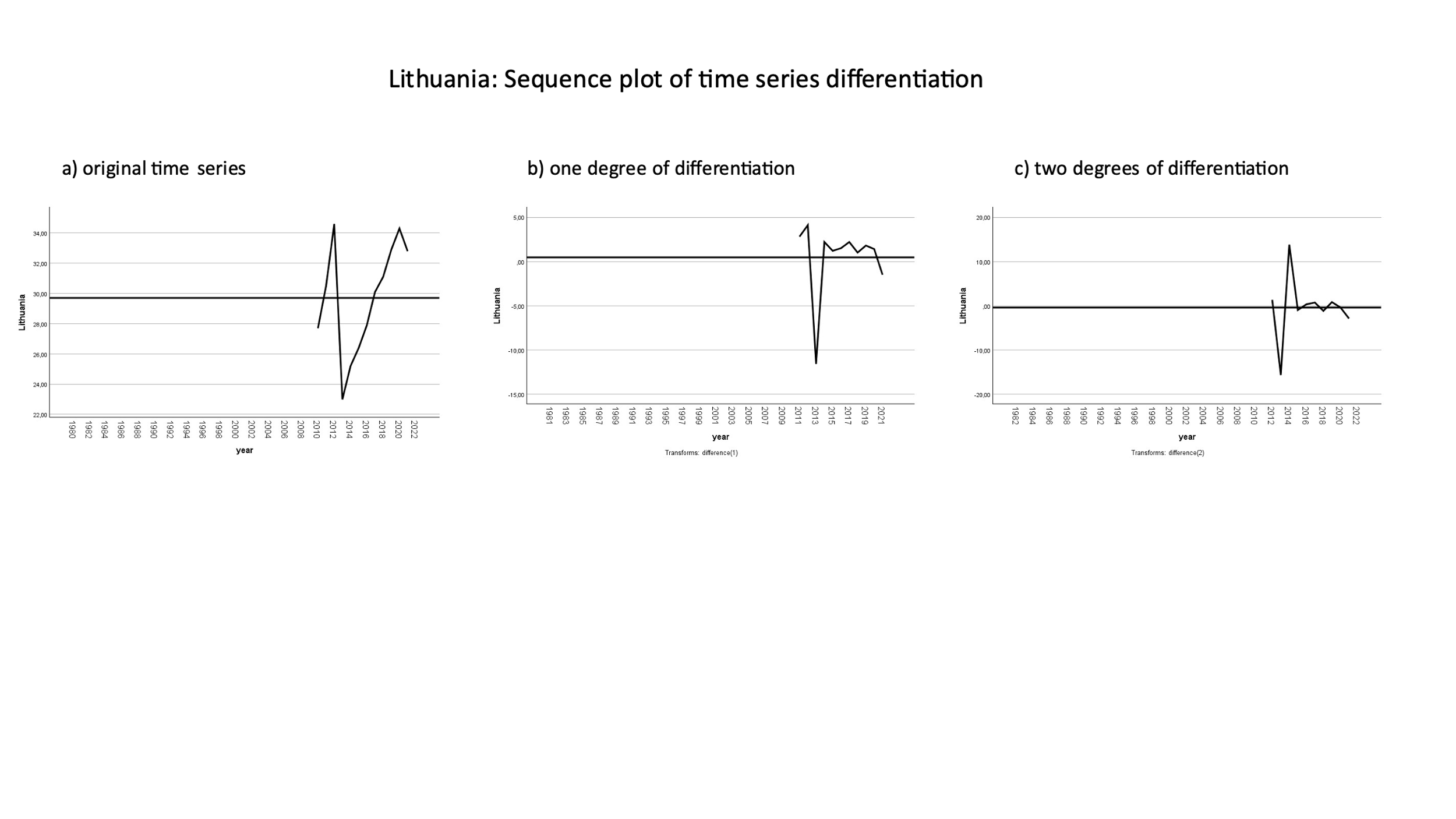


***Fig. S21:*** *Sequence chart of the development of consumption of ATC class H for Luxembourg. Depicted is the original series (a), the first-degree differentiation (b), and the second-degree differentiation (c). A mean line is overlaid to facilitate the assessment of trends and determine whether the data is stationary or non-stationary. The original time series (a) clearly displays a strong trend, which diminishes progressively with each level of differentiation, ultimately leading to stationarity in (c).*


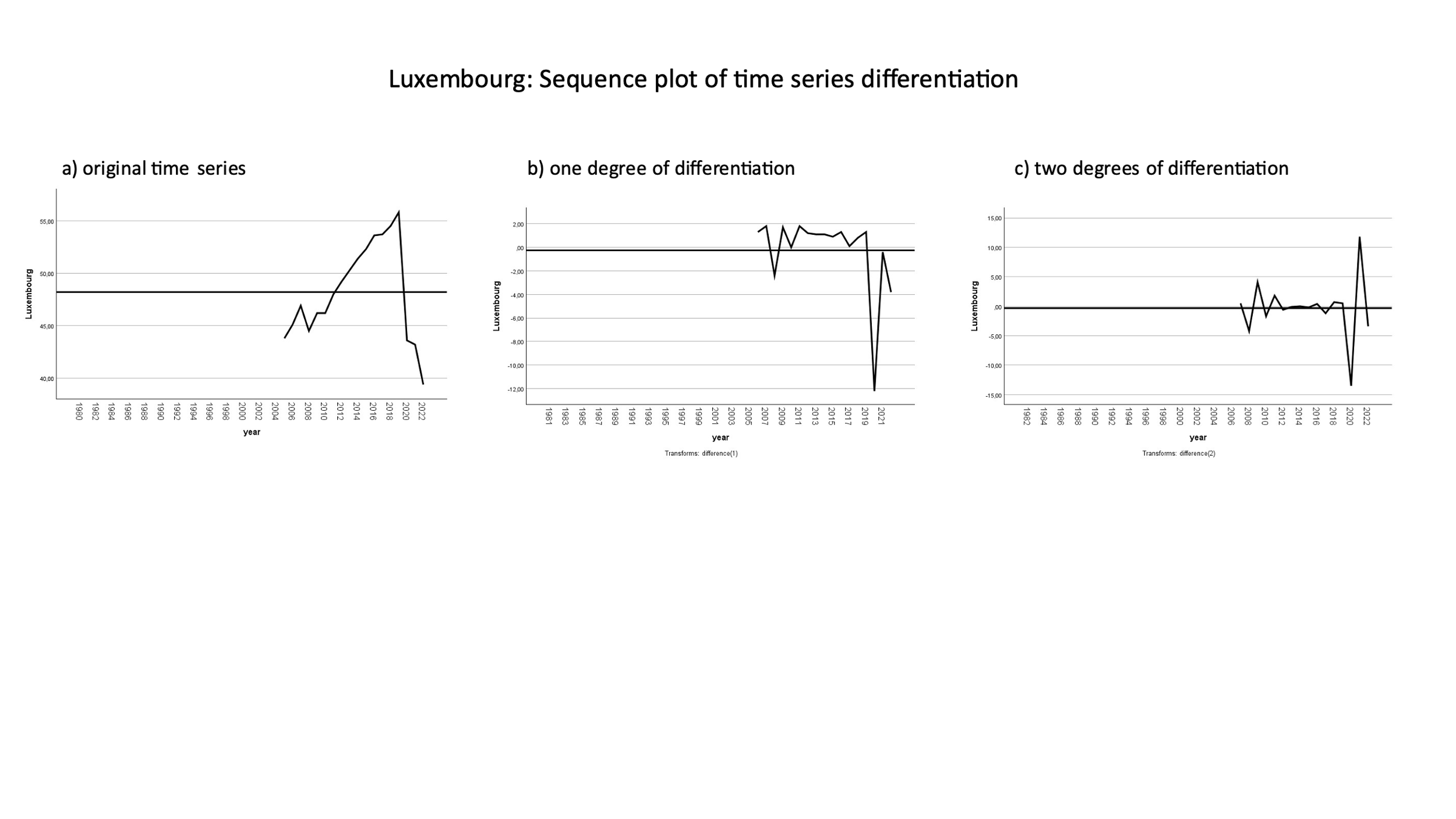


***Fig. S22:*** *Sequence chart of the development of consumption of ATC class H for Netherlands. Depicted is the original series (a), the first-degree differentiation (b), and the second-degree differentiation (c). A mean line is overlaid to facilitate the assessment of trends and determine whether the data is stationary or non-stationary. The original time series (a) clearly displays a strong trend, which diminishes progressively with each level of differentiation, ultimately leading to stationarity in (c).*


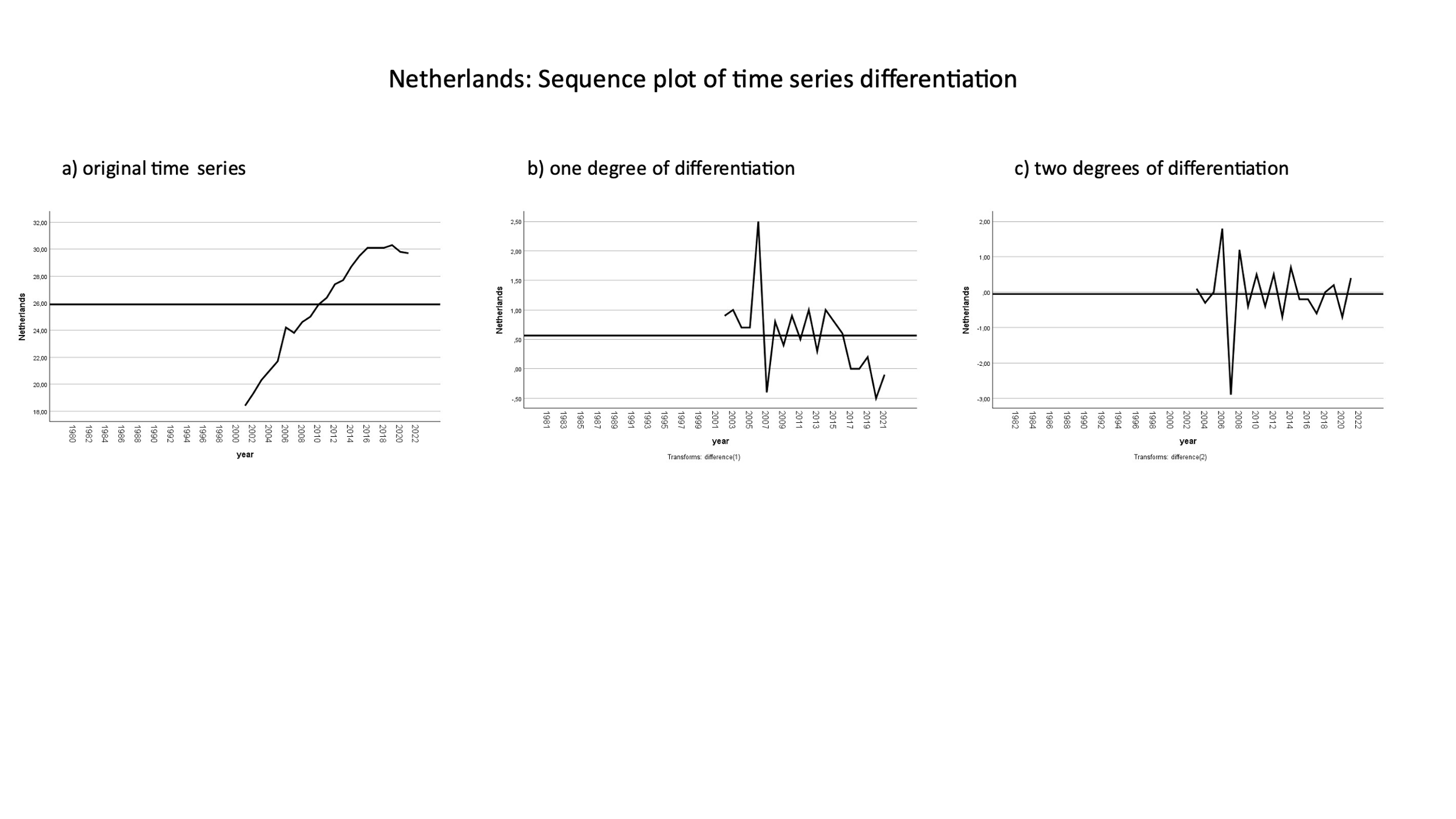


***Fig. S23:*** *Sequence chart of the development of consumption of ATC class H for Norway. Depicted is the original series (a), the first-degree differentiation (b), and the second-degree differentiation (c). A mean line is overlaid to facilitate the assessment of trends and determine whether the data is stationary or non-stationary. The original time series (a) clearly displays a strong trend, which diminishes progressively with each level of differentiation, ultimately leading to stationarity in (c).*


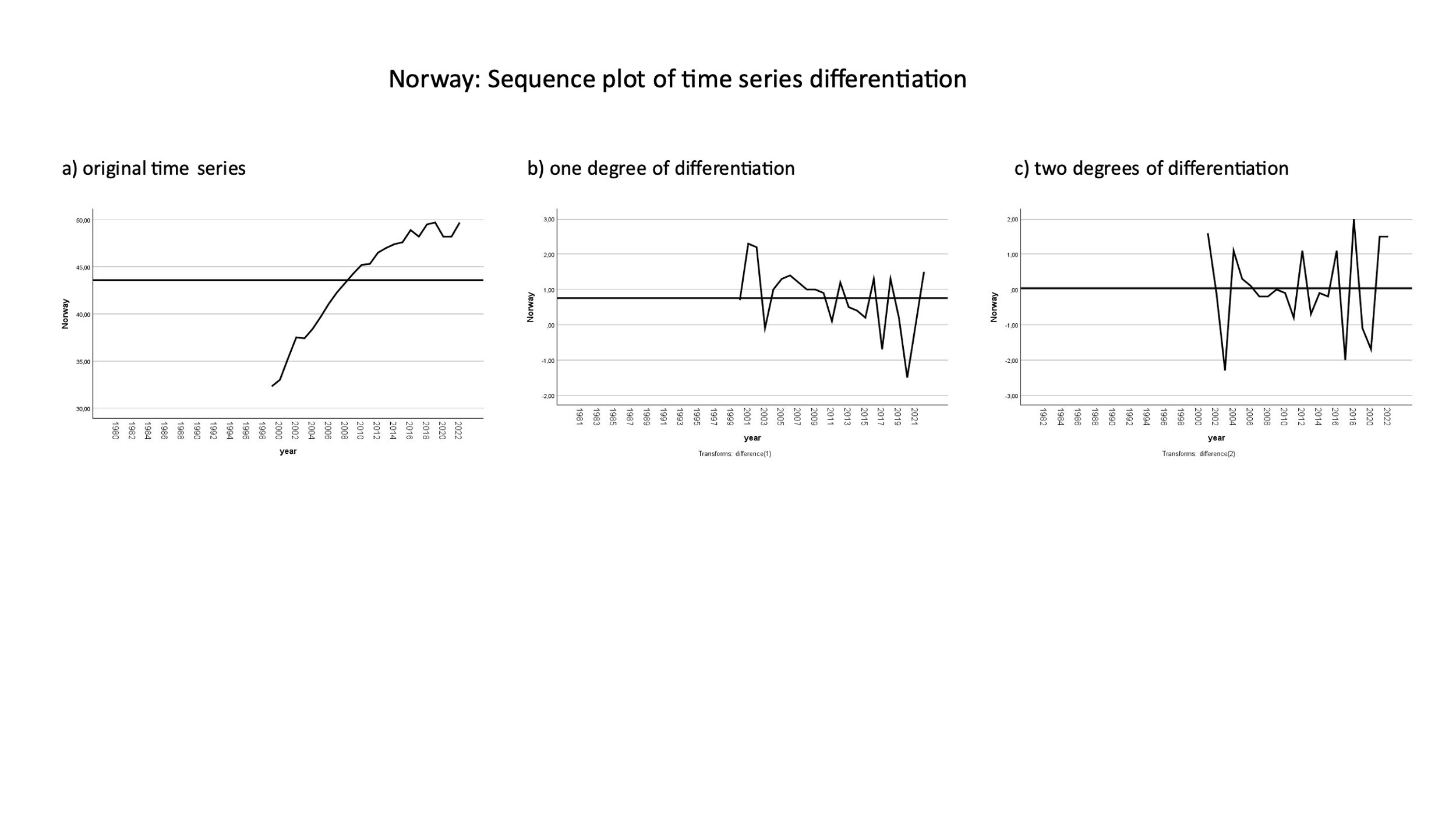


***Fig. S24:*** *Sequence chart of the development of consumption of ATC class H for Portugal. Depicted is the original series (a), the first-degree differentiation (b), and the second-degree differentiation (c). A mean line is overlaid to facilitate the assessment of trends and determine whether the data is stationary or non-stationary. The original time series (a) clearly displays a strong trend, which diminishes progressively with each level of differentiation, ultimately leading to stationarity in (c).*


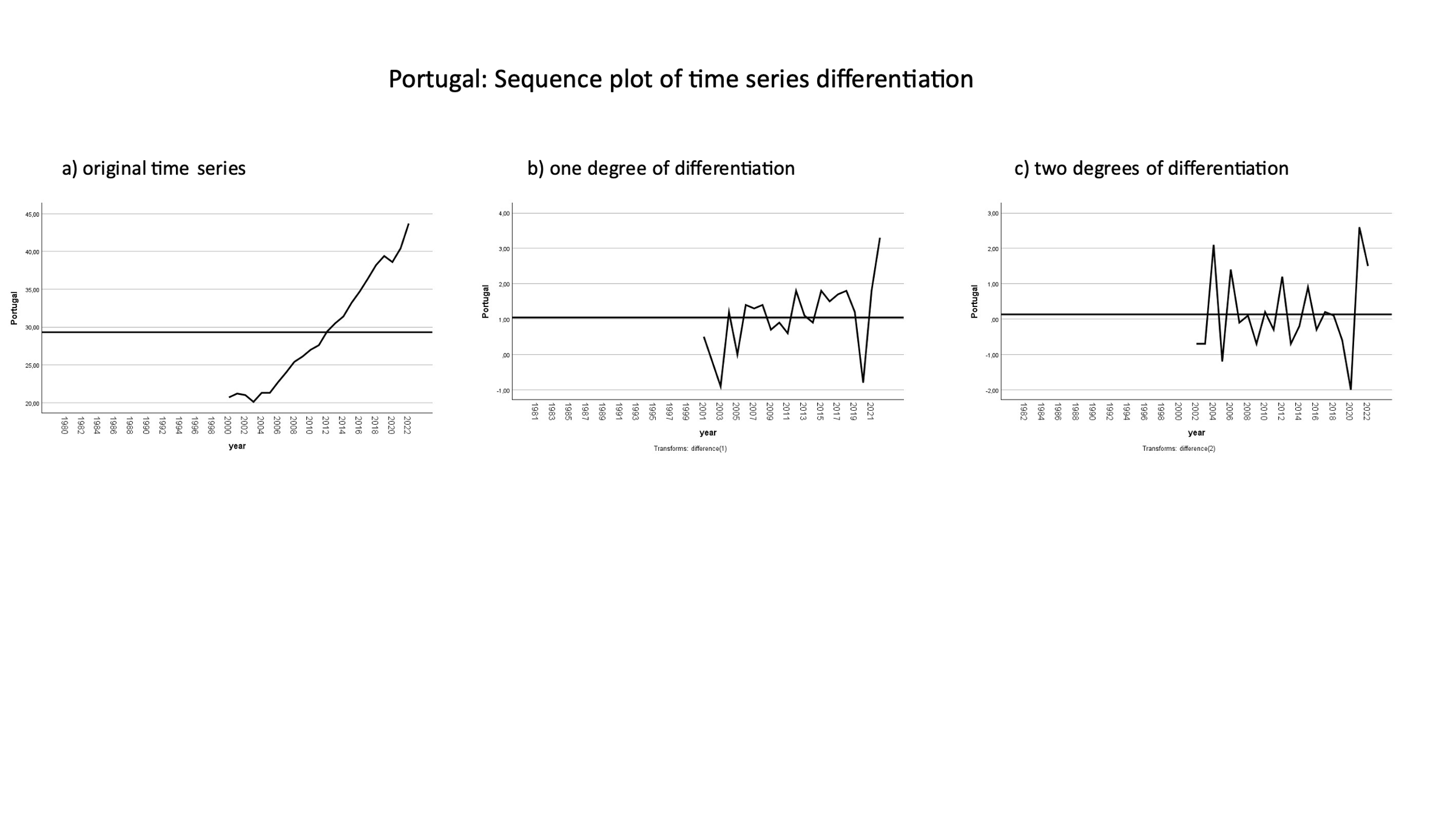


***Fig. S25:*** *Sequence chart of the development of consumption of ATC class H for Slovakia. Depicted is the original series (a), the first-degree differentiation (b), and the second-degree differentiation (c). A mean line is overlaid to facilitate the assessment of trends and determine whether the data is stationary or non-stationary. The original time series (a) clearly displays a strong trend, which diminishes progressively with each level of differentiation, ultimately leading to stationarity in (c).*


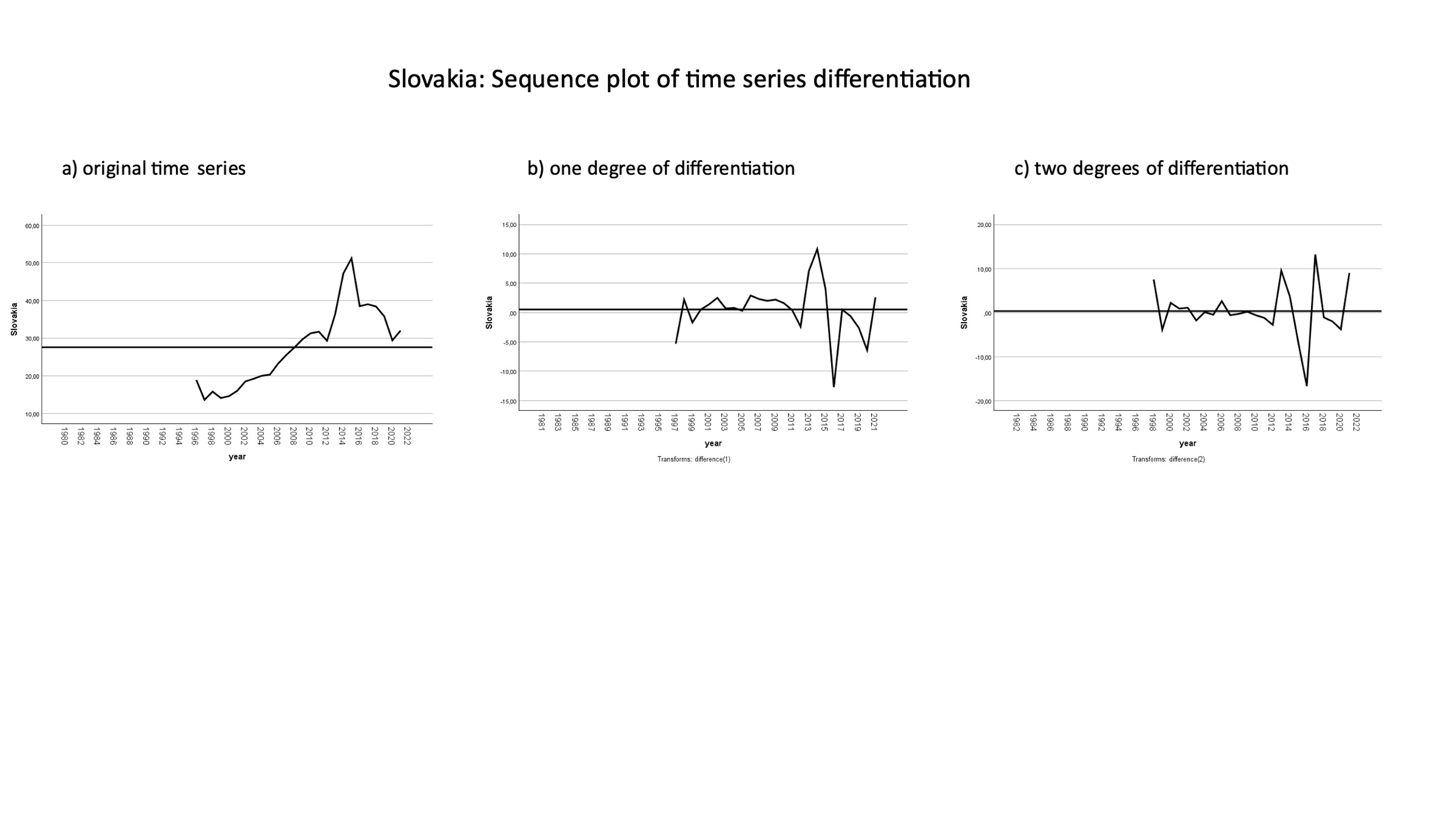


***Fig. S26:*** *Sequence chart of the development of consumption of ATC class H for Slovenia. Depicted is the original series (a), the first-degree differentiation (b), and the second-degree differentiation (c). A mean line is overlaid to facilitate the assessment of trends and determine whether the data is stationary or non-stationary. The original time series (a) clearly displays a strong trend, which diminishes progressively with each level of differentiation, ultimately leading to stationarity in (c).*


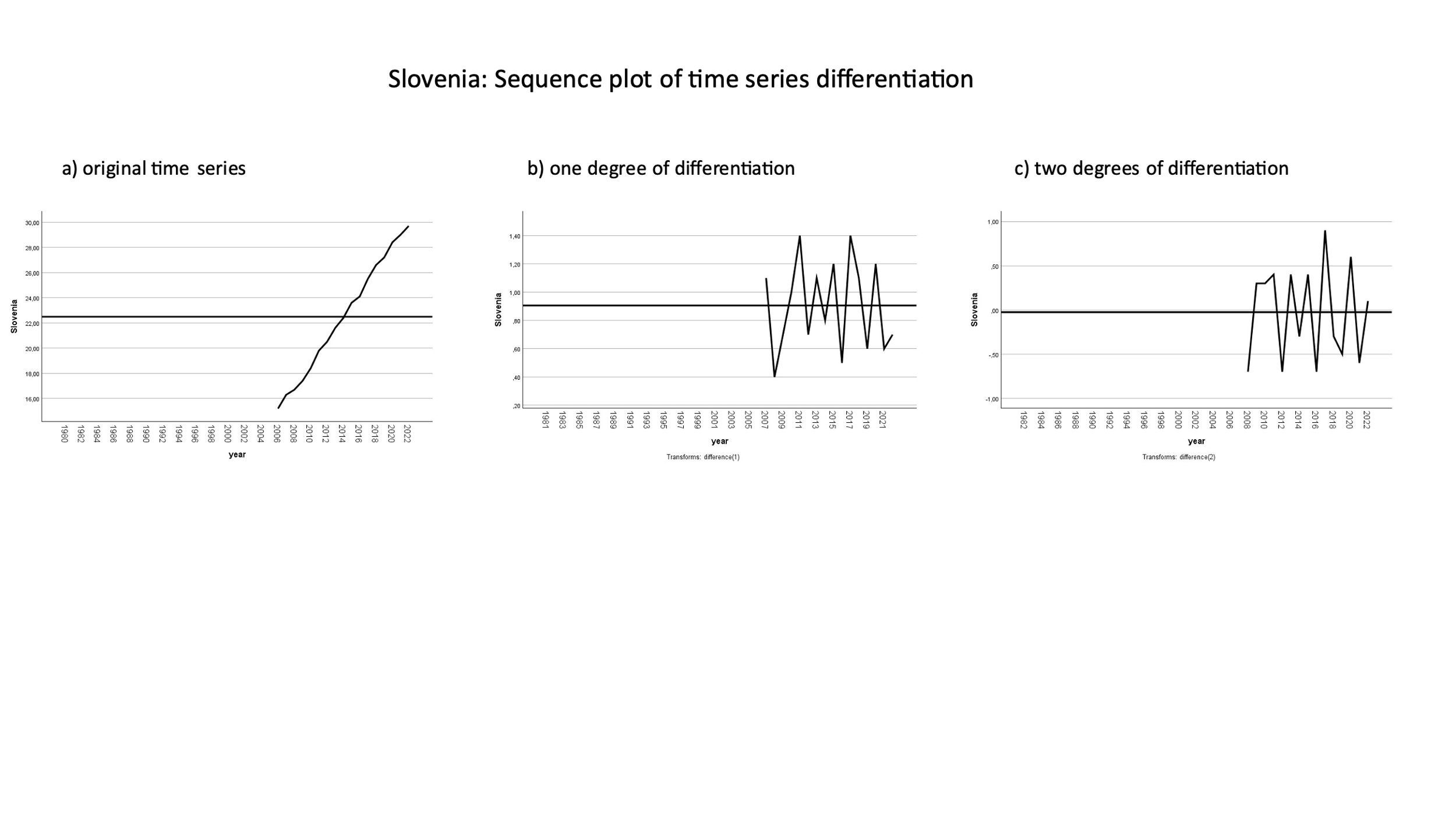


***Fig. S27:*** *Sequence chart of the development of consumption of ATC class H for Spain. Depicted is the original series (a), the first-degree differentiation (b), and the second-degree differentiation (c). A mean line is overlaid to facilitate the assessment of trends and determine whether the data is stationary or non-stationary. The original time series (a) clearly displays a strong trend, which diminishes progressively with each level of differentiation, ultimately leading to stationarity in (c).*


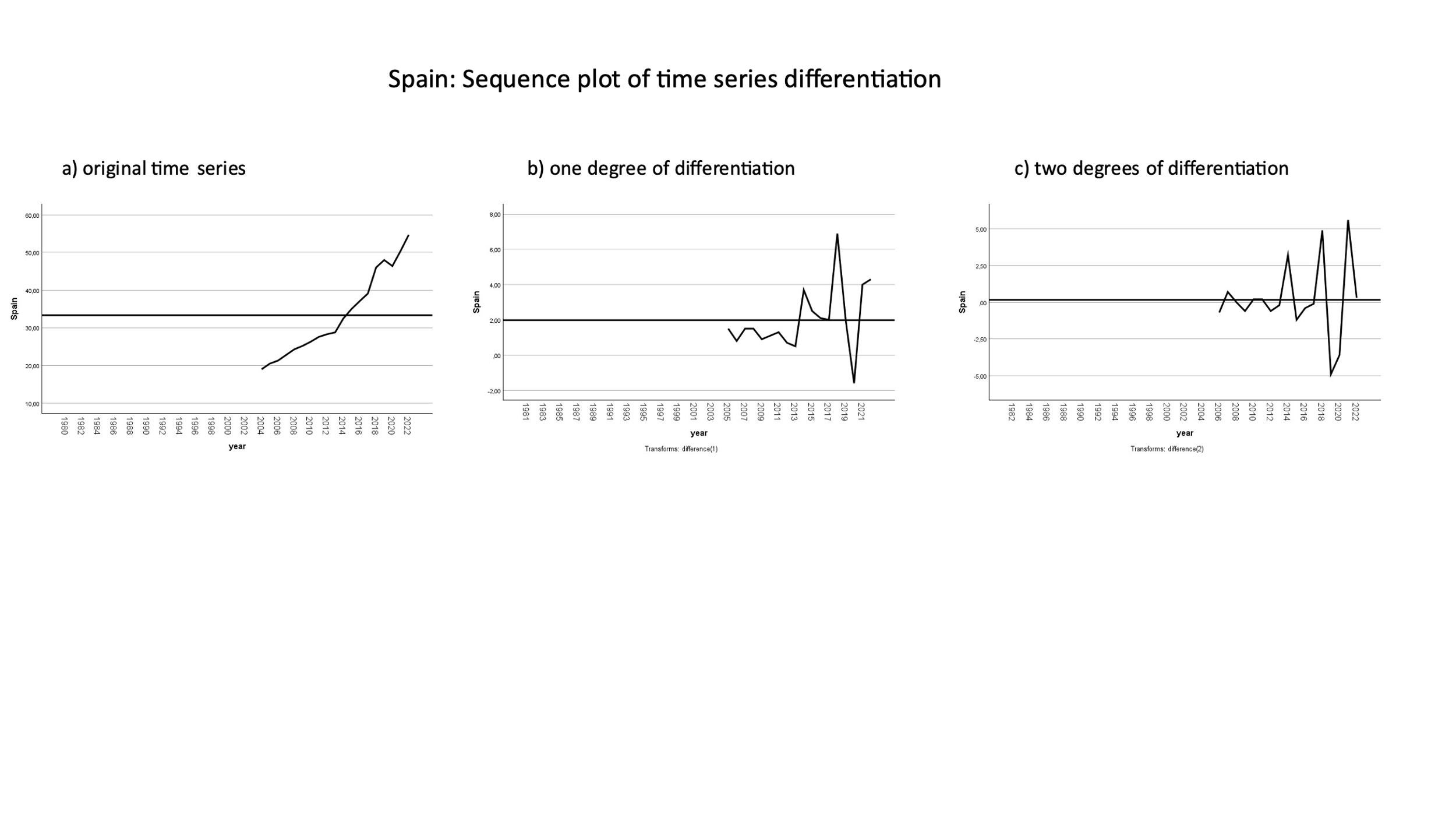


***Fig. S28:*** *Sequence chart of the development of consumption of ATC class H for Sweden. Depicted is the original series (a), the first-degree differentiation (b), and the second-degree differentiation (c). A mean line is overlaid to facilitate the assessment of trends and determine whether the data is stationary or non-stationary. The original time series (a) clearly displays a strong trend, which diminishes progressively with each level of differentiation, ultimately leading to stationarity in (c).*


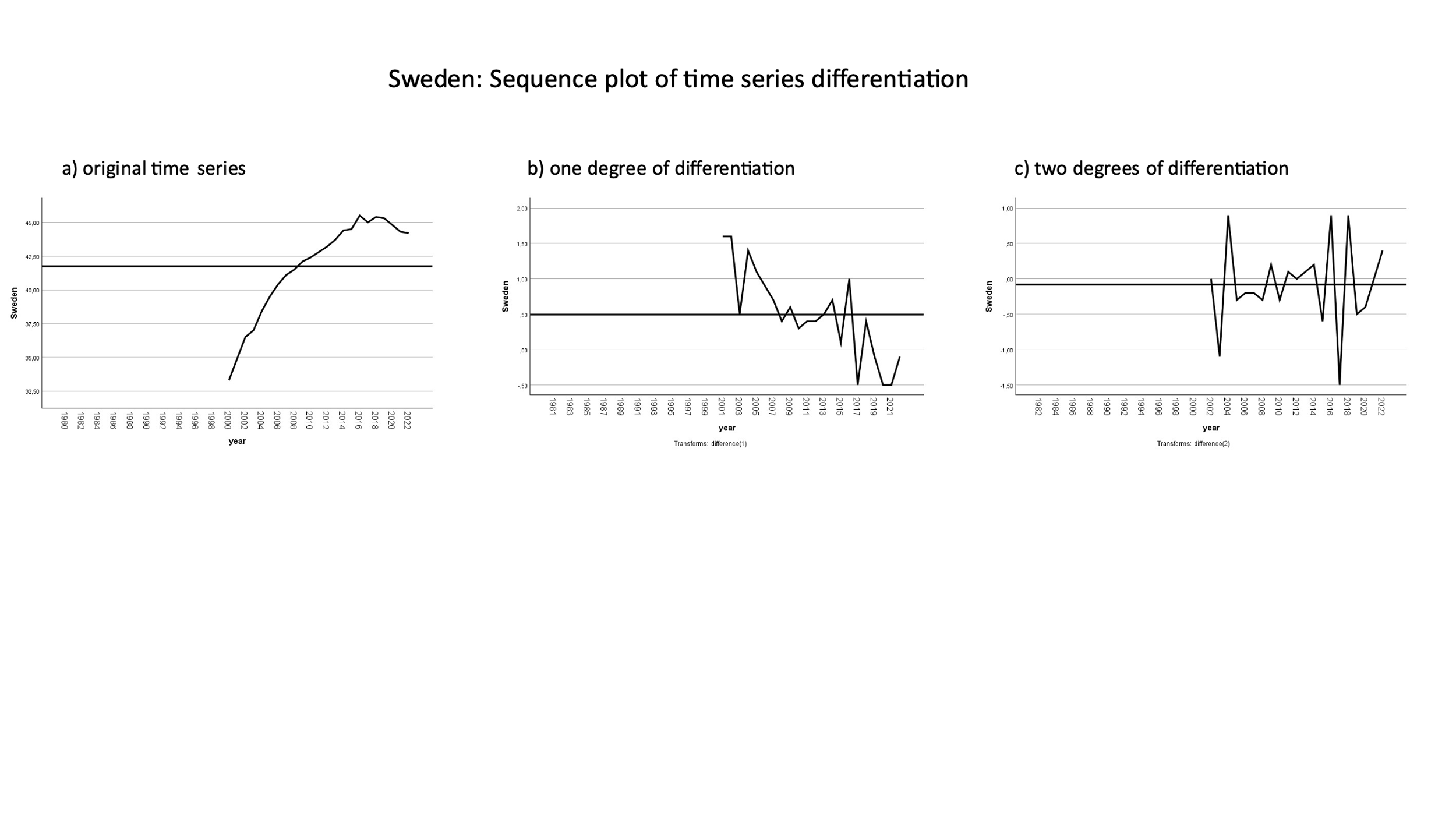


***Fig. S29:*** *Sequence chart of the development of consumption of ATC class H for Turkey. Depicted is the original series (a), the first-degree differentiation (b), and the second-degree differentiation (c). A mean line is overlaid to facilitate the assessment of trends and determine whether the data is stationary or non-stationary. The original time series (a) clearly displays a strong trend, which diminishes progressively with each level of differentiation, ultimately leading to stationarity in (c).*


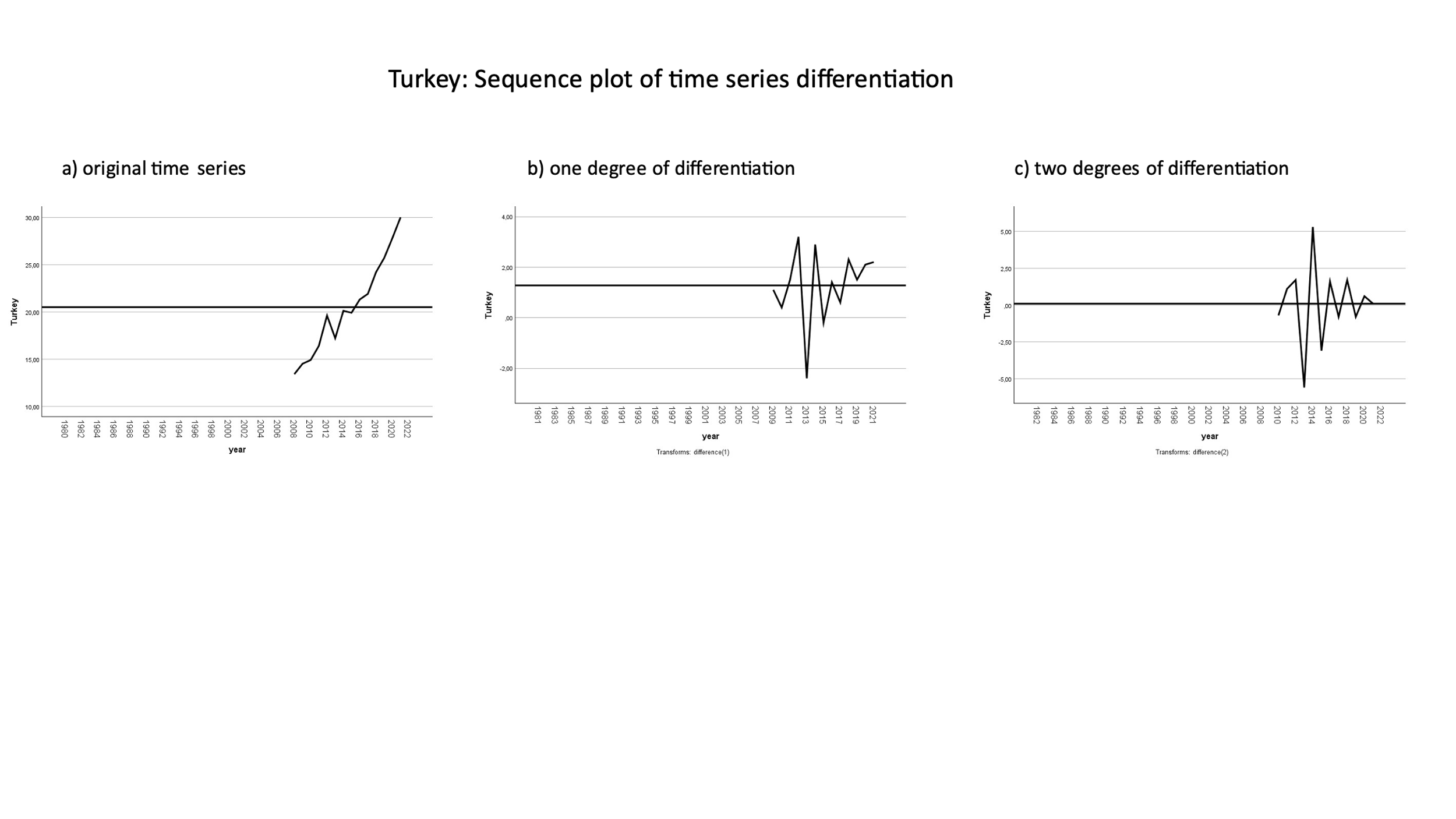


***Fig. S30:*** *Sequence chart of the development of consumption of ATC class H for the United Kingdom. Depicted is the original series (a), the first-degree differentiation (b), and the second-degree differentiation (c). A mean line is overlaid to facilitate the assessment of trends and determine whether the data is stationary or non-stationary. The original time series (a) clearly displays a strong trend, which diminishes progressively with each level of differentiation, ultimately leading to stationarity in (c).*


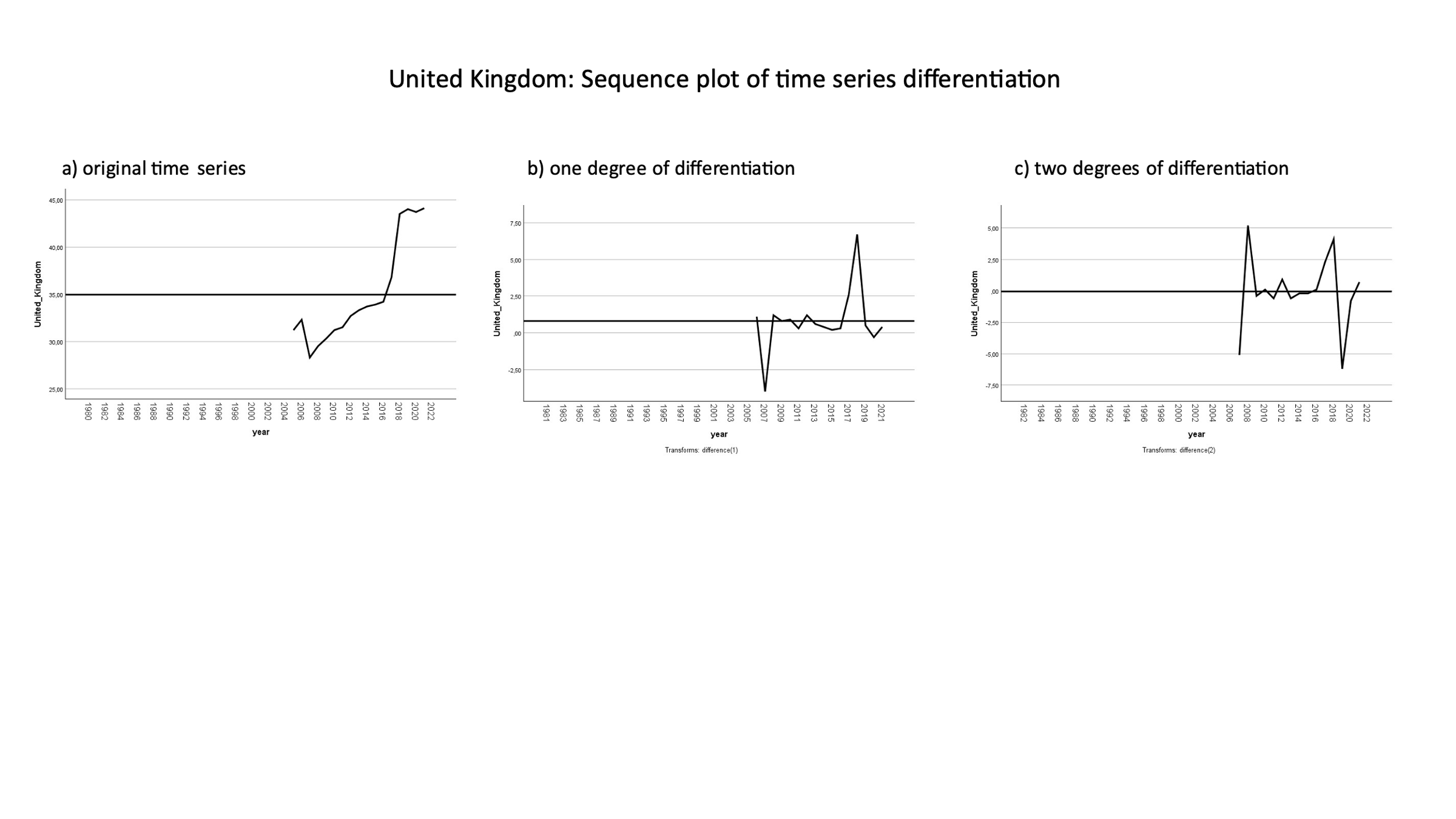


***Fig. S31:*** *Sequence chart of the development of consumption of ATC class H for the United Kingdom. Depicted is the original series (a), the first-degree differentiation (b), and the second-degree differentiation (c). A mean line is overlaid to facilitate the assessment of trends and determine whether the data is stationary or non-stationary. The original time series (a) clearly displays a strong trend, which diminishes progressively with each level of differentiation, ultimately leading to stationarity in (c).*


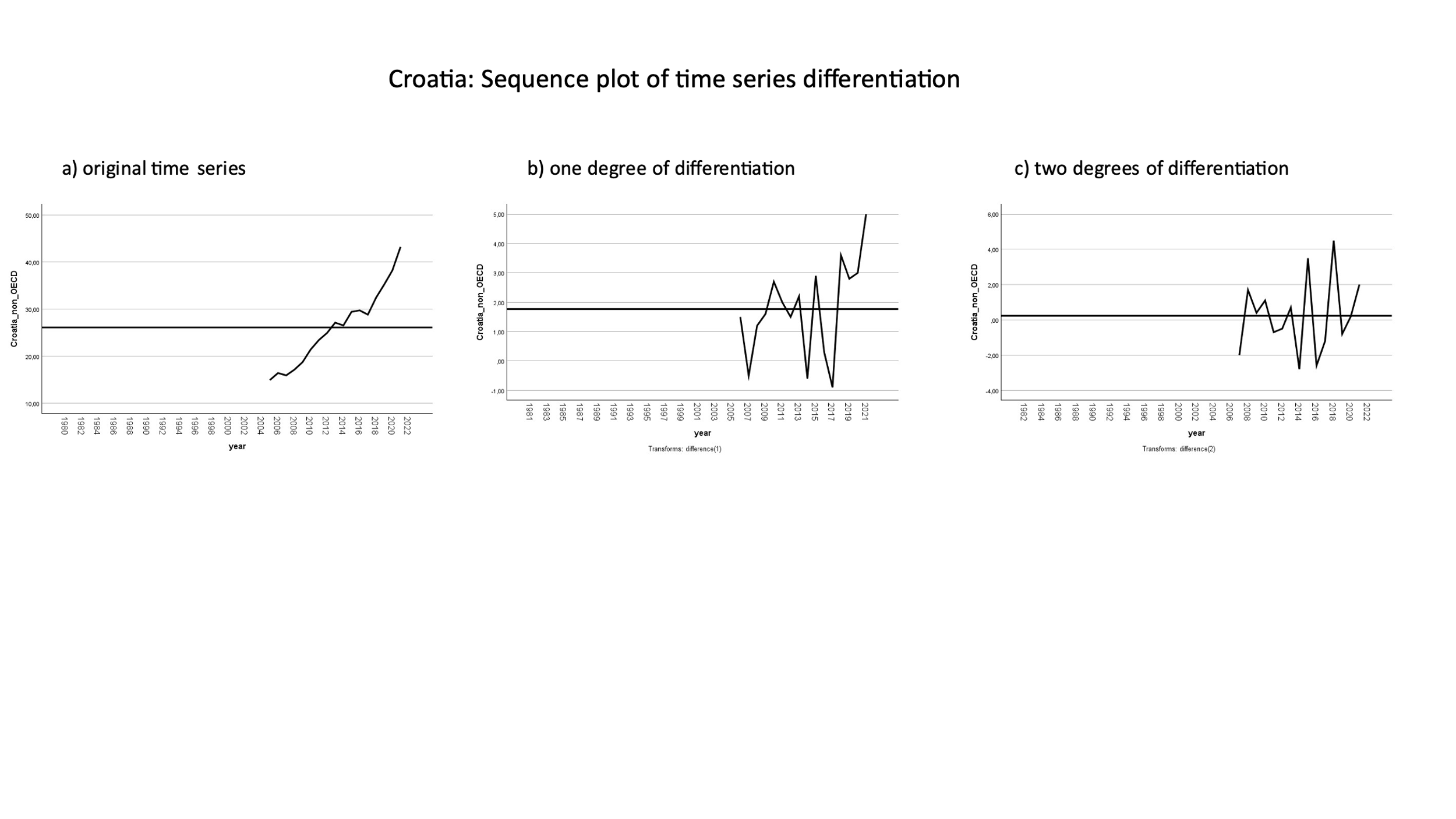


***Fig. S32:*** *Results of the autocorrelation, performed in SPSS. Depicted are the ACF and PACF plots for the original time series of Australia.*


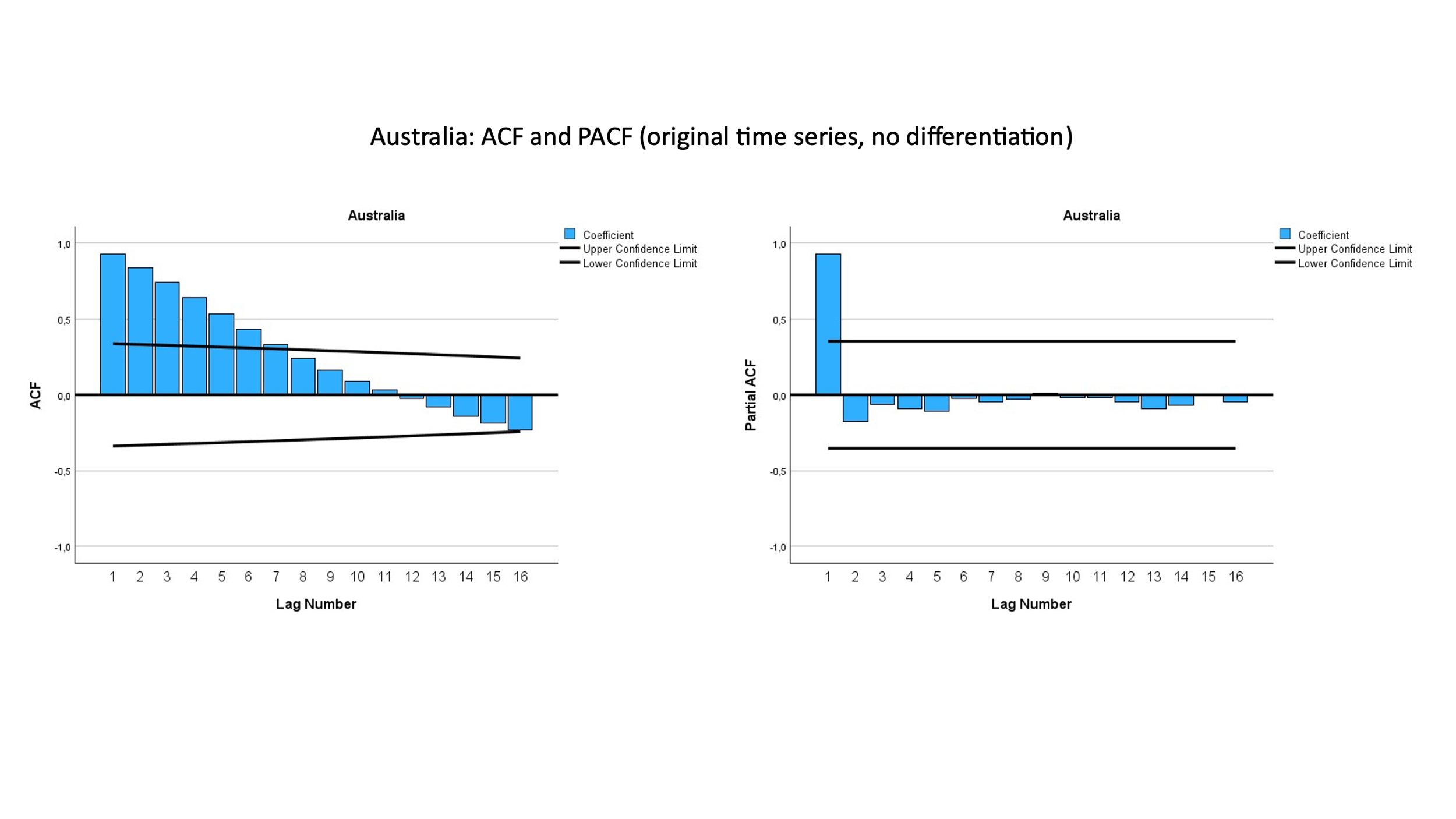


***Fig. S33:*** *Results of the autocorrelation, performed in SPSS. Depicted are the ACF and PACF plots for the original time series of Austria.*


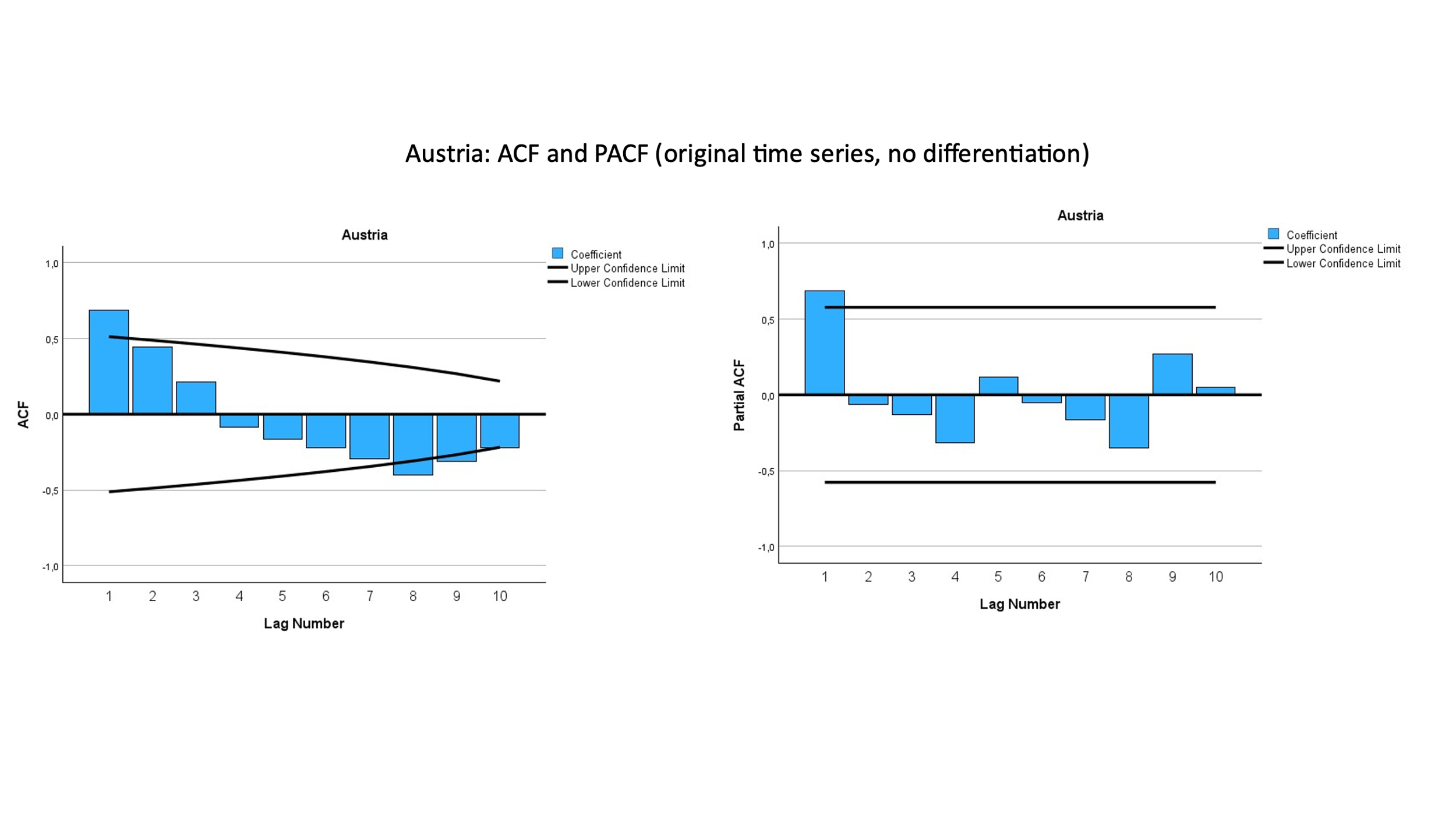


***Fig. S34:*** *Results of the autocorrelation, performed in SPSS. Depicted are the ACF and PACF plots for the original time series of Belgium.*


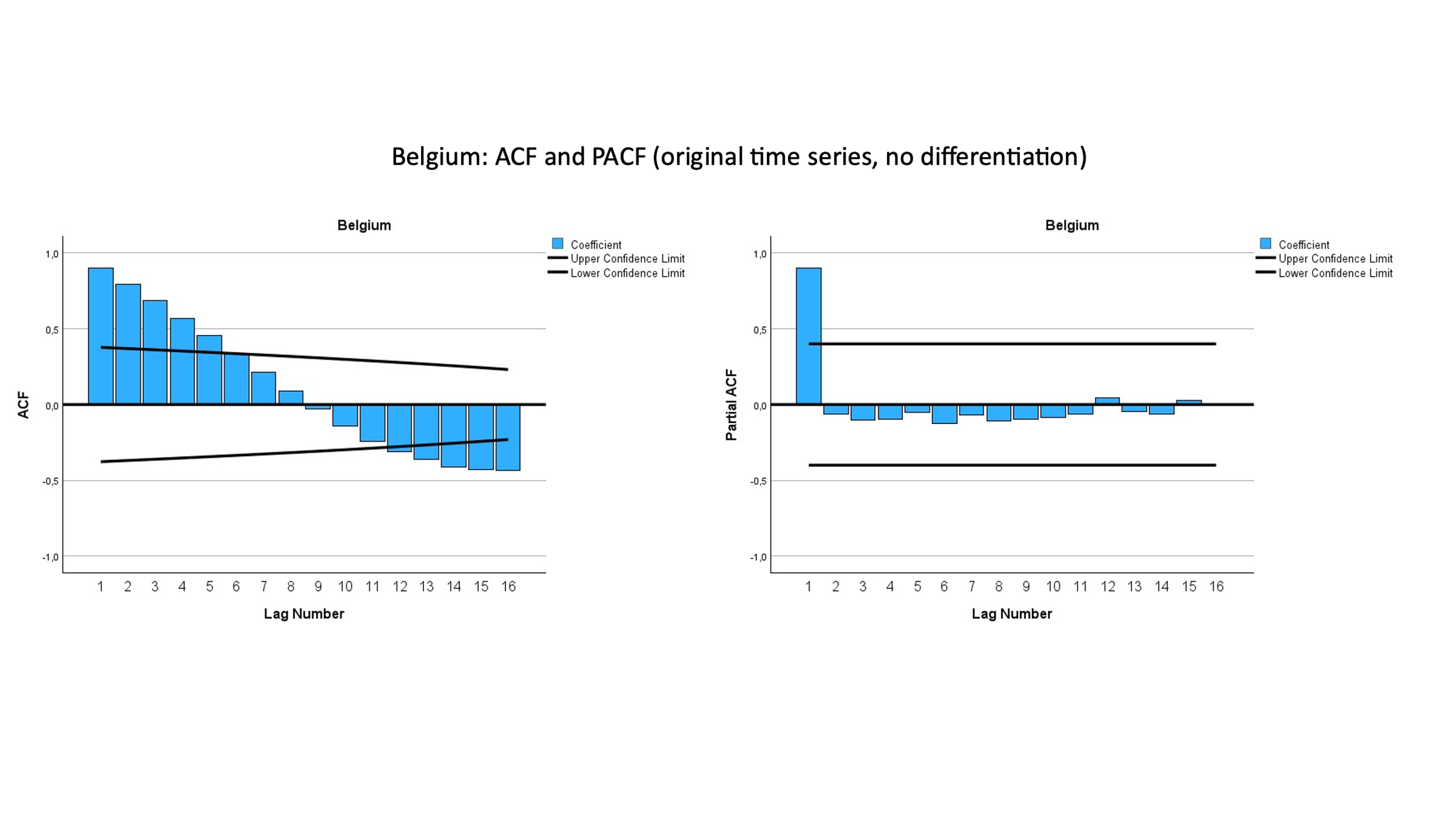


***Fig. S35:*** *Results of the autocorrelation, performed in SPSS. Depicted are the ACF and PACF plots for the original time series of Canada.*


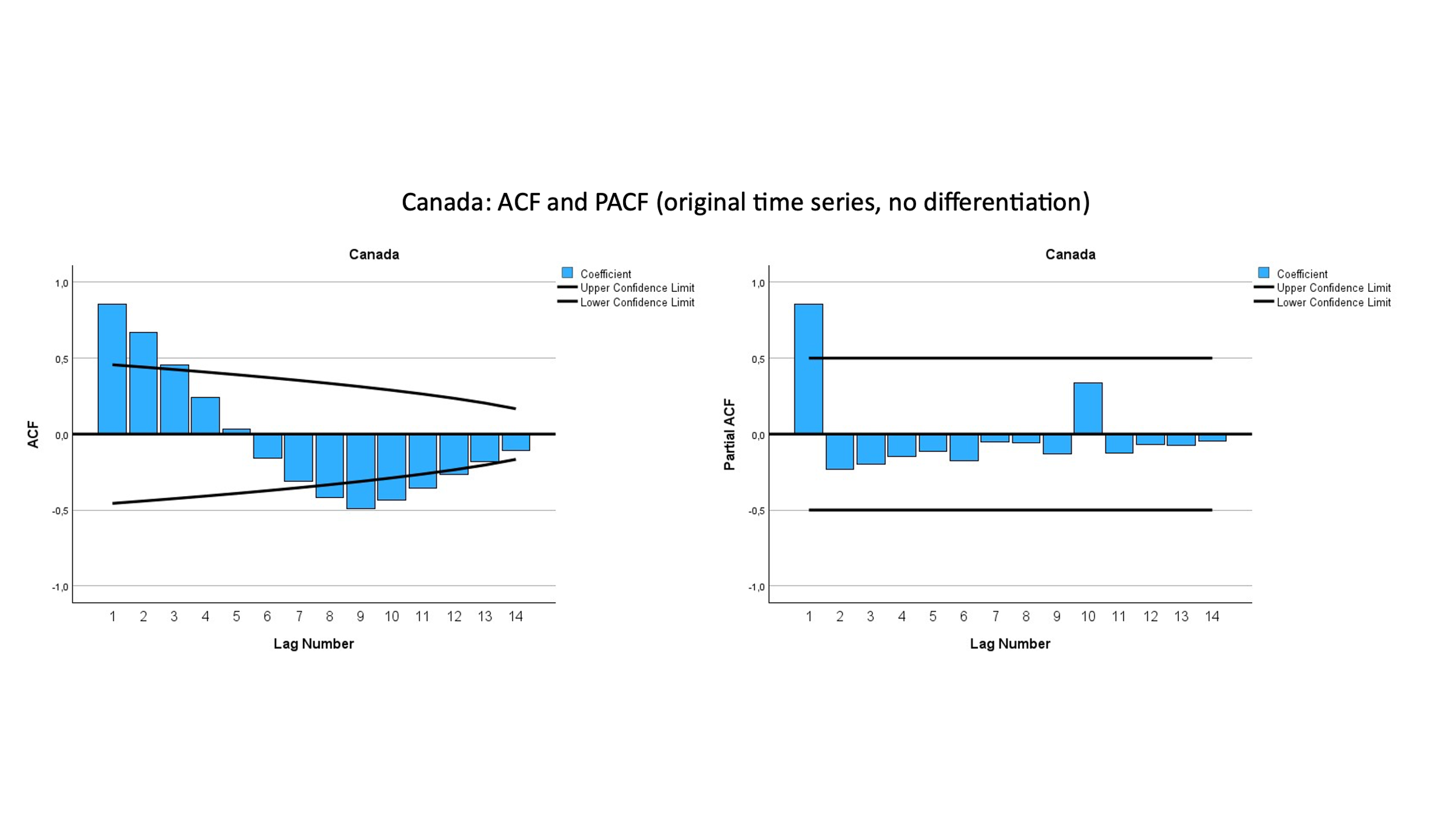


***Fig. S36:*** *Results of the autocorrelation, performed in SPSS. Depicted are the ACF and PACF plots for the original time series of Chile.*


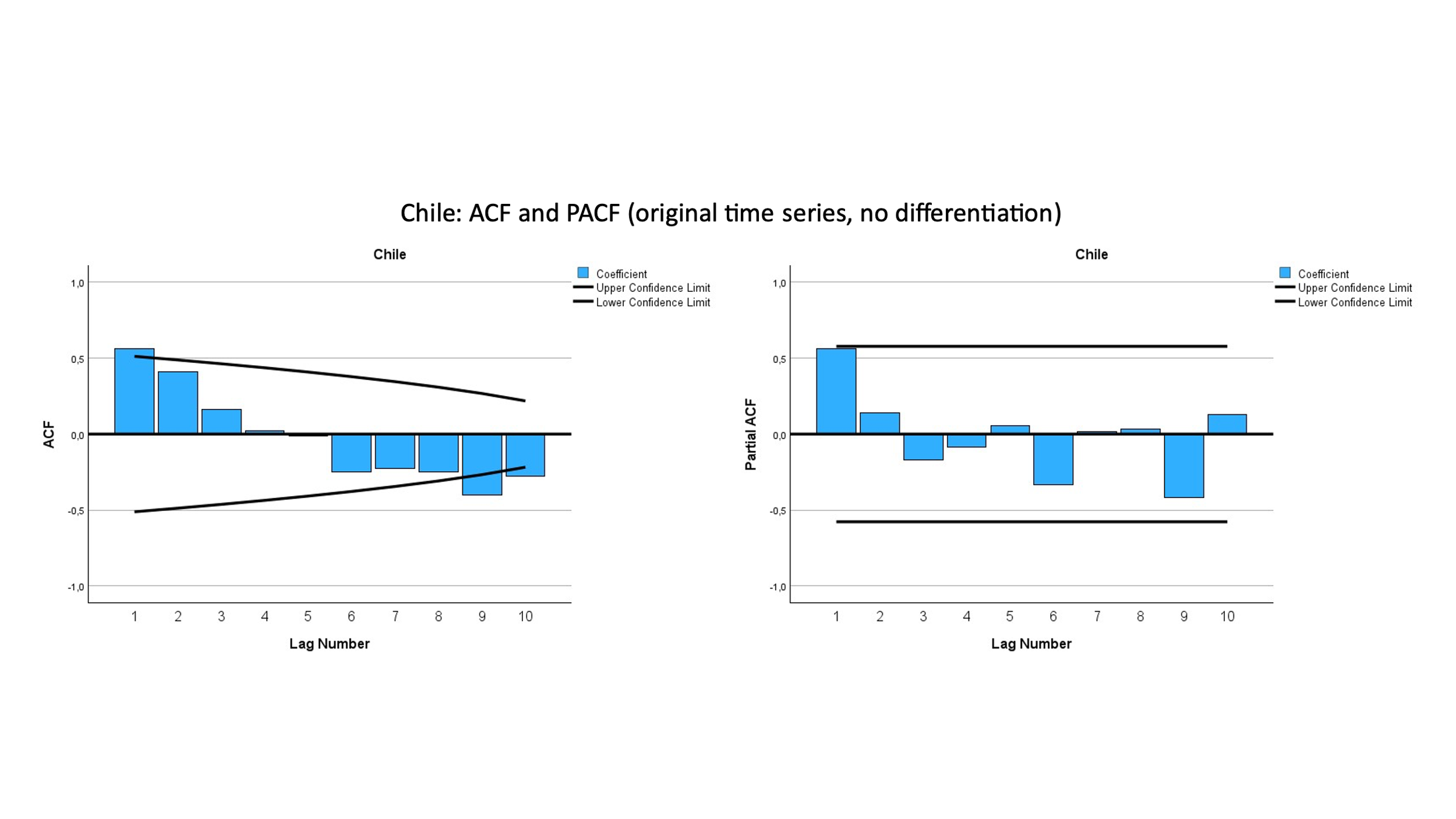


***Fig. S37:*** *Results of the autocorrelation, performed in SPSS. Depicted are the ACF and PACF plots for the original time series of Costa Rica.*


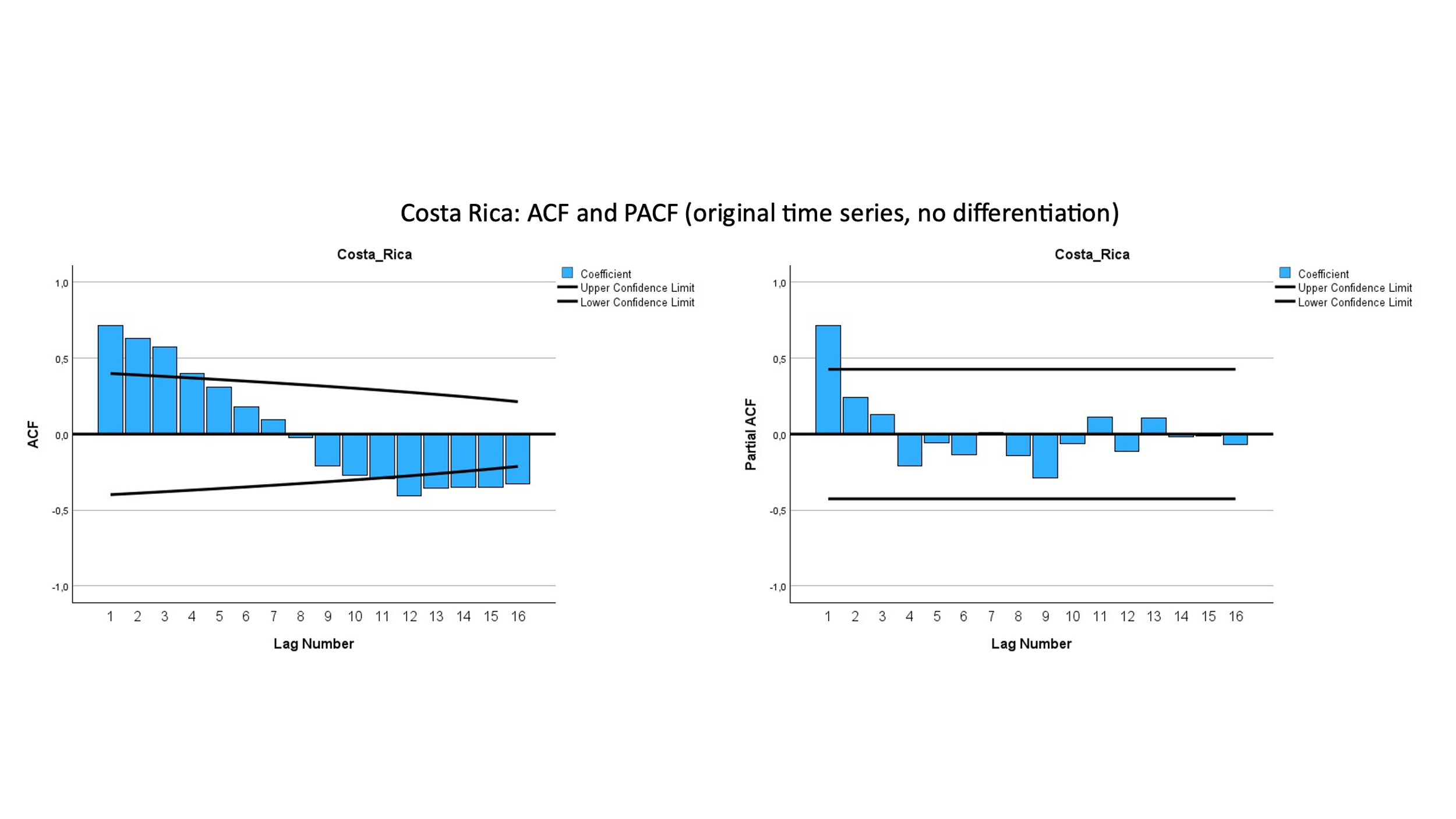


***Fig. S38:*** *Results of the autocorrelation, performed in SPSS. Depicted are the ACF and PACF plots for the original time series of Czechia.*


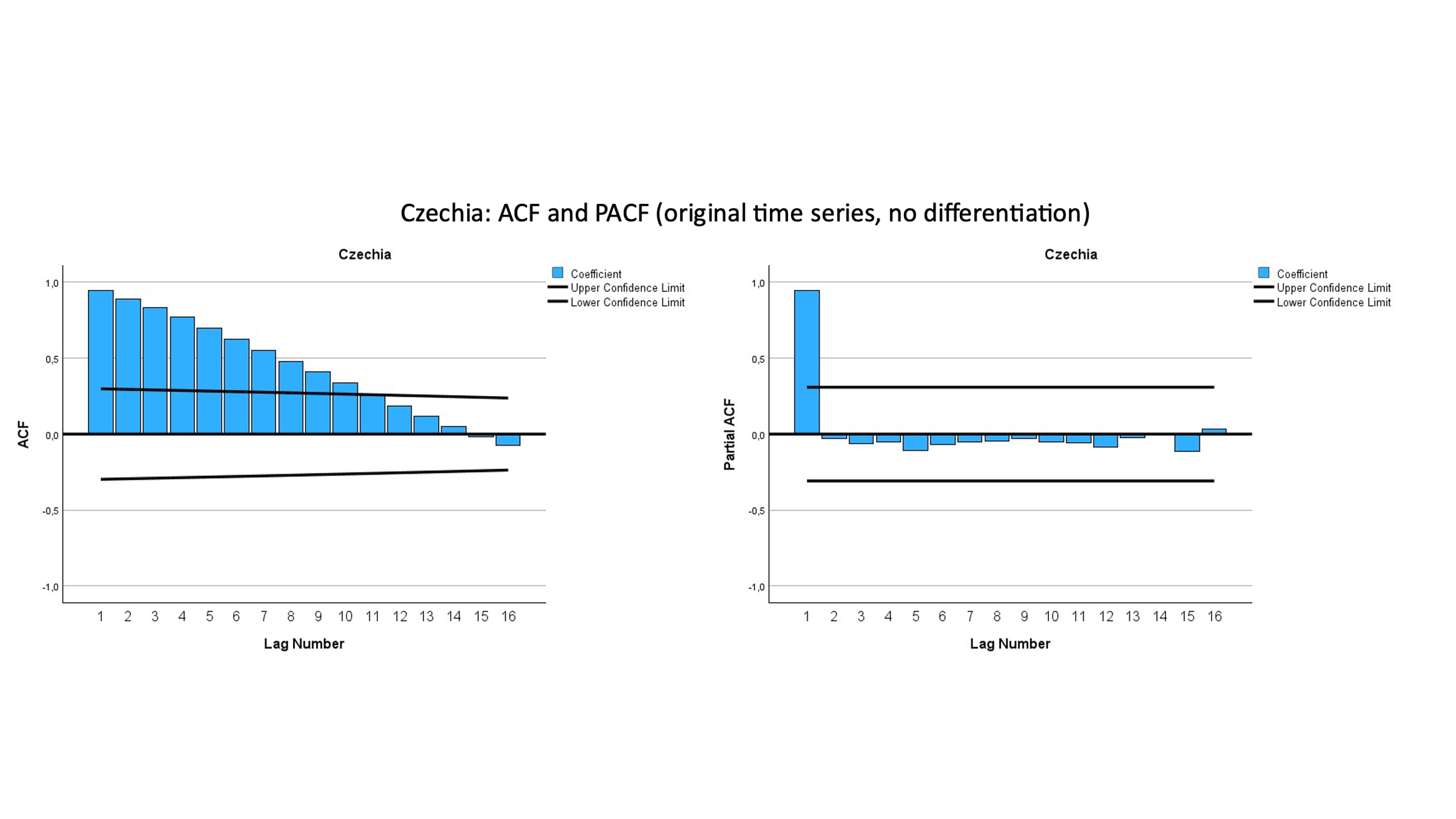


***Fig. S39:*** *Results of the autocorrelation, performed in SPSS. Depicted are the ACF and PACF plots for the original time series of Denmark.*


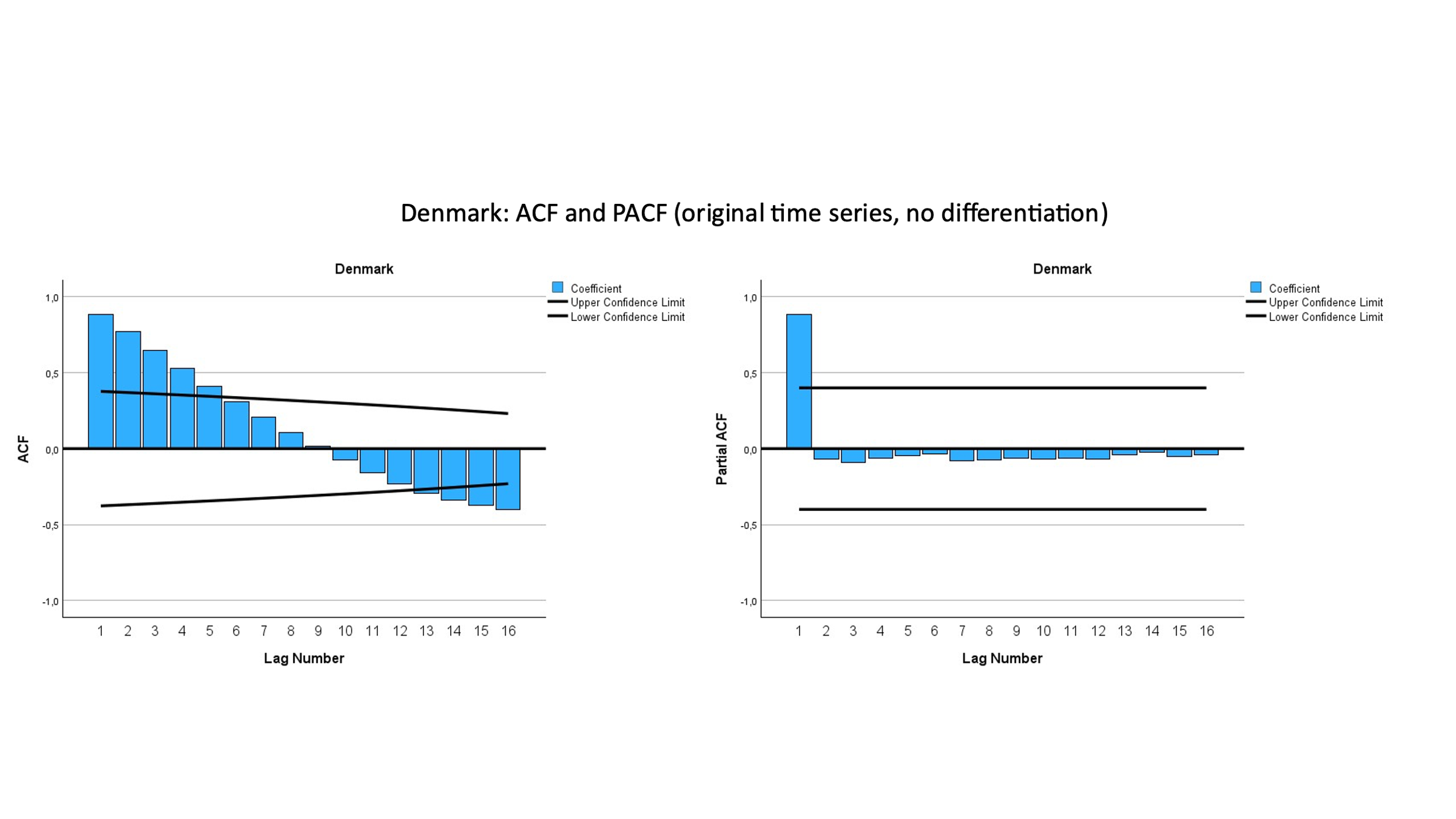


***Fig. S40:*** *Results of the autocorrelation, performed in SPSS. Depicted are the ACF and PACF plots for the original time series of Estonia.*


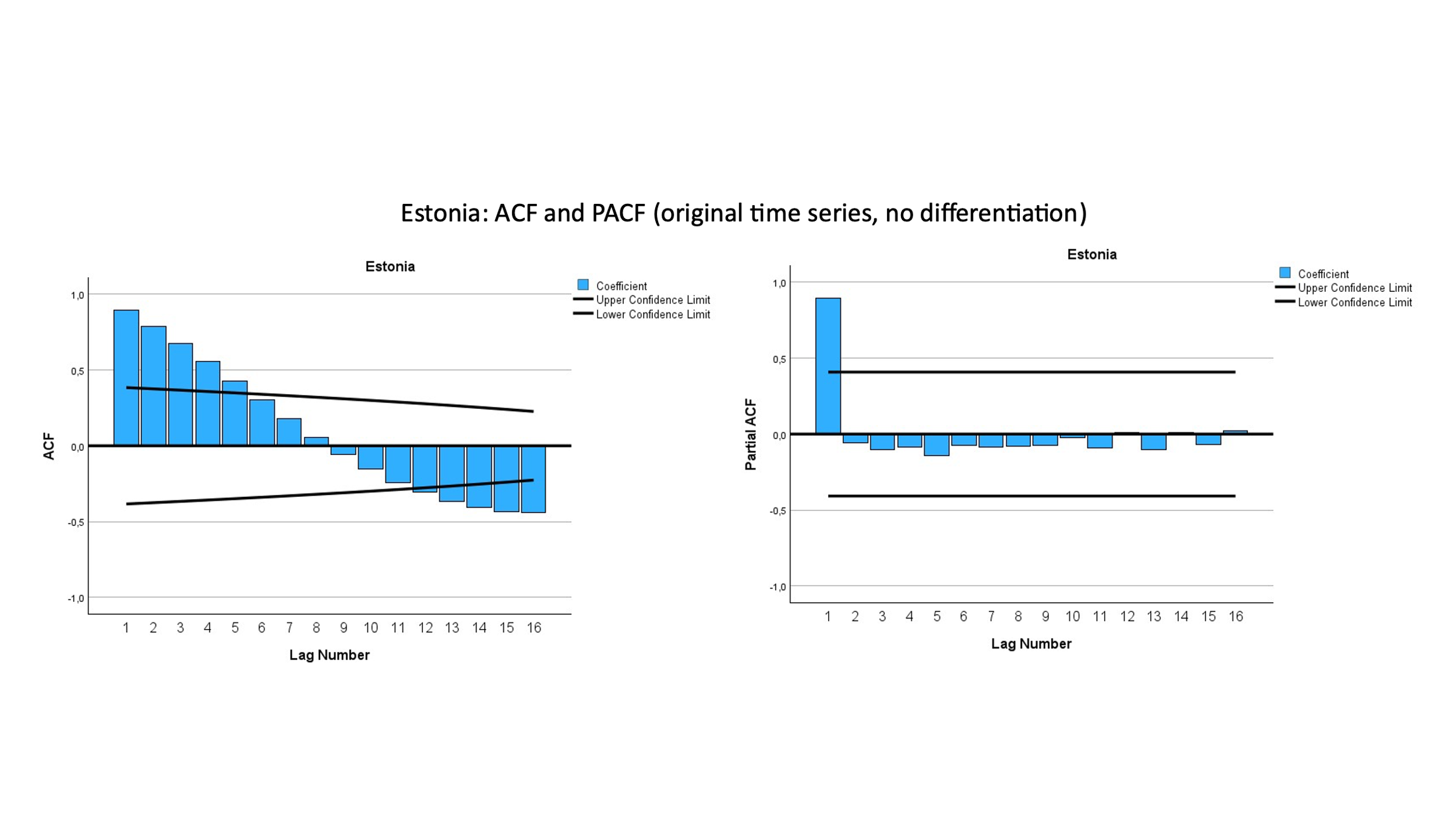


***Fig. S41:*** *Results of the autocorrelation, performed in SPSS. Depicted are the ACF and PACF plots for the original time series of Finland.*


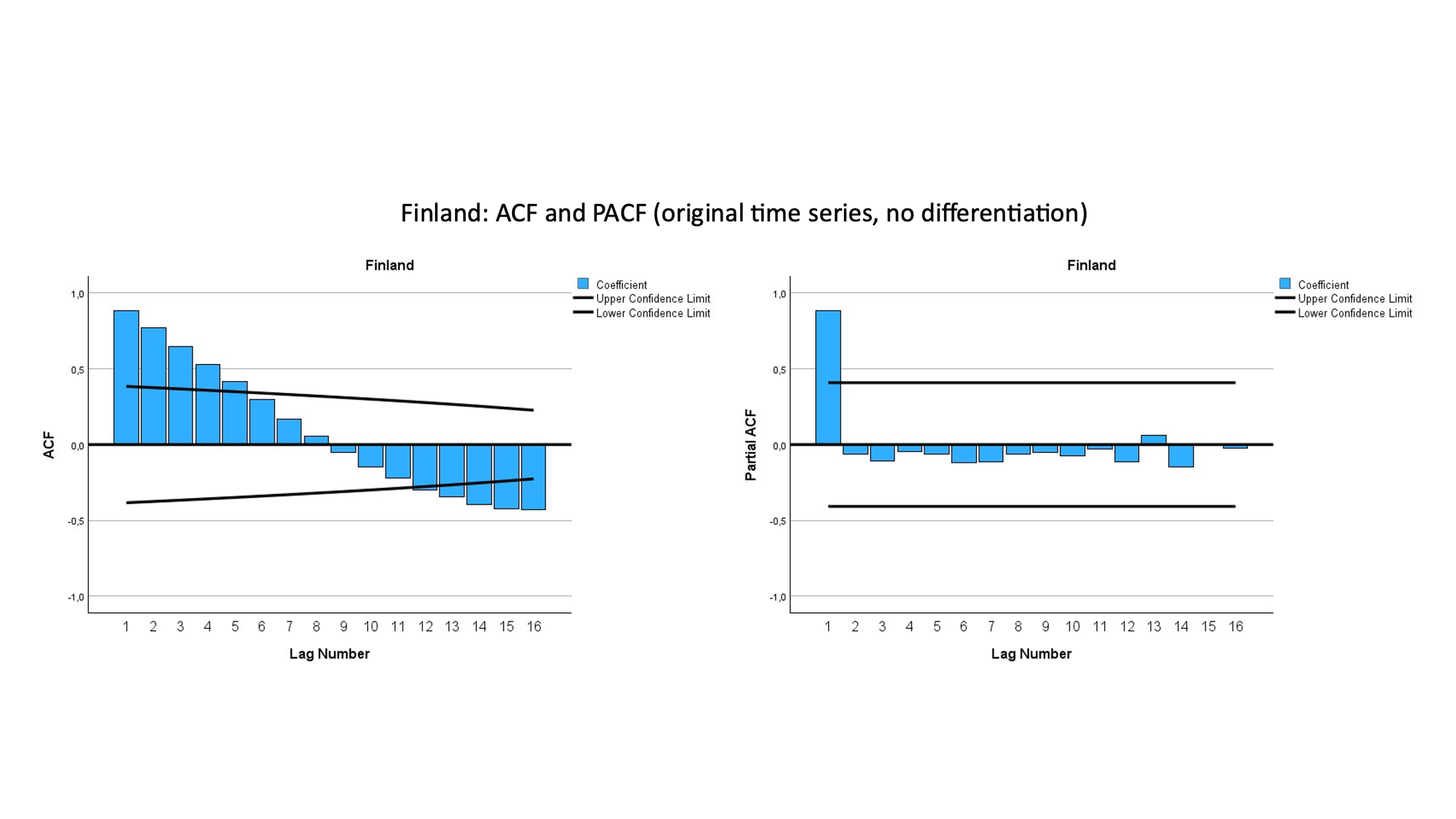


***Fig. S42:*** *Results of the autocorrelation, performed in SPSS. Depicted are the ACF and PACF plots for the original time series of France.*


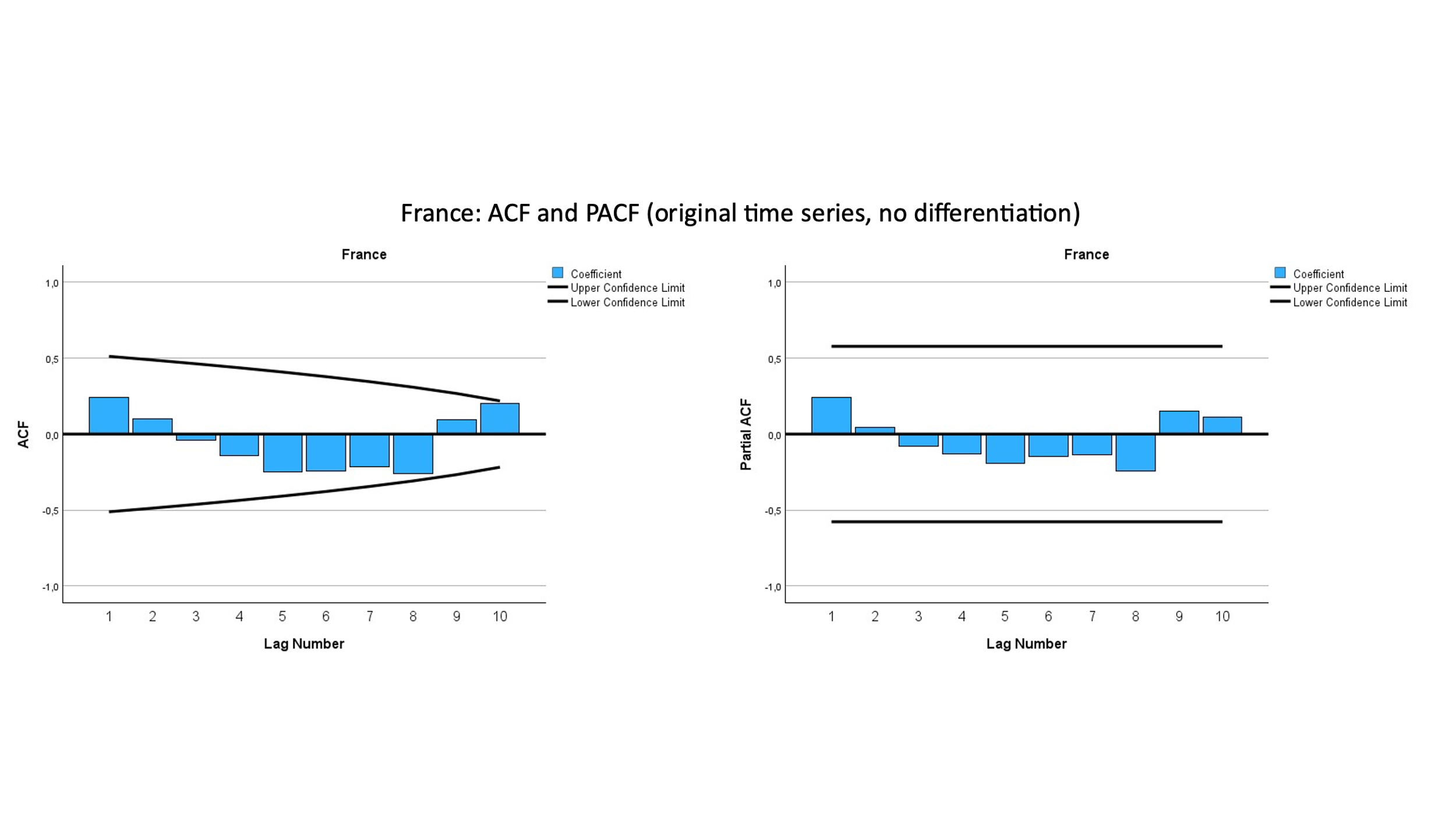


***Fig. S43:*** *Results of the autocorrelation, performed in SPSS. Depicted are the ACF and PACF plots for the original time series of Germany.*


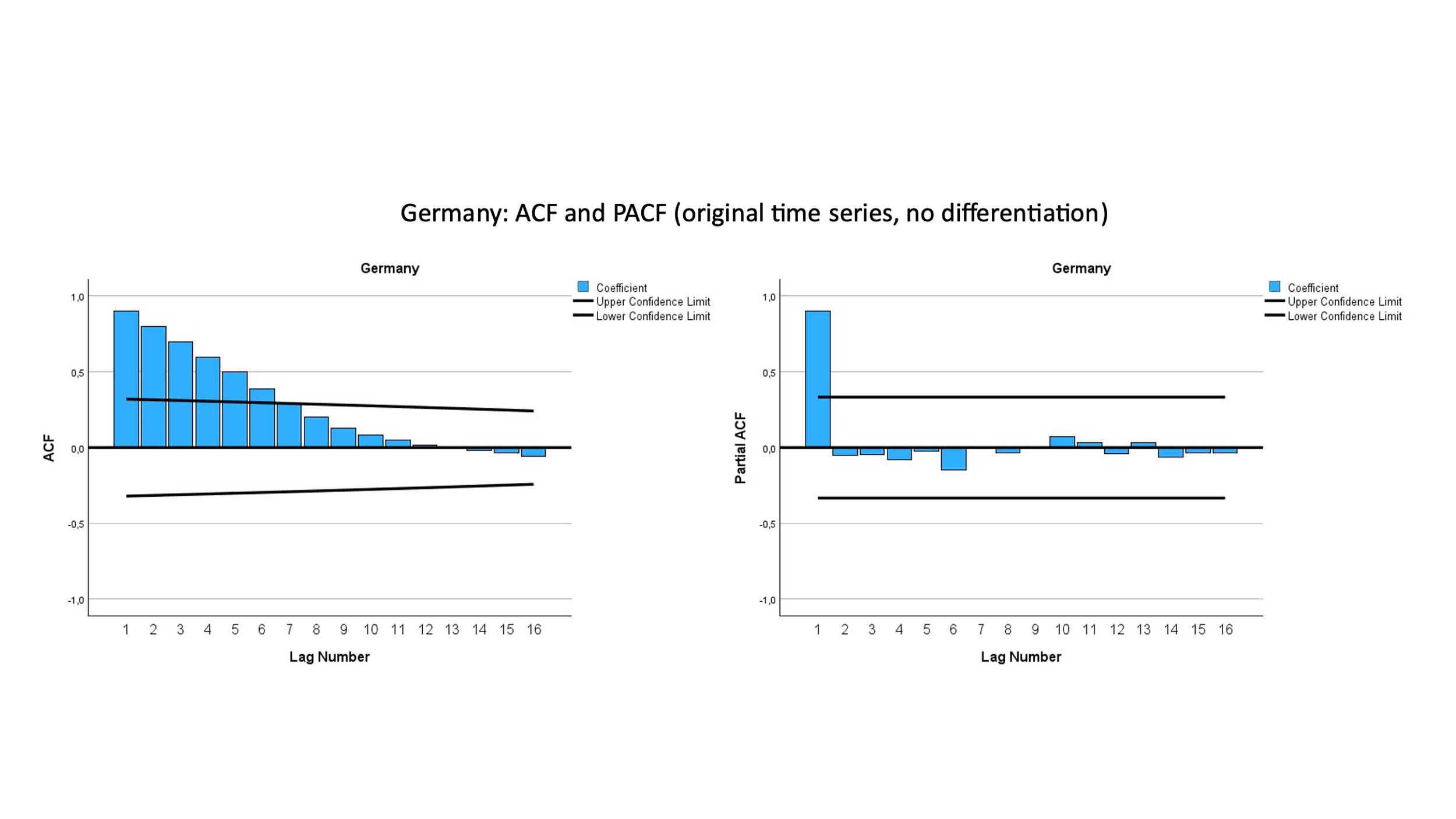


***Fig. S44:*** *Results of the autocorrelation, performed in SPSS. Depicted are the ACF and PACF plots for the original time series of Greece.*


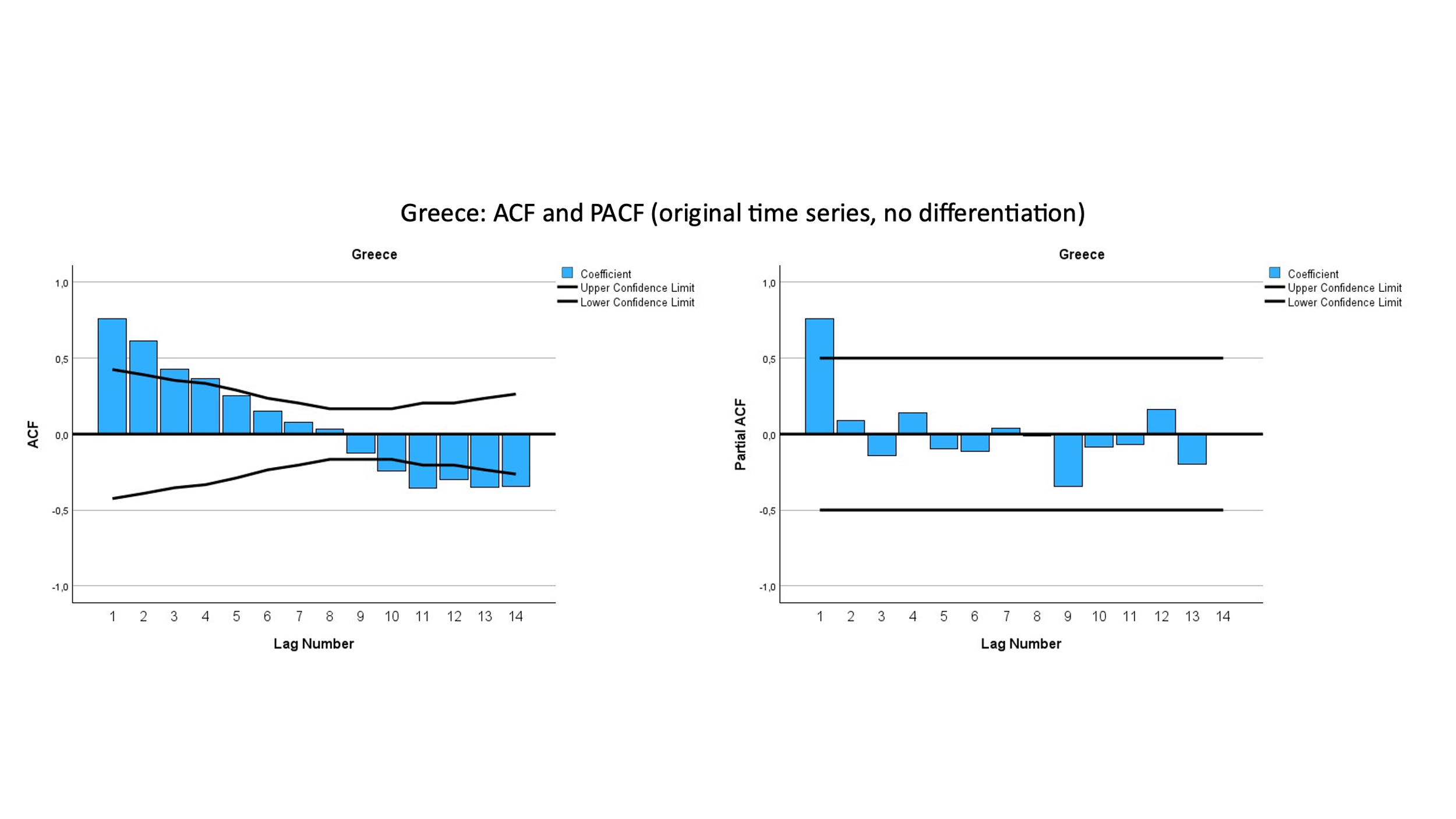


***Fig. S45:*** *Results of the autocorrelation, performed in SPSS. Depicted are the ACF and PACF plots for the original time series of Hungary.*


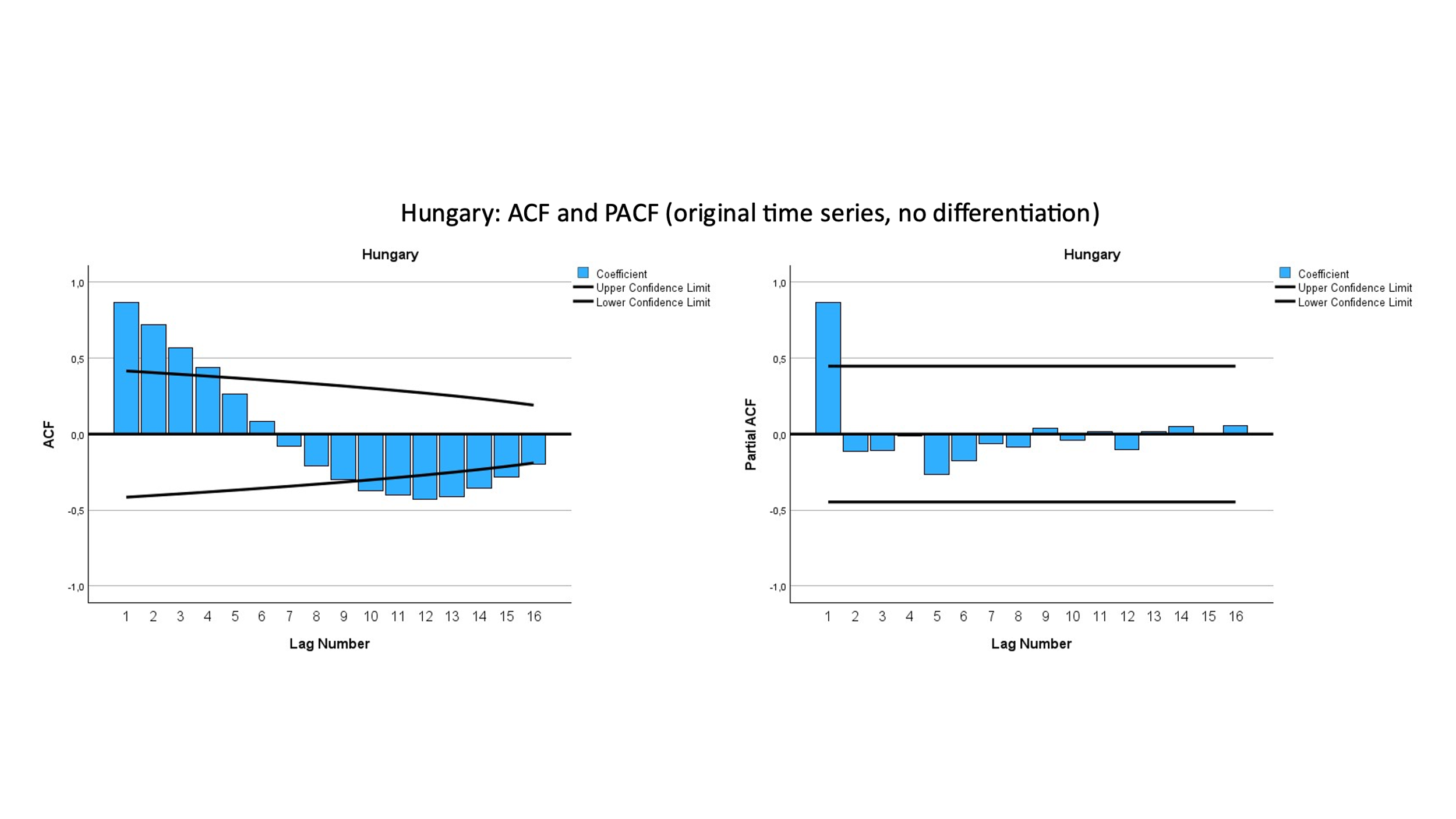


***Fig. S46:*** *Results of the autocorrelation, performed in SPSS. Depicted are the ACF and PACF plots for the original time series of Iceland.*


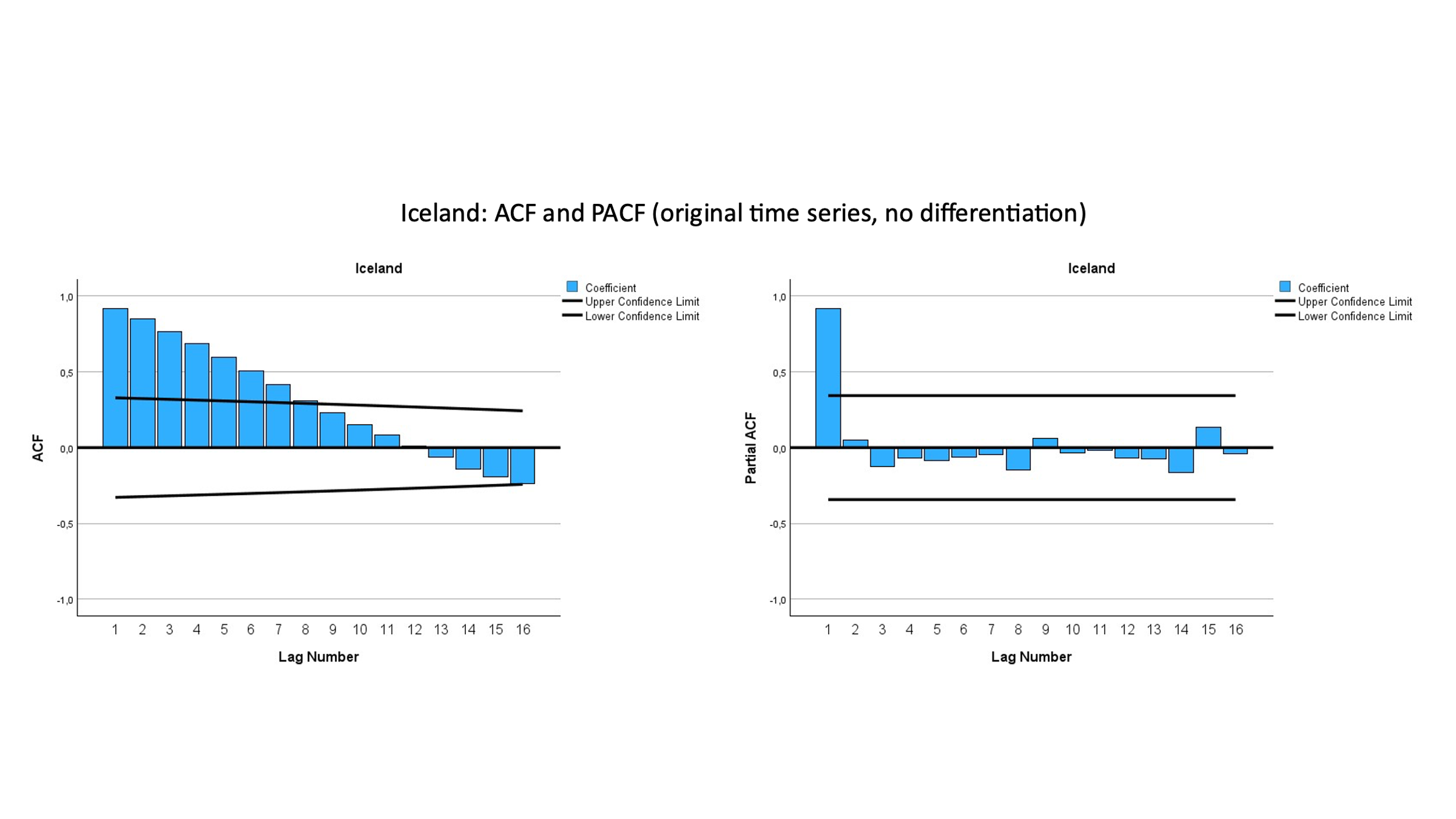


***Fig. S47:*** *Results of the autocorrelation, performed in SPSS. Depicted are the ACF and PACF plots for the original time series of Israel.*


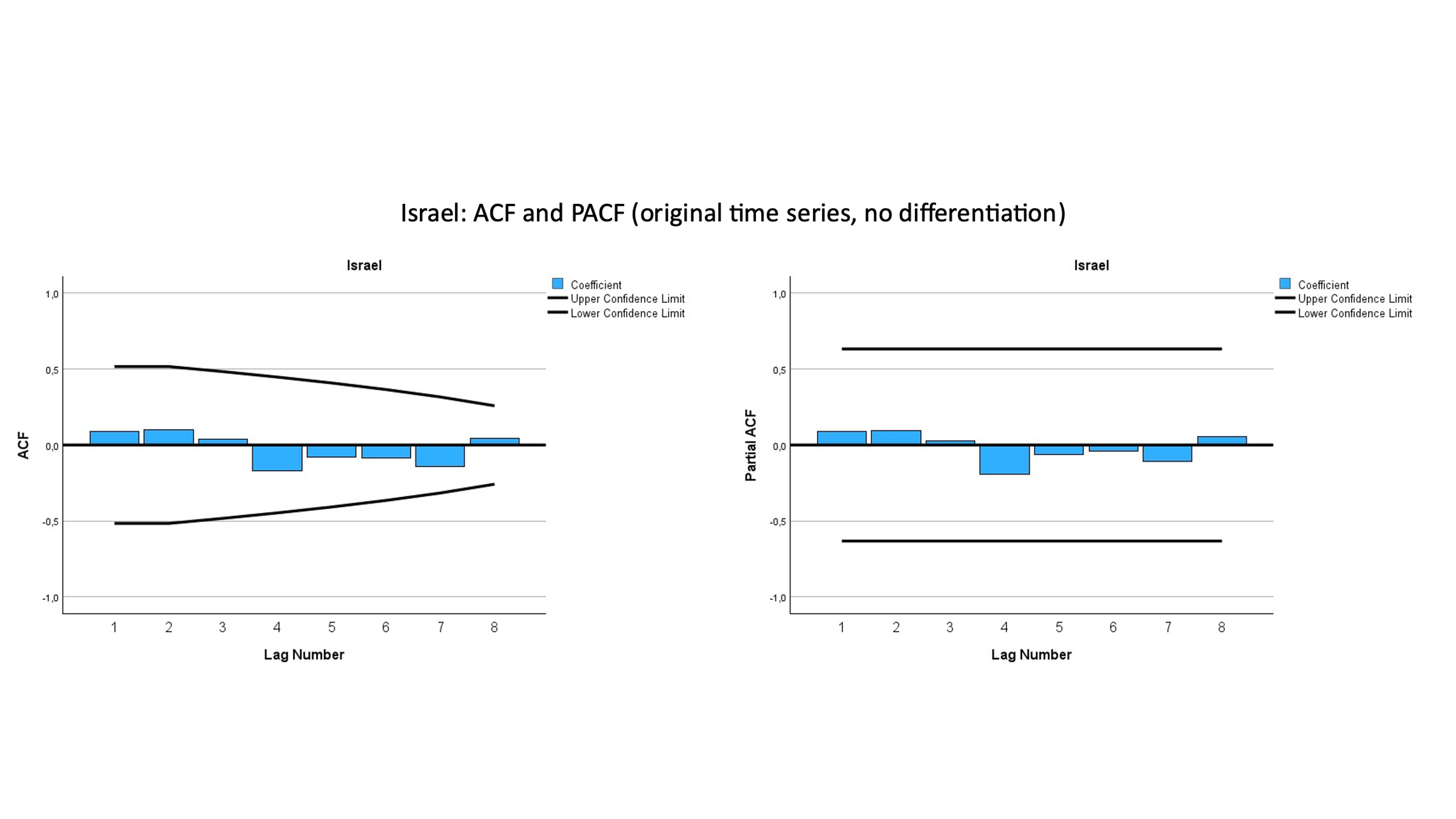


***Fig. S48:*** *Results of the autocorrelation, performed in SPSS. Depicted are the ACF and PACF plots for the original time series of Italy.*


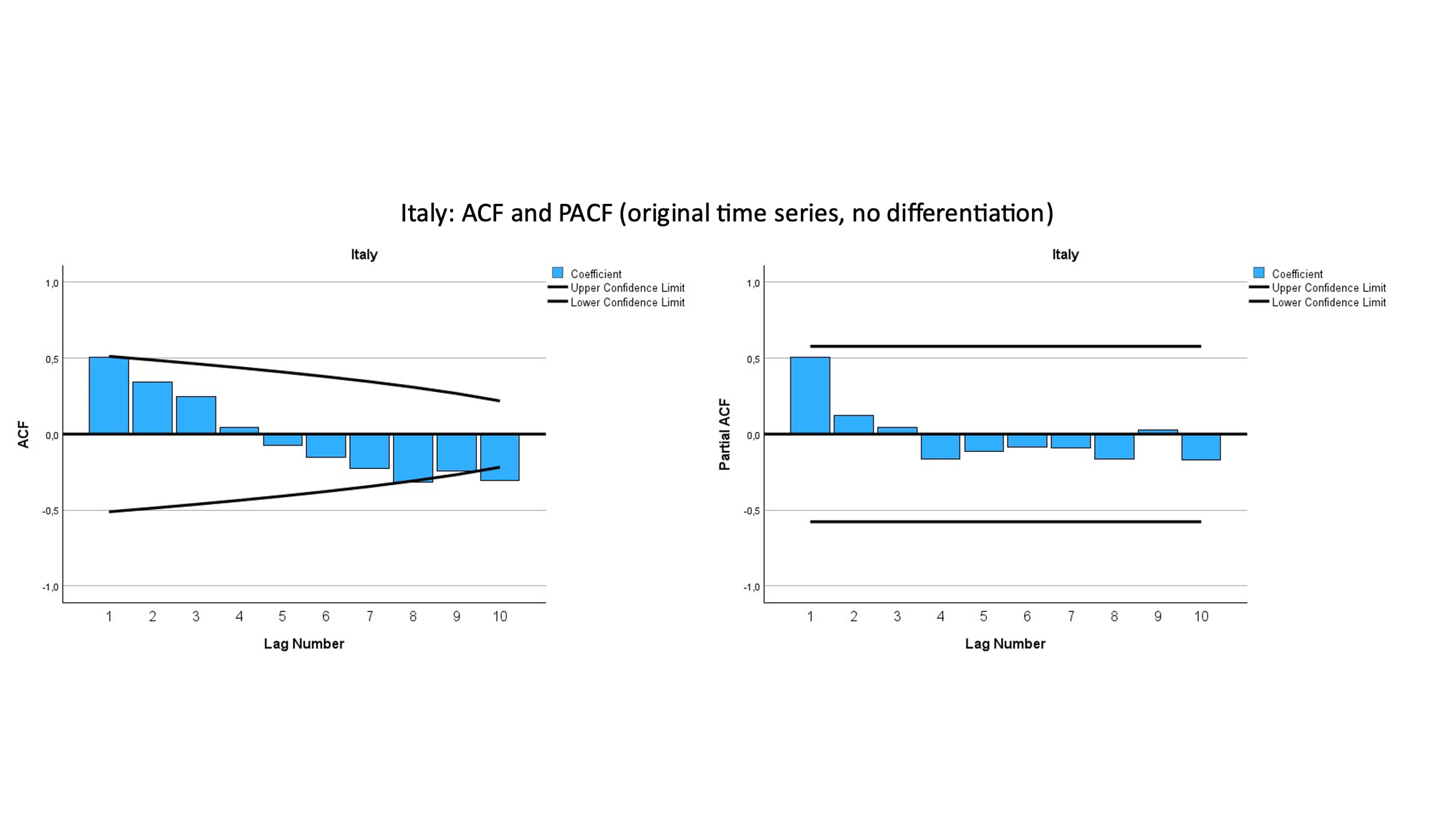


***Fig. S49:*** *Results of the autocorrelation, performed in SPSS. Depicted are the ACF and PACF plots for the original time series of Korea.*


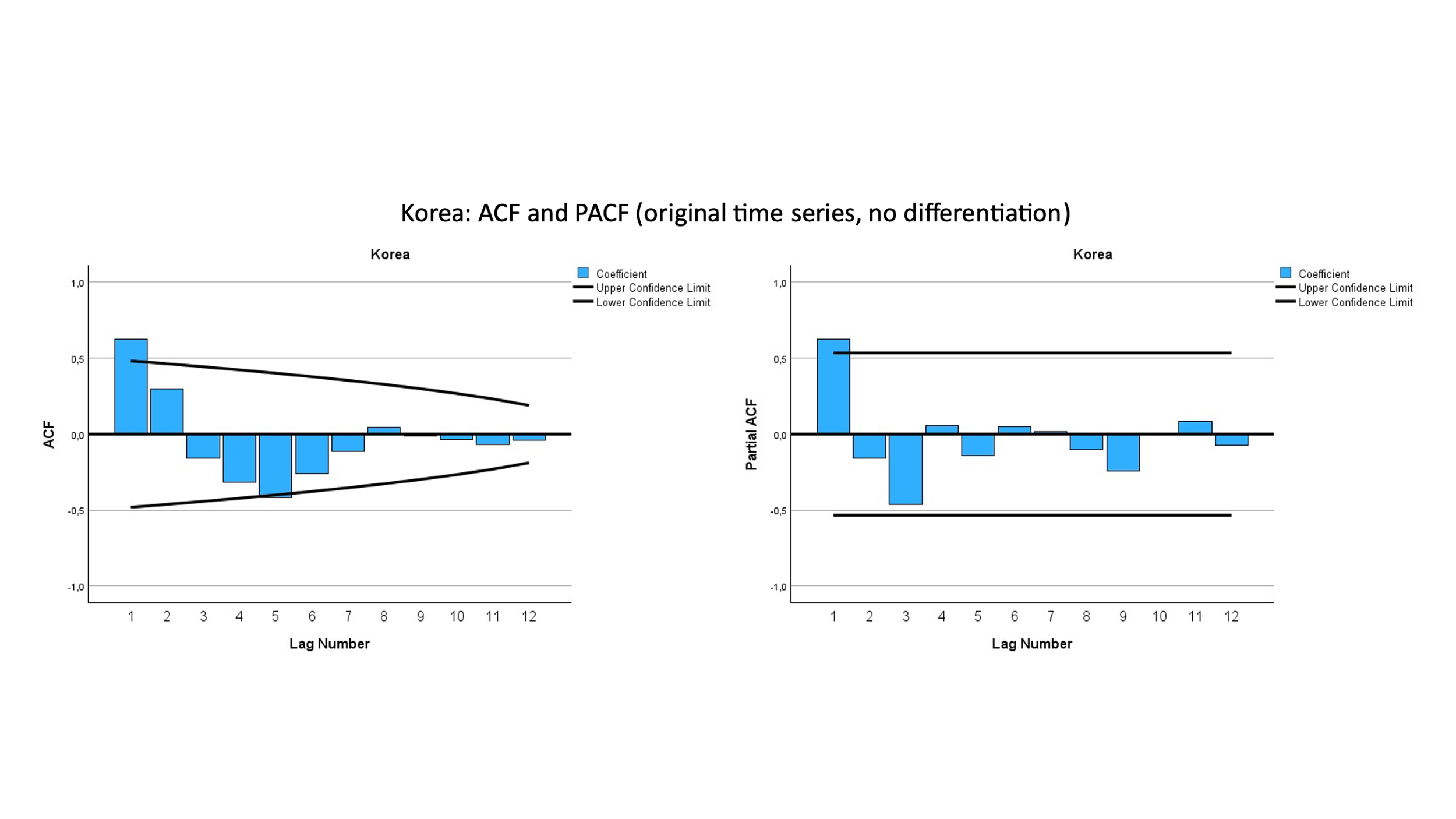


***Fig. S50:*** *Results of the autocorrelation, performed in SPSS. Depicted are the ACF and PACF plots for the original time series of Latvia.*


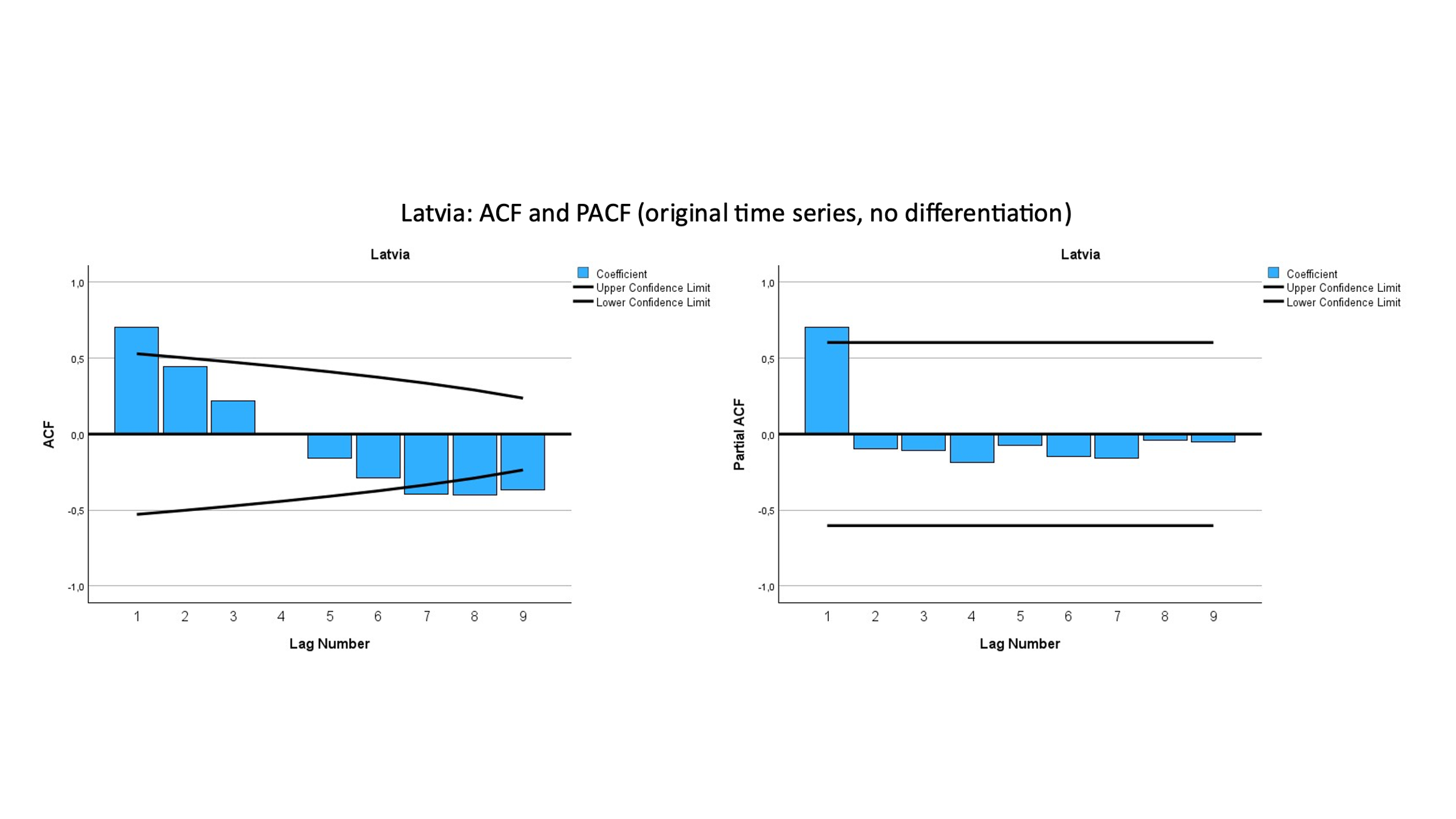


***Fig. S51:*** *Results of the autocorrelation, performed in SPSS. Depicted are the ACF and PACF plots for the original time series of Lithuania.*


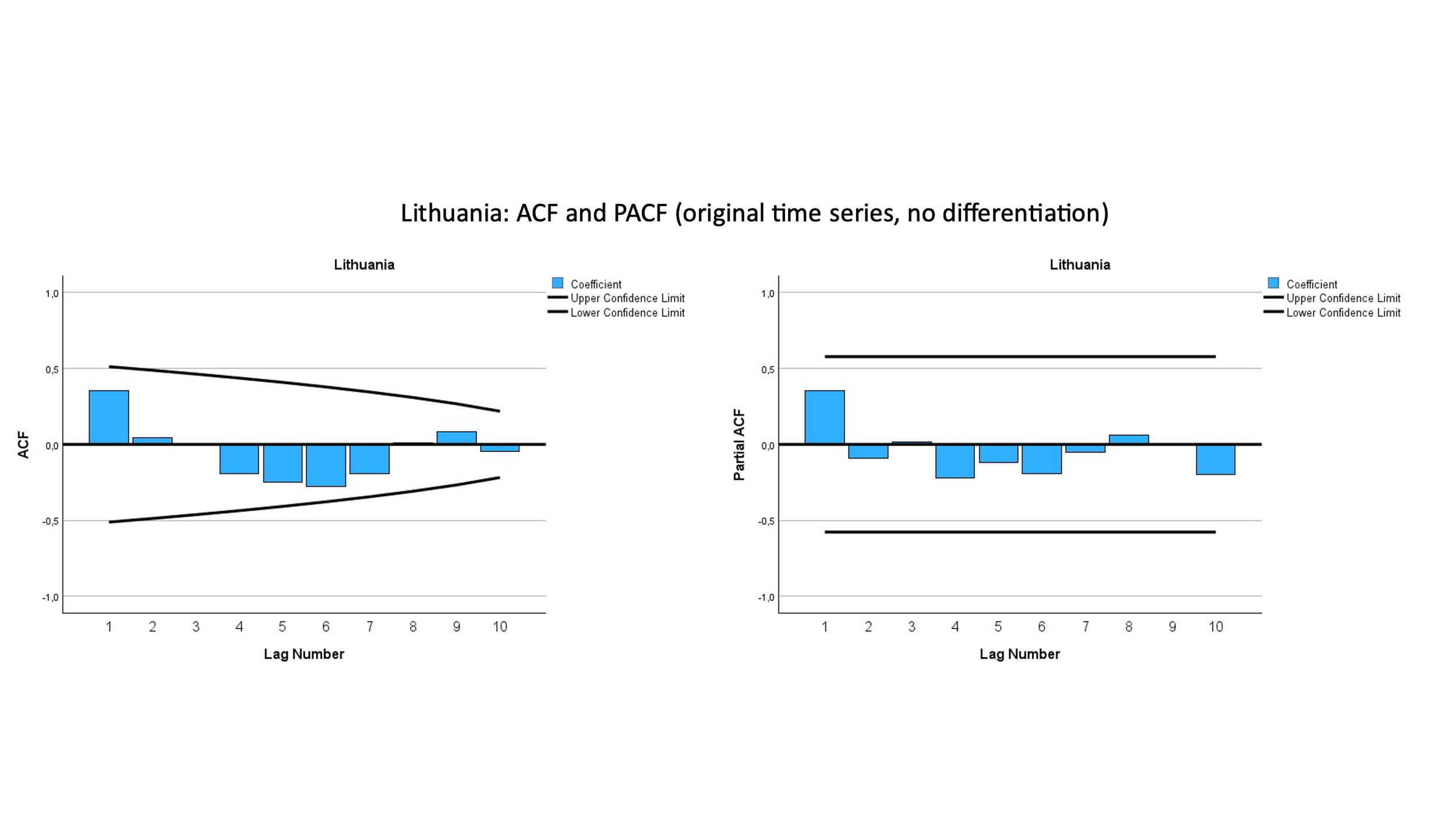


***Fig. S52:*** *Results of the autocorrelation, performed in SPSS. Depicted are the ACF and PACF plots for the original time series of Luxembourg.*


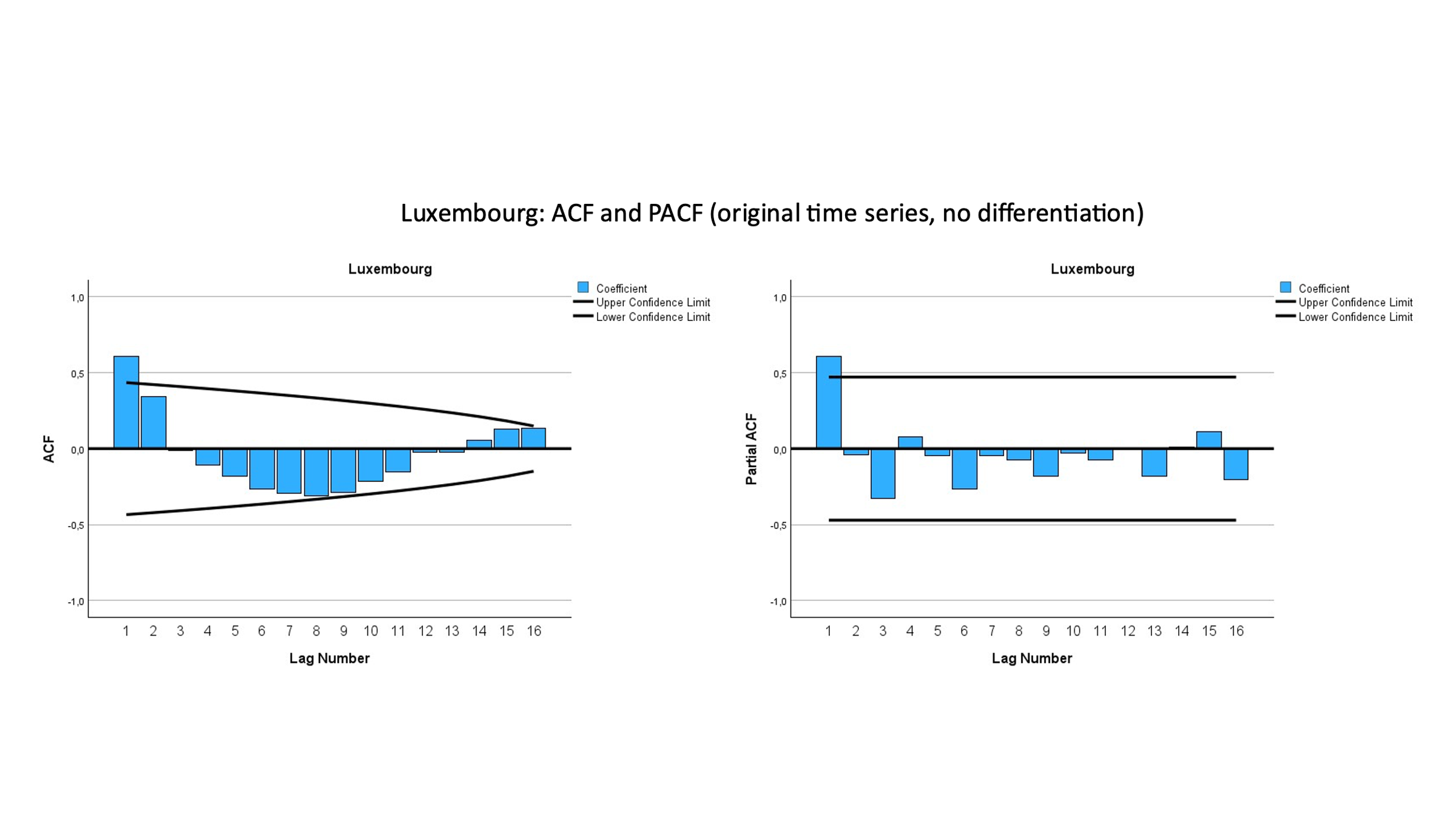


***Fig. S53:*** *Results of the autocorrelation, performed in SPSS. Depicted are the ACF and PACF plots for the original time series of the Netherlands.*


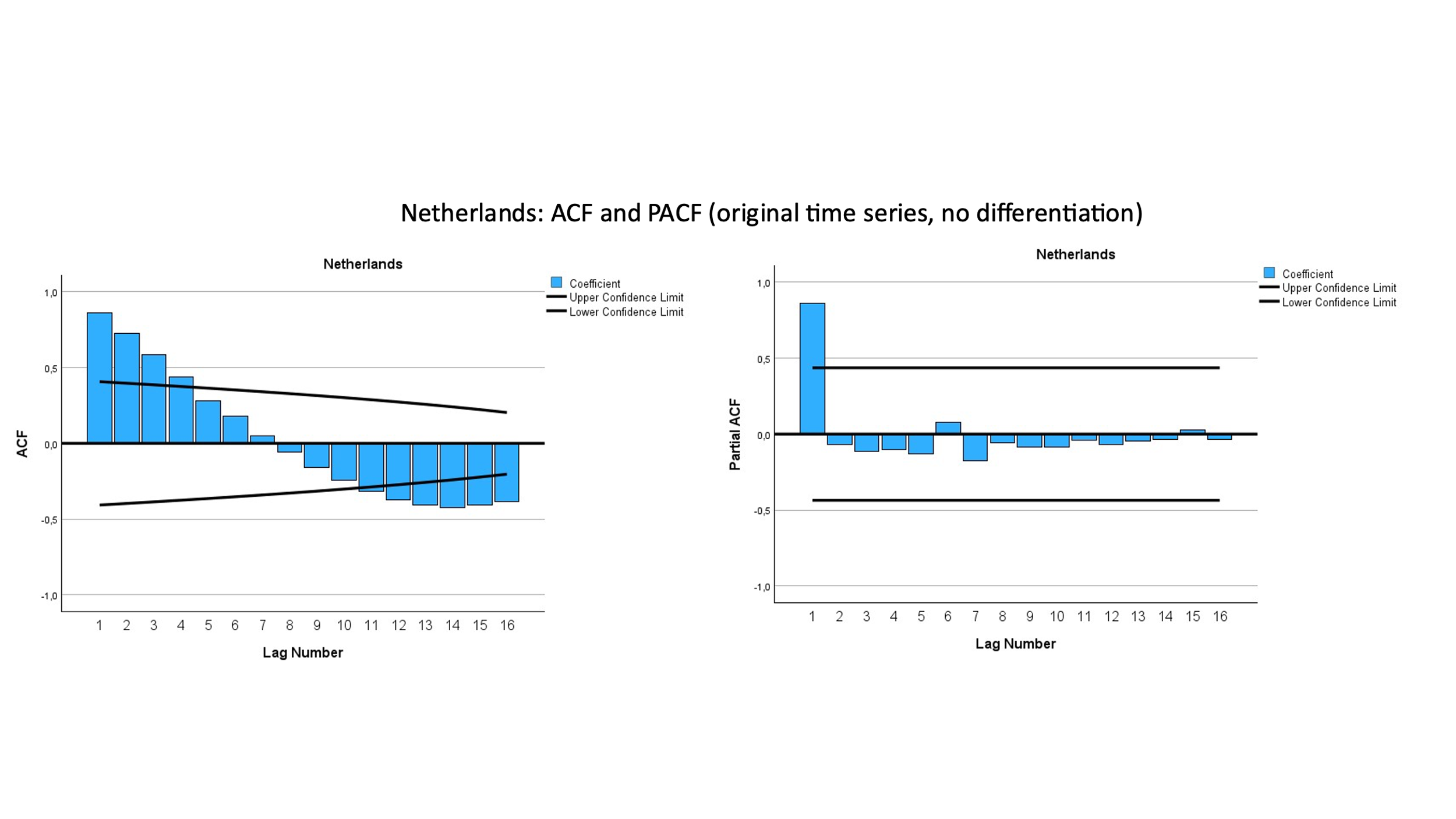


***Fig. S54:*** *Results of the autocorrelation, performed in SPSS. Depicted are the ACF and PACF plots for the original time series of Norway.*


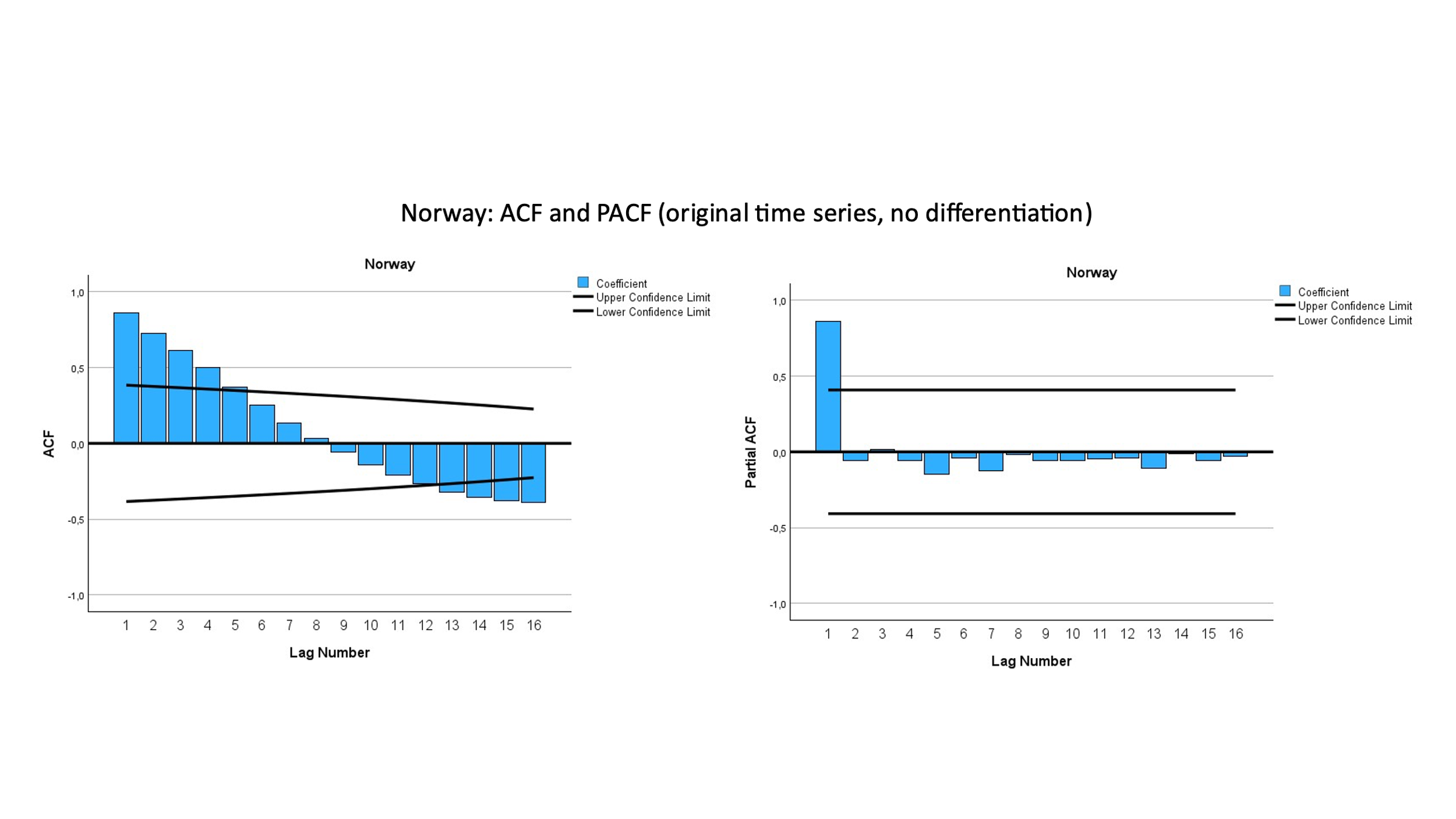


***Fig. S55:*** *Results of the autocorrelation, performed in SPSS. Depicted are the ACF and PACF plots for the original time series of Portugal.*


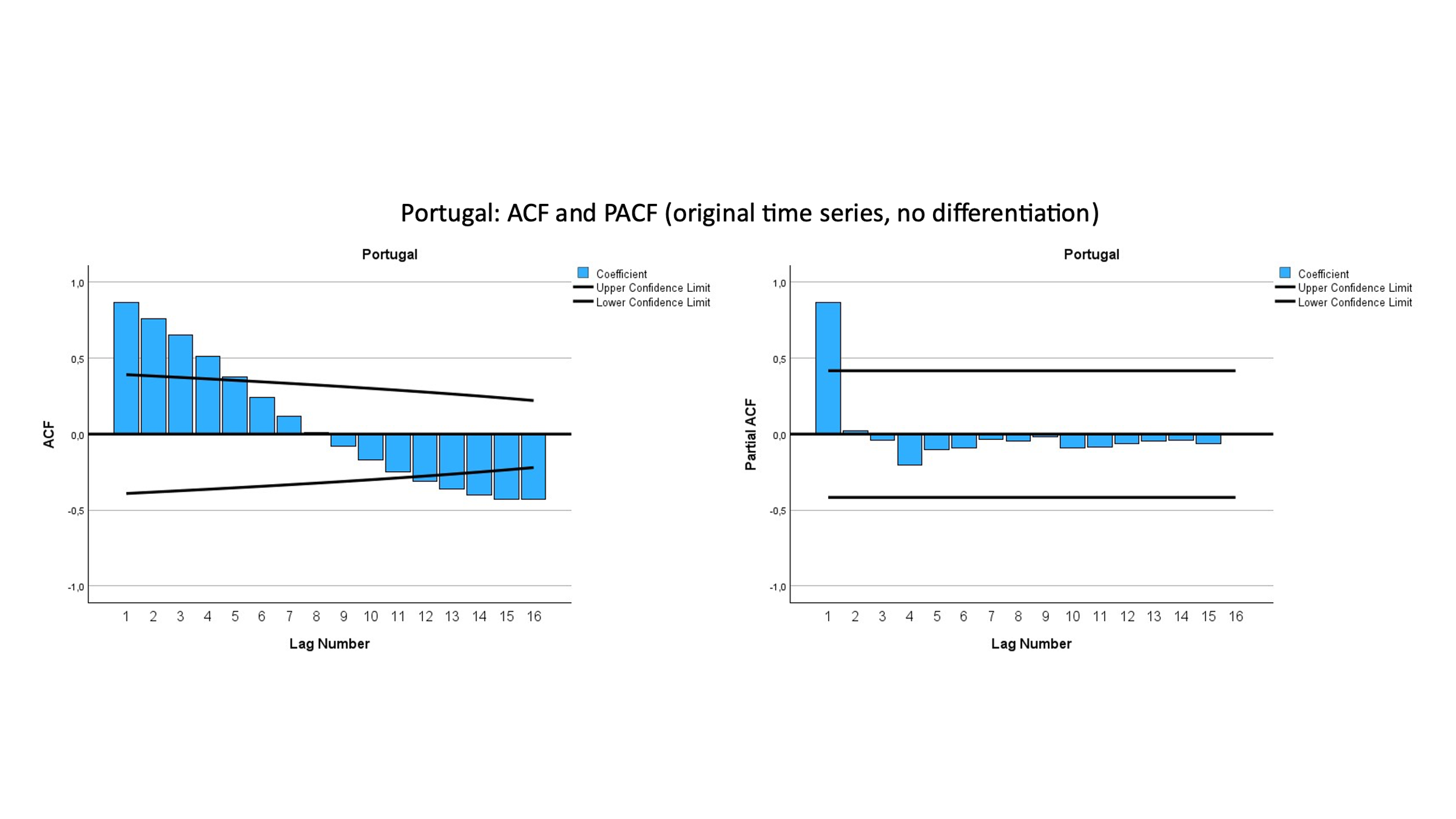


***Fig. S56:*** *Results of the autocorrelation, performed in SPSS. Depicted are the ACF and PACF plots for the original time series of Slovakia.*


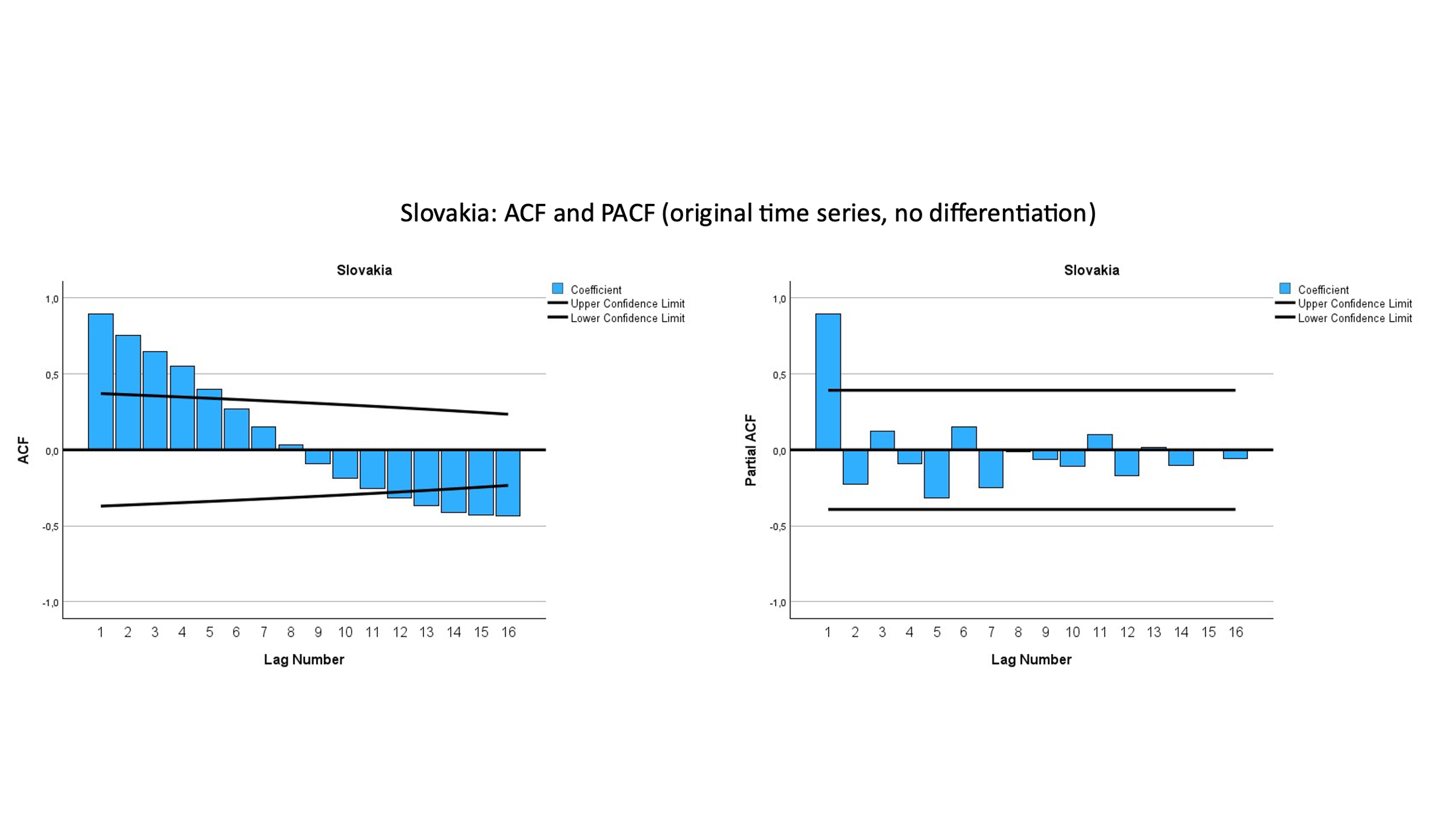


***Fig. S57:*** *Results of the autocorrelation, performed in SPSS. Depicted are the ACF and PACF plots for the original time series of Slovenia.*


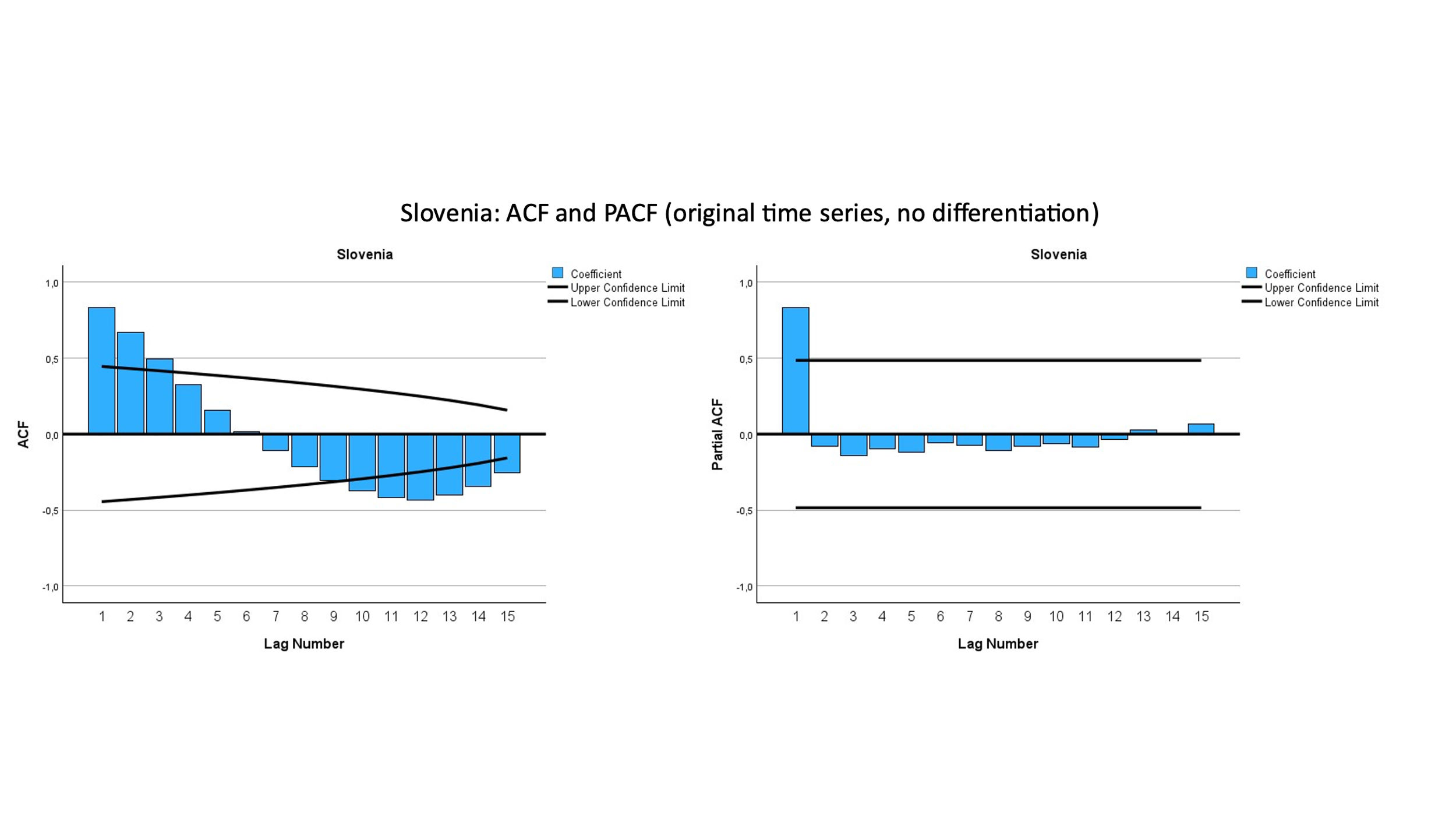


***Fig. S58:*** *Results of the autocorrelation, performed in SPSS. Depicted are the ACF and PACF plots for the original time series of Spain.*


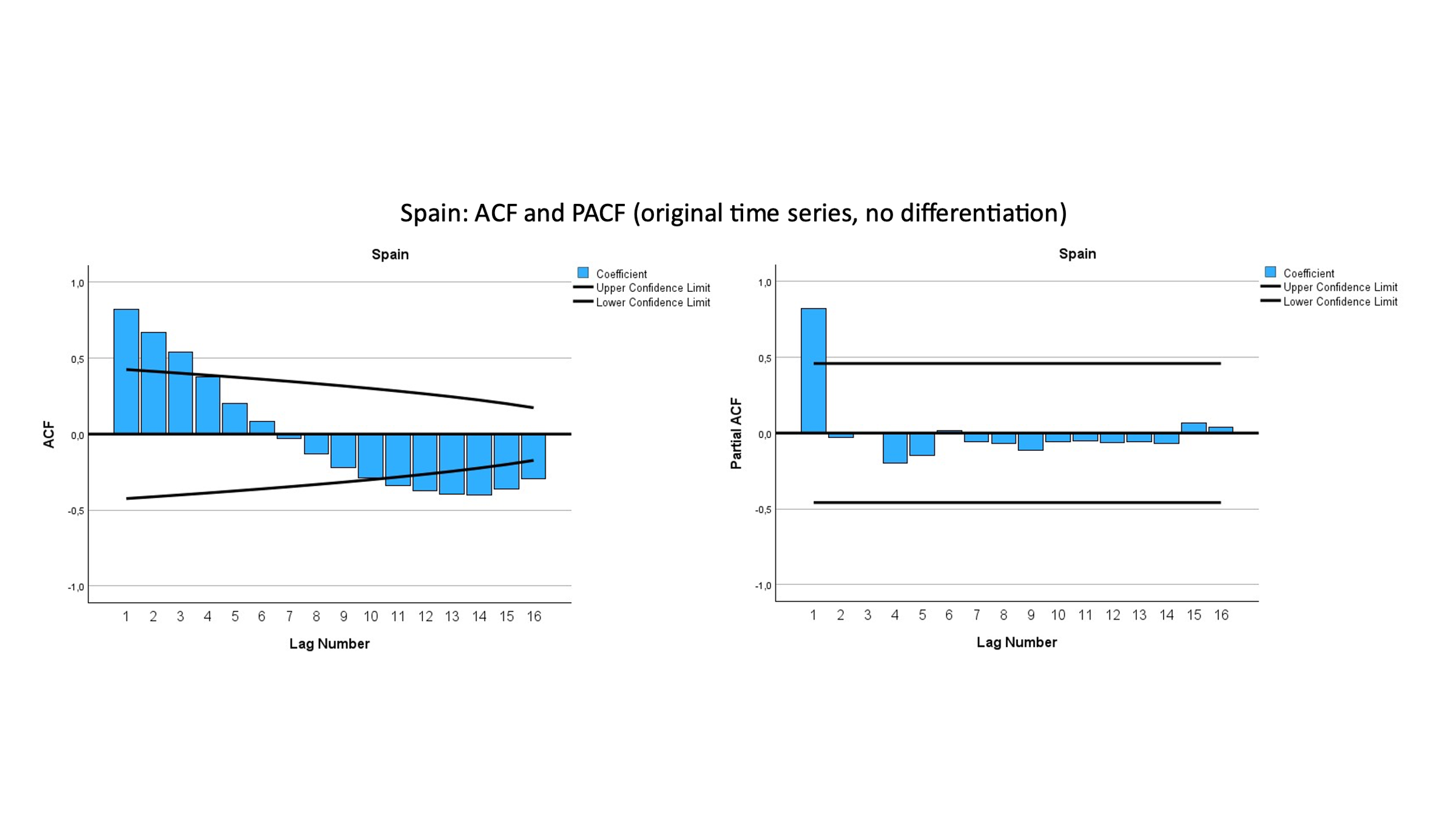


***Fig. S59:*** *Results of the autocorrelation, performed in SPSS. Depicted are the ACF and PACF plots for the original time series of Sweden.*


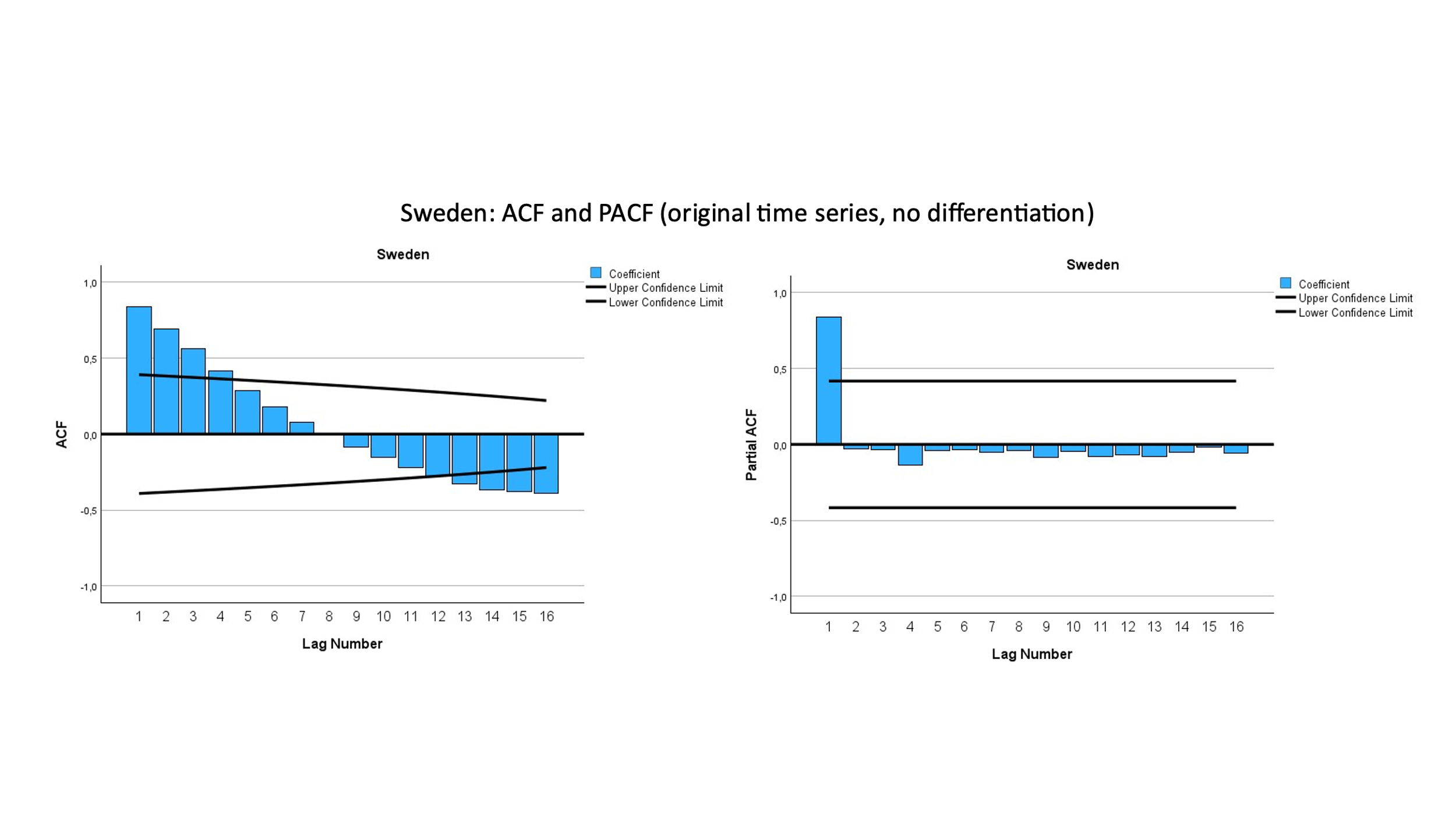


***Fig. S60:*** *Results of the autocorrelation, performed in SPSS. Depicted are the ACF and PACF plots for the original time series of Turkey.*


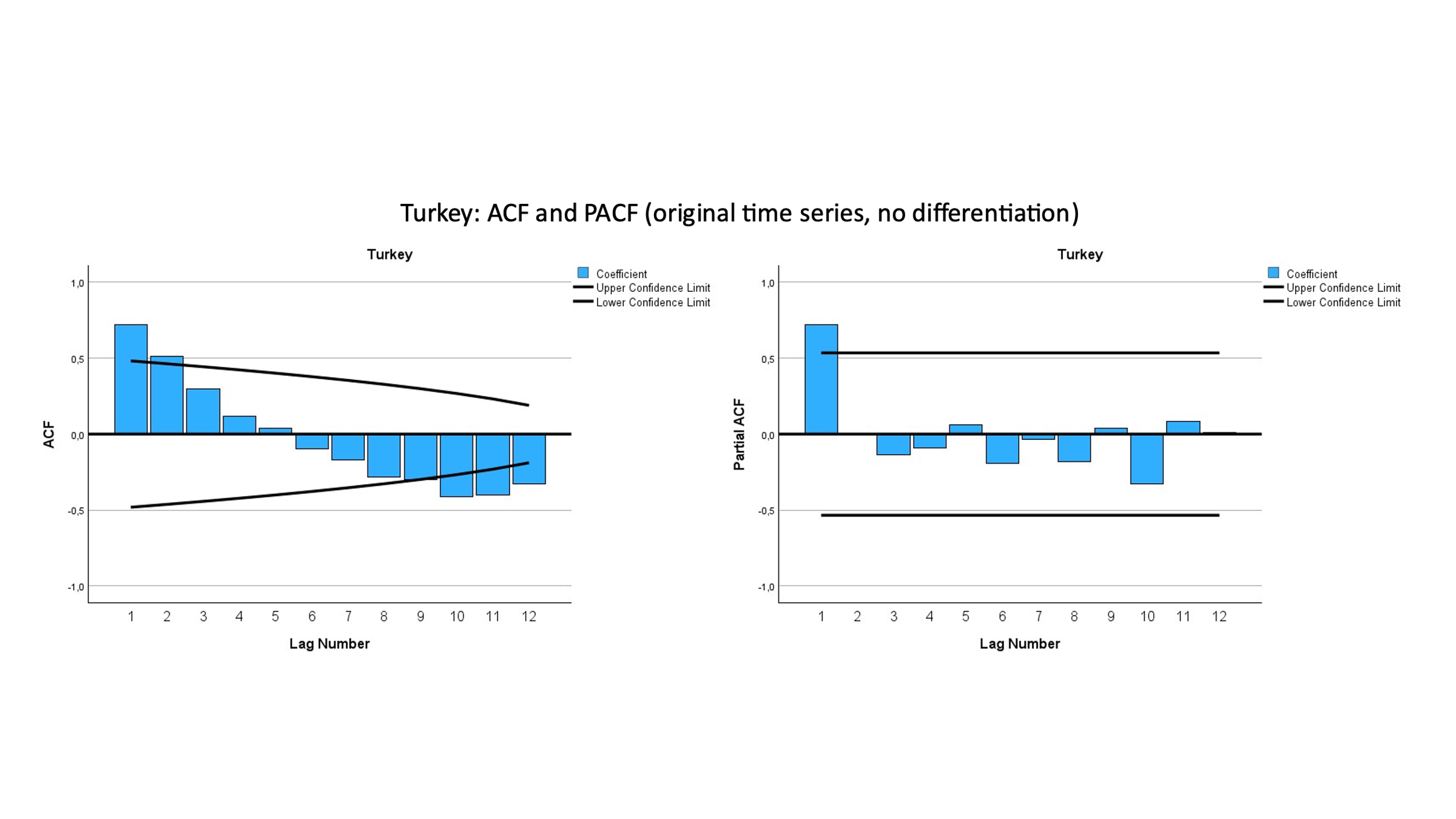


***Fig. S61:*** *Results of the autocorrelation, performed in SPSS. Depicted are the ACF and PACF plots for the original time series of the United Kingdom.*


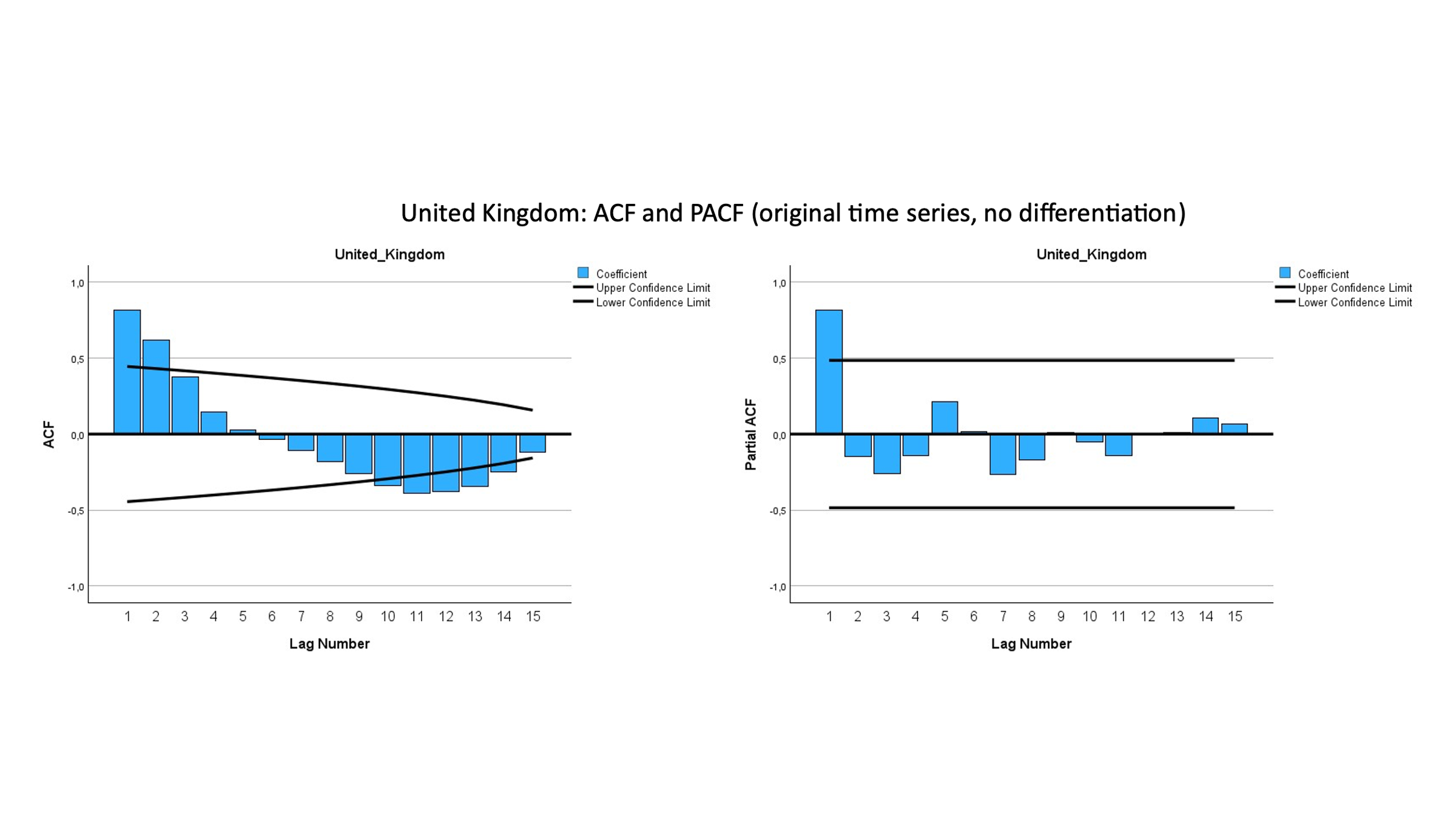


***Fig. S62:*** *Results of the autocorrelation, performed in SPSS. Depicted are the ACF and PACF plots for the original time series of Croatia.*


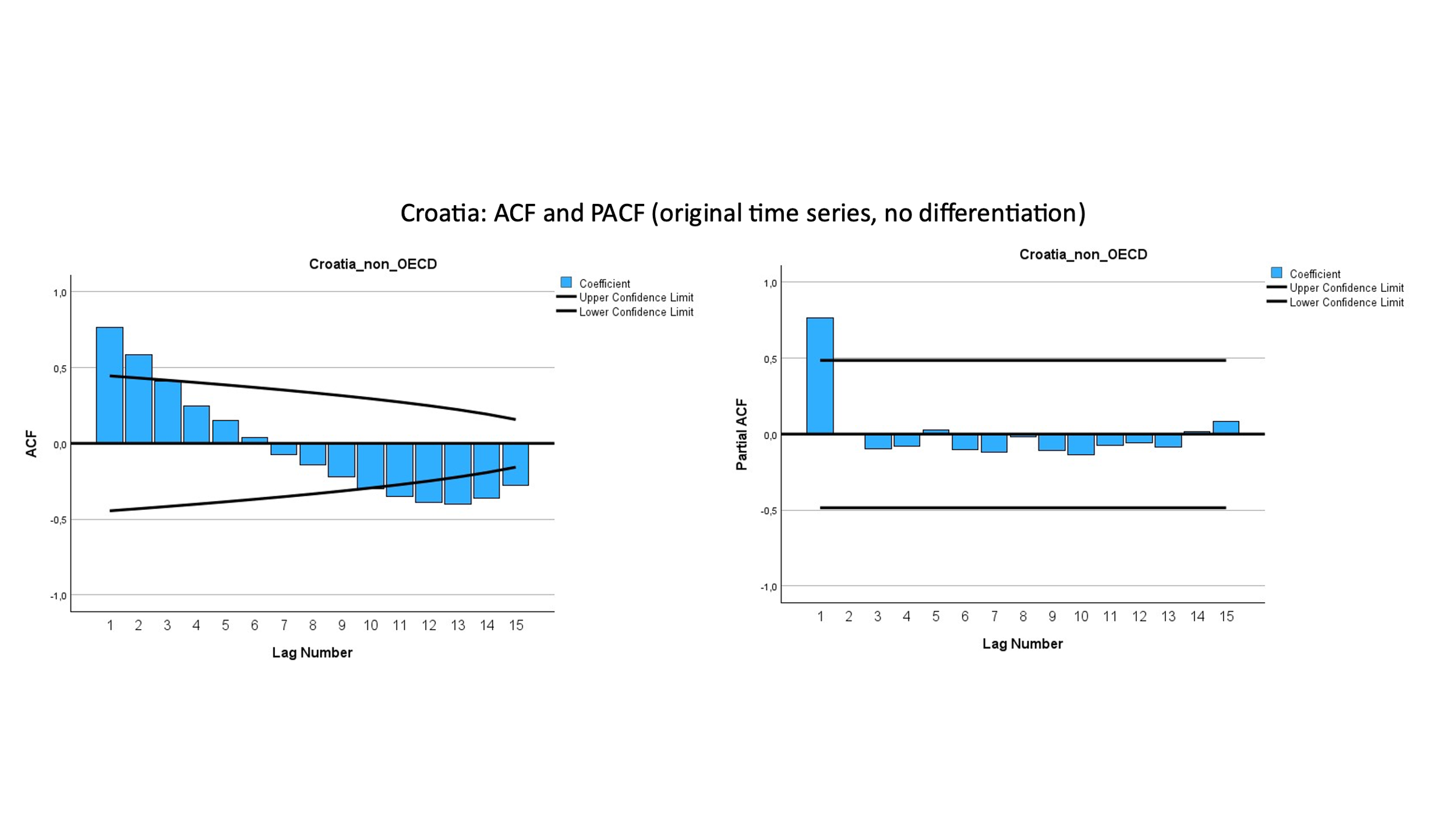


***Fig. S63:*** *Depicted are the ACF and PACF plots for the first-degree differentiation of the time series for Australia.*


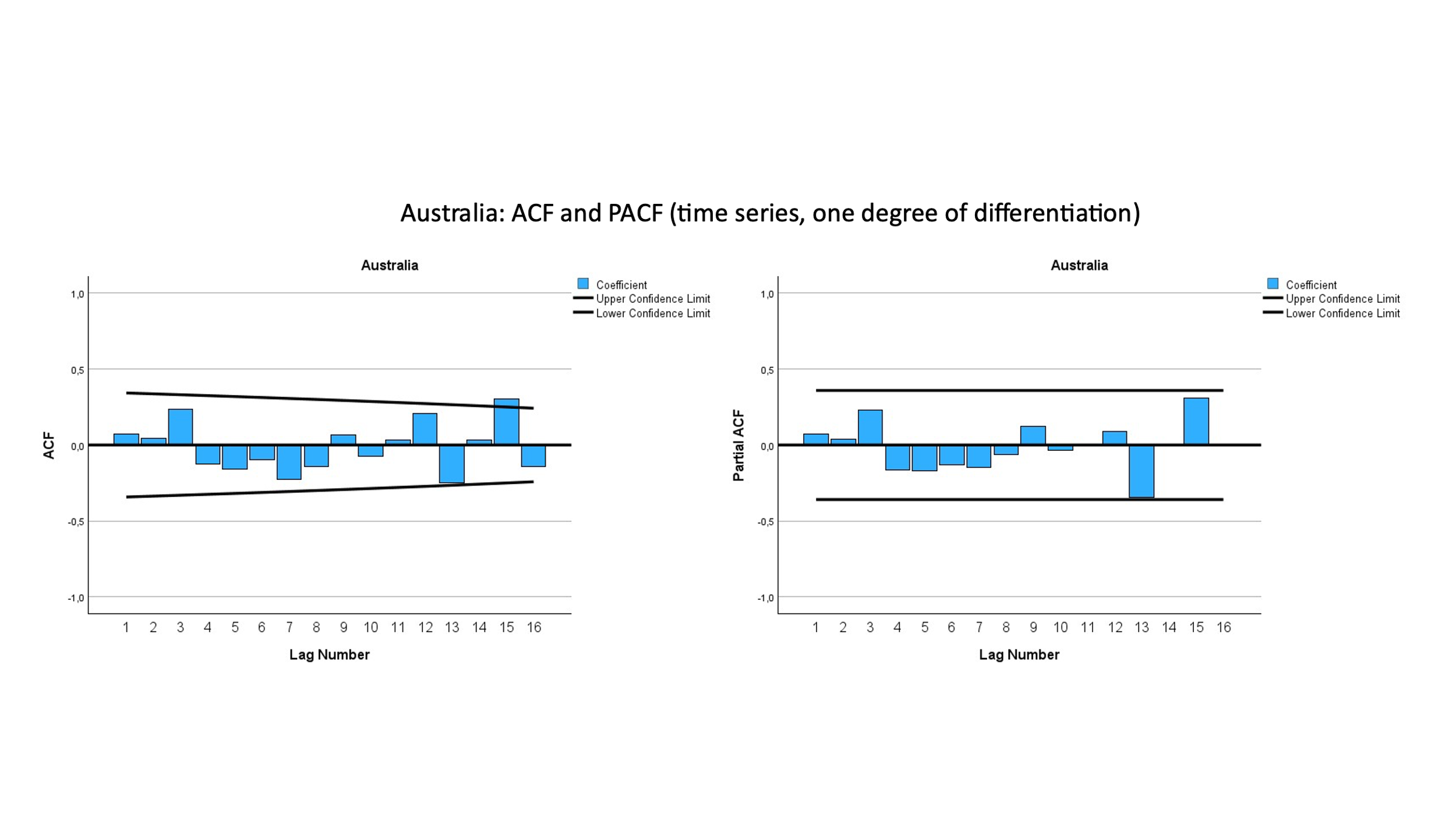


***Fig. S64:*** *Depicted are the ACF and PACF plots for the first-degree differentiation of the time series for Austria.*


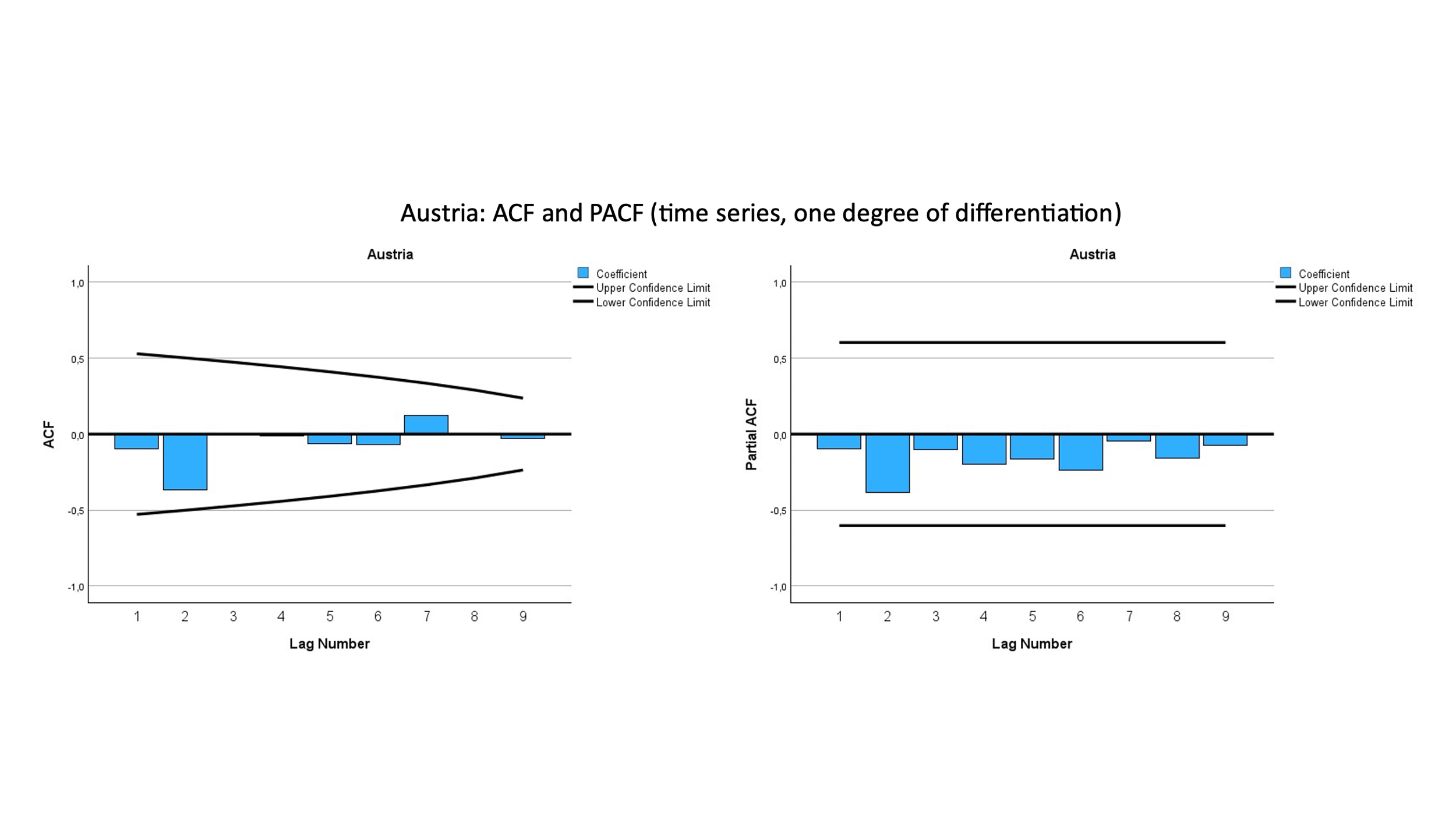


***Fig. S65:*** *Depicted are the ACF and PACF plots for the first-degree differentiation of the time series for Belgium.*


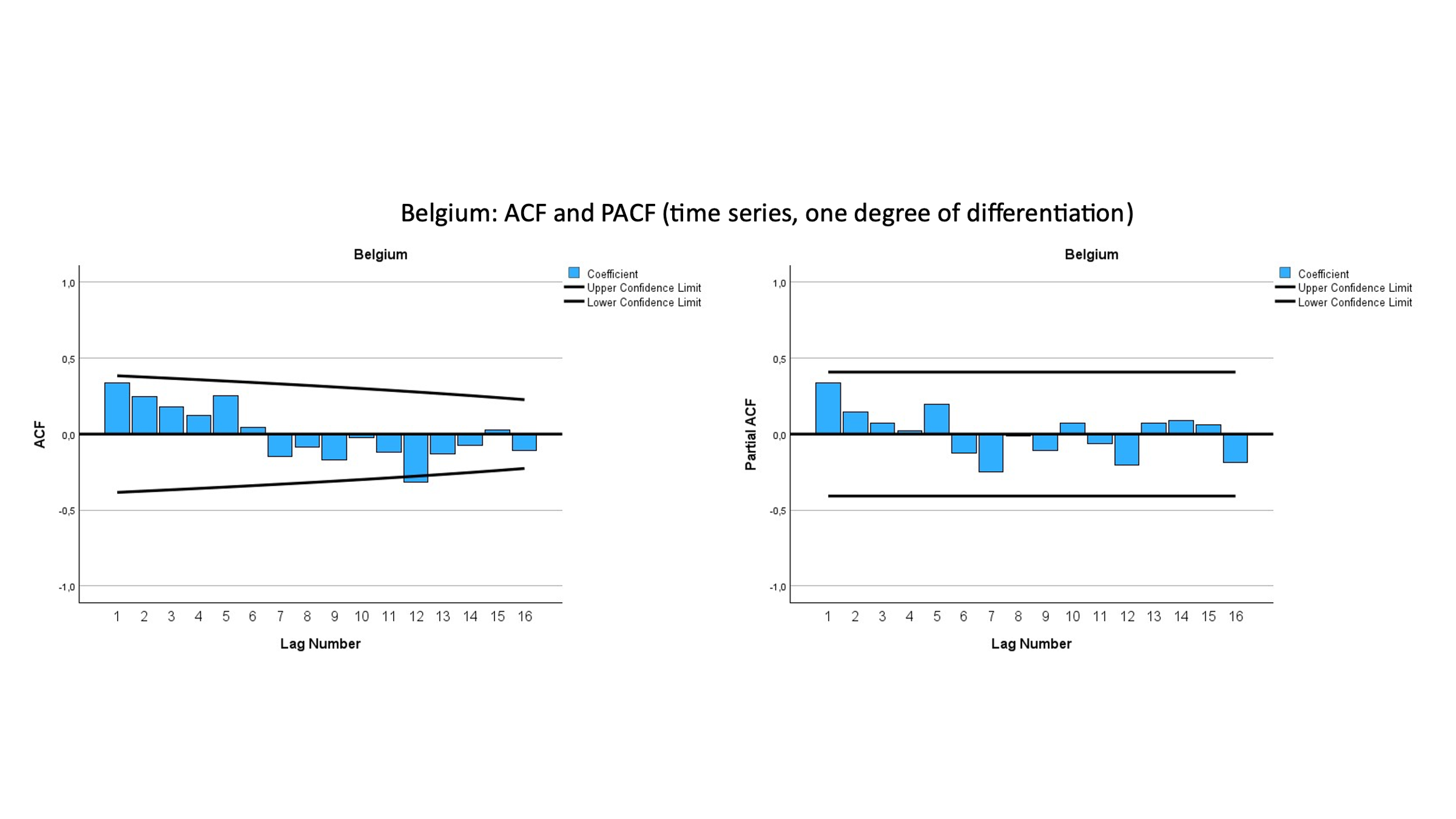


***Fig. S66:*** *Depicted are the ACF and PACF plots for the first-degree differentiation of the time series for Canada.*


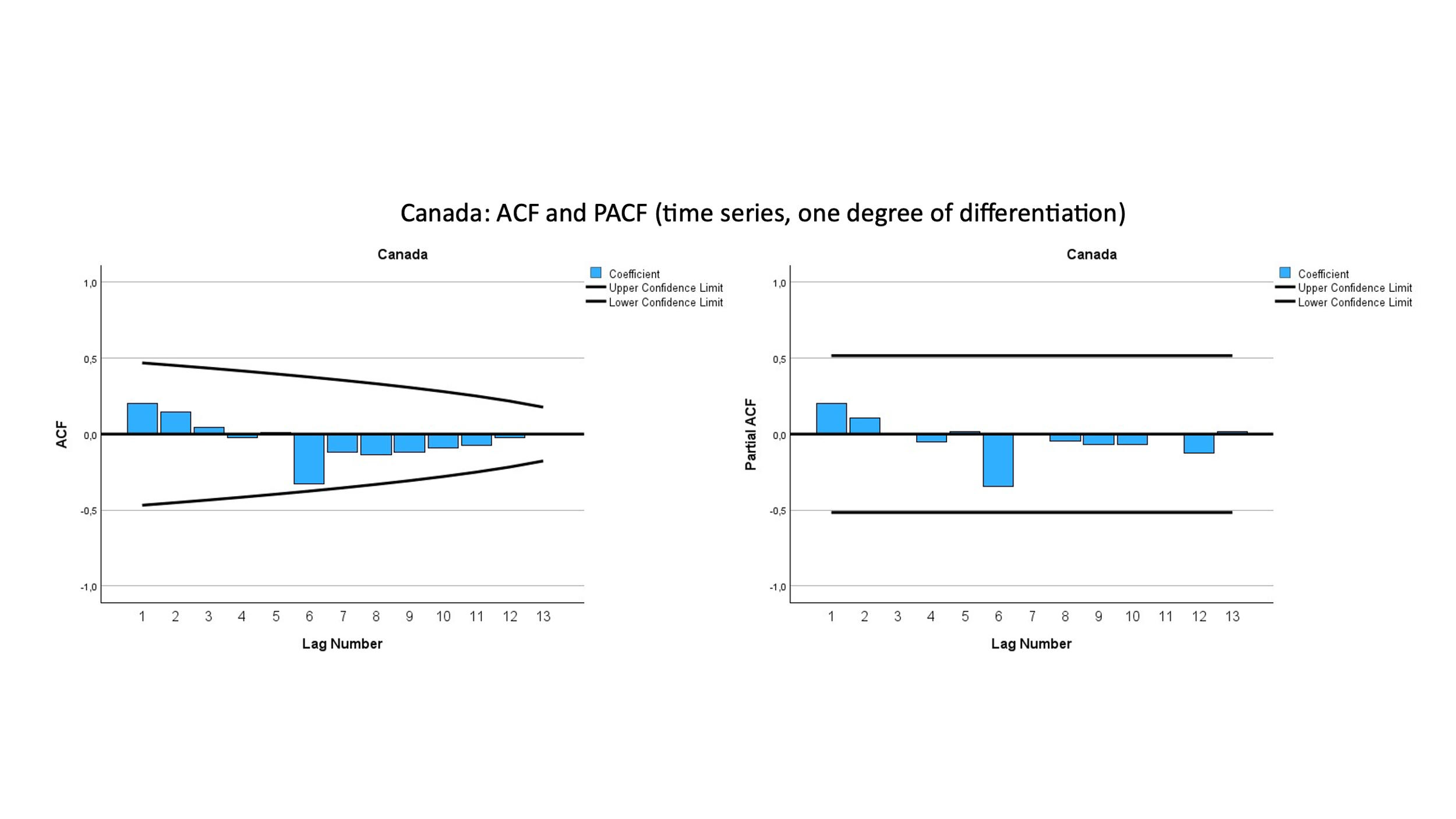


***Fig. S67:*** *Depicted are the ACF and PACF plots for the first-degree differentiation of the time series for Chile.*


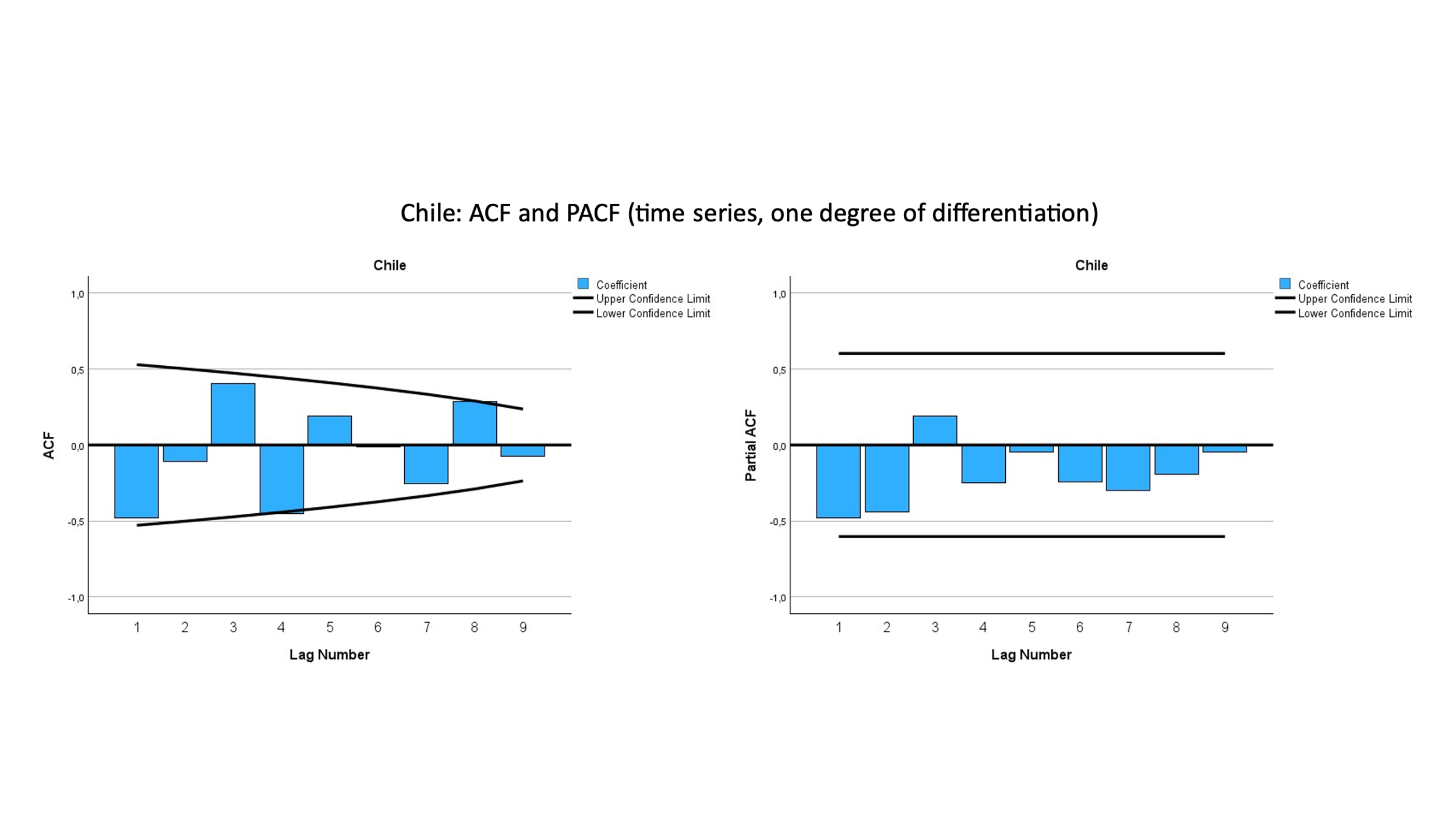


***Fig. S68:*** *Depicted are the ACF and PACF plots for the first-degree differentiation of the time series for Costa Rica.*


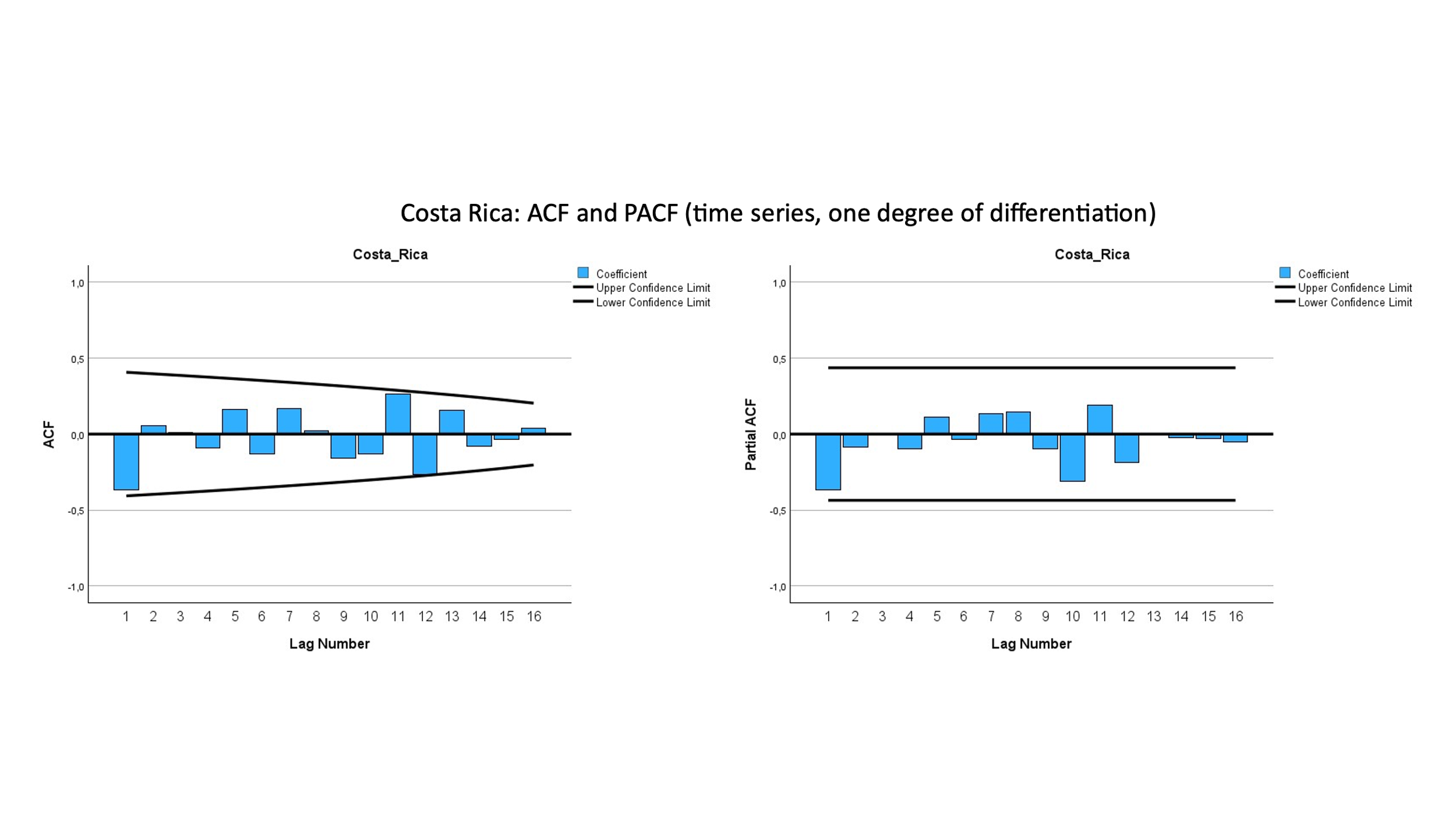


***Fig. S69:*** *Depicted are the ACF and PACF plots for the first-degree differentiation of the time series for Czechia.*


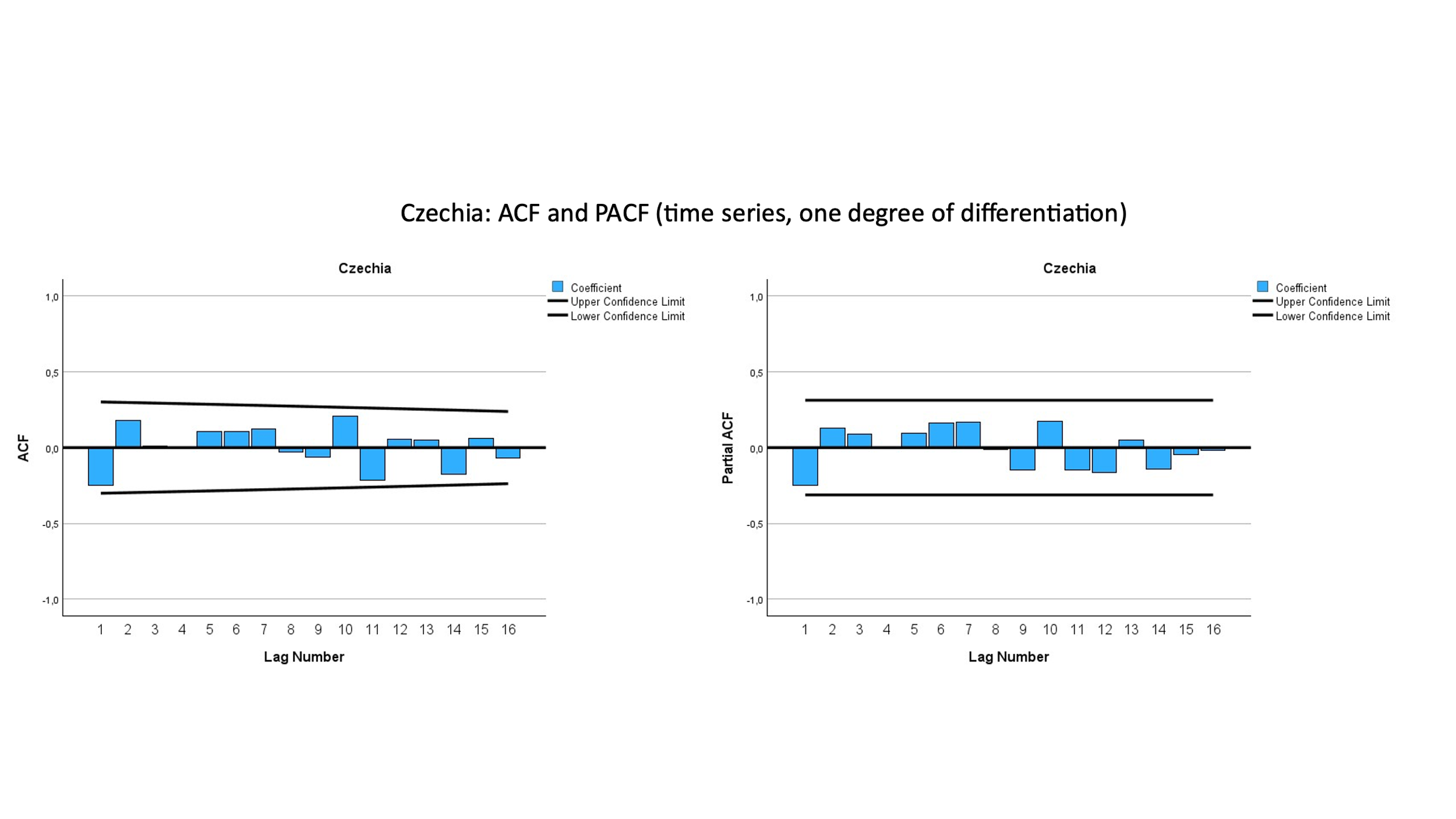


***Fig. S70:*** *Depicted are the ACF and PACF plots for the first-degree differentiation of the time series for Denmark.*


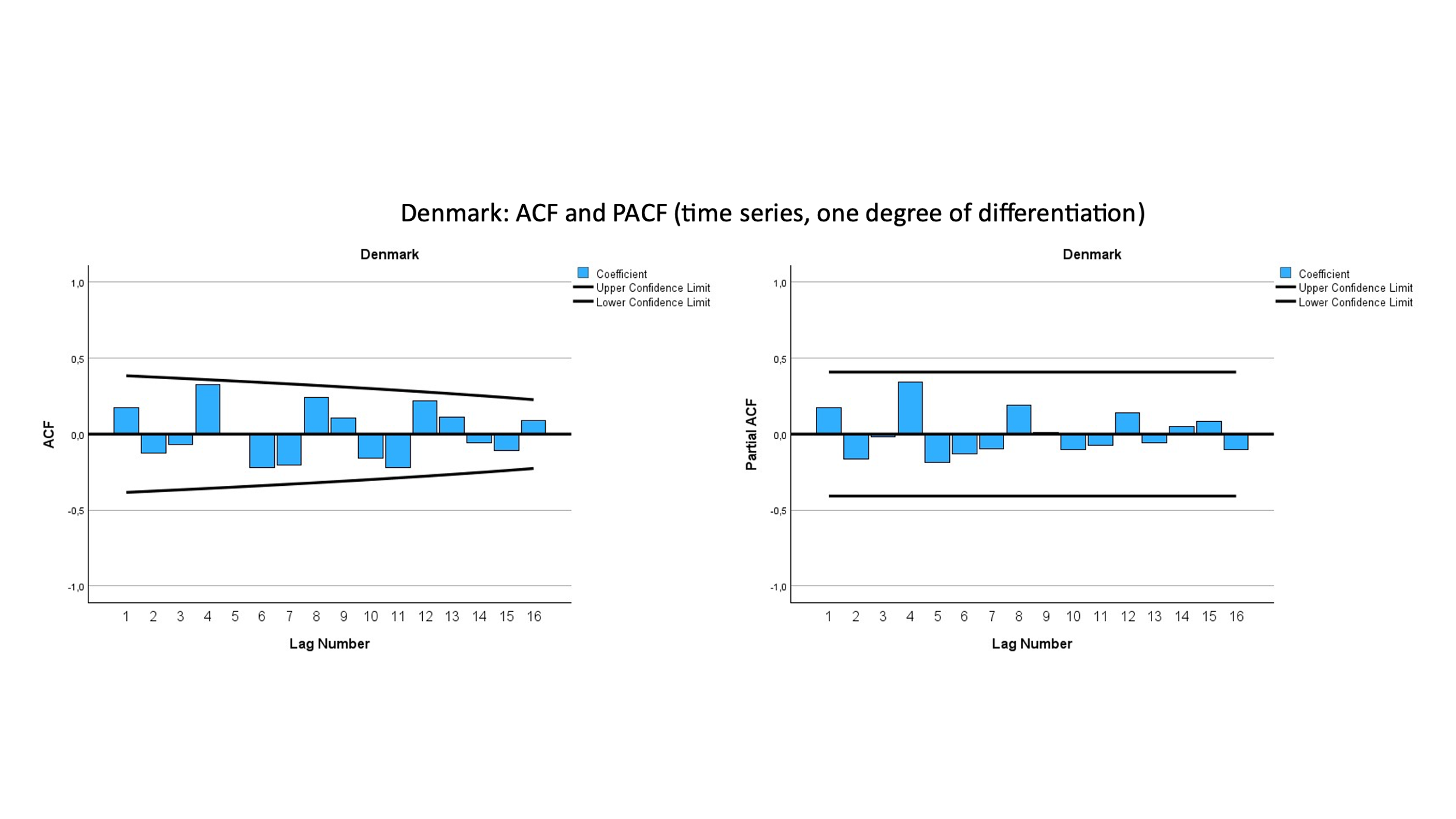


***Fig. S71:*** *Depicted are the ACF and PACF plots for the first-degree differentiation of the time series for Iceland.*


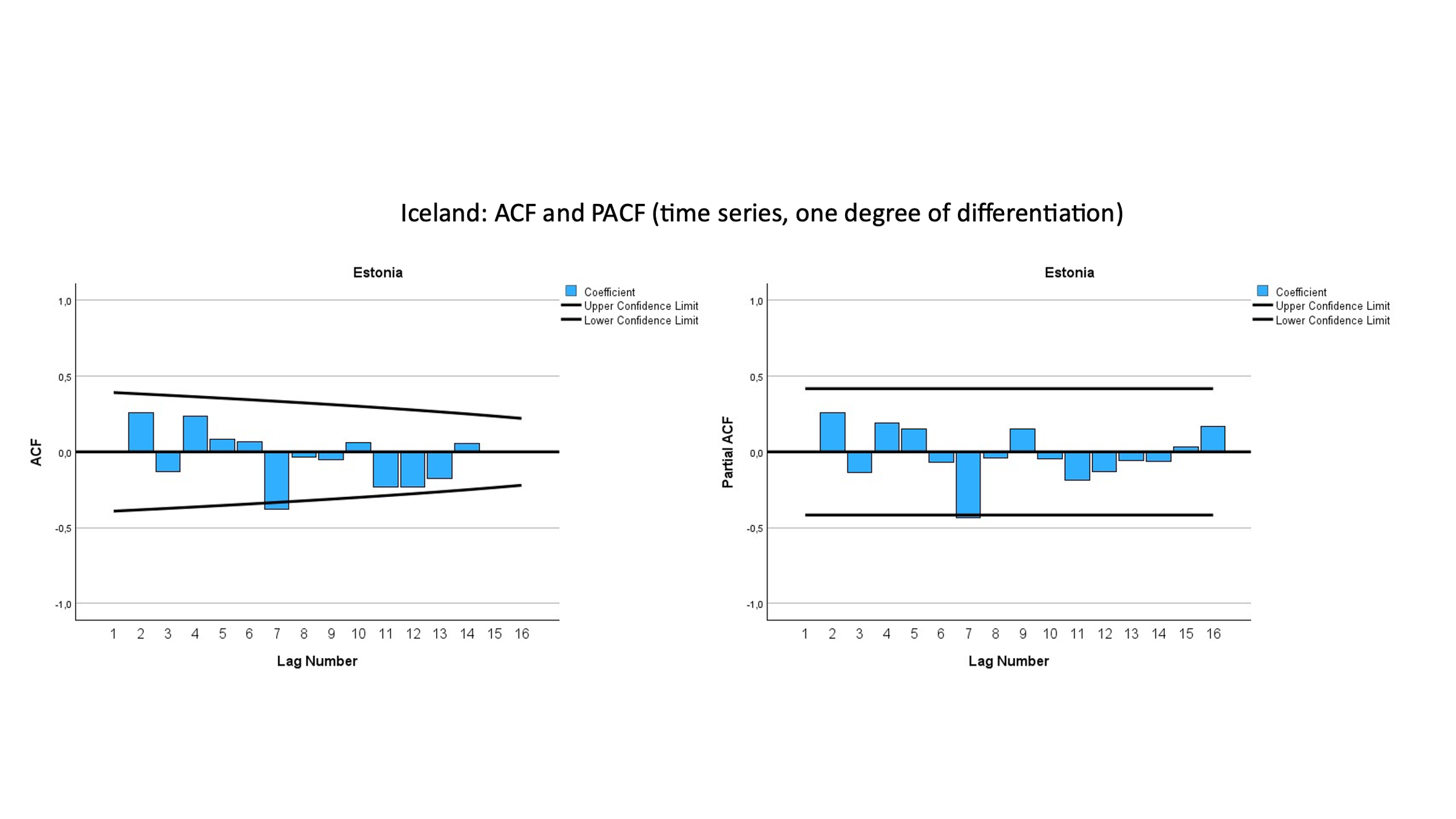


***Fig. S72:*** *Depicted are the ACF and PACF plots for the first-degree differentiation of the time series for Finland.*


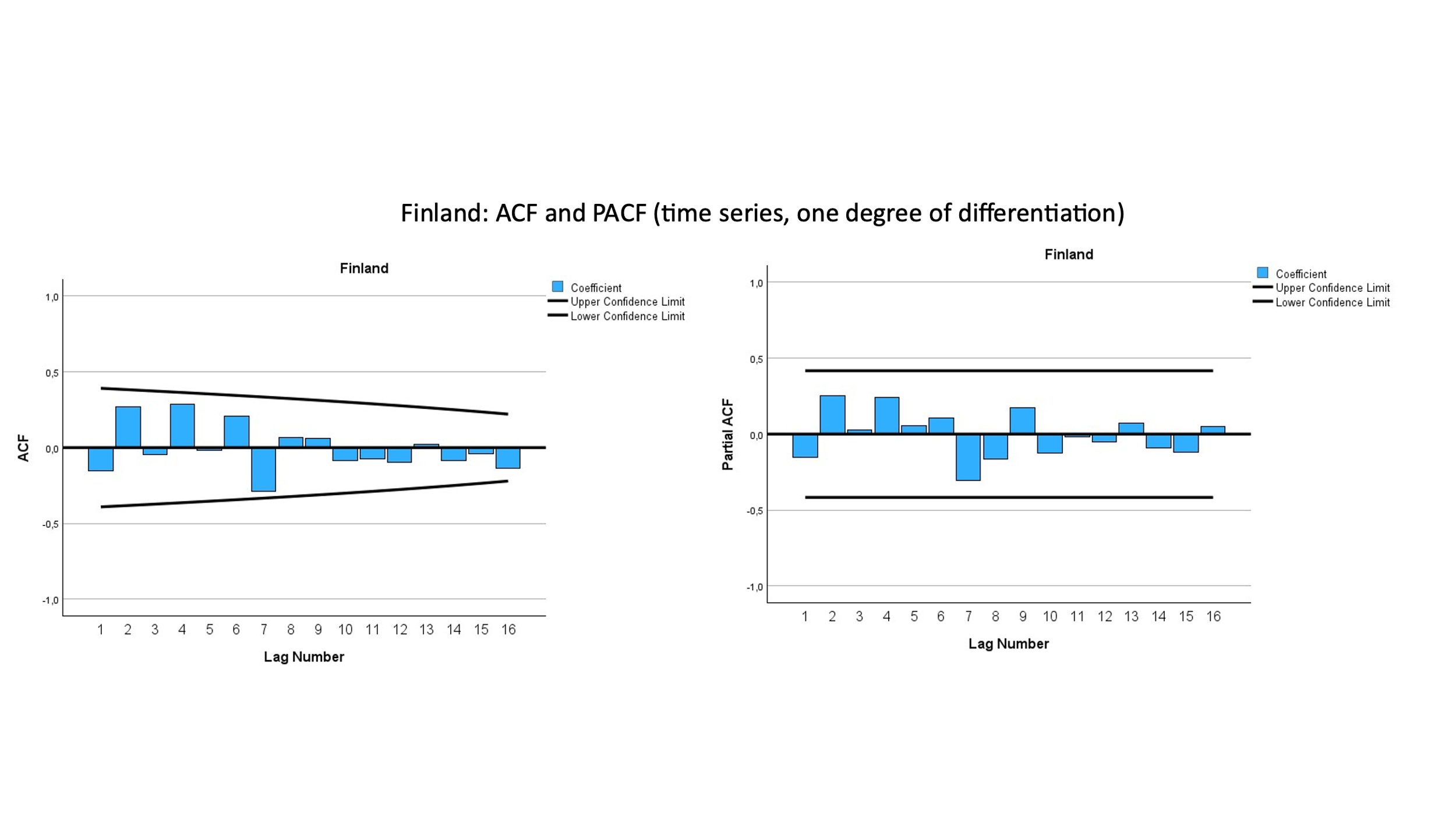


***Fig. S73:*** *Depicted are the ACF and PACF plots for the first-degree differentiation of the time series for Iceland.*


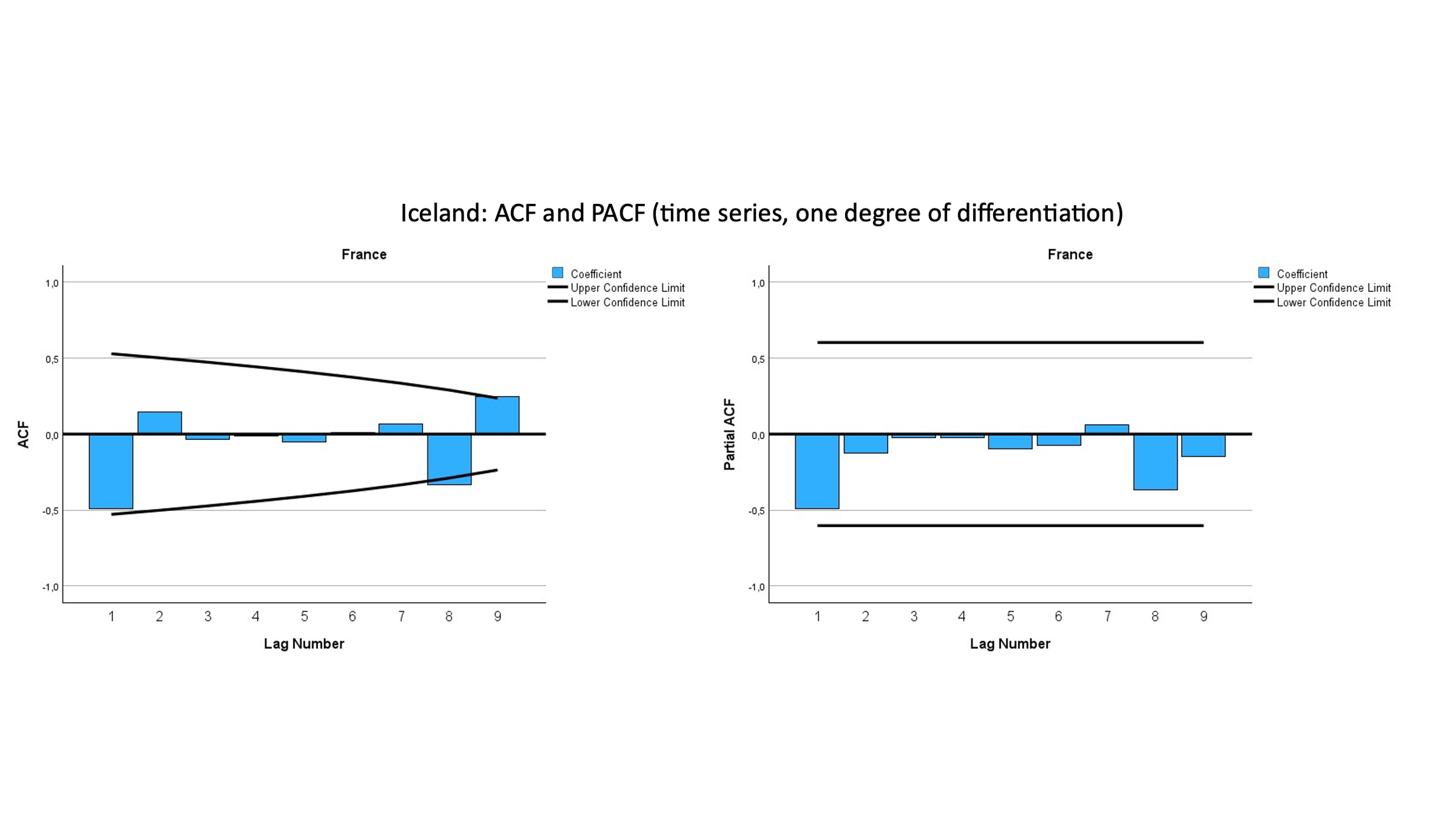


***Fig. S74:*** *Depicted are the ACF and PACF plots for the first-degree differentiation of the time series for Germany.*


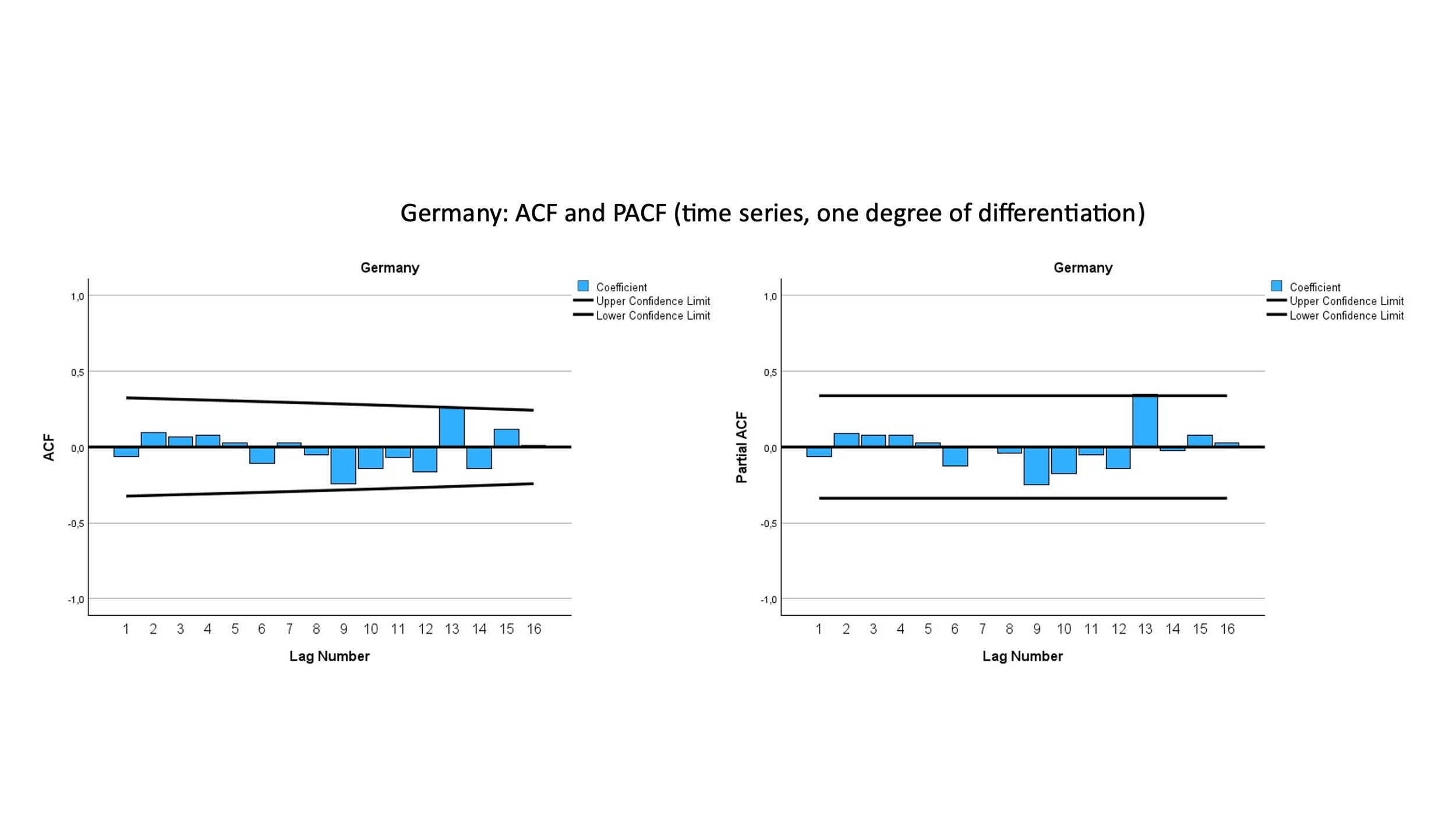


***Fig. S75:*** *Depicted are the ACF and PACF plots for the first-degree differentiation of the time series for Greece.*


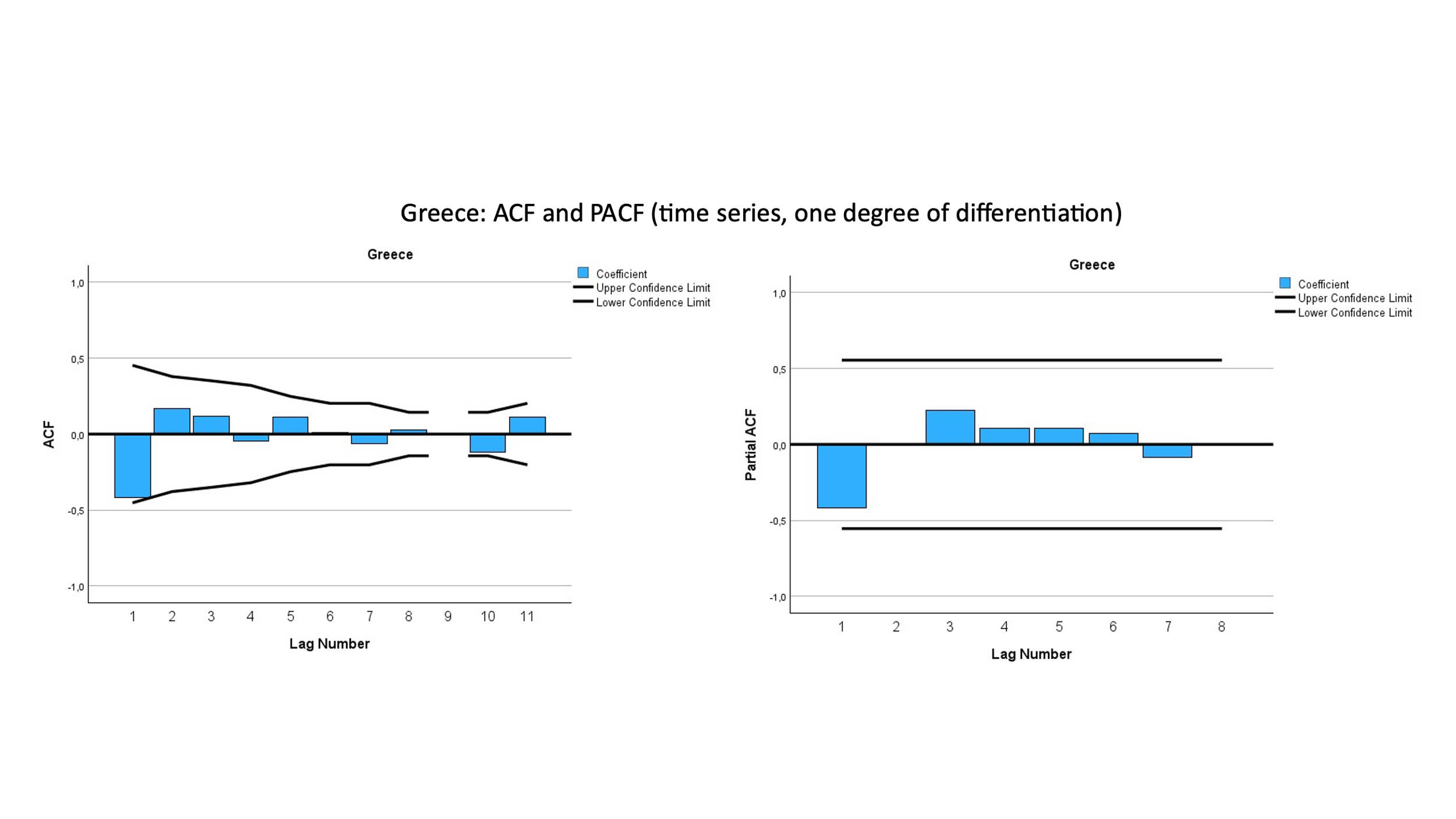


***Fig. S76:*** *Depicted are the ACF and PACF plots for the first-degree differentiation of the time series for Hungary.*


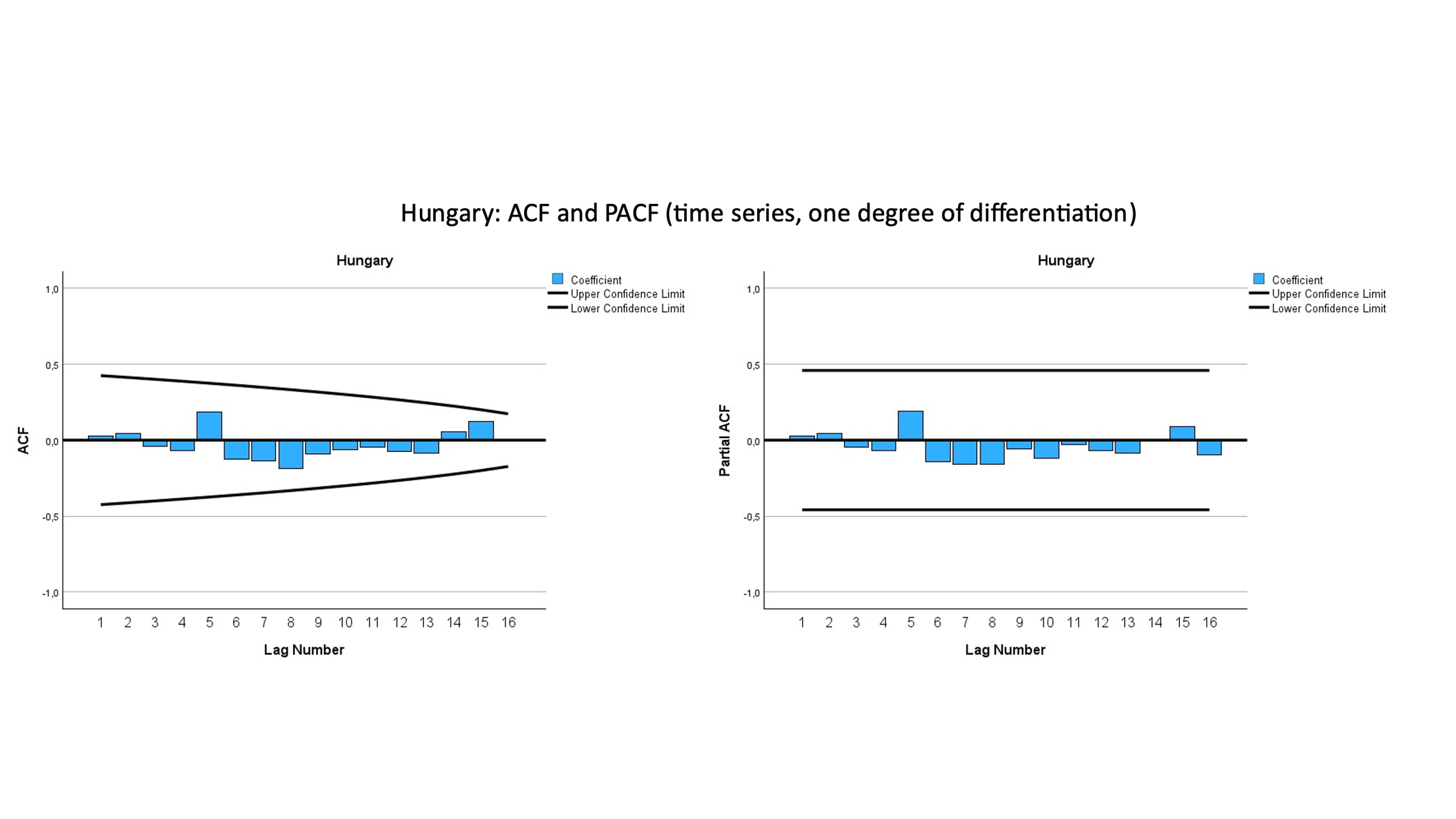


***Fig. S77:*** *Depicted are the ACF and PACF plots for the first-degree differentiation of the time series for Iceland.*


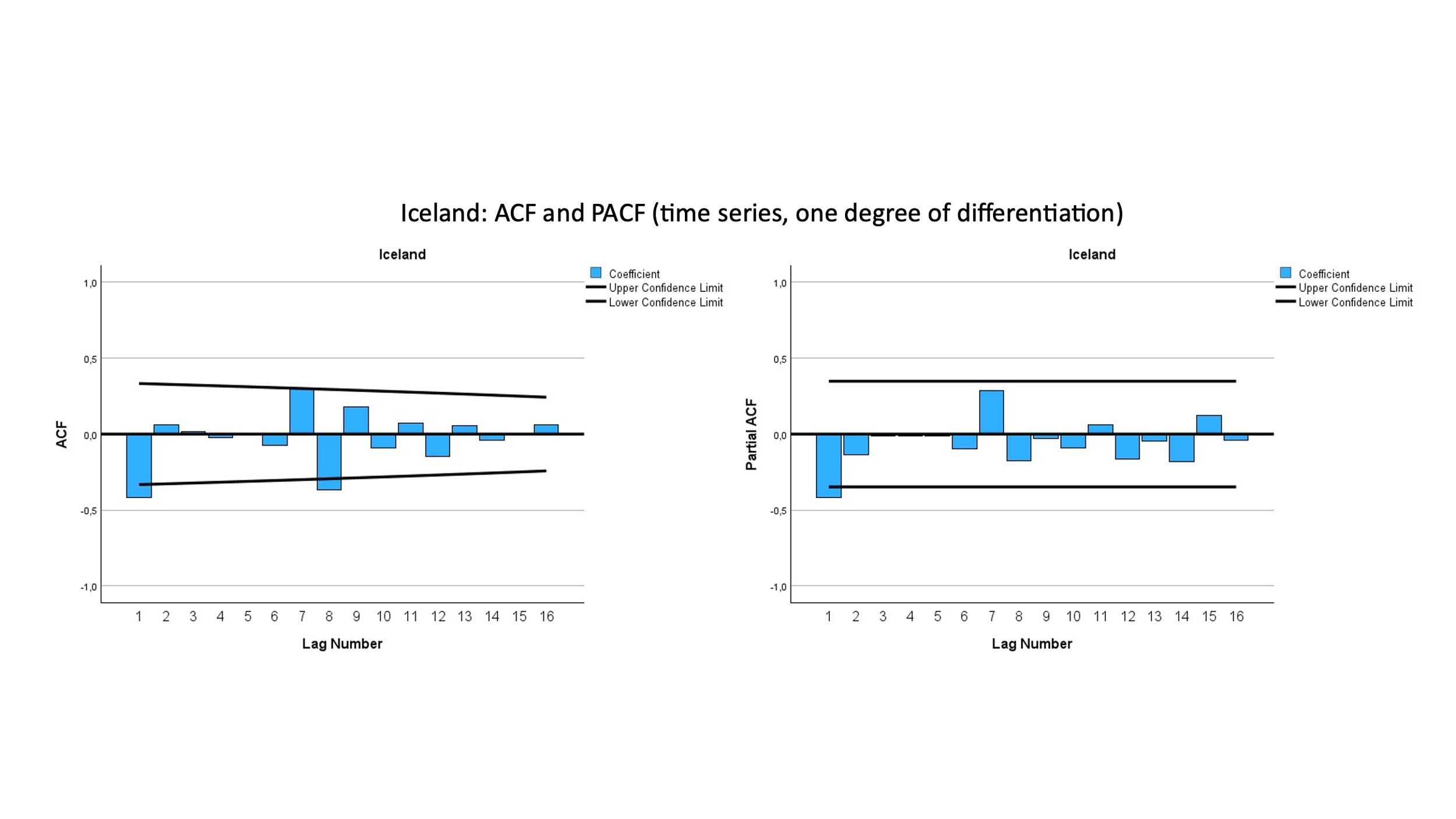


***Fig. S78:*** *Depicted are the ACF and PACF plots for the first-degree differentiation of the time series for Israel.*


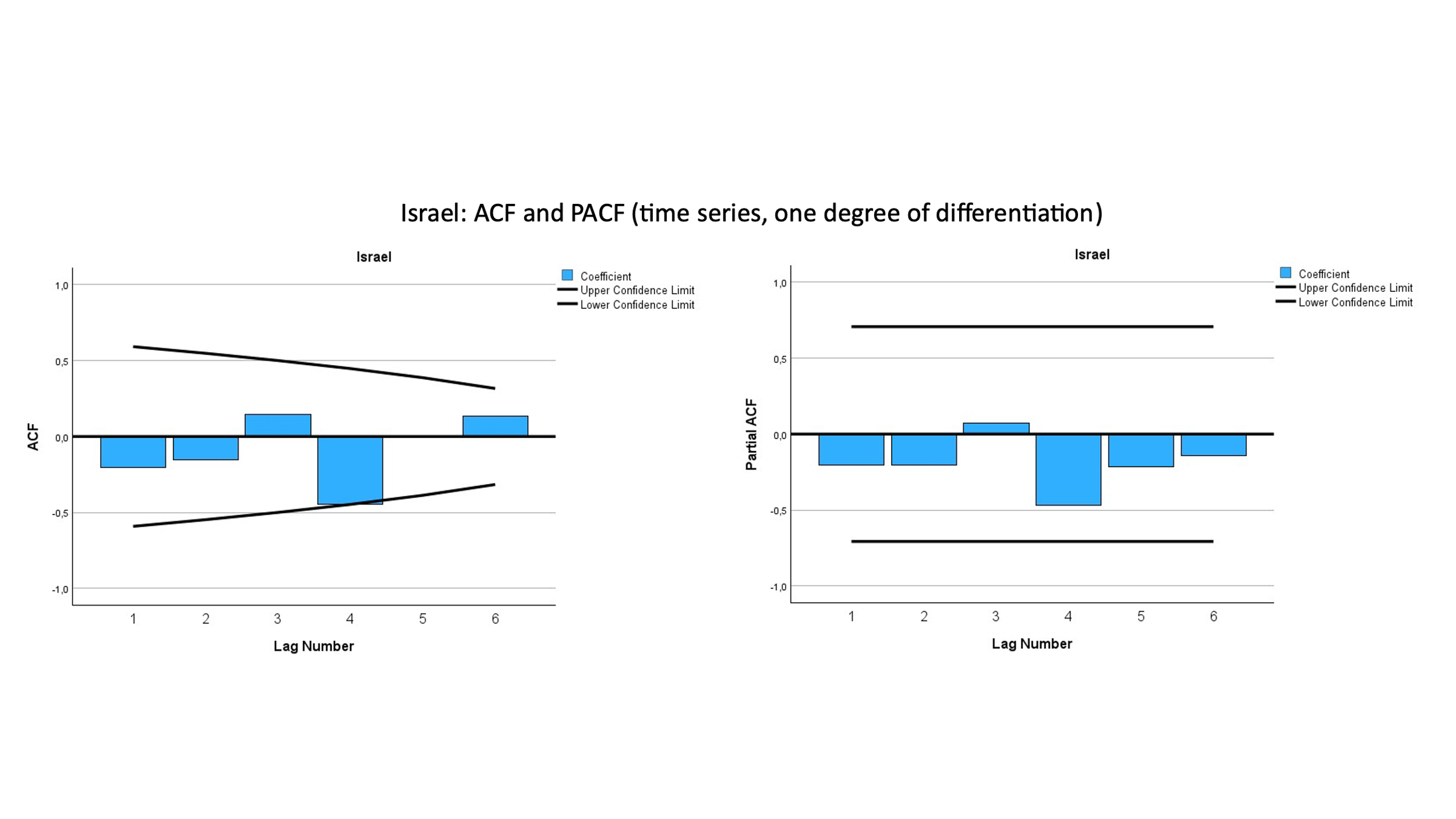


***Fig. S79:*** *Depicted are the ACF and PACF plots for the first-degree differentiation of the time series for Italy.*


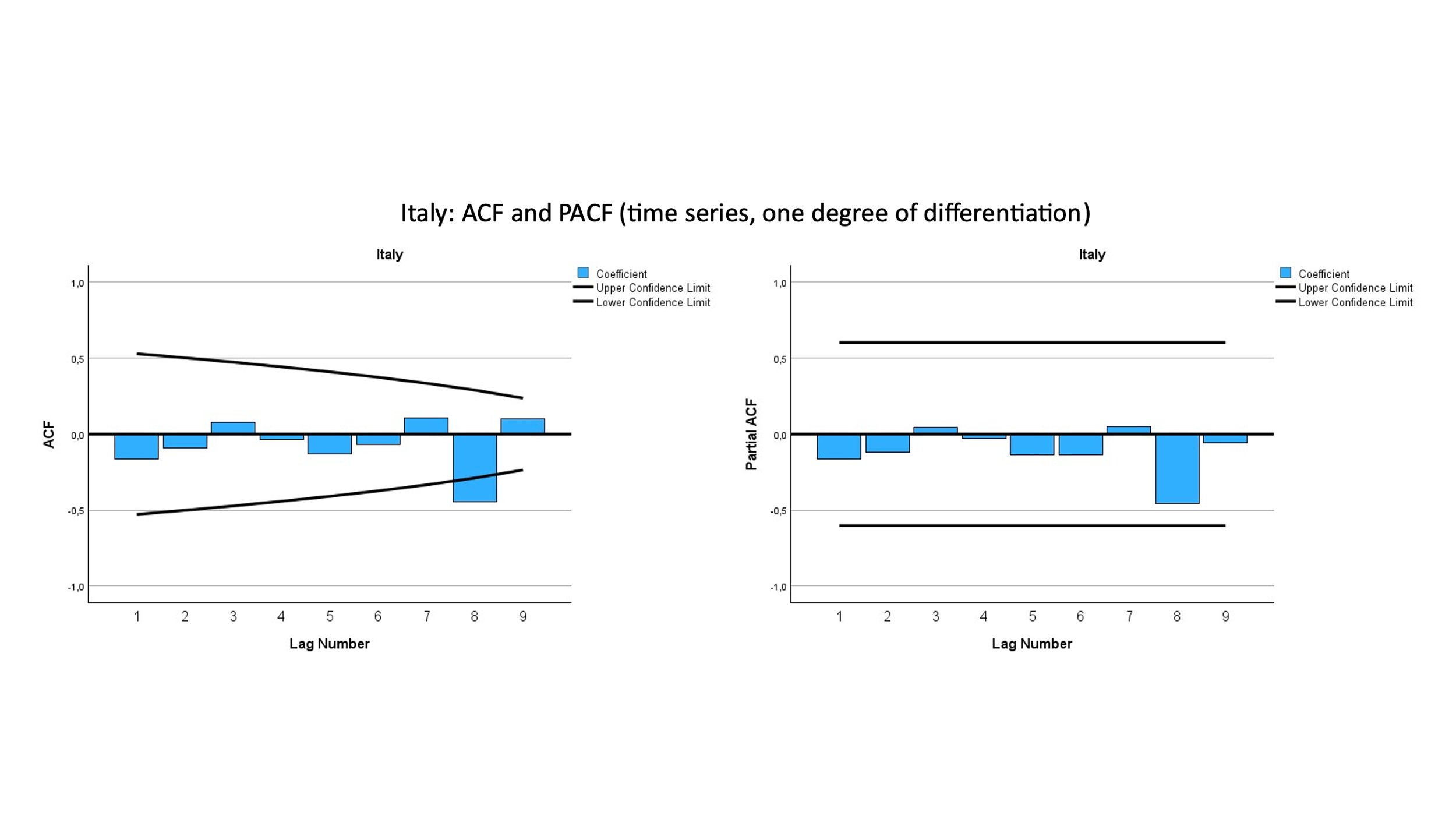


***Fig. S80:*** *Depicted are the ACF and PACF plots for the first-degree differentiation of the time series for Korea.*


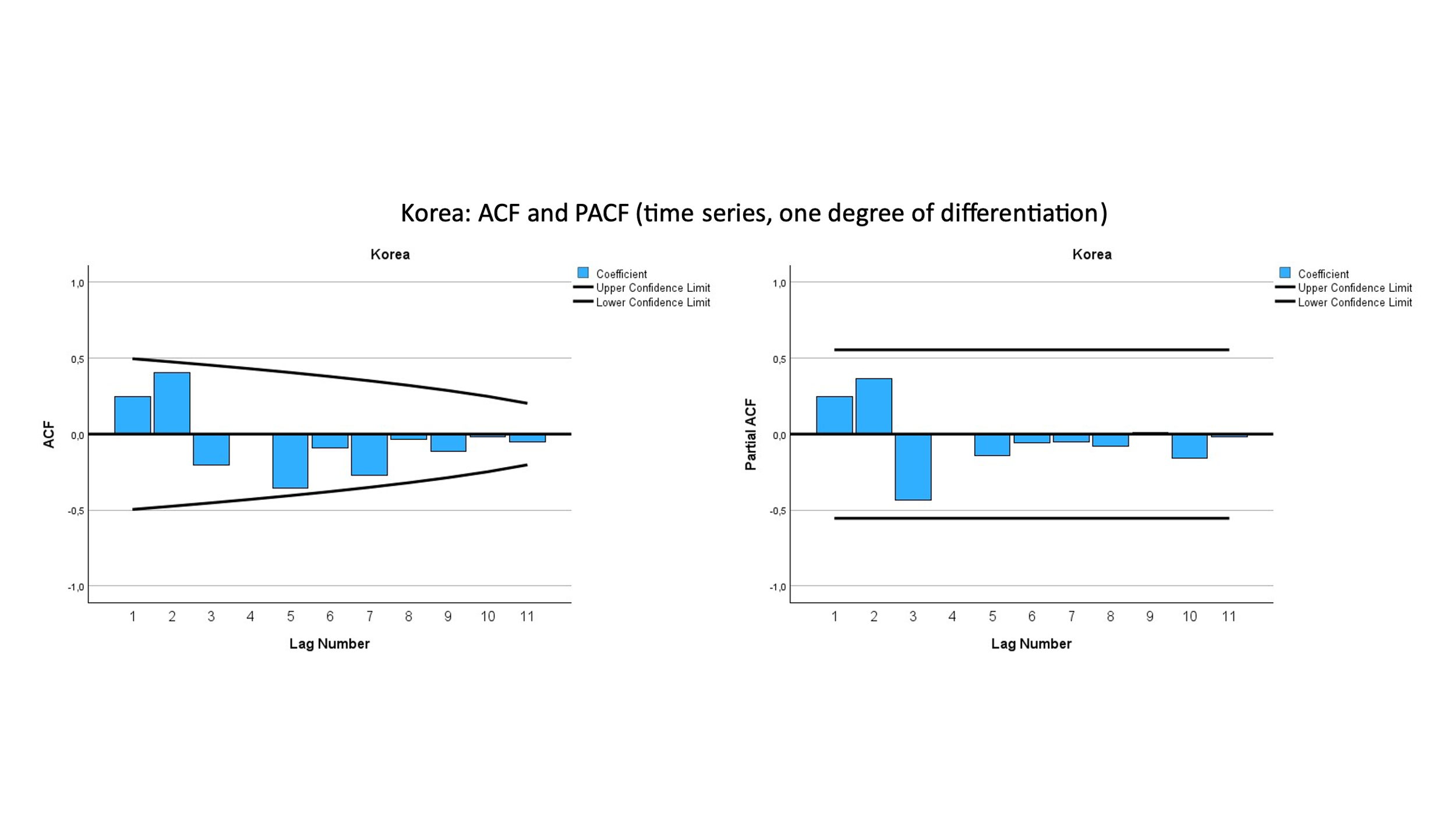


***Fig. S81:*** *Depicted are the ACF and PACF plots for the first-degree differentiation of the time series for Latvia.*


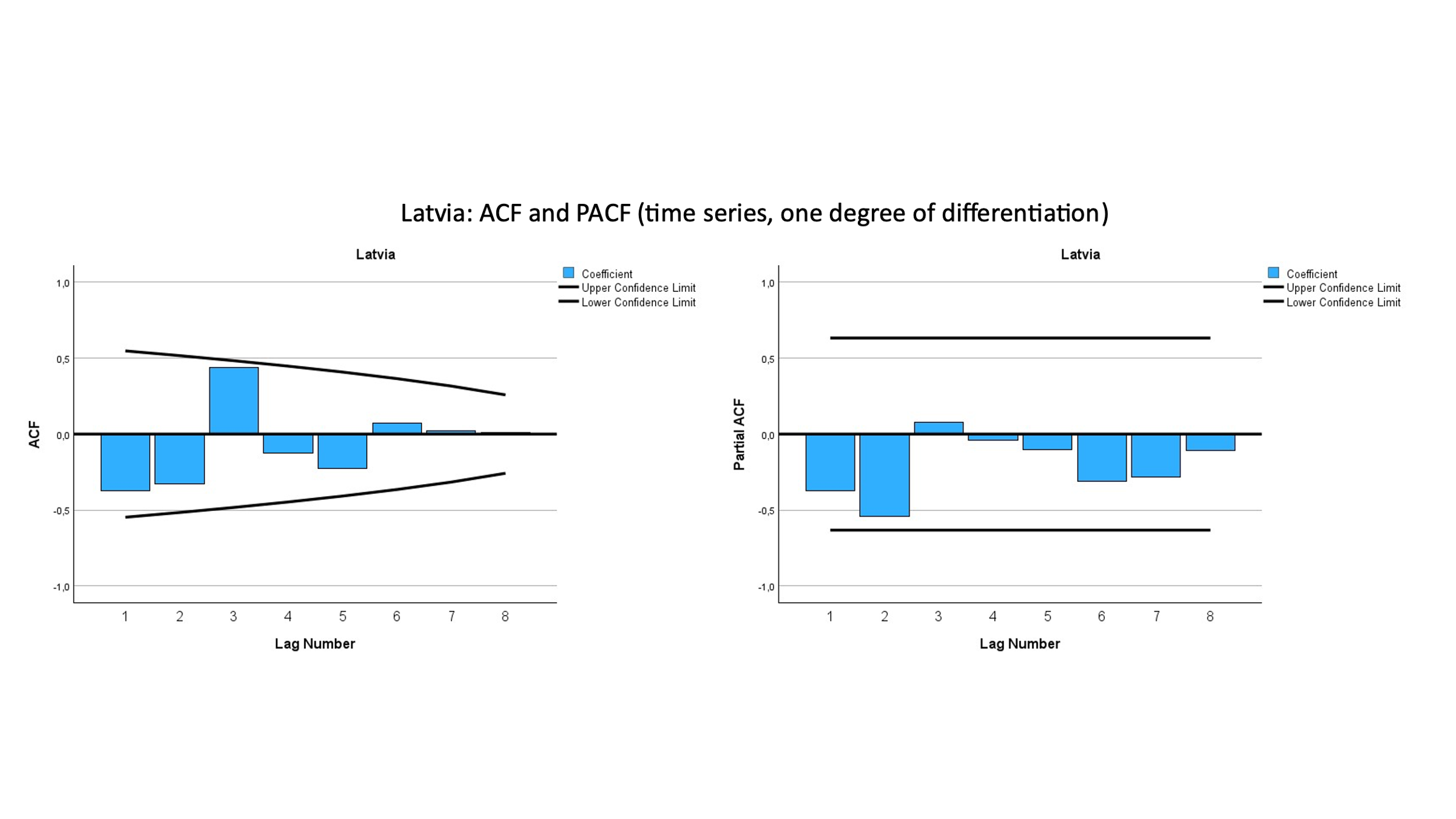


***Fig. S82:*** *Depicted are the ACF and PACF plots for the first-degree differentiation of the time series for Lithuania.*


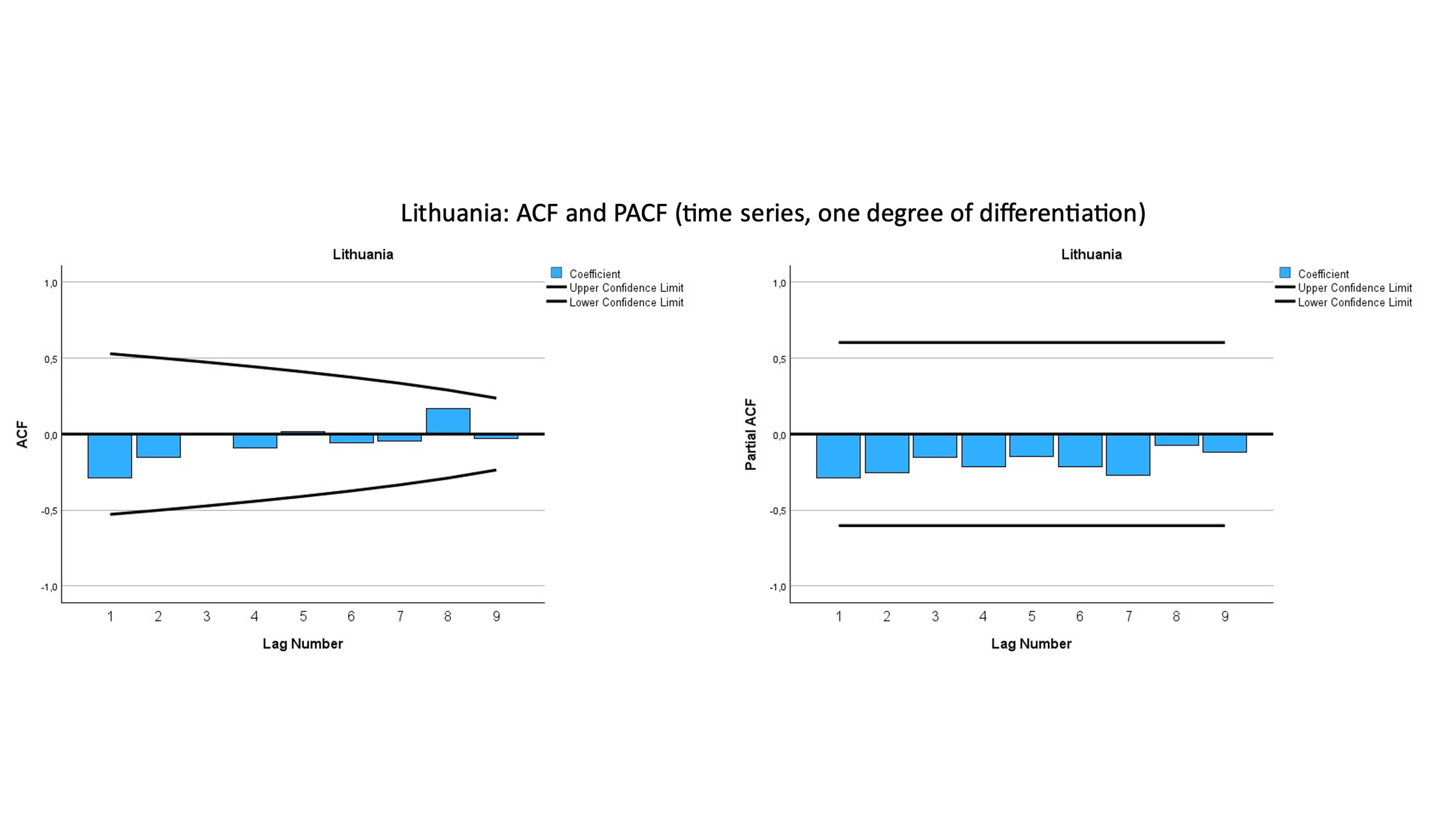


***Fig. S83:*** *Depicted are the ACF and PACF plots for the first-degree differentiation of the time series for Luxembourg.*


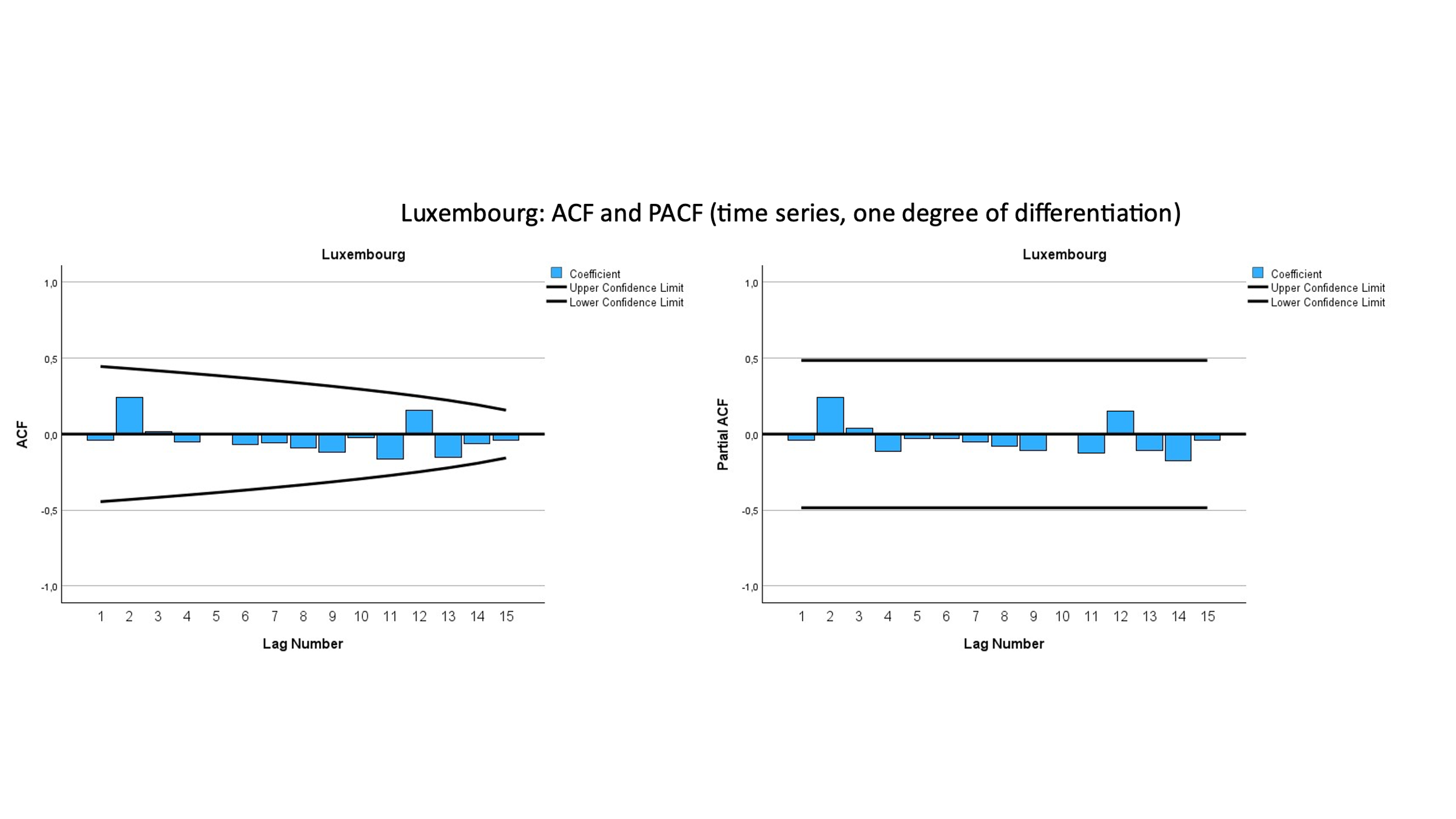


***Fig. S84:*** *Depicted are the ACF and PACF plots for the first-degree differentiation of the time series for the Netherlands.*


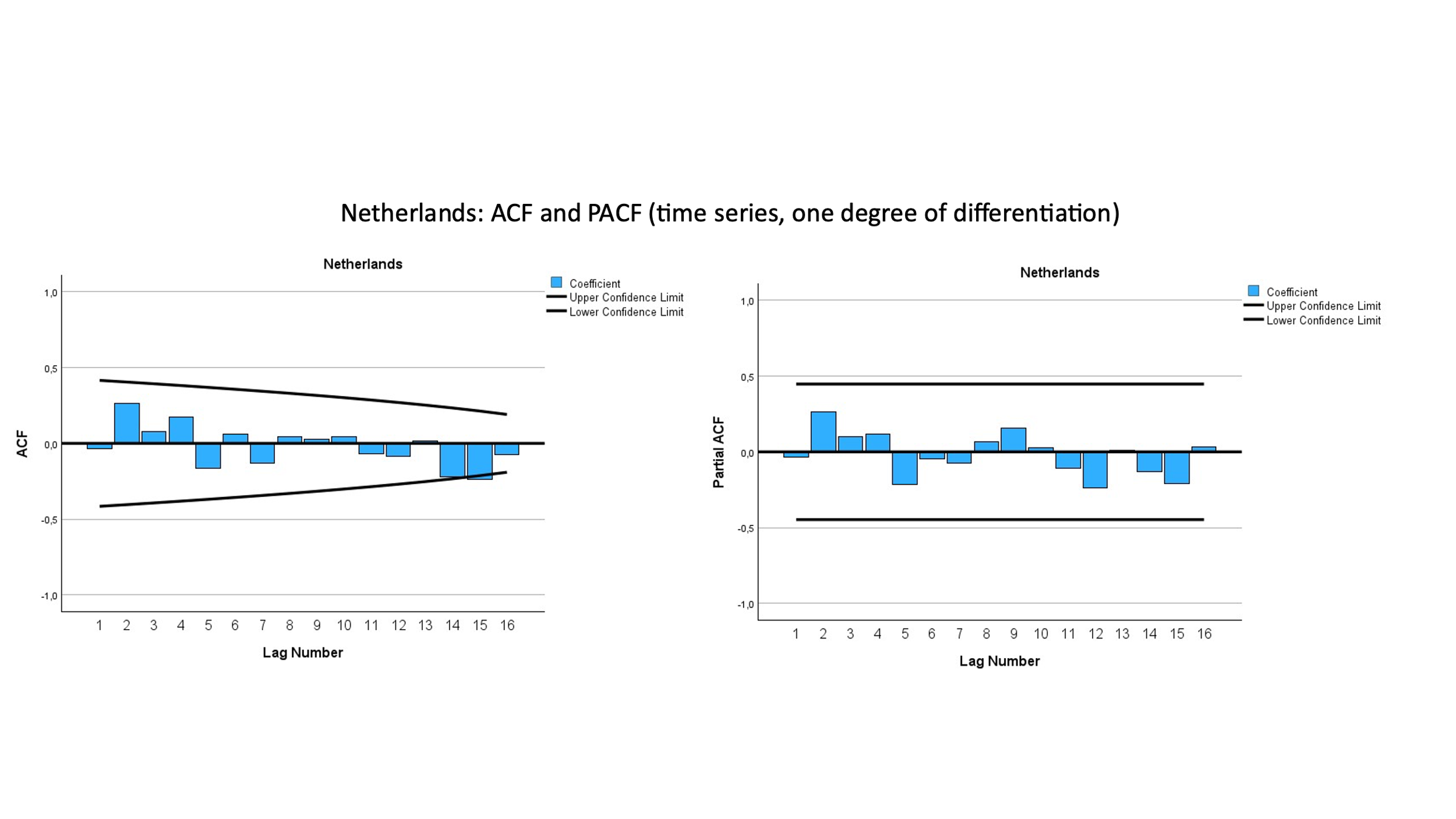


***Fig. S85:*** *Depicted are the ACF and PACF plots for the first-degree differentiation of the time series for Norway.*


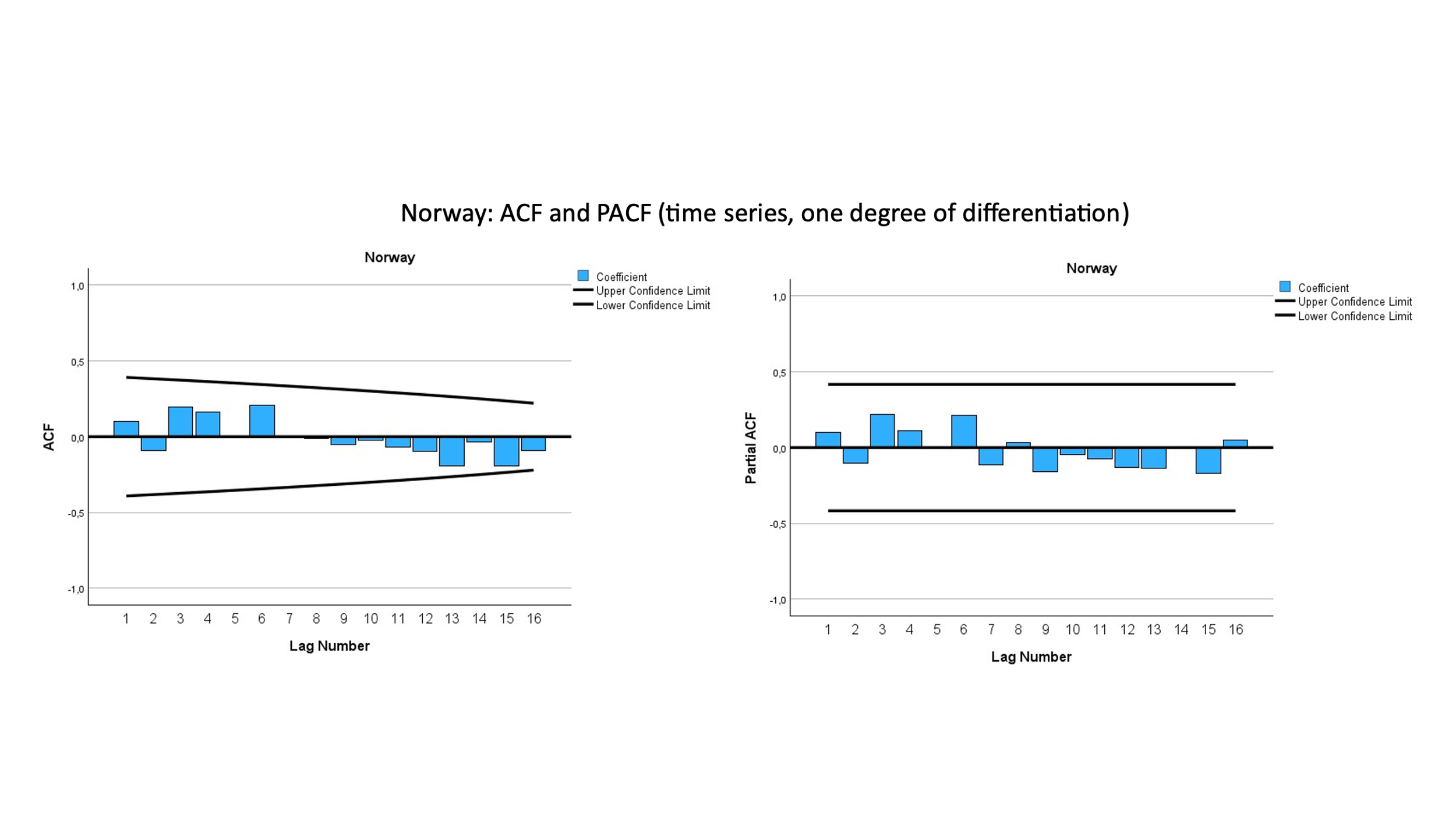


***Fig. S86:*** *Depicted are the ACF and PACF plots for the first-degree differentiation of the time series for Portugal.*


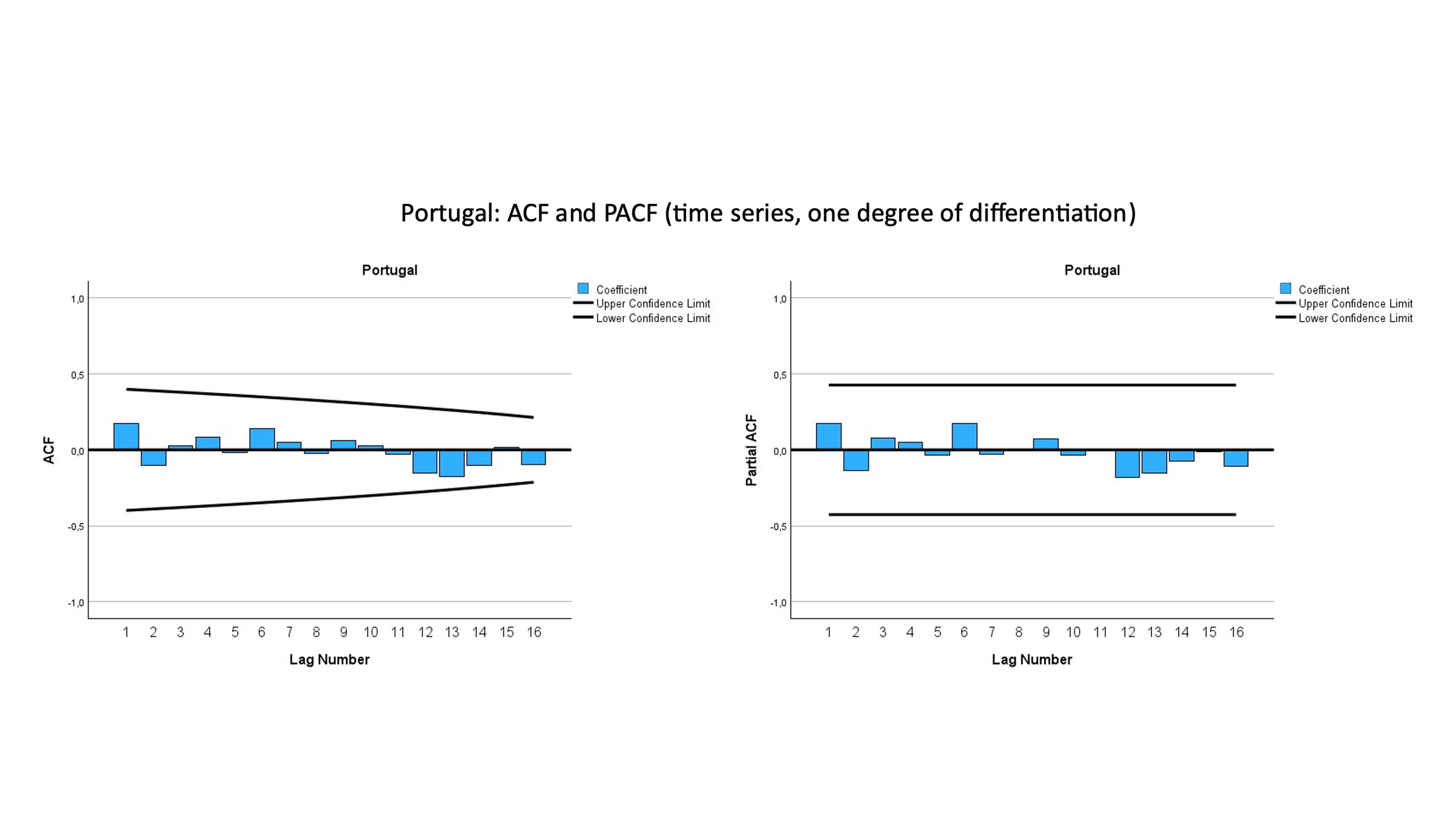


***Fig. S87:*** *Depicted are the ACF and PACF plots for the first-degree differentiation of the time series for Slovakia.*


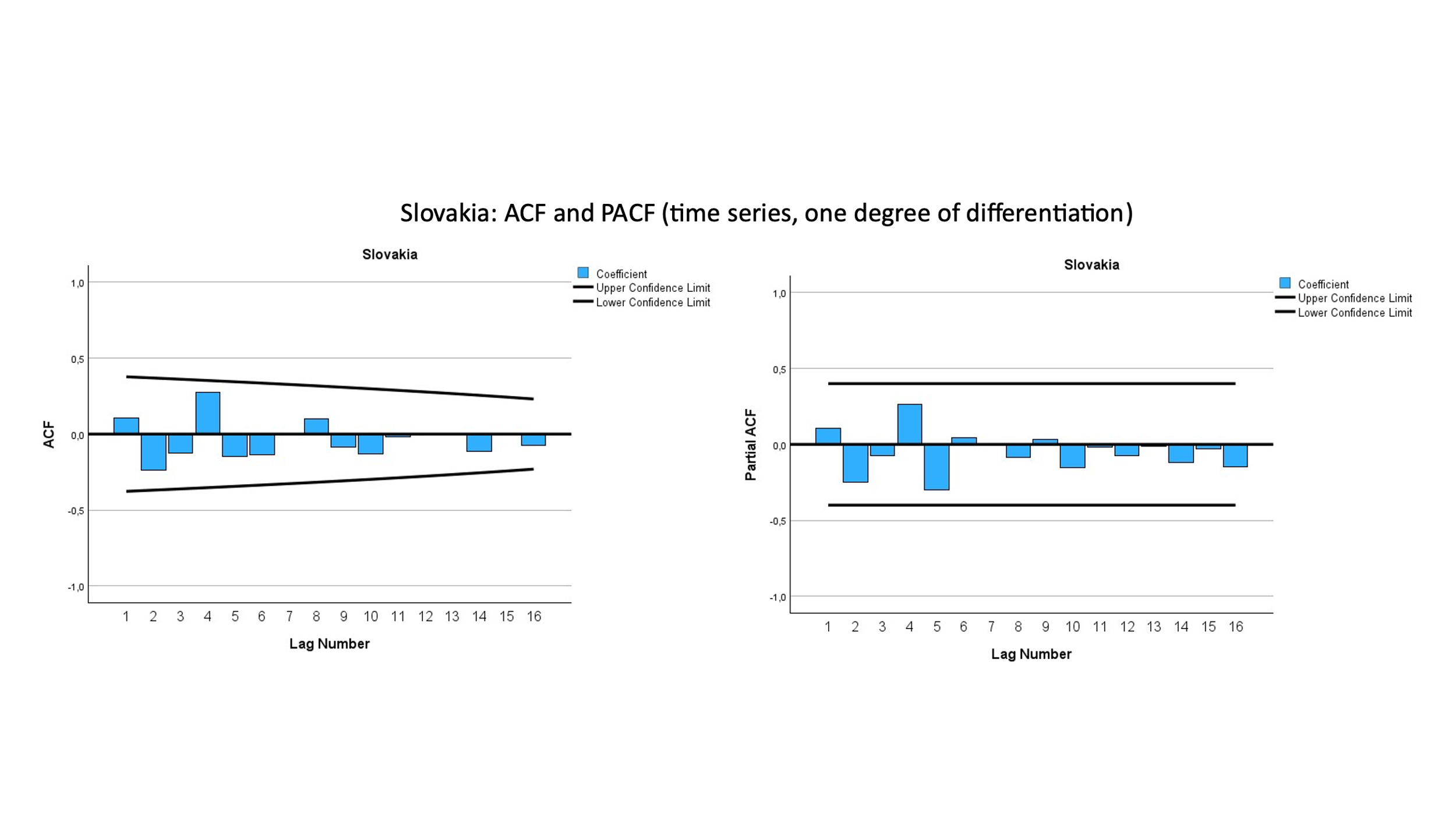


***Fig. S88:*** *Depicted are the ACF and PACF plots for the first-degree differentiation of the time series for Slovenia.*


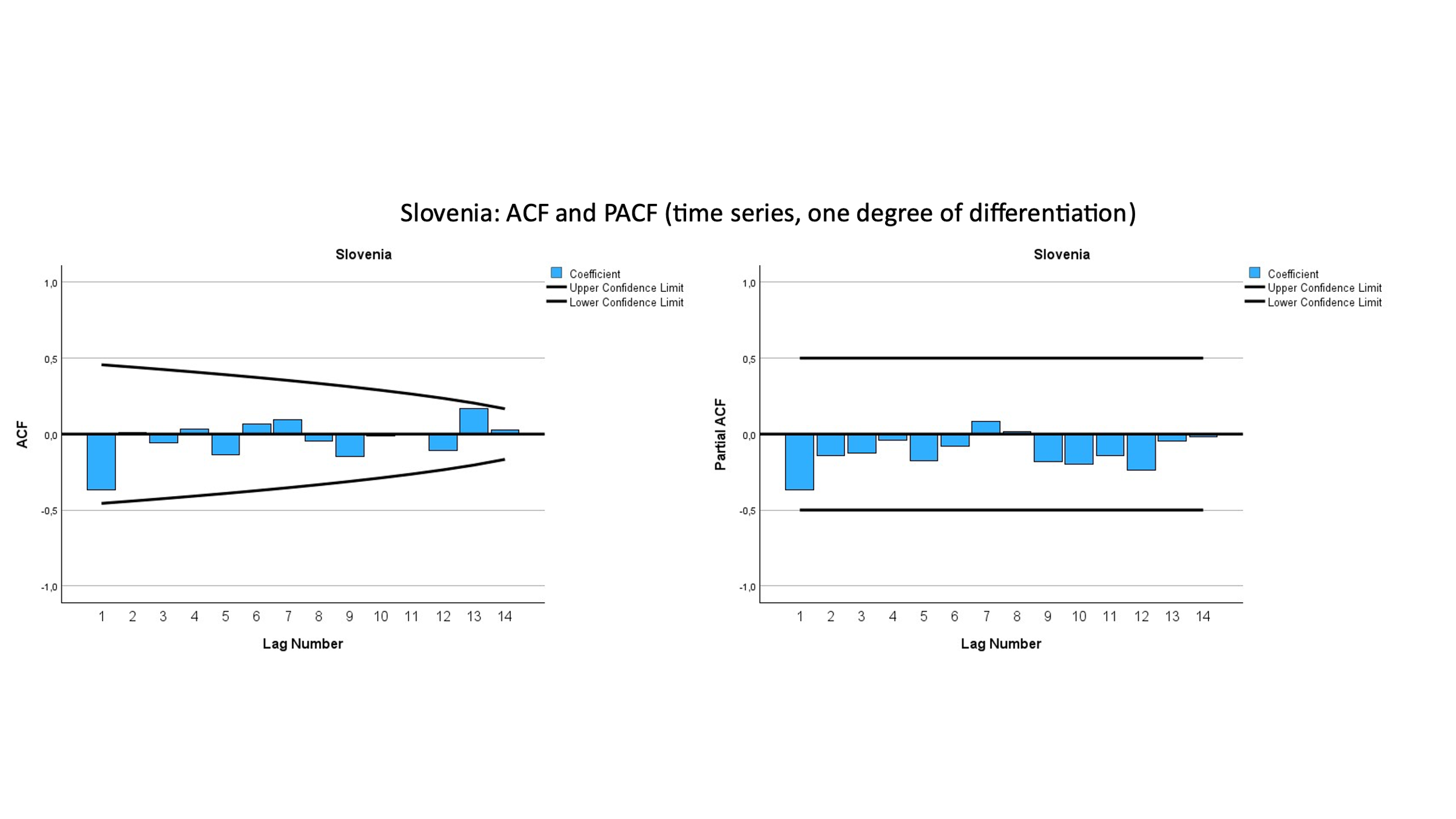


***Fig. S89:*** *Depicted are the ACF and PACF plots for the first-degree differentiation of the time series for Spain.*


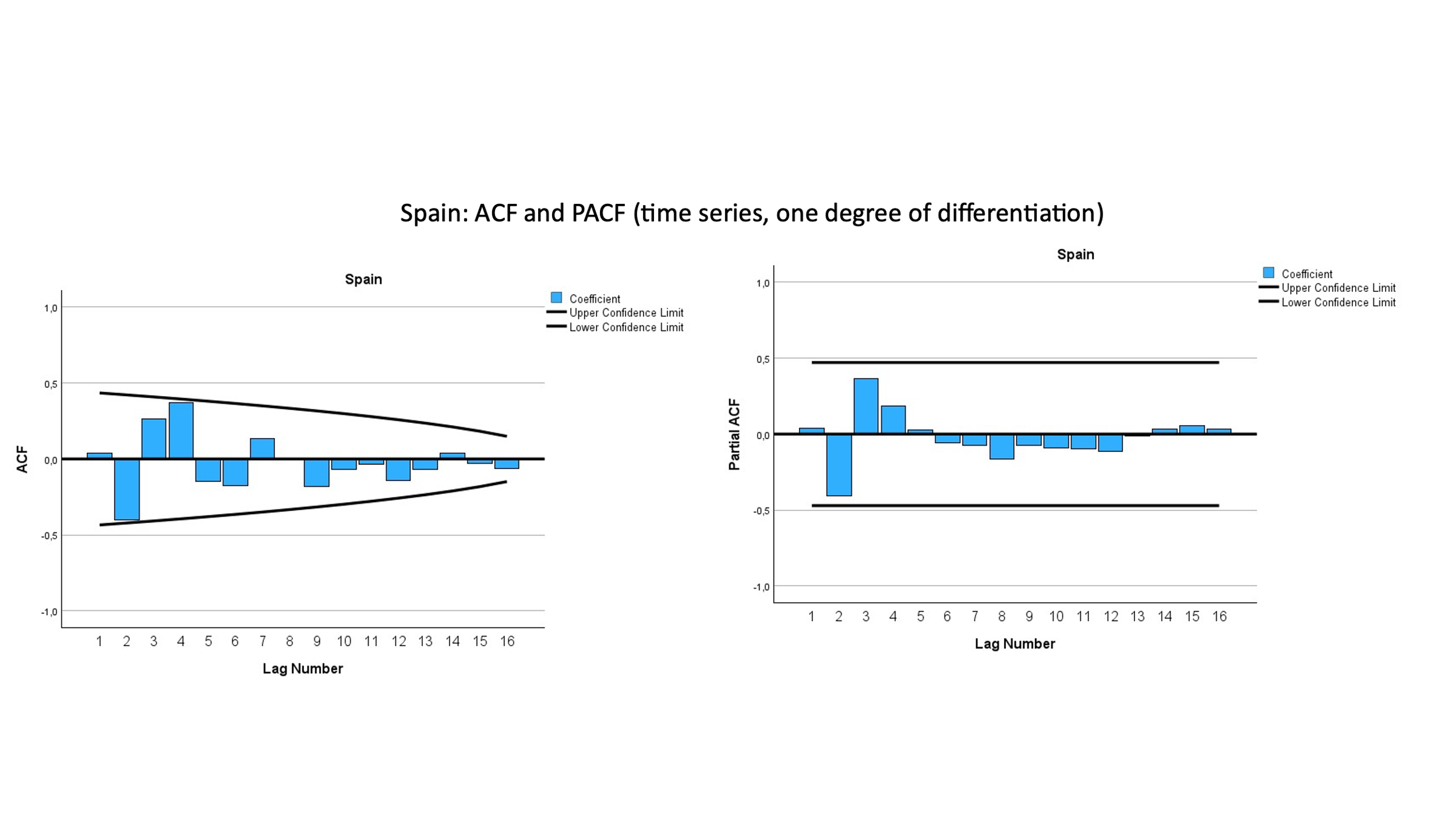


***Fig. S90:*** *Depicted are the ACF and PACF plots for the first-degree differentiation of the time series for Sweden.*


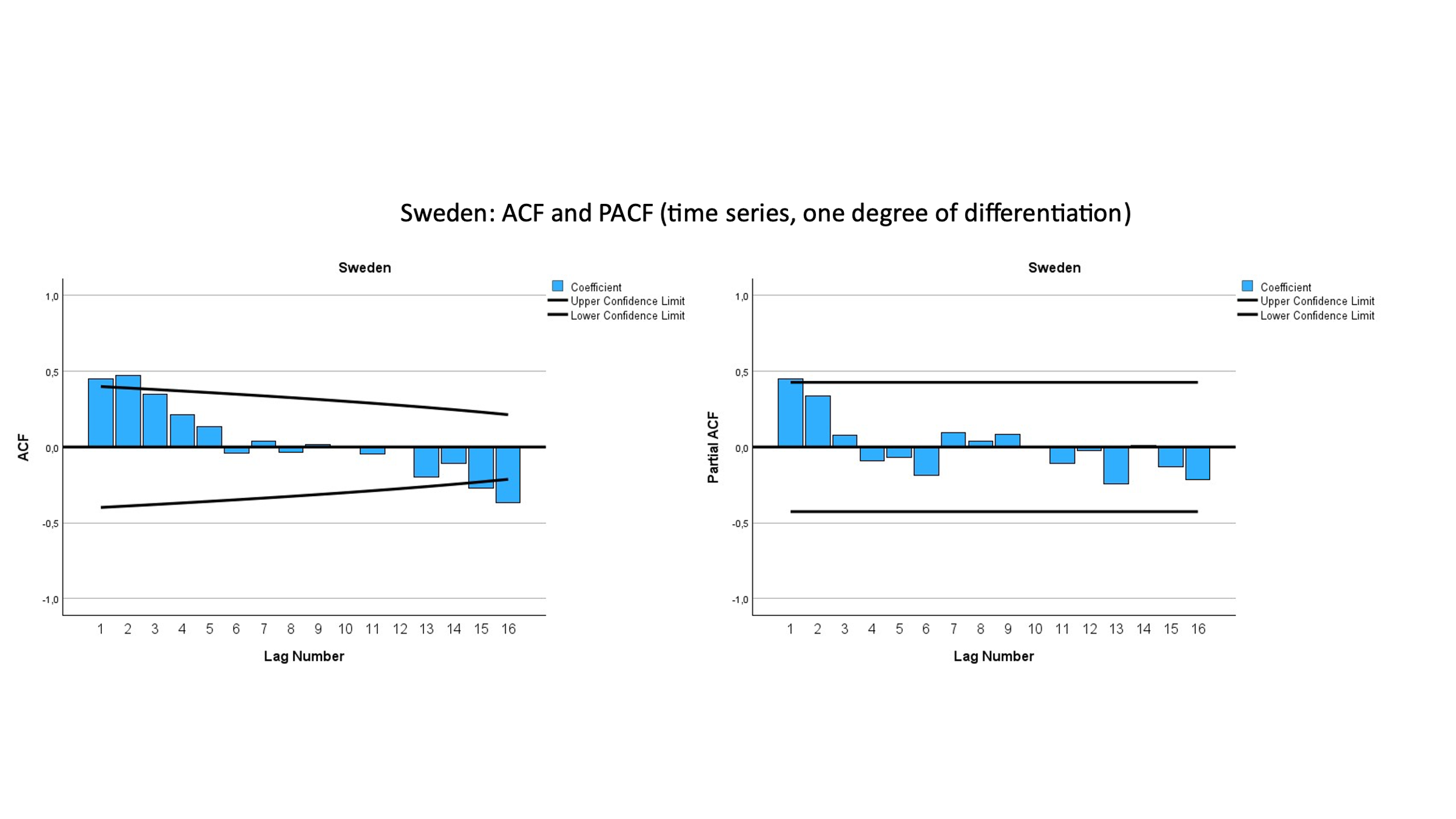


***Fig. S91:*** *Depicted are the ACF and PACF plots for the first-degree differentiation of the time series for Turkey.*


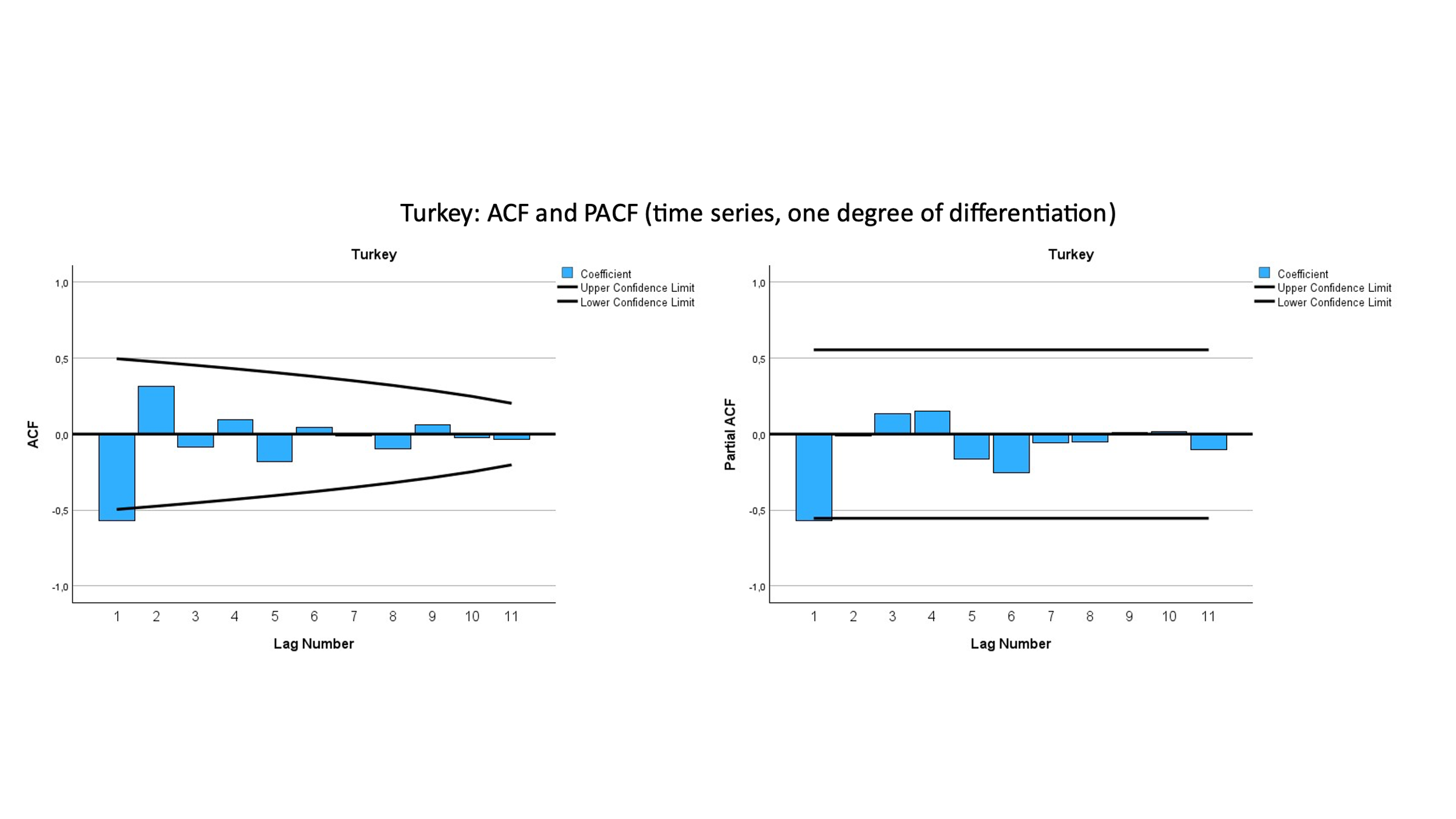


***Fig. S92:*** *Depicted are the ACF and PACF plots for the first-degree differentiation of the time series for the United Kingdom.*


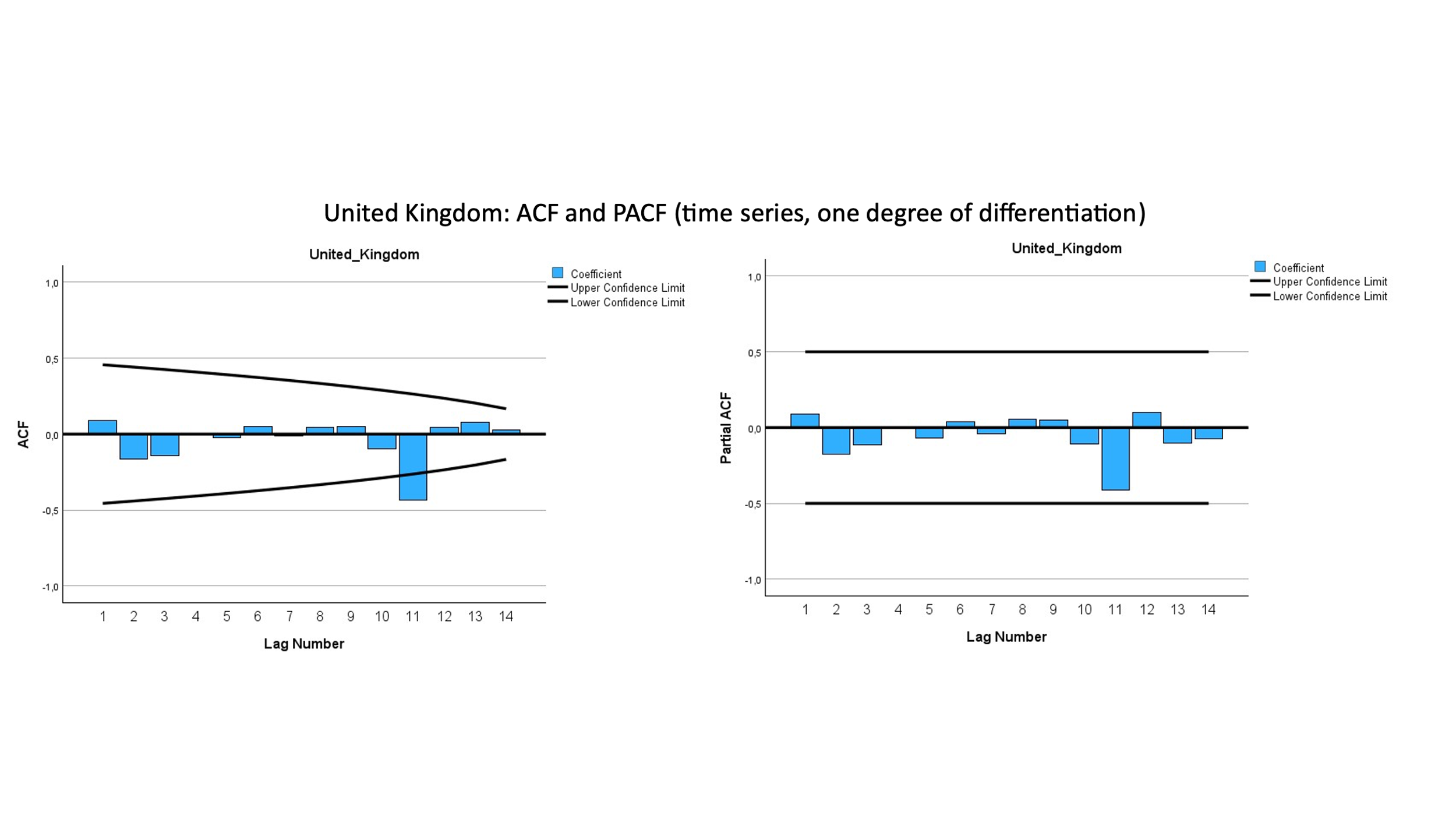


***Fig. S93:*** *Depicted are the ACF and PACF plots for the first-degree differentiation of the time series for Croatia.*


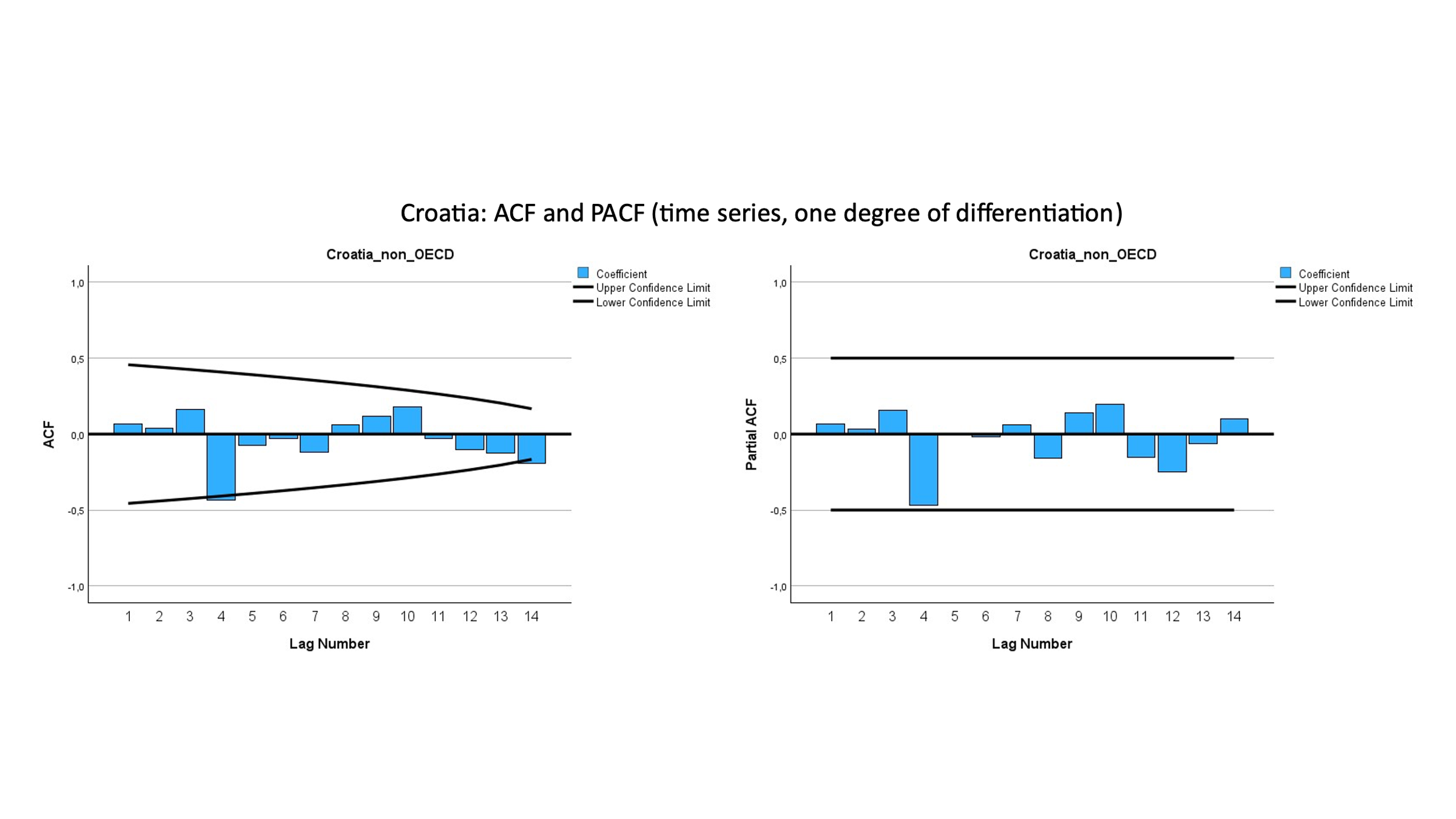


***Fig. S94:*** *Depicted are the ACF and PACF plots for the second-degree differentiation of the time series for Australia.*


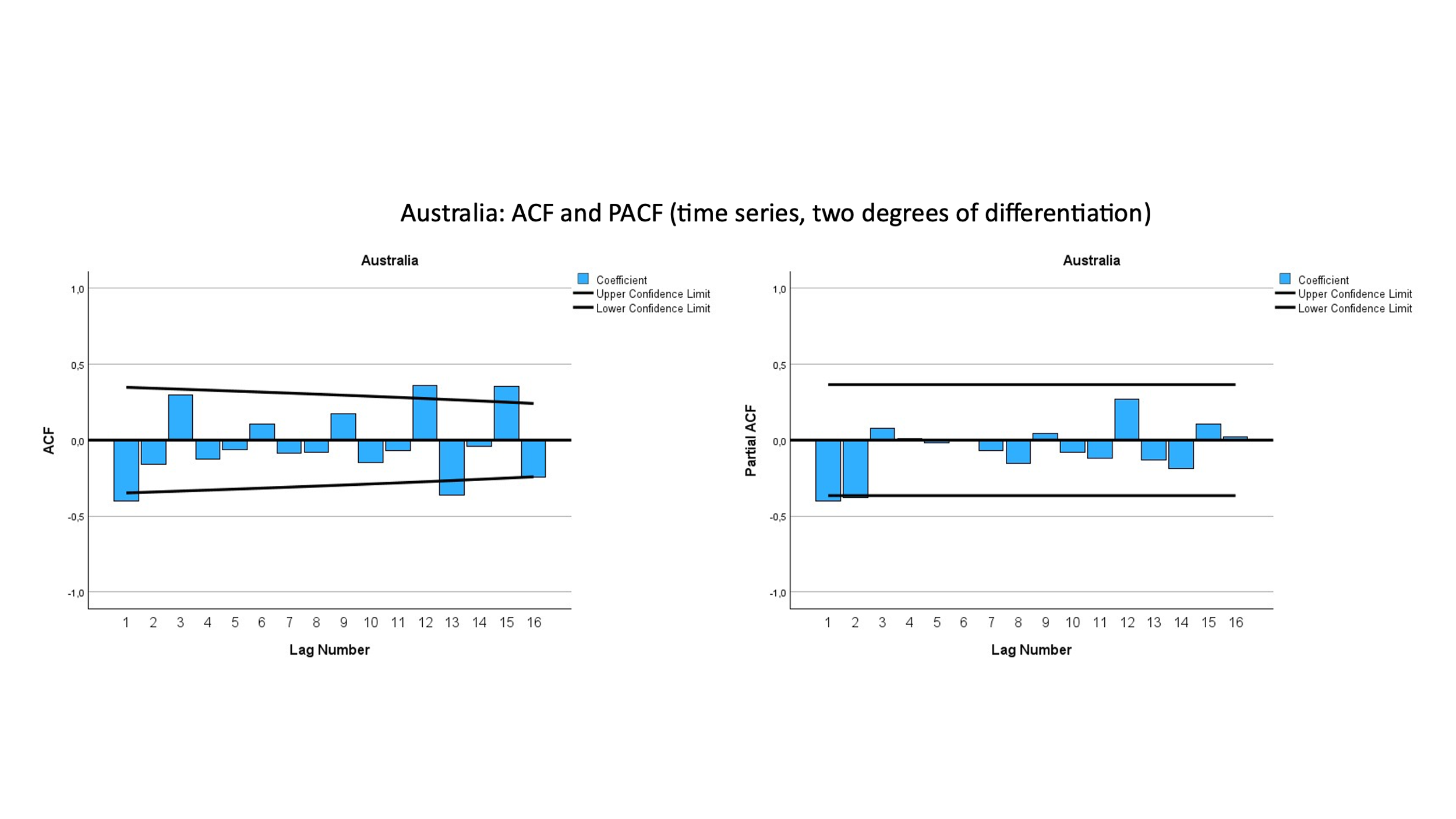


***Fig. S95:*** *Depicted are the ACF and PACF plots for the second-degree differentiation of the time series for Austria.*


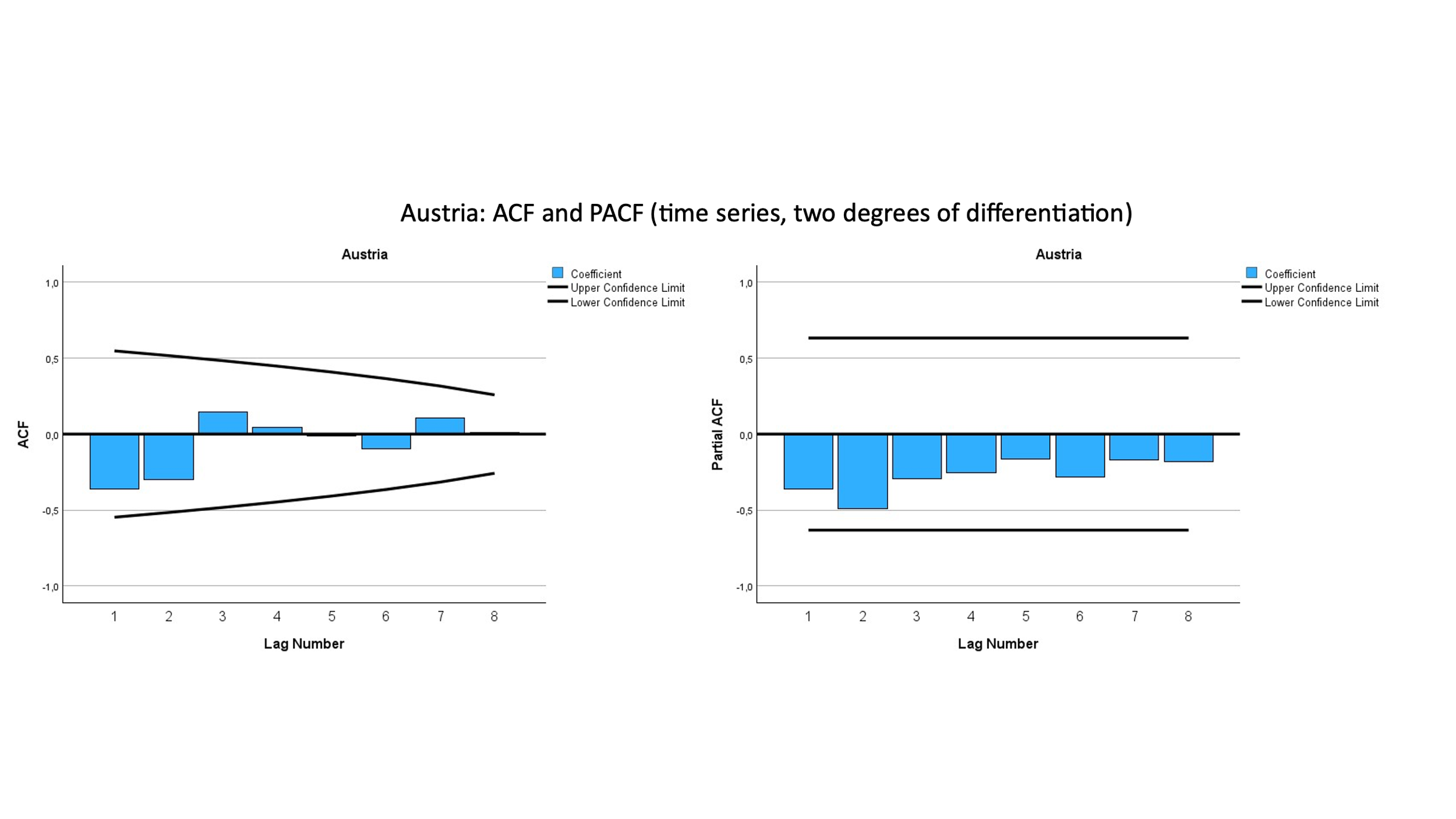


***Fig. S96:*** *Depicted are the ACF and PACF plots for the second-degree differentiation of the time series for Belgium.*


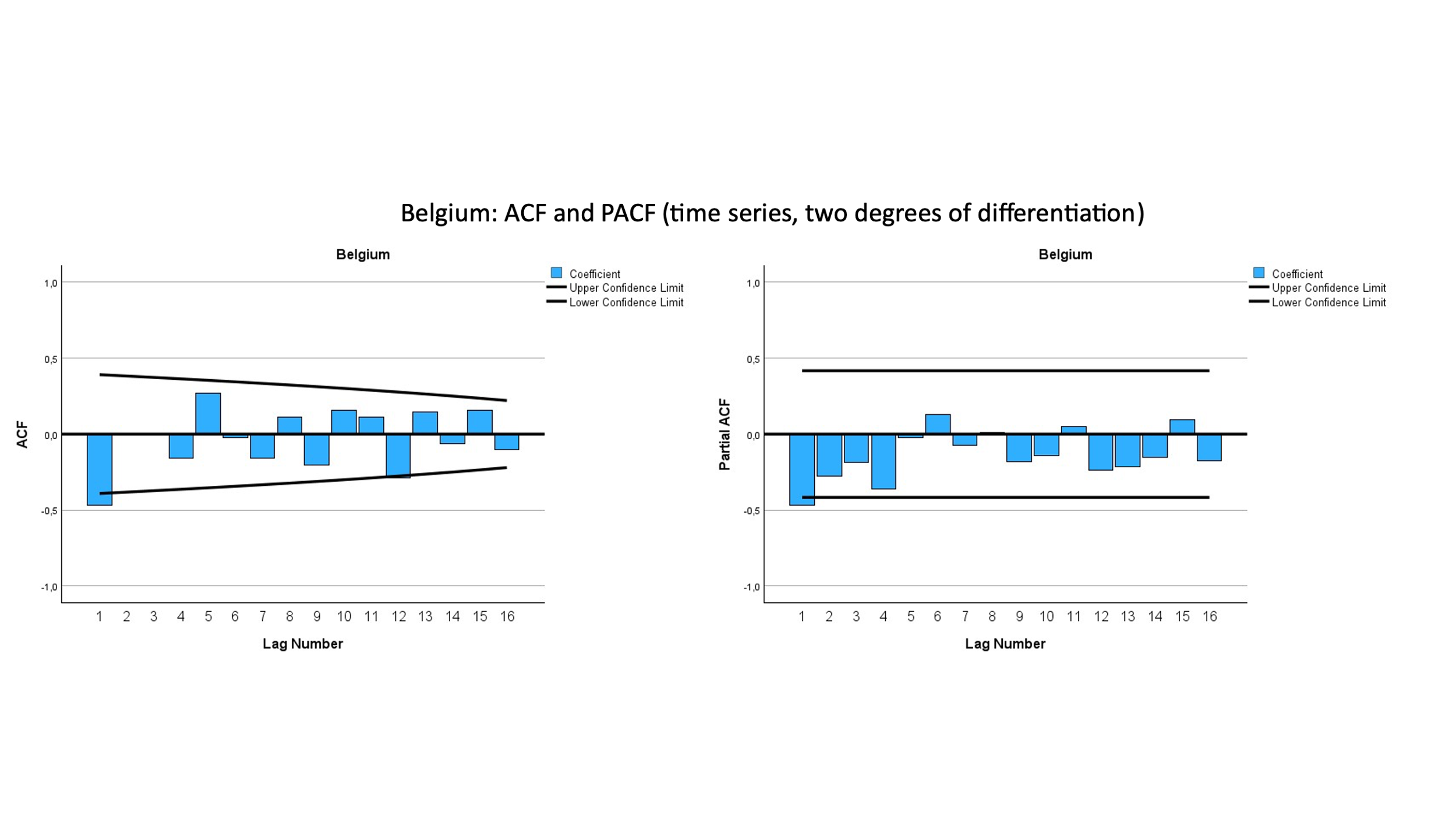


***Fig. S97:*** *Depicted are the ACF and PACF plots for the second-degree differentiation of the time series for Canada.*


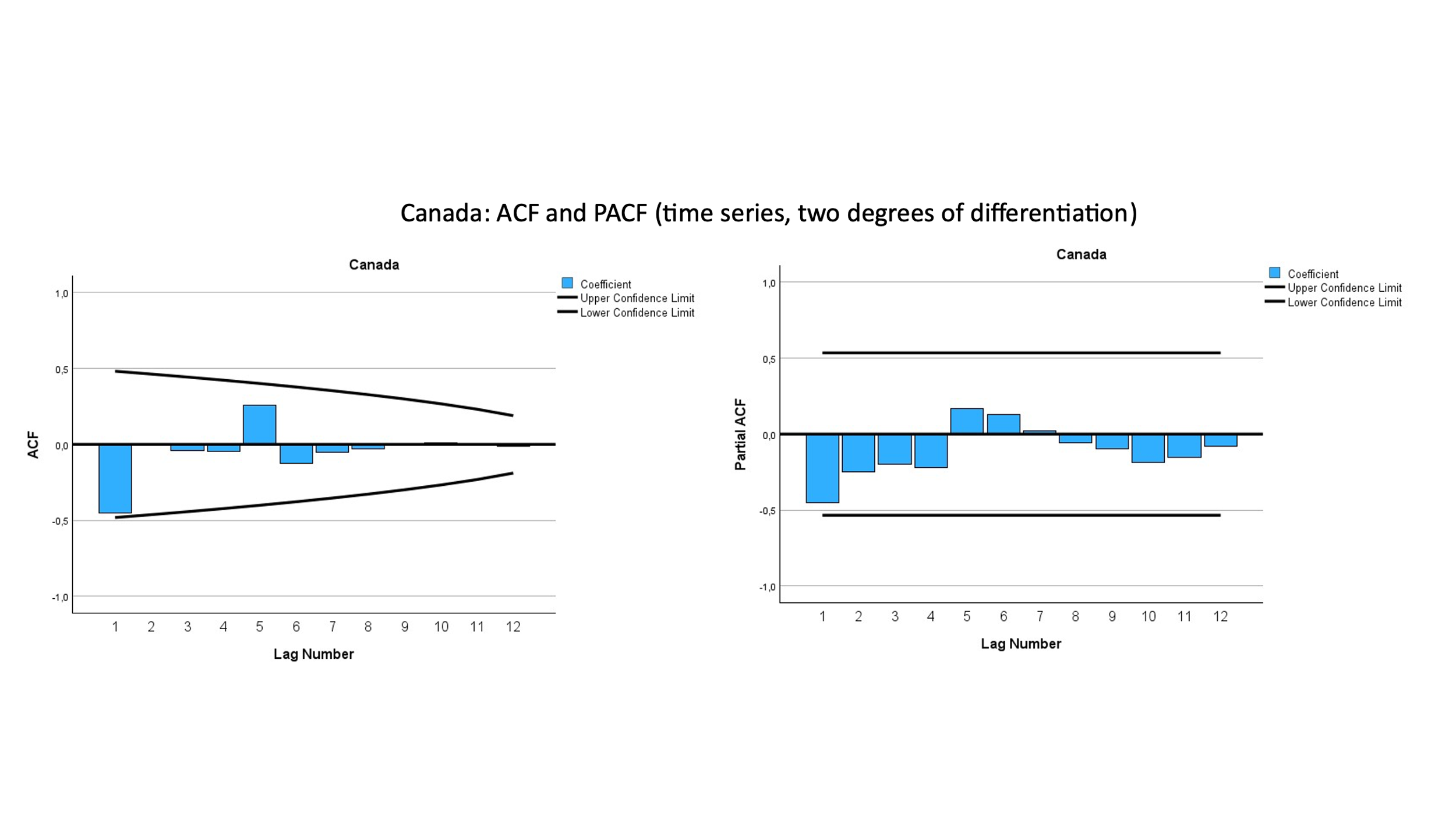


***Fig. S98:*** *Depicted are the ACF and PACF plots for the second-degree differentiation of the time series for Chile.*


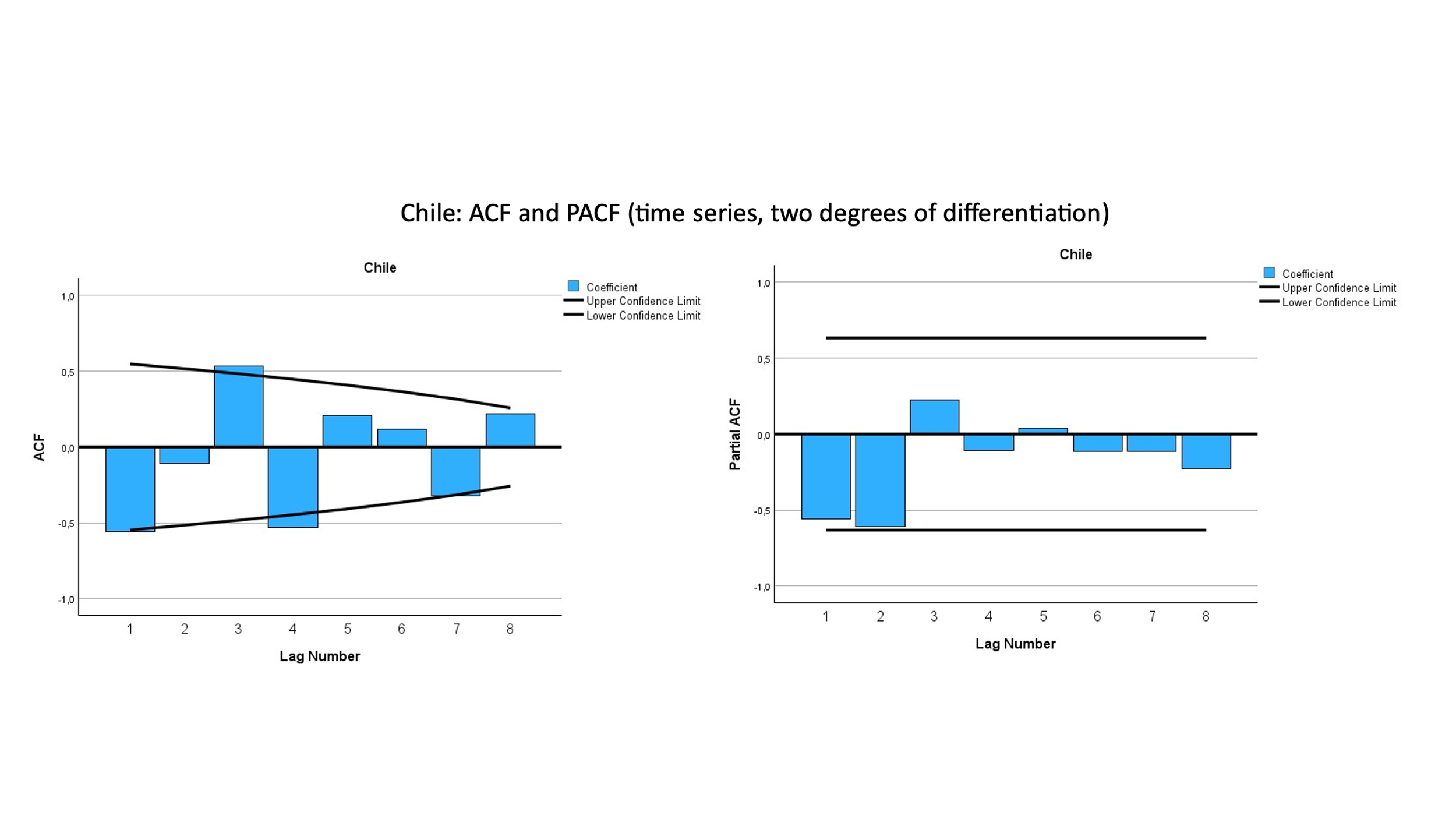


***Fig. S99:*** *Depicted are the ACF and PACF plots for the second-degree differentiation of the time series for Costa Rica.*


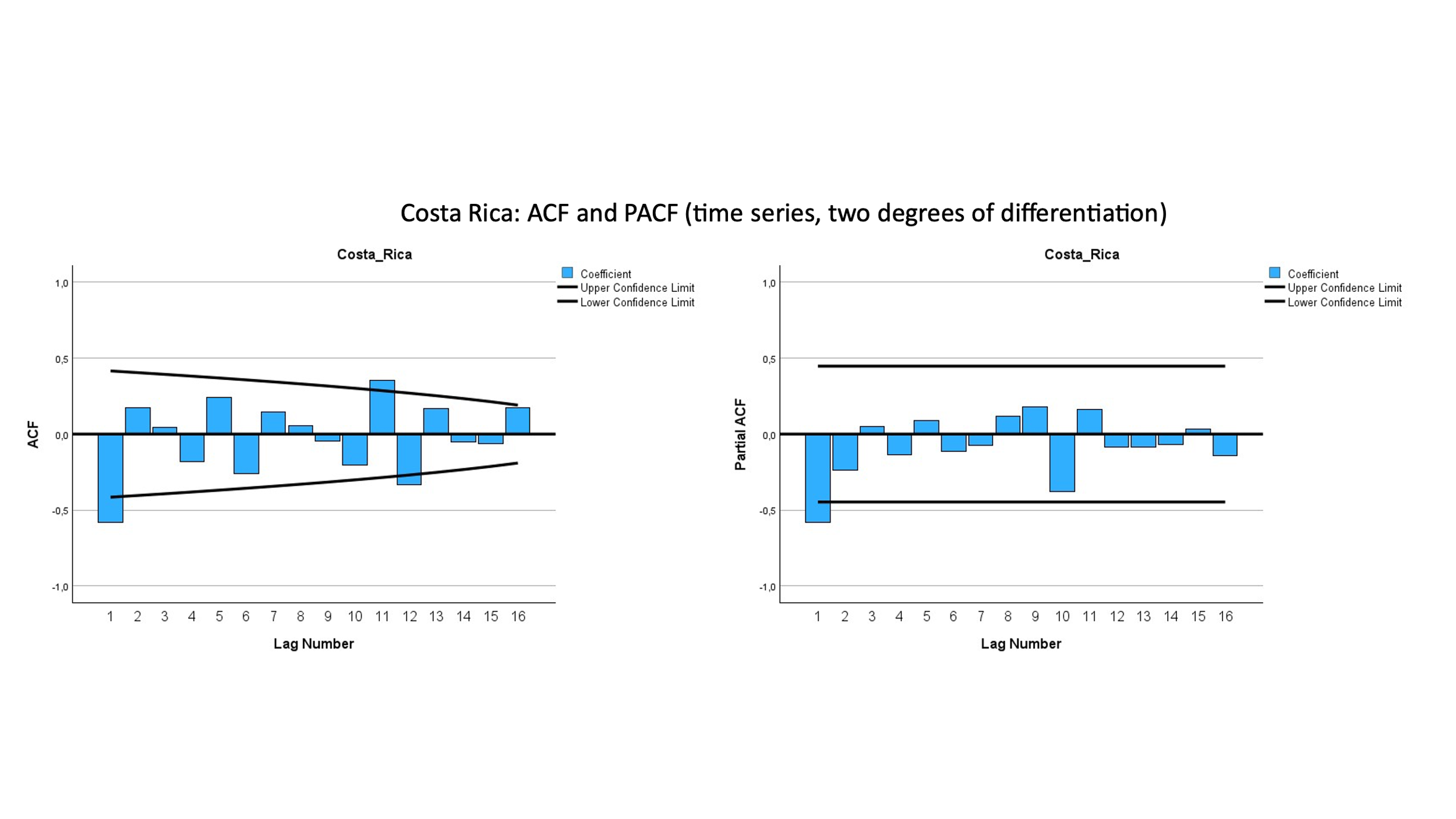


***Fig. S100:*** *Depicted are the ACF and PACF plots for the second-degree differentiation of the time series for Czechia.*


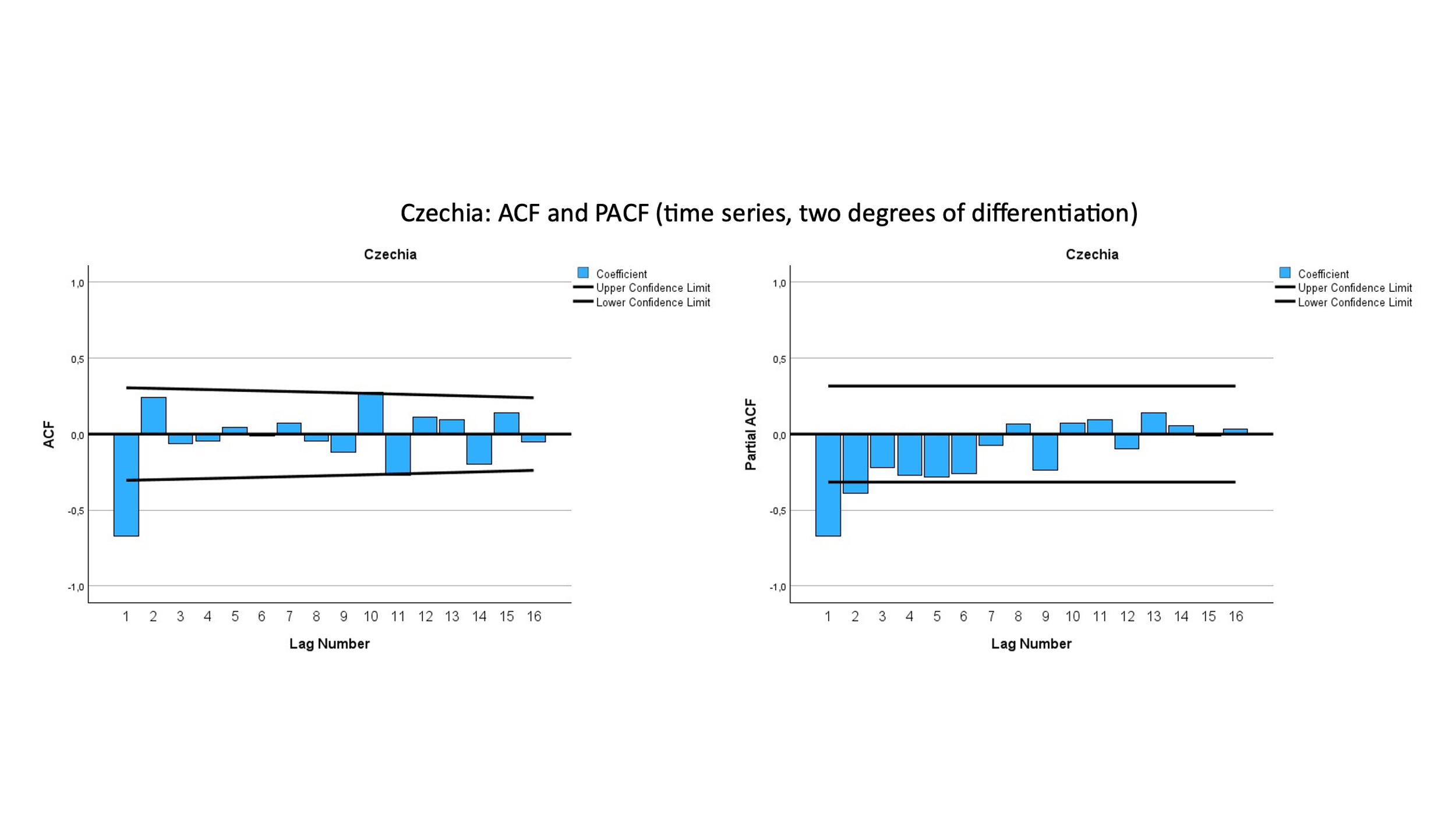


***Fig. S101:*** *Depicted are the ACF and PACF plots for the second-degree differentiation of the time series for Denmark*

***Fig. S102:*** *Depicted are the ACF and PACF plots for the second-degree differentiation of the time series for Estonia.*

***Fig. S103:*** *Depicted are the ACF and PACF plots for the second-degree differentiation of the time series for Finland.*

***Fig. S104:*** *Depicted are the ACF and PACF plots for the second-degree differentiation of the time series for France.*

***Fig. S105:*** *Depicted are the ACF and PACF plots for the second-degree differentiation of the time series for Germany.*

***Fig. S106:*** *Depicted are the ACF and PACF plots for the second-degree differentiation of the time series for Greece.*

***Fig. S107:*** *Depicted are the ACF and PACF plots for the second-degree differentiation of the time series for Hungary.*

***Fig. S108:*** *Depicted are the ACF and PACF plots for the second-degree differentiation of the time series for Iceland.*

***Fig. S109:*** *Depicted are the ACF and PACF plots for the second-degree differentiation of the time series for Israel.*

***Fig. S110:*** *Depicted are the ACF and PACF plots for the second-degree differentiation of the time series for Italy.*

***Fig. S111:*** *Depicted are the ACF and PACF plots for the second-degree differentiation of the time series for Korea.*

***Fig. S112:*** *Depicted are the ACF and PACF plots for the second-degree differentiation of the time series for Latvia.*

***Fig. S113:*** *Depicted are the ACF and PACF plots for the second-degree differentiation of the time series for Lithuania.*

***Fig. S114:*** *Depicted are the ACF and PACF plots for the second-degree differentiation of the time series for Luxembourg.*

***Fig. S115:*** *Depicted are the ACF and PACF plots for the second-degree differentiation of the time series for the Netherlands.*

***Fig. S116:*** *Depicted are the ACF and PACF plots for the second-degree differentiation of the time series for Norway.*

***Fig. S117:*** *Depicted are the ACF and PACF plots for the second-degree differentiation of the time series for Portugal.*

***Fig. S118:*** *Depicted are the ACF and PACF plots for the second-degree differentiation of the time series for Slovakia.*

***Fig. S119:*** *Depicted are the ACF and PACF plots for the second-degree differentiation of the time series for Slovenia.*

***Fig. S120:*** *Depicted are the ACF and PACF plots for the second-degree differentiation of the time series for Spain.*

***Fig. S121:*** *Depicted are the ACF and PACF plots for the second-degree differentiation of the time series for Sweden.*

***Fig. S122:*** *Depicted are the ACF and PACF plots for the second-degree differentiation of the time series for Turkey.*

***Fig. S123:*** *Depicted are the ACF and PACF plots for the second-degree differentiation of the time series for the United Kingdom.*

***Fig. S124:*** *Depicted are the ACF and PACF plots for the second-degree differentiation of the time series for Croatia.*
